# Pairwise alignment of nucleotide sequences using maximal exact matches

**DOI:** 10.1186/s12859-019-2827-0

**Published:** 2019-05-21

**Authors:** Arash Bayat, Bruno Gaëta, Aleksandar Ignjatovic, Sri Parameswaran

**Affiliations:** 1 School of Computer Science and Engineering, University of New South Wales (UNSW), Sydney, 2052 Australia; 2 Health and Biosecurity, CSIRO, 53/11 Julius Ave, North Ryde, Sydney, 2113 Australia

**Keywords:** Sequence alignment, Dynamic programming, Affine-gap penalty

## Abstract

**Background:**

Pairwise alignment of short DNA sequences with affine-gap scoring is a common processing step performed in a range of bioinformatics analyses. Dynamic programming (i.e. Smith-Waterman algorithm) is widely used for this purpose. Despite using data level parallelisation, pairwise alignment consumes much time. There are faster alignment algorithms but they suffer from the lack of accuracy.

**Results:**

In this paper, we present *MEM-Align*, a fast semi-global alignment algorithm for short DNA sequences that allows for affine-gap scoring and exploit sequence similarity. In contrast to traditional alignment method (such as Smith-Waterman) where individual symbols are aligned, *MEM-Align* extracts Maximal Exact Matches (MEMs) using a bit-level parallel method and then looks for a subset of MEMs that forms the alignment using a novel dynamic programming method. *MEM-Align* tries to mimic alignment produced by Smith-Waterman. As a result, for 99.9% of input sequence pair, the computed alignment score is identical to the alignment score computed by Smith-Waterman. Yet *MEM-Align* is up to 14.5 times faster than the Smith-Waterman algorithm. Fast run-time is achieved by: (a) using a bit-level parallel method to extract MEMs; (b) processing MEMs rather than individual symbols; and, (c) applying heuristics.

**Conclusions:**

*MEM-Align* is a potential candidate to replace other pairwise alignment algorithms used in processes such as DNA read-mapping and Variant-Calling.

**Electronic supplementary material:**

The online version of this article (10.1186/s12859-019-2827-0) contains supplementary material, which is available to authorized users.

## Background

Biological sequence alignment [1] is about finding similarities and differences between sequences. The term alignment covers a broad range of different processes. Seed-and-extend alignment method is a popular technique for aligning reads to the reference-genome. This technique is used in DNA read-mappers such as BWA [2, 3] and Bowtie [4, 5]. In the seed-and-extend technique, small subsequences of a read (called seeds) are searched in the reference-genome to find candidate regions. Once a rough alignment is identified (seeding-step), the read is typically aligned to all candidate regions using a dynamic programming algorithm (extending-step)

Seed-and-extend method is also used in similarity search tools such as BLAST [6], BLAT [7] and MUMmer [8] as well as PatternHunter [9] and PatternHunter II [10]. In order to search for the seed in the reference-genome, some methods such as BWA and Bowtie use a suffix-tree-based structure called FM-Index [11] while others such as BLAST and SNAP [12] use a hash-table index of fixed size k-mers (subsequences of length *k*).

The seeding-step varies from program to program. For example, BWA-MEM [3] looks for Maximal Exact Matches (MEMs) and MUMmer looks for Maximal Unique Matches (MUMs). GEM [13] limits the seed size to have at least *n*+1 non-overlaping seed to find all alignments with up to *n* errors. Another technique is to use spaced-seeding [14] that is used in PatternHunter and PatternHunter II.

The focus of this paper is the extending-step and not the seeding-step. Typically, in the extending-step, dynamic programming is used. Dynamic programming alignment could produce global [15], local [16] or semi-global [1] alignment. Such an extending-step could also implement different scoring systems such as edit-distance [17] scoring or affine-gap [18] scoring.

Most DNA read-mappers [2–5, 19] use a derivative of the Smith-Waterman algorithm which uses affine-gap scoring to find a semi-global alignment. Such implementations of the Smith-Waterman algorithm can be found in [20–23]. These implementations exploit data-level parallelisation (SIMD) instructions of Intel processors (SSE) to further speed up the alignment process.

Not all DNA read-mappers use the above extending-step. For example, GEM and SNAP use modified version of Gene Myers [24] and Ukkonen [25] algorithms respectively for their extending-step. Both Gene Myers and Ukkonen algorithms are edit-distance based dynamic programming alignment. There are other alignment algorithms which do not use the conventional dynamic programming technique. For example, PatternHunter uses its own custom-made extending-step. Another example is a greedy approach for aligning DNA sequences introduced in [26]. The Smith-Waterman algorithm is also accelerated on Graphic Processing Units (GPUs) [27] and Field Programmable Gate Arrays (FPGAs) [28, 29]. However, the scope of this paper is the use in conventional computing platform (CPUs), though they can be extended for use in FPGAs.

**Fig. 1:**
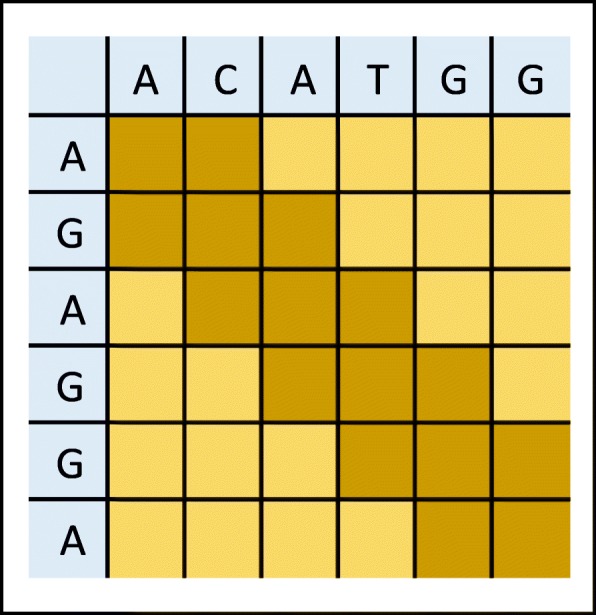
Traditional dynamic programming for pairwise alignment. For sequences of length *S* symbols there are *O*(*S*^2^) entry in the table to be processed

In this paper, we present a dynamic programming alignment algorithm called *DP-MEM* to be used in the extending-step of the DNA read-mapper tools. Our algorithm produces a semi-global alignment in which the first few bases or the last few bases can be excluded (clipped) from the alignments (not affecting alignment score). Also, the proposed algorithm implements the affine-gap scoring model. Unlike previous dynamic programming alignment algorithms which work on individual bases of the sequences (see Fig. 1, *DP-MEM* works on the MEMs which exist between a pair of short sequences.

*DP-MEM* requires all MEMs to be extracted from a given pair of sequences. To reduce the overhead of extracting MEMs, we introduce a bitwise parallel method to quickly extract all MEMs which exist between a pair of short sequences. *DP-MEM* requires MEMs to be sorted. Thus, a linear counting sort algorithm is used to speed up the sorting of extracted MEMs (Additional file 1: Section I). *DP-MEM* along with our proposed MEM extraction method and several heuristic optimisations introduced in this paper form a fast and near-accurate alignment method called *MEM-Align*.

We motivate the work in this paper with the following simplified example. Consider aligning the following sentences:

- my dog is friendly whenever playing in the park
- all dogs are friends when playing in parks

It is clear that matching words such as “dog”, “friend” and “park” rather than individual letters such as “m”, “y” and “d” would speed up the alignment process. *MEM-Align* uses MEMs (similar to matching words in sentences) to align sequences. *MEM-Align* is suitable for aligning similar sequences (i.e. a read and its candidate region in the reference-genome) where the number of existing MEMs are significantly smaller than the number of bases in the sequences.

**Fig. 2:**
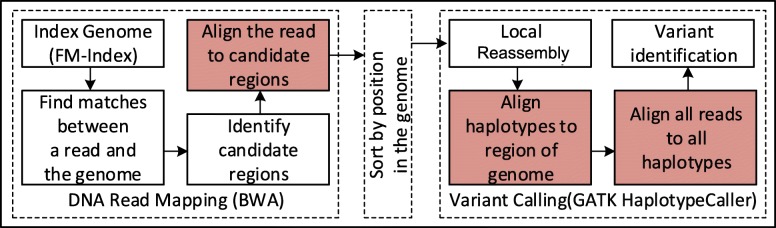
Possible applications of *MEM-Align* as a pairwise alignment algorithm for short DNA sequences. Use of pairwise alignment in a simple variant calling pipeline. Highlighted blocks use pairwise alignment

Figure 2 identifies possible applications of *MEM-Align*. In addition to DNA read-mapping software, *MEM-Align* can be used in Variant-Callers such as GAKT HaplotypeCaller [30] and Platypus [31] to align haplotypes to reference-genome and reads to haplotypes. *MEM-Align* can be used for all applications where a pair of short and similar DNA sequences are aligned.

To show the efficacy of our work, we compare the speed and accuracy results to an accelerated implementation of the Smith-Waterman algorithm as well as algorithms proposed by Gene Myers and Ukkonen.

Note that, Maximal Exact Matches has been used in the seeding-step [3]. Although *MEM-Align* uses MEMs, it is not a seeding method and should not be compared with the entire seed-and-extend alignment. *MEM-Align* should be considered a replacement for algorithms used in the extending-step.

## Method

### Approach

**Fig. 3:**
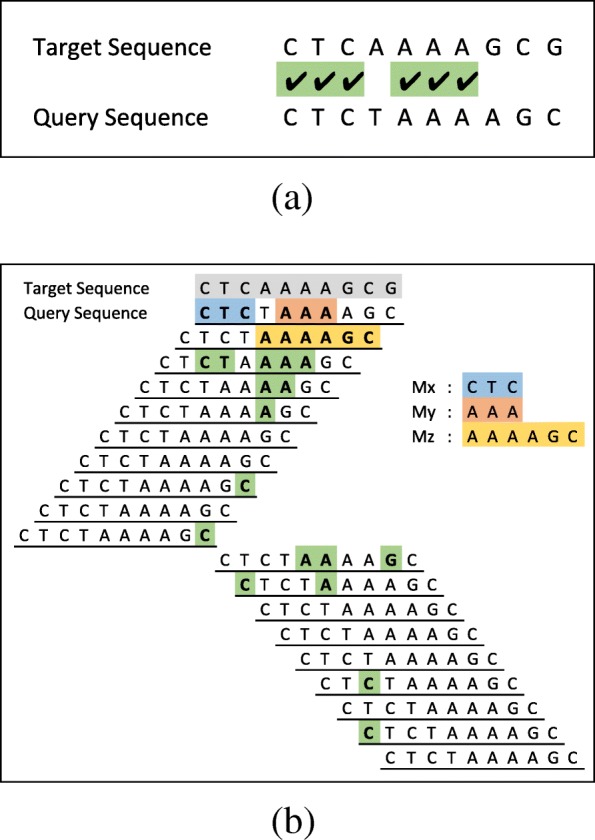
MEM extraction using shift and compare operations. **a** Identify MEMs by direct comparison of sequences. **b** The query is shifted to left until last symbol in query sequence is aligned to the first symbol in target sequence. Then the query sequence is shifted to right until the first symbol in query sequence is aligned to the last symbol of target sequence. After each shift the overlapping part of the query and target sequences are compared to identify new MEMs. Three of MEMs (*M*_*x*_,*M*_*y*_ and *M*_*z*_) are highlighted with different colours to be used for later explanation

In our proposed algorithm, the first step towards aligning sequences is to extract MEMs between sequences by directly comparing them. Figure 3a is an example which compares a target and a query sequence where CTC and AAA are two MEMs identified by the comparison. Each group of continuous identical symbols in the comparison, result in a MEM even if it is composed of only a single matching symbol. In order to extract all MEMs between the sequences, the query sequence must be shifted all the way to the right and to the left one symbol at a time (see Fig. 3b). After each shift, the comparison step must be repeated to identify new MEMs. For example, the third line in Fig. 3b represents the case where the query sequence is shifted to the right one symbol and is compared with the target sequence. The result of the comparison identifies AAAAGC as a new MEM. All other MEMs extracted by shift and compare operations are also highlighted in Fig. 3b. Three of the MEMs (*M*_*x*_,*M*_*y*_ and *M*_*z*_) are highlighted with different colours to be used for later explanation.

In the affine-gap scoring model, the alignment score *AS* is computed using Eq. 1 where *N*_*m*_ is number of matches each receiving a match score of *R*_*m*_,*N*_*x*_ is number of mismatches each receiving a mismatch penalty of *P*_*x*_,*N*_*o*_ is number of gap openings each receiving a gap open penalty of *P*_*o*_ and *N*_*g*_ is total length of all gaps, each gap receiving a gap extension penalty of *P*_*g*_. There would be a gap opening for each group of continuous gap. For example, if there are two gaps in the alignment, where the length of the first gap is three and the length of the second gap is four, then there are two gap openings (*N*_*o*_=2) and the total length of the gap is seven (*N*_*g*_=3+4=7).

1 $$ {}AS = (N_{m} \times R_{m}) - ((N_{x} \times P_{x}) + (N_{o} \times P_{o}) + (N_{g} \times P_{g}))  $$

**Fig. 4:**
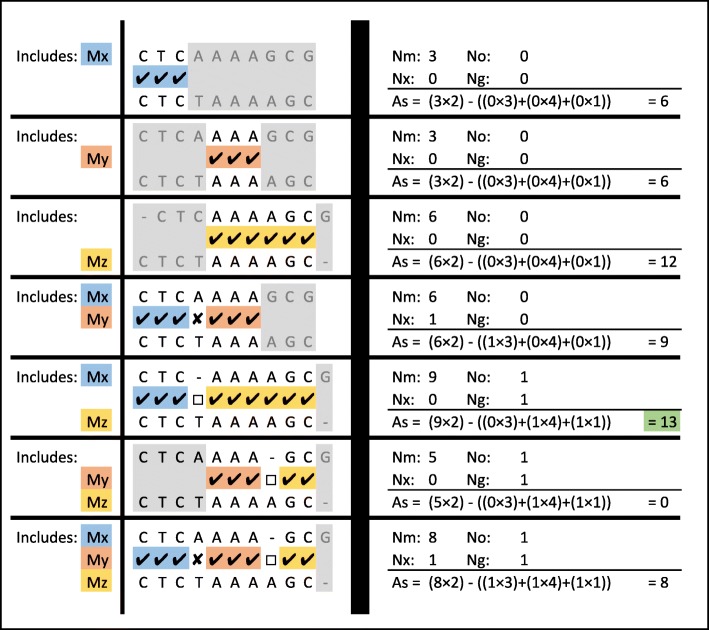
All possible combination of MEMs in the alignment

Given the list of all MEMs, the alignment can be computed using partial alignments. For example, consider MEMs *M*_*x*_, *M*_*y*_ and *M*_*z*_ in Fig. 3b. The partial alignments made by taking different combinations of *M*_*x*_, *M*_*y*_ and *M*_*z*_ along with the number of matches, mismatches and gaps, as well as the resulting alignment scores are shown in Fig. 4. The alignment that only includes *M*_*x*_ and *M*_*z*_ results in the highest alignment score. Note that, *M*_*y*_ and *M*_*z*_ overlap each other and when both are considered in the same alignment the overlap is excluded from *M*_*z*_. Considering all MEMs in Fig. 3b results in many more combinations where none of them achieves a higher score.

**Fig. 5:**
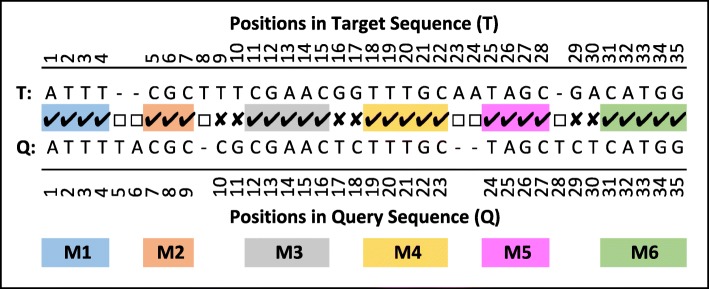
An example alignment with highlighted MEM

Examining all possible combination of MEMs would be exhaustive. In “Alignment algorithm” section we describe a novel dynamic programming algorithm *DP-MEM* that efficiently finds the best combination without considering all cases. *DP-MEM* needs to know which parts of the sequences match but not the actual symbols in the sequences. The input to *DP-MEM* is the positioning of MEMs in the target and in the query sequences which are obtained during the MEM extraction process described in “MEM extraction” section. How MEMs are represented with their positions and how the number of matches, mismatches and gaps are computed when MEMs are combined in an alignment are explained in the remainder of this section. Figure 5 is another example alignment with six MEMs (*M*_1_ to *M*_6_) that form the alignment between target sequence *T* and query sequence *Q*. For simplicity there is no overlap between MEMs in this example. Each MEM *M*_*i*_ is represented as a triplet of integer numbers: the starting positions in *T* and in *Q* (*ST*_*i*_ and *SQ*_*i*_ respectively) and its length (*L*_*i*_). The ending positions in *T* and in *Q* can be computed later (*Φ*_2*E*_ of Algorithm 2). Table 1 lists the length and the positioning of *M*_1_ to *M*_6_ in *T* and in *Q*.

**Table 1:** Starting and ending position of MEMs in Fig. 5

*M*_*i*_	*L*_*i*_	*S* *T*_*i*_	*S* *T*_*i*_	*B* *Q*_*i*_	*E* *Q*_*i*_
*M*_1_	4	1	4	1	4
*M*_2_	3	5	7	7	9
*M*_3_	5	11	15	12	16
*M*_4_	5	18	22	19	23
*M*_5_	4	25	28	24	27
*M*_6_	5	31	35	31	35

The number of mismatches and gaps between adjacent MEMs *M*_*i*_ and *M*_*j*_ (*M*_*i*_ is on the left of *M*_*j*_) is computed from their positioning in sequences. First, the distance between *M*_*i*_ and *M*_*j*_ in *T* and in *Q* denoted by ${LT}_{i}^{j}$ and ${LQ}_{i}^{j}$ respectively are computed (*Φ*_2*F*_ of Algorithm 2) then the number of mismatches between *M*_*i*_ and *M*_*j*_, denoted by $N_{x}^{i,j}$, is the minimum of ${LT}_{i}^{j}$ and ${LQ}_{i}^{j}$. The length of the gap between *M*_*i*_ and *M*_*j*_, denoted by $N_{g}^{i,j}$, is equal to the absolute value of ${LT}_{i}^{j}-{LQ}_{i}^{j}$ (*Φ*_2*G*_ of Algorithm 2). A negative value of ${LT}_{i}^{j}-{LQ}_{i}^{j}$ indicates an insertion (there are more symbols in the query sequence) and a positive value indicates a deletion (there are more symbols in the target sequence).

For example, consider *M*_2_ and *M*_3_ in Fig. 5 where there are three symbols between them in *T* (${LT}_{2}^{3}=3$) and only two symbols between them in *Q*$\left ({{LQ}_{2}^{3}=2}\right)$. This situation indicates two mismatches and a gap (deletion). Table 2 elaborates how the number of mismatches and gaps are computed for the example alignment in Fig. 5.

**Table 2:** Computing number of mismatches and gaps between MEMs in Fig. 5

*M*_*i*_	*M*_*j*_	${LT}_{i}^{j}$	${LQ}_{i}^{j}$	${LT}_{i}^{j}-{LD}_{i}^{j}$	Gaps	Mismatches
*M*_1_	*M*_2_	0	2	-2	2 insertion	0
*M*_2_	*M*_3_	3	2	1	1 deletion	2
*M*_3_	*M*_4_	2	2	0	0	2
*M*_4_	*M*_5_	2	0	2	2 deletion	0
*M*_5_	*M*_6_	2	3	-1	1 insertion	2

In case there are both mismatches and gaps between *M*_*i*_ and *M*_*j*_, all gaps are considered continuous to reduce the gap open penalty (only one gap open penalty is applied for a continues gap). Thus, for all adjacent MEMs that have gaps between them, only one gap open penalty is applied. The placement of mismatches and the only continuous gap is not important, as it would not affect the alignment score. We assume that the mismatch penalty is constant (this is usual for DNA sequences).

If there is an overlap between *M*_*i*_ and *M*_*j*_ either in the target sequence or in the query sequence, the overlap should be excluded from *M*_*j*_. The length of overlap ${MO}_{i}^{j}$ is the maximum of the length of overlap in target and in the query (*Φ*_2*B*_ of Algorithm 2). To exclude overlap, ${MO}_{i}^{j}$ should be added to *ST*_*j*_ and *SQ*_*j*_ and subtracted from *L*_*j*_ (*Φ*_2*D*_ of Algorithm 2).

### MEM extraction

**Fig. 6:**
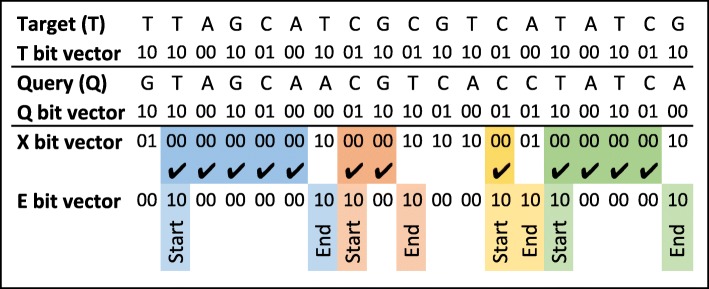
Representation of sequences with bit-vectors. XOR output (X) with highlighted MEMs. Edges bit-vector (E) identifies the start and the end of each MEMs

There are methods [3, 32] to extract maximal exact matches between lengthy sequences such as an entire genome. However, these methods are based on preprocessing and indexing of one or both sequences which is a time-consuming operation. For example, in DNA read aligner, the reference-genome is indexed once, and the same index is used each time a new read is aligned. We are looking for a quick algorithm to identify MEMs between relatively short sequences that change for each alignment. A brute force method for this problem (Additional file 1: Section II) is slow and inefficient (with the complexity of *O*(*n*^3^)). We propose a fast bit-level parallel method to speed up the MEM extraction process. Our MEM extraction method is based on the shift and compare operations shown in Fig. 3b. The first step is to represent sequences with bit-vectors, where A, C, T, and G are encoded as 00, 01, 10, and 11, respectively (Additional file 1: Section III). Figure 6 illustrates an example sequence pair, along with corresponding bit-vector representations. In a commodity computer, the machine word is usually 64 bits which can accommodate 32 nucleotide symbols. Since a sequence is usually larger than 32 symbols, the corresponding bit-vector is stored in multiple machine words. Each operation on bit-vectors of sequences of size *n* symbols acts on $\lceil \frac {n}{32} \rceil $ machine words.

With bit-vectors representation of sequences, shifting a sequence by one symbol is the same as shifting the bit-vector by two bits, and comparing sequences can be done with XOR instruction (32 symbols at a time). In the XOR output (*X*), 00 means that symbols are matched, and a sequence of 00s shows a MEM. A set of shift and bitwise operations as shown in Algorithm 1 computes *X* and subsequently the edge bit-vector (*E*) in which the start and the end of each MEM are highlighted with set bits (bits with a value of one). Figure 6 shows the *X* and the *E* bit-vectors with highlighted MEMs. The positioning of MEMs in sequences are then computed from the edge bit-vector (Additional file 1: Section IV).


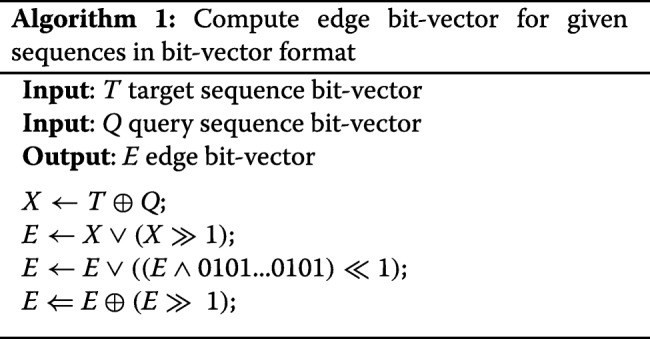


### Alignment algorithm

In “Approach” section, we show that by considering different combinations of MEMs and computing the alignment score for the corresponding alignment, one can identify the combination of MEMs that results in the maximum alignment score. However, examining all possible combination of MEMs is a naive solution. A more systematic way of finding the alignment efficiently is to use dynamic programming.

Dynamic programming is the method of approaching the solution to a problem by defining and solving smaller subproblems. Solutions for subproblems are used to solve a bigger problem at each step. The process is repeated until all subproblems are solved. Eventually, the solution to one of the subproblems would be the solution to the initial problem. When all subproblems are solved a backtracking process identifies a series of solutions that contribute to the final solution. In dynamic programming, there should be an ordering of the input data along which the recursion procedure proceeds.

We sort all MEMs according to the position of their end in query sequence (*EQ*). MEMs which end in the same position are ordered in an arbitrary way. The *j*^*th*^ subproblem is to find the alignment of subsequences of *T* and *Q* which end at *j*^*th*^ MEM *M*_*j*_ (*T*[1...*ET*_*j*_] and *Q*[1...*EQ*_*j*_] respectively). We will show that this ordering of MEM is sufficient to support the correct recursion.

In the sorted list of MEMs, *EQ*_*i*_=*EQ*_*j*_ indicates that one of *M*_*i*_ or *M*_*j*_ fully overlaps the other MEM in the query sequence. Since in *Φ*_2*B*_ of Algorithm 2 the overlap region is excluded, *M*_*i*_ and *M*_*j*_ cannot be in the same alignment. Thus *i*^*th*^ and *j*^*th*^ subproblems are solved independently from each other and the order of *i* and *j* in the sorted list could be arbitrary. If *EQ*_*k*_>*EQ*_*j*_ (*k*>*j* in the sorted list), *M*_*k*_ could not be a part of the alignment that ends in *M*_*j*_. Thus the *j*^*th*^ subproblems can be solved independently from the solution to the *k*^*th*^ subproblem. Note that it is also possible to sort MEMs based on their ending position in the target sequence (*ET*) using a similar justification.

**Fig. 7:**
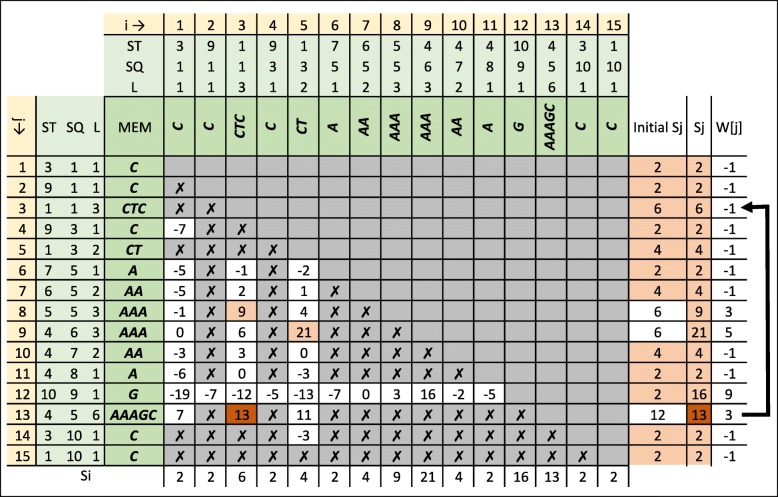
Dynamic programming table used in Algorithm 2 to process extracted MEMs in Fig. 3b. Cell *i* and *j* represent the value of $S_{i}^{j}$. Empty cells are not evaluated in *Φ*_2_. Evaluation of cells with cross mark are skipped in *Φ*_2*A*_. Initial Value of *S*_*j*_ is computed in *Φ*_1_. Final value of *S*_*j*_ and its source (what maximises *S*_*j*_) are highlighted for each row. The highest *S*_*j*_ (*S*_13_) is the alignment score. *M*_13_ is the last MEM in the alignment and the MEM before that is *M*_*W*[13]_=*M*_3_. Since *W*[3]=−1,*M*_3_ is the first MEM in the alignment. The scoring system for this alignment is *R*_*m*_=2,*P*_*x*_=3,*P*_*o*_=4 and *P*_*e*_=1

**Fig. 8:**
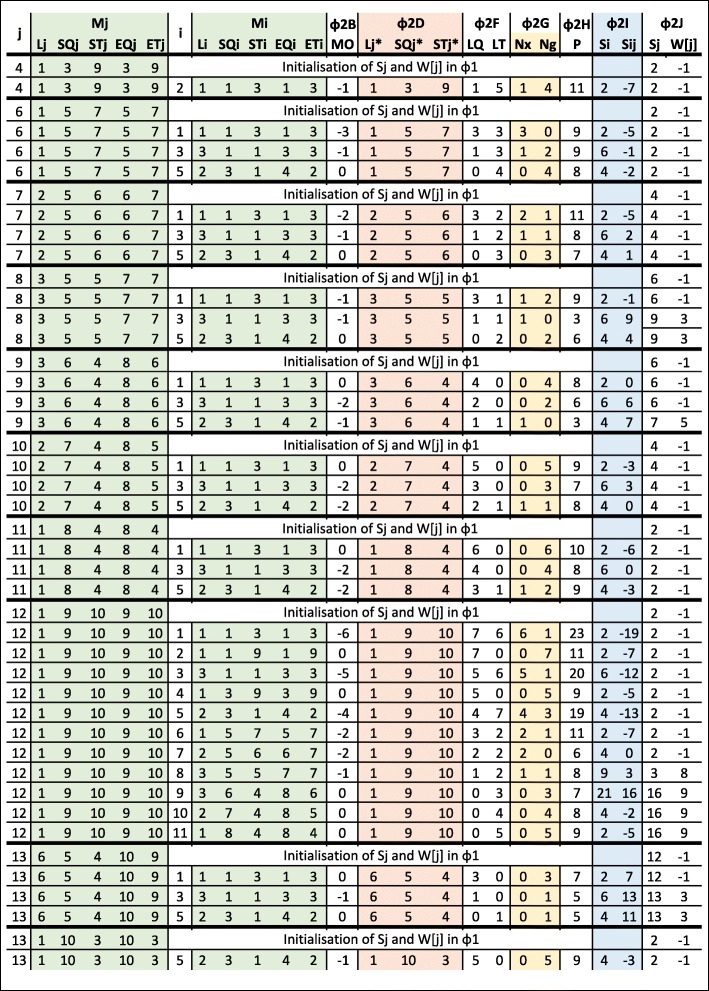
Intermediate values to compute $S_{i}^{j}$ in Fig. 7. Note that Sij in this figure refers to $S_{i}^{j}$

Our proposed dynamic programming algorithm (*DP-MEM*) is elaborated in Algorithm 2. For the example MEMs extracted in Fig. 3b, the dynamic programming table and intermediate value computed in the algorithm are shown in Figs. 7 and 8 respectively. The input to *DP-MEM* is the list of MEMs where each MEM (*M*_*j*_) is a triplet of integers [*L*_*j*_,*SQ*_*j*_,*ST*_*j*_]. The second input *n* is the number of MEMs in the list. The output *S* is the alignment score for the sequences. The algorithm prints out the indices of all MEMs that forms the alignment where the first and the last printed numbers are the indices of the rightmost and the leftmost MEMs in the alignment respectively. All steps of Algorithm 2 are commented in the following:

- *Φ*_1_: Scoring each MEM for all of its matching symbols. Note that there are *L*_*j*_ matching symbols in *M*_*j*_. *S*_*j*_ represents the highest alignment score for the alignment ending at *M*_*j*_. Initialising *S*_*j*_ in this step is similar to computing the partial alignment score when only *M*_*j*_ is included in the alignment. *W*[ *j*] is used for backtracking. The value of -1 indicates that the current *S*_*j*_ is obtained by considering *M*_*j*_ alone in the alignment.
- *Φ*_2_: Computing *S*_*j*_ for each MEM (*M*_*j*_). To compute *S*_*j*_, for each MEM *M*_*i*_ where *M*_*i*_ appears before *M*_*j*_ in the list, the algorithm adds *M*_*j*_ to the alignment ending at *M*_*i*_ (extending previously found alignments) and looks for the extension that maximises *S*_*j*_ (solving a bigger subproblem using previously solved subproblems).
- *Φ*_2*A*_: Skip extension when it is not possible. If *ET*_*i*_>*ET*_*j*_ then *M*_*i*_ contains part of target sequence which is beyond the alignment ending at *M*_*j*_ and the extension is not possible. If *EQ*_*i*_=*EQ*_*j*_ or *ET*_*i*_=*ET*_*j*_ or *SQ*_*i*_≥*SQ*_*j*_ or *ST*_*i*_≥*ST*_*j*_ then one of the MEMs fully overlaps the other MEM. In this case, *M*_*i*_ and *M*_*j*_ cannot be in an alignment together.
- *Φ*_2*B*_: Computing the length of the overlap between *M*_*i*_ and *M*_*j*_. If ${MO}_{i}^{j}$ is less than or equal to zero, then no overlap exists.
- *Φ*_2*C*_: Keeping a copy of *M*_*j*_ before excluding overlap.
- *Φ*_2*D*_: If overlap exists, excluding overlap from *M*_*j*_
- *Φ*_2*E*_: Computing ending position of *M*_*j*_ in *T* and *Q*.
- *Φ*_2*F*_: Computing the distance (number of symbols) between *M*_*i*_ and *M*_*j*_ in *T* and *Q*.
- *Φ*_2*G*_: Computing number of mismatches and gaps between *M*_*i*_ and *M*_*j*_.
- *Φ*_2*H*_: Computing the penalty for the mismatches and gaps between *M*_*i*_ and *M*_*j*_ ($P_{i}^{j}$). If gap exists, only one gap open penalty is subtracted.
- *Φ*_2*I*_: Computing alignment score $\left (S_{i}^{j}\right)$ when *M*_*j*_ is added to the alignment ending at *M*_*i*_. The score for all the matching symbols in *M*_*j*_ (*L*_*j*_×*R*_*m*_) is added to the alignment score for the alignment ending at *M*_*i*_ (*S*_*i*_). Then the penalty for the gaps and mismatches between *M*_*i*_ and *M*_*j*_$\left (P_{i}^{j}\right)$ is subtracted.
- *Φ*_2*J*_: If extending *M*_*j*_ to the alignment ending in *M*_*i*_ results into a score $\left (S_{i}^{j}\right)$ higher than current score for *M*_*j*_ (*S*_*j*_) then the new score is stored in *S*_*j*_. Also *W*[*j*] is set to *i* to keep track of the *M*_*i*_ that maximise the score for *M*_*j*_.
- *Φ*_2*K*_: Restoring the the value of *M*_*j*_ before exclusion so that *M*_*j*_ can be used in other alignment extensions.
- *Φ*_3_: Looking for the MEM with the highest *S*_*j*_. This MEM is the last MEM in the alignment (*M*_*e*_). The highest score (*S*_*e*_) is returned as *S* which is the highest alignment score for the given sequences. The index of the MEM that maximises *S*_*j*_ is stored in *e* to begin backtracking from *M*_*e*_.
- *Φ*_4_: In the alignment, the immediate previous MEM to *M*_*e*_ is the one that maximises the alignment score for *M*_*e*_. The index of such MEM is stored in *W*[ *e*]. As a result, the iteration of *f*←*W*[*f*] visits the index of all MEMs in the alignment. When *W*[*f*] is equal to -1, *M*_*f*_ is the first MEM in the alignment and the iteration is stopped.

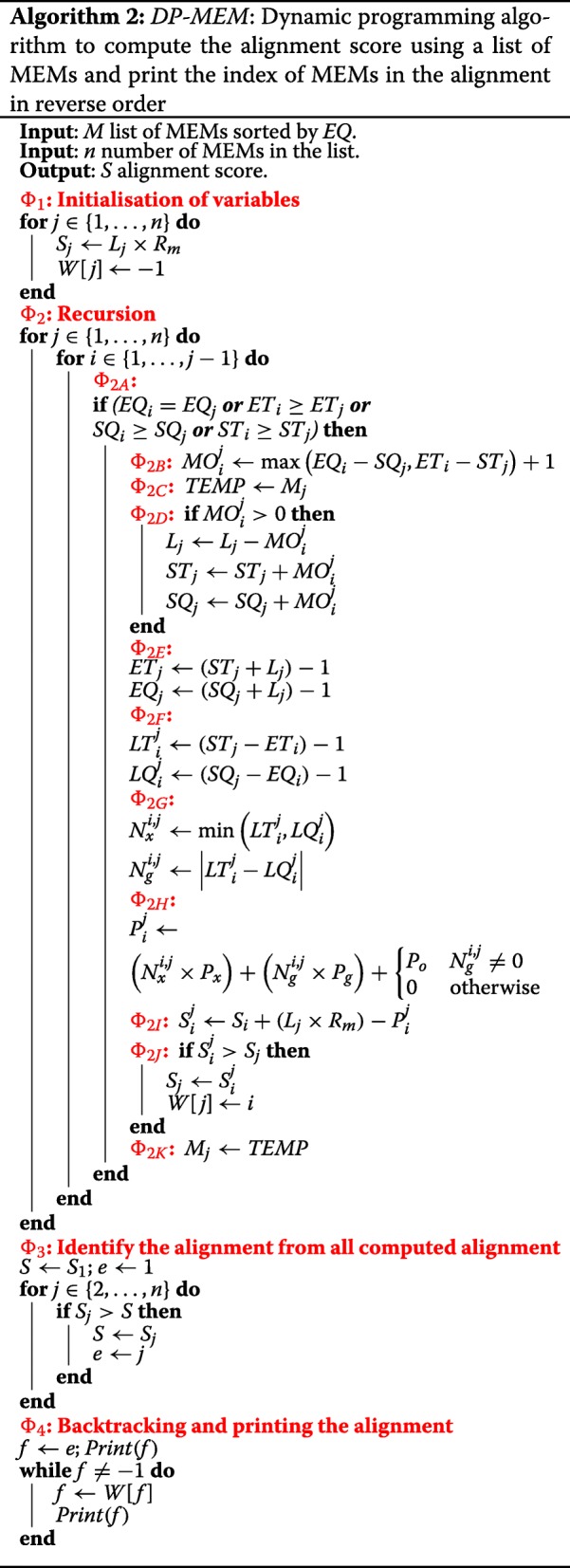


*W*[*j*] only keeps one index for *M*_*j*_. However, there might be cases that multiple MEMs maximise *S*_*j*_ (i.e. $S_{j}=S_{x}^{j}=S_{y}^{j}$). Also, there might be cases that multiple MEMs maximise the alignment score (i.e. *S*=*S*_*x*_=*S*_*y*_). Considering all these cases and backtracking through all the paths results in retrieving multiple alignments, all of which are have the same score. Reporting multiple alignment paths is not implemented in our experimental implementation, though there is no practical limitation to implementing it.

In our algorithm, we do not penalise mismatches and gaps before the first MEM and after the last MEM in the alignment. This results in a local alignment algorithm. By considering these penalties the algorithm generates a global alignment (Additional file 1: Section V).

The equation to compute $P_{i}^{j}$ in *Φ*_2*H*_ of Algorithm 2 assumes that there is no matching symbol between *T* and *Q* in the area between *M*_*i*_ and *M*_*j*_ (all symbols are counted as mismatches or gaps). Although this assumption is not true for all *M*_*i*_, it is always true for the *M*_*i*_ that leads to maximum $S_{i}^{j}$ which overrules the effect of the assumption being incorrect for other *M*_*i*_. As a proof, assume there is a matching symbol in the area between *M*_*i*_ and *M*_*j*_. The matching symbol would be a MEM (*M*_*k*_). *M*_*k*_ is already extended to the alignment ending at *M*_*i*_. Thus, when extending *M*_*j*_ to *M*_*k*_ a higher score is achieved when compared to extending *M*_*j*_ to *M*_*i*_.

Chaining colinear seeds as discussed in [33] have been widely used in the alignment of large sequences, i.e. genome-to-genome alignment. It has been also used to identify candidate regions for a read given a set of MEMs in BWA. Chaining algorithms with scoring are similar to the dynamic programming algorithm we proposed (*DP-MEM*). However, there are differences that make *DP-MEM* suitable for pairwise alignment of short sequences. *DP-MEM* takes into account that all MEMs within a certain gap size are provided in the input and optimises the number of iteration in the algorithm. *DP-MEM* also implements a heuristic approach to compensate for the effect of short MEMs removed from the input list resulting gaps between MEMs.

### Optimisation

Given sequences of length *n*, the algorithm to extract MEMs (provided in “MEM extraction” section) requires 2(*n*−1) shift and 2*n*−1 compare operations on bit-vectors (each act on $\lceil \frac {n}{32} \rceil $ machine words) that result in an algorithm with complexity of *O*(*n*^2^) to produces edge bit-vectors for the given pair of sequences. The complexity of the function that computes positioning of MEMs from the edge bit-vector and sorts them based on *EQ* is yet to be added. Further, if *m* MEMs are extracted, *Φ*_2_ of Algorithm 2 (*DP-MEM*) has the complexity of *O*(*m*^2^). Considering the complexity of MEM extraction and *DP-MEM*, *MEM-Align* is much slower than available alignment algorithms. To speed up the process, we present several optimisations for *MEM-Align* which achieves faster runtime by sacrificing accuracy. On the other hand, we introduce methods to improve accuracy with a minimal performance loss.

#### Banded alignment

Banded alignment [34] is a known heuristic method to speed up the alignment process. This technique limits the pattern of the gaps in the alignment (Additional file 1: Section VI). Consequently, if the alignment between two sequences does not follow this pattern, the algorithm will not identify the alignment. In traditional dynamic programming, the alignment is obtained after computing the value of all cells in the table. However, with the banded alignment optimisation, only cells on the diameter and close to diagonal are evaluated. *gl* is the width of the band in banded alignment where cells farther than *gl* to the diameter are not evaluated. Darker cells in Fig. 1 show the case where *gl*=1.

Unlike traditional dynamic programming approach, *MEM-Align* does not have a similar table to apply banded alignment. However, we found that we can simulate the same effect by limiting the number of shift operations in the MEM extraction process. For example, if we shift the query sequence up to *gl* to the right and to the left, we achieve banded alignment with the band of *gl*. Banded-alignment reduce the complexity of MEM extraction from *O*(*n*^2^) to *O*(*n*.(2*gl*+1)) where *gl* is a small and fixed value. Thus, the complexity of MEM extraction is *O*(*n*) when banded alignment is applied. Also, with the said banded alignment, it is likely that fewer MEMs are extracted which speeds up the subsequent algorithmic steps.

#### Short MEM removal

We observed that the majority of extracted MEMs are short and are the result of randomly matching symbols. To speed up *MEM-Align*, MEMs shorter than *sl* are filtered out during MEM extraction process. This reduces the number of MEMs to be extracted and processed (subsequently speeding up the algorithm). Filtering short MEM is done by extending Algorithm 1 with a set of shift and bitwise operations (Additional file 1: Section VII).

On the other hand, there are rare cases in which short MEMs are part of the alignment; for example, a matching symbol surrounded by mismatches. Without having all MEMs in the input list, *DP-MEM* is not able to find the same alignment as it finds when all MEMs exist in the input list. In order to compensate for lost short MEMs in the input, we modify *Φ*_2*H*_ of *DP-MEM* to consider the possibility of having short matches between MEMs (Additional file 1: Section VIII).

**Fig. 9:**
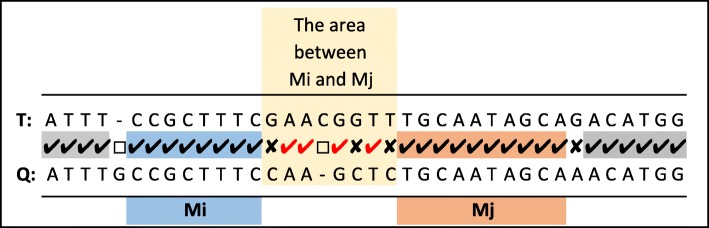
An example that shows multiple short MEM in the small area between *M*_*i*_ and *M*_*j*_ in an alignment

There might be more difficult cases where in the alignment, multiple short MEMs exist between two MEMs (see Fig. 9). The only way to correctly identify the score for the area between *M*_*i*_ and *M*_*j*_ in *Φ*_2*H*_ is to apply a global alignment to this region. However, *Φ*_2*H*_ is a frequent operation and should remain fast. Consequently, we decided to partially overcome the problem by limiting possible cases (a heuristic method).

If there are gaps in the area between *M*_*i*_ and *M*_*j*_, we assume there is only one continuous gap either to the left end or to the right end of the area. Then, only two alignments are possible for the area. The number of matching symbols is counted for both cases in a sequential manner and the one that results in maximum matches is taken as the number of matches between *M*_*i*_ and *M*_*j*_ (Additional file 1: Section IX). The sequential comparison is an expensive operation and we devise a method to avoid the sequential comparison when possible (Additional file 1: Section X).

Any other case that does not fit the above assumption results in an alignment with a lower score. However, considering the low rate of gaps and mismatches, the possibility of having multiple gaps and mismatches in a small area is low.

#### Efficient alignment extension

In *Φ*_2_ of *DP-MEM*, *M*_*j*_ extends all alignments that end in {*M*_1_…*M*_*j*−1_} (if possible). However, for each *M*_*j*_ there is a smaller subset *Ω*_*j*_⊆{*M*_1_…*M*_*j*−1_} such that by extending *M*_*j*_ to all alignments ending in a *M*_*i*_∈*Ω*_*j*_ the alignment which ends in *M*_*j*_ is found (Eq. 2). In other words, there would be fewer $S_{i}^{j}$ to be evaluated. Definition of the set *Ω*_*j*_ and the proof of Eq. 2 are provided in Additional file 1: Section XI. The definition of *Ω*_*j*_ is affected when short MEM removal optimisation is applied (Additional file 1: Section XII).

2 $$  \max\limits_{M_{i} \in \Omega_{j}}{S_{i}^{j}} = \max\limits_{1 \leq i \leq j-1}{S_{i}^{j}}  $$

#### Hybrid alignment

To maintain the accuracy of the algorithm, we decided to utilise a hybrid method that is a combination of *MEM-Align* and Smith-Waterman algorithm. We define three cases in which *MEM-Align* may be inaccurate. If the alignment of a pair of sequences falls down into one of these cases, we use the Smith-Waterman algorithm to align sequences. These cases are:

- When the sequences are repetitive, and the number of extracted MEMs exceeds the threshold *TM*. We found *MEM-Align* is likely to produce inaccurate alignment when aligning repetitive sequences. An appropriate *TM* value decreases the chance of reporting inaccurate alignment with a negligible increase in the average processing time.
- When the computed alignment score for the alignment generated by *MEM-Align* is lower than a threshold *TS*. This case mostly occurs when there is a gap in the alignment which cannot be identified due to banded alignment.
- When no MEM longer than *sl* exists to be extracted (a rare case). If *sl* is set to a high value and the similarity between sequences is low,

Although sending sequence pairs to an external algorithm results in additional computation, the number of sequences sent to the external algorithm remains small if appropriate values are chosen for *TM* and *TS*.

#### Skipping distant MEMs

When the distance between *M*_*i*_ and *M*_*j*_ is large, it is not likely to have *M*_*i*_ and *M*_*j*_ as adjacent MEMs in the alignment. Therefore, the algorithm skips the extension if the distance between *M*_*i*_ and *M*_*j*_ is longer than a threshold *TD* (further reducing the number of $S_{i}^{j}$ to be evaluated). This optimisation slightly improves the performance with a negligible side effect on accuracy.

## Results

In order to evaluate *MEM-Align*, four synthetic datasets and one realistic dataset, shown in Table 3, were considered. Each of these contains one million sequence pairs. Synthetic datasets were prepared by random selection of short sequences from the reference human genome followed by simulated variations. The realistic dataset was taken from mapped reads of sample *HG00096* downloaded from the 1000 genomes project [35]. In the realistic dataset, each mapped read is paired with a sequence of the same size taken from its mapping location in the reference-genome. All datasets are available along with the *MEM-Align* package. These multiple datasets allowed estimating the impact of sequence length and sequence divergence levels on the speed and accuracy of the various algorithms. All the tests were run on a Linux (version 3.13.0-58-generic) machine with Intel i5-3470 processors, in single thread mode.

**Table 3:** Datasets

Dataset	Sequence length	Variation rate		
		SNP	Indel	Indel expansion
DSL	125	1%	0.1%	5%
DLL	500	1%	0.1%	5%
DSH	125	5%	0.5%	10%
DLH	500	5%	0.5%	10%
DRQ	250	Natural	Natural	Natural

Two different configurations of *MEM-Align* (MA1 and MA2) as described in Table 4 were compared with four other alignment algorithms: A SIMD implementation of Smith-Waterman (SSW) [22]; an implementation of Ukkonen algorithm (UKK) taken from SNAP [12]; a Gene Myers algorithm (GM); and, a combination of Gene Myers with Hirschberg algorithm (GMH) implemented in the SeqAN package [23]. In order to implement hybrid alignment, *MEM-Align* uses the SSW algorithm internally. The appropriate values of *TM* and *TS* depend on sequence length and varies for each dataset (Table 4). The lower *TS* in MA2 (compared to MA1) leads to a lower number of sequence pairs being sent to SSW and results in a faster but less accurate alignment. On the other hand, MA1 delivers higher accuracy at the cost of higher execution time.

**Table 4:** *MEM-Align* Configurations

	Datasets				
	DSL	DSH	DLL	DLH	DRQ
TM	50	50	200	200	90
TS (MA1)	100	80	450	300	215
TS (MA2)	20	20	200	100	50

For all datasets *gl*=6,*sl*=4and *TD*=25were used

Accuracy was measured using two different metrics: the number of suboptimal alignments; and, the average alignment score difference between the reported alignment score and the optimal alignment score (reported by SSW). For the purposes of measuring accuracy, if the alignment score reported by an algorithm is less than the alignment score reported by SSW, the alignment is considered suboptimal.

Details regarding how SSW, UKK, GM and GMH were used in our experiments are available in Additional file 1: Section XIII. Execution time and accuracy are measured for existing alignment algorithms as well as for several configurations of *MEM-Align*, showing the effect of varying parameters on differing datasets. In order to get a deeper insight into the effect of the optimisations on *MEM-Align* the following metrics were also measured.

- The number of sequences sent to SSW because of *TM* and *TS*.
- The average number of extracted MEM for those sequences that are not sent to SSW.
- Number of alignment extensions (number of evaluated $S_{i}^{j}$ in Fig. 7) avoided by *Ω* and *TD* optimisation.
- Number of times sequential string comparison of the area between *M*_*i*_ and *M*_*j*_ is avoided.
- Proportion of execution time spent on each of the sub-processes including file access (IO), string to bit-vector conversion, MEM extraction, sorting, alignment, backtracking and the time spent by SSW to process sequence pairs sent to SSW.

The complete experimental report is provided in Additional file 1: Section XV. Here, we only highlight a fraction of the results that demonstrate the effects of various parameters. Note that for a streamlined application where the error rate, genome and sequence length are fixed, *MEM-Align* parameters only need to be tuned once. Thus, for the purpose of this evaluation, the *MEM-Align* parameters were tuned using datasets that were different from the ones used in the evaluation.

Our string to bit-vector conversion function (Additional file 1: Section III) is about 25 times faster than the conventional shift-and-insert loop method. Using the counting sort strategy to sort MEMs (Additional file 1: Section I) is 8.2 and 3.4 times faster than using the quick-sort algorithm when the average number of extracted MEMs for a pair of sequences is about 1330 and 42 respectively.

**Fig. 10:**
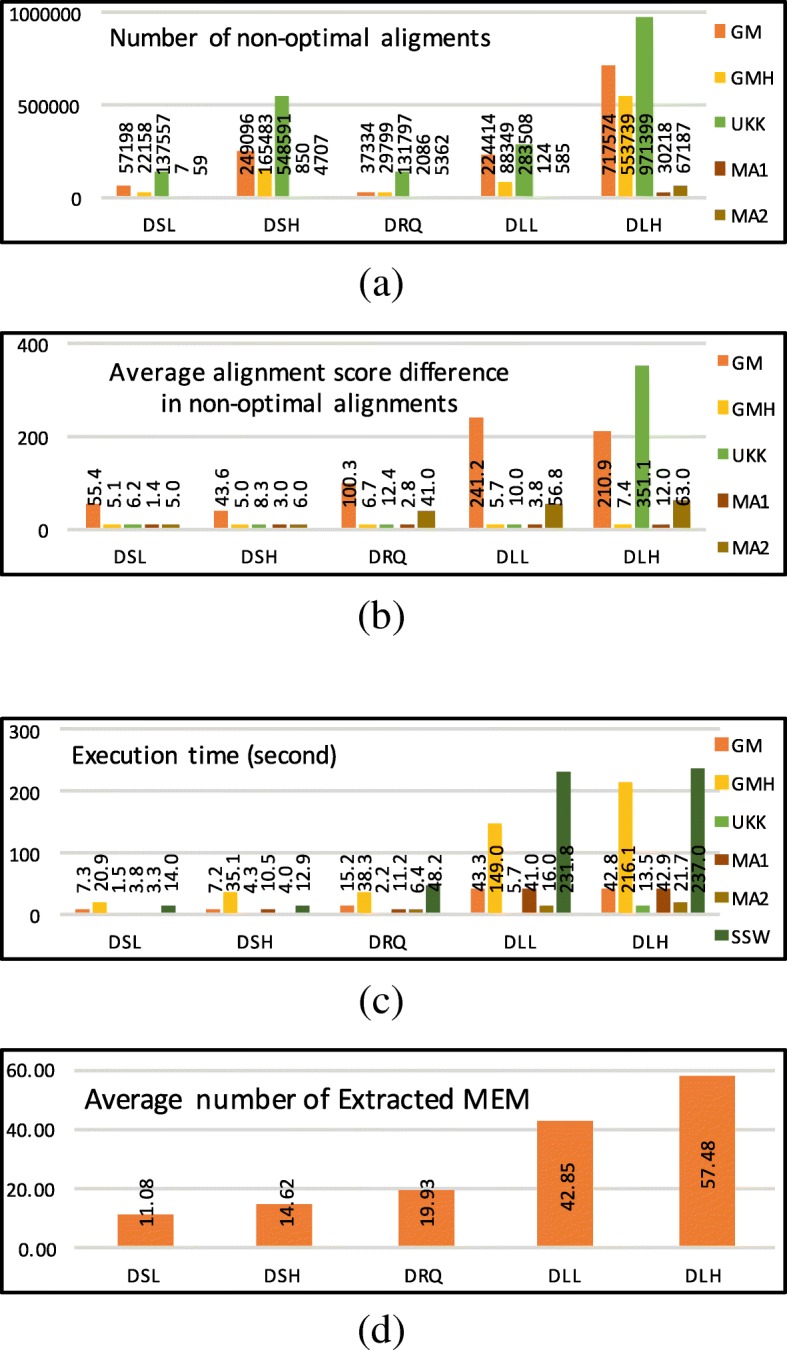
Comparing two configurations of *MEM-Align* (MA1 and MA2) with Gene Myers (GM) and Gene Myers and Hirschberg (GMH) as well as Ukkonen (UKK) and Smith-Waterman (SSW) algorithms. **a** Number of suboptimal alignments. **b** Average alignment score difference in suboptimal alignments. **c** Execution time in seconds. **d** Average number of extracted MEMs for a pair of sequences (MA2)

Figure 10a, b and c show the number of suboptimal alignments, average alignment score difference, and execution time respectively. While the execution time of SSW is quadratic in the length of the sequence, the UKK execution time seems to be linear in sequence length. The *MEM-Align* execution time is a more complex function and depends on other factors such as error rate and given parameters. Although UKK is the fastest algorithm, *MEM-Align* results in a considerably lower number of suboptimal alignments and is only slightly slower than UKK. Figure 10a suggests that the alignment produced by edit-distance based methods such as GM and UKK differs from the alignment produced by SSW (gold standard) for a large number of input sequences. However, *MEM-Align* can produce alignment identical to SSW in most cases. Thus MEM-Align is a better alternative for SSW in read-mapping applications.

Figure 10d represents the average number of extracted MEMs for a pair of sequences (*Θ*) for MA2 configuration of *MEM-Align*. *Θ* is in direct relation with the time spent on execution time. *Θ* is a helpful guideline for identifying the optimal value of *TM*. Although the exact function has not been identified yet, it appears that 4*Θ*<*TM*<5*Θ* is a suitable estimation.

**Fig. 11:**
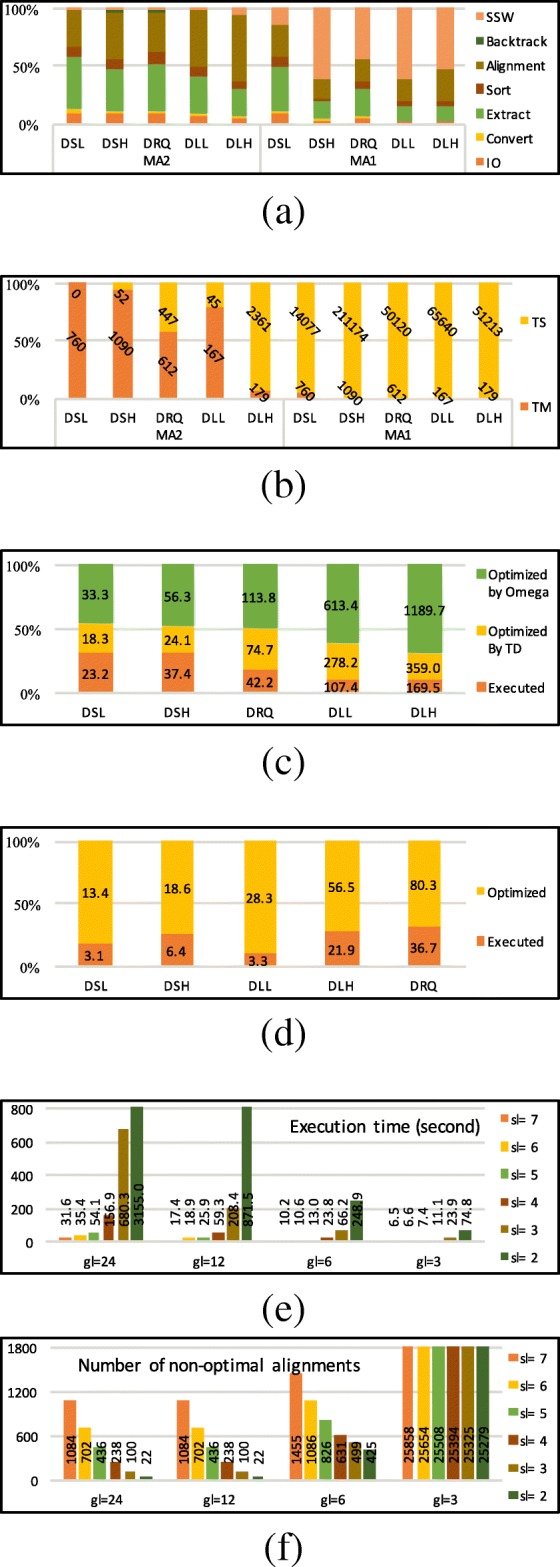
The effect of proposed optimisations on *MEM-Align*. **a** Proportion of execution time for different algorithmic steps. **b** Number of sequence pairs sent to SSW by TM and TS. **c** Number of alignment extensions in million for MA2 (normalised bar). **d** Number of string comparisons per million for MA2 (normalised bar). **e** Execution times varying *gl* and *sl*. **f** Number of suboptimal alignments varying *gl* and *sl*

The proportion of execution time for each algorithmic step of *MEM-Align* is shown in Fig. 11a. Since the execution times for some configuration of *MEM-Align* are negligible compared to other configurations, in Fig. 11a all execution times are scaled to a 100% bar to allow for better visualisation. MA1 spends more time in SSW since more sequences are sent to SSW because of higher value for *TS*.

The number of sequence pairs sent to SSW because of *TM* and *TS* are shown in Fig. 11b. Data in Fig. 11b are scaled to 100% bar in order to reflect the percentage of sequence pairs sent to SSW yet the labels show the actual value.

Figure 11c reports the expected number of times the alignment extension is executed along with the number of times alignment extension is avoided (optimised) because of using the set *Ω* and the parameter *TD*. Both of these optimisations have been noticeably effective. Figure 11d demonstrates the number of times sequential string comparison of the area between *M*_*i*_ and *M*_*j*_ is avoided. All bars are scaled to 100%, yet labels show the actual number in millions.

The effect of varying *sl* and *gl* (on DLL dataset) are shown in Fig. 11e (execution time) and Fig. 11f (the number of suboptimal alignments). While *sl*<4 exponentially increases execution time, *sl*>4 result in significant increase in number of suboptimal alignments. *sl*=4 seem to be the most appropriate value. *gl*=6 delivers the best trade off between speed and accuracy for this data set. In these figures *TM*=1,000,000, *TD*=1,000 and *TS*=0 are set to disable corresponding optimisation and only show the effect of *sl* and *gl*. To see the effect of *TM*, *TS* and *TD* refer to Additional file 1: Section XV.

## Discussion

Due to the similarity in many operations, there is potential to internally implement the SHD filter [36] in the MEM extraction step with minimal additional computation. In [37], SHD is accelerated using custom hardware resulting in up to 17.3 times speed up. Similar Hardware acceleration can be applied to our MEM extraction process.

Finally, we are aware that Gene Myers and Ukkonen algorithms are edit-distance based alignments and do not support affine-gap scoring. The reason we compare them to *MEM-Align* is that they are used in recent DNA read-mappers as an alternative to the Smith-Waterman algorithm. Our results demonstrate that *MEM-Align* is likely to be a better substitute for the Smith-Waterman algorithm as it allows for affine-gap scoring.

The implementation of *MEM-Align* contains a module that prints out human-readable colourful alignments. For each alignment, this module highlights extracted MEMs in the alignment as well as removed short MEMs which are part of the alignment and are identified by the sequential string compare operation. A sample output is provided in Additional file 1: Section XIV.

## Conclusions

Pairwise alignment is one of the most frequently utilized operations in sequence analysis. Considering the growth in the amount of sequence data to be processed in the near future [38], even a small improvement in the alignment operation can save significant computational power. *MEM-Align* delivers considerable speed improvement, especially in the case of longer sequences where the traditional alignment methods slow down quickly.

Decision making based on sequenced data is life critical. However, errors are common in the sequencing process. Most analysis overcome errors by utilising redundant data (overlap sequences). We believe the negligible number of suboptimal alignments produced by *MEM-Align* can be partly compensated by the existing redundancy in the data. When speed matters, *MEM-Align* is the best algorithm as it is fast and its output is near identical to the Smith-Waterman algorithm with affine-gap scoring. Other alternative such as UKK and GM are also fast but only support edit-distance based scoring.

## Additional file

Additional file 1 Supplementary Data. Supporting material as well as the complete experimental result are provided in supplementary data. (PDF 3044 kb)

## Abbreviations

DLH: Dataset of long sequences with high error rate
DLL: Dataset of long sequences with low error rate
DRQ: Dataset of real query sequences
DSH: Dataset of short sequences with high error rate
DSL: Dataset of short sequences with low error rate
MEM: Maximal exact matches
SHD: Shifted hamming distance
SIMD: Single instruction multiple data
SSE: Streaming SIMD extensions
