# Supplementary material for: Pairwise alignment of nucleotide sequences using maximal exact matches
Source: BMC Bioinformatics. 2019 May 21;20:261. doi: 10.1186/s12859-019-2827-0 (PMC6528274; doi:10.1186/s12859-019-2827-0)
Supplement: Supplementary file 1 — Supplementary Data. Supporting material as well as the complete experimental result are provided in supplementary data. (PDF 3044 kb) [file 12859_2019_2827_MOESM1_ESM.pdf]

Pairwise alignment of nucleotide sequences using maximal exact matches **(Supplementary Data)**

Arash Bayat, Bruno Gaëta, Aleksandar Ignjatovic and Sri Parameswaran

March 14, 2019

I Sorting

There is a wide range of sorting algorithm for various applications. In case of *MEM-Align* where extracted MEMs must be sorted in order of EQ, the counting sort is an efficient option. Counting sort is a linear time sorting algorithm with complexity of $O(3n) = O(n)$ that is applicable only when the sorted values vary in a small range. Since the EQ varies between 1 and the sequence length which is a relatively small value, counting sort strategy is well suited to this problem.

Counting sort consist of three steps: the number of MEMs ending at each position is computed in array A_{cnt} (Δ_1); the cumulative number of MEMs ending before each position is computed in array A_{cum} (Δ_2); for each MEM its index in the sorted list (SLI) is identified and the MEM is copied to its place in the sorted list SL (Δ_3). Algorithm 1 clarifies the sorting process.

```
Input: {M_1 ... M_n} List of MEMs
Output: SL Sorted List of MEMs
// Δ1: Compute A_cnt
A_cnt ← [0, 0, 0, ..., 0];
for i ∈ {1, ..., n} do
    A_cnt[EQ_i] ← A_cnt[EQ_i] + 1;
end
// Δ2: Compute A_cum
A_cum[1] ← 0;
for i ∈ {2, ..., n} do
    A_cum[i] ← A_cum[i − 1] + A_cnt[i − 1];
end
// Δ3: Sort MEMs
A_tmp ← [0, 0, 0, ..., 0];
for i ∈ {1, ..., n} do
    SLI_i ← A_cum[EQ_i] + A_tmp[EQ_i];
    SL[SLI_i] ← M_i;
    A_tmp[EQ_i] ← A_tmp[EQ_i] + 1;
end
```

Algorithm 1: Sort MEM by EQ

II Brute force MEM extraction

Algorithm 2 represents a brute force method to extract all possible MEMs from a pair of sequences. In Algorithm 2, we consider each position in Q against each position in T to see if an exact match begins from that point. If $Q[i] \neq T[j]$ then no match is started; otherwise if $Q[i-1] = T[j-1]$ then the exact match is started from the previous position and is already extracted. If $Q[i] = T[j]$ and the previous symbols are not matched then it is the start of a maximal exact match. Thus we extend the exact match up to the end of T or Q, whatever comes first. The γ symbol at the end and start of T is to guarantee that a mismatch always appears before and after each MEM. This brute force solution to extract MEMs comes with complexity of $O(n^3)$.

```
Input: T, Q sequences of length n
Output: List a list of MEMs
// Seal T with γ which is not a sequence
   alphabet
T[0] ← γ;
T[n + 1] ← γ;
for i ∈ {1, ..., n} do
    for j ∈ {1, ..., n + 1} do
        if Q[i] ≠ T[j] ∨ Q[i − 1] = T[j − 1] then
            continue
        end
        k ← i + 1;
        l ← j + 1;
        while k ≤ n ∧ l ≤ n + 1 do
            if Q[k] ≠ T[l] then
                M.SQ ← i;
                M.ST ← j;
                M.L ← k − i;
                Insert(List, M);
                break;
            end
            else
                k ← k + 1;
                l ← l + 1;
            end
        end
    end
end
```

Algorithm 2: Brute force MEM extraction with complexity $O(n^3)$

III DNA string to bit-vector conversion

Given a pair of sequences, the first processing step of *MEM-Align* is to transform sequences into bit-vectors. A

bit-vector is stored as array of longest machine word, i.e. an array of 64-bit words each of which stores up to 32 symbols (assuming 2-bit per symbol for nucleotide sequences). For this transformation, a regular method is to shift and insert symbols into the data-word one by one. However, we propose a fast bit-vector method to convert nucleotide sequences into bit-vectors.

The third and second rightmost bits of the ASCII code for A, C, T, and G form four different combinations: 00, 01, 10, and 11 respectively. These values remain the same even if lower case letters are used. As a result, considering a 64-bit machine word W as an array of eight ASCII nucleotide symbols (each 8-bit long), the procedure represented in Algorithm 3 illustrates a method to transform all eight symbols into a bit-vector (16-bit long) located in the leftmost part of W. Finally, every four consecutive words are merged together to compress all 32 symbols into one 64-bit machine word.

Note that when copying an ASCII string into an array of 64-bit words, a little-endian machine such as Intel copies the left most symbol into the least significant byte of the first word in the array. As a consequence, Algorithm 3 reverses the sequence to store the leftmost symbol into the leftmost 2 bits of the output bit-vector.

Input: IA eight ASCII code
Output: W 2-bit encoded of IA

$W \leftarrow IA \wedge 0606060606060606(hex);$
$W \leftarrow W \gg 1;$
$W \leftarrow W \vee (W \ll 10) \vee (W \ll 20) \vee (W \ll 30);$
$W \leftarrow W \wedge FF000000FF000000(hex);$
$W \leftarrow (W \gg 8) \vee (W \ll 32);$

Algorithm 3: ASCII to bit-vector

IV Edge bit-vector to triple number representation of MEMs

The last step in the extraction process is to identify the start and the length of the MEMs in the edge bit-vector produced in Algorithm 1 of the paper. In the edge bit-vector the start and the end of each MEM is marked with a set bit. there is an even number of set bits in the bit-vector. Let SB_i be the position of the ith set bit in bit-vector. The ith MEM in the bit-vector M_i is marked by SB_{2i-1} and SB_{2i}. Parameters that describe M_i are then computed using Equation 1 where sh is the number of times T is shifted to the left by one prior to extraction (negative sh is considered for right shifts)

$$OFS_i = sh \tag{1a}$$
$$L_i = \frac{SB_{2i} - SB_{2i-1}}{2} \tag{1b}$$
$$SQ_i = \frac{SB_{2i-1}}{2} + 1 \tag{1c}$$
$$ST_i = SQ_i + OFS_i \tag{1d}$$

In order to compute SB for all set bits, one strategy is to shift out bits (two bits at a time) and check if the left most bit is set. On average there should be few MEMs in a bit-vector which means there are few set bits. As a consequence, shift-and-check loop is an inefficient solution. Since most modern processors have instructions to count the number of leading or trailing zeroes in a machine word, this operation is speeded up using these instructions. the Bit Scan Forward (BFS) and Bit Scan Reverse (BSR) instructions of Intel processors as well as the Leading Zero Count (LZCNT) instruction of ARM processors are just two examples.

V Global Alignment

Algorithm2 of the paper (*DP-MEM*) describes a method to compute semi-global alignment between sequences. Here we explain how *DP-MEM* could be modified to produce a global alignment where all symbols in Q must be included in the alignment. This does not necessarily mean that the first and the last exact matches of the alignment must start and end from the start of Q and to the end of Q. In fact, there are global alignments that consider leading and trailing symbols as mismatches or insertions (whatever leads to lower penalty). As a result, global alignment should allow the alignment to start and end with any MEMs similar to the proposed semi-global alignment. To apply these changes when computing initial value of S_j in Φ_1 of *DP-MEM*, we should also consider the penalty for all symbols which comes before M_j in Q. This penalty is computed as GLP_j in Equation 2a. Then the initial value of S_j is computed using Equation 2b. Also, in Φ_3 of *DP-MEM*, the algorithm looks for the MEM with highest alignment score to be chosen as the last MEM in the alignment. This should be changed for the global alignment as we should also consider the penalty for mismatches or insertions that comes after the last MEM in Q. This penalty is computed in Equation 2c where Len refers to the length of query sequence. Then the correct value of S is computed in Equation 2d where n is total number of MEMs.

$$GLP_j = \min \begin{cases} ((SQ_j) - 1) \times P_g + P_o \\ ((SQ_j) - 1) \times P_x \end{cases} \tag{2a}$$
$$S_j = (L_i \times R_m) - GLP_i \tag{2b}$$
$$GTP_j = \min \begin{cases} (Len - EQ_i) \times P_g + P_o \\ (Len - EQ_i) \times P_x \end{cases} \tag{2c}$$
$$S = \max_{1 \le j \le n} (S_j - GTP_j) \tag{2d}$$

VI Banded Alignment

I The effect of banded alignment

Gap limited optimisation does not necessarily limit the length of the gap to gl. To understand how this optimisation limits the output alignment, INS_i and DEL_i are defined as the total length of insertions and deletions from the start of the alignment up to the ith symbol in the alignment. Then G_i is defined as $INS_i - DEL_i$. When gap limited alignment is applied, in the output alignment the value of G_i is always bounded by $-gl$ and gl.

If the alignment does not satisfy the condition above, it cannot be found using gap limited optimisation. However, in real datasets, lengthy gaps are rare and choosing a proper value for gl should result in a negligible probability of an alignment being missed.

II Alternative methods to handle large gaps

In banded alignment a smaller value for gl results in the higher processing speed with the cost of missing alignment that contains large gaps. Note that large structural variations such as copy number variations are not expected to be identified in read mapping process. Hear, large gap refer to a gap shorter than the length of a read but longer than what can be identified when banded alignment is applied. Although these large gaps are missing by banded alignment, we believe there are other option to identify most of large gaps prior to alignment.

DNA read mappers search for exact matches of subsequences of the read in the reference genome to find candidate regions in the reference genome that the read could possibly map to them. Read mappers usually take several subsequences and search them all to eliminate false positive candidate locations. Assuming that one subsequence is taken form the beginning of the read and the other taken from the end of the read. If both of these subsequences are found in the same candidate region but the distance between them in the read and in the candidate regions differs a lot then it is probable that a large gap exist in the alignment and normal alignment procedure (Smith-Waterman) should be followed. With no doubt, it is possible to bring a case in which a large insertion and a large deletion take place in the middle of a read and the above method could not identify the situation (there might be more cases). One should consider how rare such examples can be in reality. Furthermore, recent variant caller such as GATK Haplotypecaller [30] and Platypus [31] apply a local *de-novo* assembly and realignment process prior to variant identification in which missing gaps can possibly be identified.

The above argument is not to proof that we could always avoid what is missed by banded alignment, but it supports the fact that there are other strategies to identify large gaps and reduce pressure on time-consuming alignment process.

VII Masking short MEMs from the edge bit-vector

In order to mask short MEMs during the MEM extraction process described in Section 0.2 of the paper, ϕ_1 and ϕ_2 are inserted to Algorithm 1 of the paper. The modified algorithm is shown in Algorithm 4. The new algorithm replaces MEMs shorter than sl with mismatches. As a consequence, MEMs shorter than sl are not extracted by the method explained in Section IV. The short MEM removal procedure consists of two sub-processes: ϕ_1 and ϕ_2. In ϕ_1 a bit-vector F is formed in which each match (00) indicates that the next $sl-1$ symbols on the right were matched. In ϕ_2 each match in the F bit-vector is extended to the right by $sl-1$ symbols to form the modified E bit-vector in which MEMs shorter than sl are masked.

Although masking short MEM comes with additional processing, its overall effect on execution time is positive because there would be less MEMs in each bit-vector to list and also less MEMs in total to be processed subsequently.

Input: E edge bit-vector
Input: sl short MEM length
Output: E edge bit-vector, masked short MEM

$X \leftarrow T \oplus Q$;
$E \leftarrow X \vee (X \gg 1)$;
$E \leftarrow E \vee ((E \wedge 0101...0101) \ll 1)$;
// ϕ_1: `Forming F`
$F \leftarrow E$;
for $i \in \{1, \ldots, sl-1\}$ **do**
  $F \leftarrow F \vee (E \ll (2 \times i))$;
end
// ϕ_2: `Forming modified E`
$E \leftarrow F$;
for $i \in \{1, \ldots, sl-1\}$ **do**
  $E \leftarrow E \wedge (F \gg (2 \times i))$;
end
$E \Leftarrow E \oplus (E \gg 1)$;

Algorithm 4: MEM extraction with short MEM removal

VIII Short MEM Removal

There are situations where short MEMs can appear in the alignment. If these short MEMs are removed by optimisation in Section 0.4 of the paper, the Algorithm 2 of the paper (*DP-MEM*) can no longer identify the alignment. We present modifications to the *DP-MEM* algorithm to deal with some of these situations.

The simplest case where a short MEM occurs in an alignment is when two edits are close enough to each other to form a MEM shorter than sl. In a more complex case, concentration of more than two edits in a narrow region (REG in Figure 1) results in consecutive short MEMs in the alignment resulting in all of them being eliminated. Figure 1 represents four general cases where short MEMs appear in the alignment. In all cases, M_i and M_j as well as grey (removed) short MEMs are part of the alignment. In case 1, only mismatches exist, while in case 2, one gap (here gap refers to a continuous gap of any length) on one side of the REG also exists. In case 3, there is one gap but in the middle of REG. Case 4 represents the situation in which two or more gaps exist in the REG.

Since $N_x^{i,j}$ and $N_g^{i,j}$ can no longer be computed correctly in Φ_{2G} of *DP-MEM* respectively, thus computing P_i^j in Φ_{2H} of *DP-MEM* is not possible. Considering M_i and M_j consecutive long MEMs in an alignment, the distance between them could not be assumed as all mismatches and gaps as there could have been short MEMs between them that were removed. In fact, depending on the value of sl the overall score for region REG could be positive but end up being represented with a negative P_i^j.

In order to deal with all of the above cases, P_i^j should be computed using a global alignment that forces all symbols

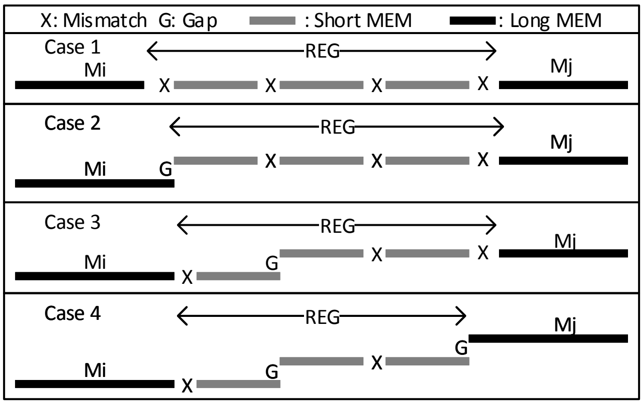

Figure 1: Generalized cases where two or more edits result in short MEMs in an alignment. There could be multiple consecutive short MEMs that result in elimination of region *REG*. Gaps and Mismatches are represented using G and X respectively.

of Q in REG to be aligned to the corresponding region in T. However, this solution is time-consuming as it should be executed each time P_i^j is computed. As a result, we propose a faster method in Section IX to retrieve the correct P_i^j. This method only supports the first two cases of Figure 1.

Note that the rate of gaps in the alignment is much smaller than the rate of substitutions; thus the probability of having two gaps near each other is extremely small. Also, as the gap open penalty is usually high, the alignment tends to put gaps together, rather than leaving multiple separate gaps in a narrow region. Case 3 in Figure 1 has a similar probability as case 2 but we neglect it for the sake of performance of our proposed algorithm. Experimental results of the paper shows that our proposed method delivers acceptable accuracy on a realistic dataset.

IX Sequential string compare

Since in the first two cases of Figure 1 at most one gap is assumed, $N_g^{i,j}$ can be computed correctly as in Φ_{2G} of *DP-MEM*. To retrieve the number of mismatches $N_x^{i,j}$ and matches $N_m^{i,j}$ between M_i and M_j, our proposed method is to look back into subsequences of T and Q which are bounded by M_i and M_j (TS_i^j and QS_i^j respectively). If there is no gap TS_i^j and QS_i^j are of the same size ($LT_i^j = LQ_i^j$). In this case, there is only one way to align symbols and $N_x^{i,j}$ and $N_m^{i,j}$ can be counted by comparing symbols in TS_i^j and TQ_i^j one by one sequentially. If there is a gap, assuming that the gap is attached to either M_i or M_j, there are only two alignments, that is, aligning TS_i^j and TQ_i^j to the left and right of each other. Then $N_x^{i,j}$ and $N_m^{i,j}$ are counted in the overlapping region of TS_i^j and TQ_i^j. Finally, The alignment that results in the lower $N_x^{i,j}$ is chosen.

The computation of P_i^j in Φ_{2G} of *DP-MEM* is also affected as there are also matches between M_i and M_j. Equation 3 computes the correct value for P_i^j.

$$P_i^j = (N_x^{i,j} \times P_x) + (N_g^{i,j} \times P_g) + \begin{cases} 1 & N_g^{i,j} \neq 0 \\ 0 & \text{otherwise} \end{cases} - (N_m^{i,j} \times R_m) \tag{3}$$

Short MEMs might appear before or after the first and the last MEM of the alignment. This issue is treated similarly to short MEMs between two long MEMs as discussed above. After the alignment has been found, the subsequences of T and Q that appears on the left of the first MEM of the alignment M_{first} are aligned to the right of each other. Then, the numbers of matches and mismatches are counted from right to left for the whole overlapping region. The match score and mismatch penalty are progressively added to and subtracted from S_{first} to see at which point it has been maximised. Finally, the beginning of M_{first} in T and Q is extended left-ward to the point that maximum S_{first} is achieved. A similar procedure is applied to the last MEM of the alignment in the reverse direction.

X Efficient Sequential Compare

Comparing TS_i^j and TQ_i^j in a sequential manner for all computed P_i^j is yet another time-consuming process; thus we propose an optimisation to avoid computing P_i^j in some cases. In the proposed optimisation we compute minimum possible P_i^j and check if the resulting S_i^j is higher than the current maximum computed S_j. If this condition is met, the actual P_i^j is computed and S_i^j is compared to the current maximum S_j.

In order to estimate the minimum possible P_i^j, we assume REG is composed mainly of groups of contiguous $sl - 1$ matches which are separated by individual mismatches. On both sides of REG there should be a gap or a mismatch to separate it from the rest of the alignment. In each sl group of symbol there should be at least one mismatch. Based on the number of remaining symbols (computed using equation 4a and the existence of the gap on one side of REG one or two additional mismatches might be added to the end. The maximum possible $N_m^{i,j}$ ($maxN_m^{i,j}$) and subsequently minimum possible $N_x^{i,j}$ ($minN_x^{i,j}$) in REG are then computed using Equation 4b and 4c respectively, based on the length of the REG which is given by $L_{reg} = min(LT_i^j, LQ_i^j)$ and the parameter sl.

$$MOD = L_{reg} \mod sl \tag{4a}$$

$$minN_x^{i,j} = \left\lfloor \frac{L_{reg}}{sl} \right\rfloor + \begin{cases} 1 & MOD = 0 \wedge N_g^{i,j} = 0 \\ 1 & MOD = 1 \wedge N_g^{i,j} = 0 \\ 2 & MOD \geq 1 \wedge N_g^{i,j} = 0 \\ 0 & MOD = 0 \wedge N_g^{i,j} \geq 0 \\ 1 & MOD = 1 \wedge N_g^{i,j} \geq 0 \\ 1 & MOD \geq 1 \wedge N_g^{i,j} \geq 0 \end{cases} \tag{4b}$$

$$maxN_m^{i,j} = L_{reg} - minN_x^{i,j}; \tag{4c}$$

XI The set Ω: Definition and Proof

To better understand how Ω_j is defined, a diagram in Figure 2 represents a set of MEMs as lines where MEMs in a row have the same offset OFS (see Equation 1). The placement of lines in each row represents the placement of the related MEMs in query sequence (MEMs with the same offset cannot overlap and are disconnected). In Figure 2, black lines represent M_i where $i < j$ and bold lines represent Ω_j. Those MEMs that are not involved in the computation of S_j are drawn in grey. Ω_j has at most one member in each row. $M_{\omega(j,ofs)}$ is a member of Ω_j if it is the closest MEM to M_j amongst other MEMs in the same row (with the same OFS). Also, $M_{\omega(j,ofs)}$ should not be fully overlapped by M_j.

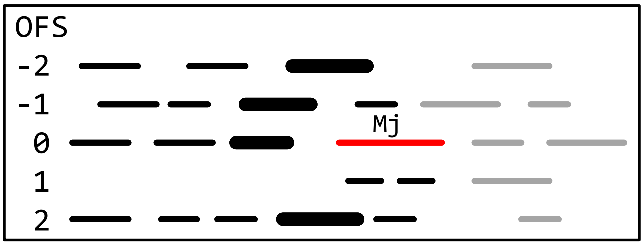

Figure 2: Representation of MEMs with lines. Each row contains MEMs with the same offset in the same order they appear in query sequence. Black lines represent M_i where $i < j$. Set Ω_j is shown with bold black line.

In order to prove Equation 2 of the paper which is the basis of the efficient alignment extension optimisation introduced in Section 0.4 of the paper, the following sets are defined first:

- H^j_{ofs}: a set of MEMs such that for $M_i \in H^j_{ofs}$, $OFS_i = ofs$ and $EQ_i < SQ_{\omega(j,ofs)}$. In other words, a set of MEMs in the same row that appears before the bold line in the row ($M_{\omega(j,ofs)}$). For example, H^j_{-1} is highlighted with green colour in Figure 3.

- H^j_*: a set of MEMs such that for $M_i \in H^j_*$, $i < j$ and $SQ_i > SQ_j$. In other words, all black line in all rows that appears after a black line. H^j_* is highlighted with blue colour in Figure 3.

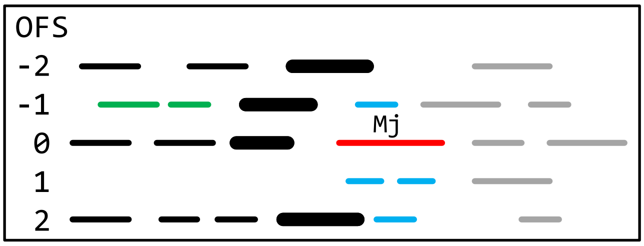

Figure 3: H^j_{-1} is highlighted with green colour. H^j_* is highlighted with blue colour.

Note that regardless of ofs, there is no common element between Ω_j, H^j_{ofs} and H^j_* whereas their union is equal to $\{M_i : i < j\}$.

The next step is to prove Inequality 5a and Inequality 5b.

Inequality 5a states that for all $M_i \in H^j_{ofs}$ there is a member of Ω_j, $M_{\omega(j,ofs)}$, such that $S^j_{\omega(j,ofs)}$ is larger or equal to S^j_i; thus, it is possible to avoid computation of S^j_i if M_i is a member of H^j_{ofs}.

Inequality 5b states that for all $M_i \in H^j_*$ there exists M_k such that $k < j$ and S^j_k is larger or equal to S^j_i; thus, it is possible to avoid computation of S^j_i if M_i is a member of H^j_*.

As a result, to prove Equation 2 of the paper it is sufficient to prove Inequality 5a and Inequality 5b. In the proof we assume computation of S^j_i is not skipped by the condition in Φ_{2A} of *DP-MEM*.

$$\forall M_i \in H^j_{ofs}(S^j_{\omega(j,ofs)} \geq S^j_i) \tag{5a}$$

$$\forall M_i \in H^j_* \exists M_k(S^j_k \geq S^j_i) \tag{5b}$$

For clarity let ω be equal to $\omega(j, ofs)$. Also let L^k_j be equal to length of M_j when its overlap with M_k (if exist) is excluded. By definition of S^j_i in Φ_{2I} of *DP-MEM*, Equation 6a and 6b hold; thus proving Inequality 5a reduces to proving Inequality 6c. By definition of S_j in Φ_3 of *DP-MEM*, S_ω is larger or equal to S^ω_i. Thus, it suffices to prove Inequality 6d. Using Equation 6e, Inequality 6d reduces to Inequality 6f.

$$S^j_\omega = S_\omega + (L^\omega_j \times R_m) - P^j_\omega \tag{6a}$$

$$S^j_i = S_i + (L^i_j \times R_m) - P^j_i \tag{6b}$$

$$S_\omega + (L^\omega_j \times R_m) - P^j_\omega \geq S_i + (L^i_j \times R_m) - P^j_i \tag{6c}$$

$$S^\omega_i + (L^\omega_j \times R_m) - P^j_\omega \geq S_i + (L^i_j \times R_m) - P^j_i \tag{6d}$$

$$S^\omega_i = S_i + (L^i_\omega \times R_m) - P^\omega_i \tag{6e}$$

$$(L^i_\omega + L^\omega_j - L^i_j) \times R_m \geq P^\omega_i + P^j_\omega - P^j_i \tag{6f}$$

Figure 4 show all possible cases for M_i, M_ω and M_j. Note that by definition $OFS_i = OFS_\omega$ thus M_i and M_ω should be on the same row and without overlap. In case 1 $OFS_j = OFS_\omega$ that force all three MEMs in the same row and without overlap. In case 2 and 3 $OFS_j \neq OFS_\omega$ thus M_ω could have overlap with M_j (case 2) but it cannot be fully overlap based on definition. Figure 4 also represent parameter required to evaluate Inequality 6f. In all three cases Inequality 6f is simplified to $R_m > -P_x$ that is true for all $R_m > 0$ and $Px > 0$.

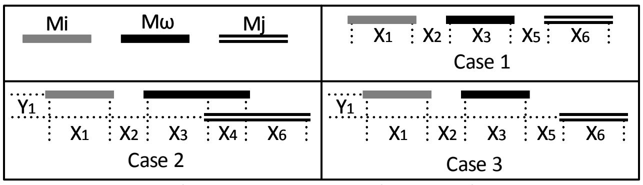

α	β	$N^{\alpha,\beta}_x$	$N^{\alpha,\beta}_g$	$N^{\alpha,\beta}_o$	L^α_β
i	k	X_2	0	0	$X_3 + X_4$
k	j	X_5	Y_1	1	X_6
i	j	$X_2 + X_3 + X_5$	Y_2	1	$X_4 + X_6$

Figure 4: Possible arrangements of MEMs for Equation 5a.

There is no need to prove Inequality5b since computation of S^j_i is skipped in Φ_{2A} of *DP-MEM* if $M_i \in H^j_*$. Note that for $M_i \in H^j_*$, M_i is fully overlapped by M_j.

XII The effect of sl on Omega

Figure 5 shows the example in which there are three Short MEM with length $sl-1$ (grey lines) appears before M_j. All these short MEMs have the same offset as M_j and surrounded by mismatches. If MEM shorter than sl are removed from the list of MEMs, then the proof in Section XI is no longer valid. In this example M_i is a member of $H^j_{OFS_i}$. However, S^j_ω is less than S^j_i which is in conflict with the proof in Section XI.

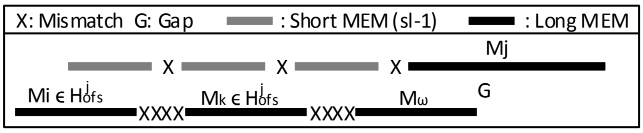

Figure 5: An example in which if short MEMs were removed, the Ω optimisation may result in suboptimal alignment.

Ω optimisation may result in suboptimal alignment when short MEMs are removed. However, considering that cases similar to Figure **??** are extremely rare and also taking into account the speed up achieved by Ω optimisation, we decided to leave both optimisation be applied to our implementation of *MEM-Align*.

To minimise the chance of producing suboptimal alignment, we extend the set Ω_j in the following way. For each $M_k \in \Omega$ we also exclude M_l in Ω if $OFS_k = OFS_l$ and EQ_l is maximum of EQ_x where M_x is a member of $H^j_{OFS_k}$. In other word, M_l is the immediate MEM before M_k in the same row (with the same offset).

XIII Implementation details

I Smith-Waterman

The SSW library [22] includes a test program that aligns every sequence of the target file with all sequences in query file. We modified this test program to only align i^{th} sequence in target file with i^{th} sequence in query file. We also comment unnecessary *printf* to make sure the algorithm deliver its maximum speed. This modified version along with the script that uses it is available in *MEM-Align* package. Since SSW does not apply gap extend penalty for the first gap the value of gap open penalty for this algorithm is set to the summation of required gap open penalty and gap extend penalty. For example if P_o and P_e are set to 6 and 1 for other algorithm we set P_o to 6+1 for SSW.

We also process input with KSW [**?**] (another SIMD implementation of Smith-Waterman form *Klib* library) for validation purpose. This implementation has been used in BWA. A similar modification is applied to KSW and the modified source code is available in *MEM-Align* package. Note that for KSW we do not need to change P_o as it apply both gap open and gap extend penalties for the first gap in a group of a continuous gap. In all experiment the resulting alignment score from KSW and SSW were identical. However, KSW is slightly slower than SSW.

II Ukkonen

SNAP [12] read mapper implements a modified version of Ukkonen that returns the alignment path in a CIGAR string format (refer to the SAM file format specification for details). We take this implementation of Ukkonen for evaluation. Thus the SNAP source code is modified to only execute the Ukkonen alignment function for input sequence pairs instead of running the entire mapping process. The modified version of SNAP is available in *MEM-Align* package. The CIGAR string is processed by a program (written in C and available in *MEM-Align* package) to compute the alignment score based on a given scoring values. Note that in the CIGAR string produced by this implementation of Ukkonen matches and mismatches are represented with different symbols ("=", "X" respectively); thus the alignment score can be computed from the CIGAR string. The execution time for computing alignment scores from CIGAR strings is not included in the execution time of Ukkonen algorithm.

III Gene Myers

We used an implementation of Gene Myers algorithm from the SeqAN package. This implementation is also used in [36] for evaluation purposes. In order to make use of the functions available in this implementation, a computer program (written in C and available in *MEM-Align* package) is developed which call the alignment function for the input sequence pairs. Note that Gene Myers is implemented as a global alignment in the SeqAN package and the reported alignment score is a global alignment score which cannot be compared against the semi-global alignment score produced by Smith-Waterman. In order to compute the semi-global alignment score for the alignment produced by Gene Myers we print the alignment and then a computer program (written in C and available in *MEM-Align* package) compute semi-global alignment score for the produced alignment. To be accurate when computing semi-global alignment score, we exclude leading and trailing part of the alignment to get the highest possible score. Since printing alignment is a time-consuming file operation, for a fair comparison of execution time, we first processed the input sequence pairs without printing out the alignment and recorded the execution time. Then we process input once again to print out the alignment.

IV Gene Myers combined with Hirschberg

SeqAn package also implements a version of Gene Myers algorithm combined with Heisenberg algorithm. We also implement this algorithm in a similar fashion we implement the Gene Myers algorithm.

XIV Alignment representation with colours

Figure 6 represents seven alignments generated by *MEM-Align*. For each alignment, the first two lines show the aligned sequences. The third line identifies matches, mismatches, insertions, deletions and clipped bases (those excluded from alignment) with letters 'M', 'X', 'I', 'D', and

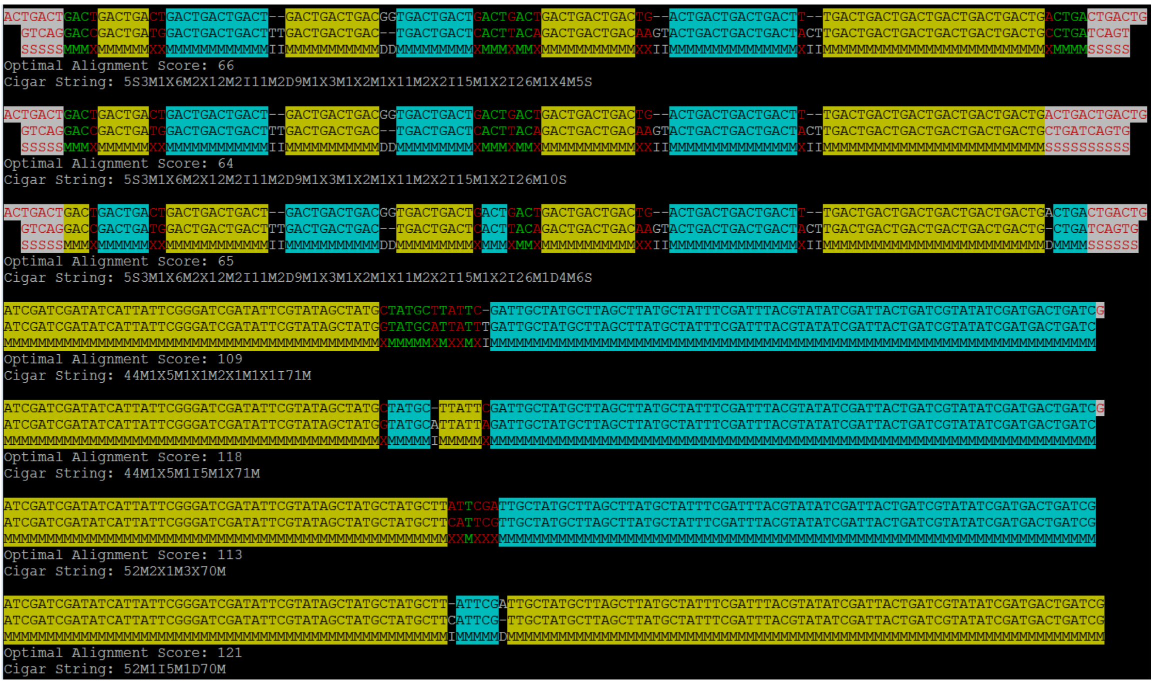

Figure 6: Example output form *MEM-Align* containing seven alignments represented in colours along with cigar strings and alignment scores.

'S' respectively. The alignment score and the CIGAR string are reported in the fourth and fifth line of each alignment.

The first alignment (from top) is a case showing different situations that MEM align can deal with them. White background shows the clipped regions at the beginning and the end of the alignment. Blue and yellow background are exact matches that form the alignment. Two different colours are used to simplifying alignment visualisation. Bases between these exact matches were not extracted as a MEM at the time of MEM extraction process. These are short MEMs removed due to *sl*. Those bases represented with red are actual mismatches and those represented by green are recovered matches which are recovered during a sequential string compare operation. As shown in the first alignment, there are matches before and after the first and the last exact match (respectively) in the alignment which is separated by some mismatches. These matches are also recovered using a sequential comparison.

The second, the fourth and the sixth alignments show examples where sequential string compare operation of *MEM-Align* fails to identify the correct matches in the regions where short MEMs are removed due to a large *sl* value. The third, fifth and seventh alignments are correct alignments (of the same sequences in the second, the fourth and the sixth alignments respectively) when a smaller *sl* value is used during the alignment.

In the second alignment, there are some matches in the clipped region on the right side of alignment. However, these matches are not recovered as they are separated from the last exact match due to a gap. In the third alignment, a smaller *sl* value is used to align the same sequences in the second alignment. This change in the value of *sl* results in identifying true matches as an individual MEM and subsequently producing correct alignment. Note that in the third alignment, those recovered matches (green bases) at the middle and left side of the second alignment are now considered as separate exact matches too.

The fourth alignment shows a case where two mismatches and a gap at the middle result in the elimination of MEMs in the middle of alignment. Since the gap is located in the middle of this area the sequential compare operation is not capable of correctly identify the true alignment of that region and subsequently *MEM-Align* produce a suboptimal alignment. However, when the same sequences are aligned with a smaller value for *sl* (in the fifth alignment) the alignment is reported by the *MEM-Align*. Similarly, the last two alignments in Figure 6 show how the sequential string compare operation of *MEM-Align* fails to identify the correct alignment when there are two gaps close to each other and result in short MEM which is removed due to *sl*. Note that the last alignment is the alignment produced by a small *sl* value.

XV Complete experimental results

I Comparing Methods

In addition to algorithms compared to *MEM-Align* in the original paper, we also consider an implementation of Smith-Waterman from K-lib (KSW) [?]. KSW is reliable as it has been used by BWA mapper so we use it for verification purpose.

Figures 8 , 9 and 10 represent execution time, number of suboptimal alignments and average alignment score difference in suboptimal alignments respectively where results are grouped by dataset. Figures 11 , 12 and 13 are as same as Figures 8 , 9 and 10 respectively with results grouped by algorithm.

Figures 14 , 15 and 16 are scaled version of Figures 8 , 9 and 10 respectively and Figures 11 , 12 and 13 are scaled

version of Figures 11 , 12 and 13 respectively all of which are provided to better visualise results.

While the execution time of SSW is quadratic in the length of the sequence, the UKK execution time seems to be linear in sequence length. The *MEM-Align* execution time is a more complex function and depends on other factors such as error rate and given parameters. Although UKK is the fastest algorithm, *MEM-Align* results in a considerably lower amount of suboptimal alignments and stays in second place when comparing execution time. The average alignment score difference is relatively high for MA2 processing longer sequences. However, considering the small number of suboptimal alignments in MA2, this high average alignment score difference should not be considered as a negative point.

Figure 20 represent normalised execution time in which the execution time of DLL, DLH, and DRQ datasets are divided by the execution time of DSL, DSH, DSL datasets respectively. normalised execution time are shown in log_2 scale. Since sequence length are 125, 250 and 500 (1X, 2X, and 4X), it is possible to see how the length of sequences affects the execution time of the algorithm. SSW and KSW are clearly a quadratic algorithm with complexity $O(n^2)$ (n is the length of input sequences). The execution time of GM, UKK seems to be linear ($O(n)$). Although sequence length can affect the execution time of *MEM-Align*, the difference between the value of orange and yellow bar for MA1 shows that other factors such as error rate and the given parameter are more likely to be influential.

The default scoring is $R_m = 1$, $P_x = 4$, $P_o = 6$ and $P_e = 1$. Results for algorithm with a star ('*') in algorithm name represent cases where $P_x = 2$ and $P_o = 8$ are considered. The new scoring scheme affects MA1 and MA2 in a completely different way. In MA2 the new scoring value results in being more accurate and producing less suboptimal alignment as it is less likely that gaps close to each other appear in the alignment (because of higher P_o and lower Px). In contrast to our expectation, the number of suboptimal alignments in MA1 is increased with new scoring value. This increase in the number of suboptimal alignments is due to the increase in the alignment score of all alignments (subsequently suboptimal alignments) that results in fewer number of suboptimal alignments exceed TS threshold and being bypassed to SSW. This increase in alignment score also occurs in MA2. However, the value of TS in MA2 is less sensitive to this change in alignment score. Figure 7 shows the increase in the number of sequence pairs bypassed to SSW by TS in MA1 and MA2 compared with MA1* and MA2* respectively. Note that execution time is only shown for default scoring values.

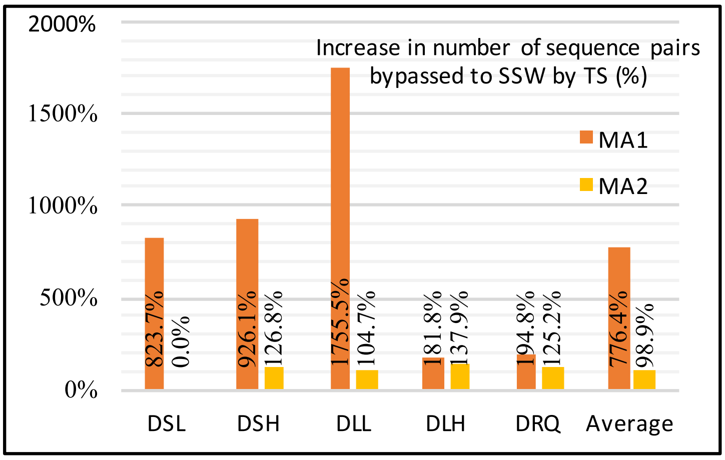

Figure 7: Increase in number of sequence pairs bypassed to SSW by TS in MA1 and MA2 compared with MA1* and MA2* respectively.

II Results for individual dataset

Since a large number of figures are provided in this section. Since similar figures are provided for each dataset, Table 1 grouped figures by dataset (in columns). Figures in each row of Table 1 represent specific measurement. In the flowing, we explain each of which measurement in details. Note that the default value of each parameter (INF) is set as follows unless otherwise mentioned: $sl = 4$; $TM = 1000000$, $TD = 1000$ and $TS = 0$. Default values for TM, TS and TD were set in order to make them ineffective meaning that the related optimisation is disabled.

Row 1, 2 and 3 represent execution time, number of suboptimal alignments and average alignment score difference in suboptimal alignments respectively when gl and sl vary. Row 4 and 5 are scaled version of row 1 and 2 respectively and provided for efficient data visualisation. Comparisons of $sl < 4$ with other values of sl, and of $gl = 3$ with $gl = 6$ show that execution time and accuracy are extremely sensitive to the values of gl and sl. Also, note that none of these parameters can compensate for the effect of the other if the other is not chosen optimally. As a result, both of these value must be chosen with care.

Row 6, 7 and 8 represent execution time, number of suboptimal alignments and average alignment score difference in suboptimal alignments respectively when TM varies. Note that optimal value of TM is a function of the average number of extracted MEM. As a result, we also vary gl in these figures to change the average number of MEM and subsequently its combined effect with TM. Note that $TM = INF$ indicate the case where TM optimisation is disabled.

TM is initially proposed to reduce the execution time of *MEM-Align*. Before introducing set Ω and its related optimisation, *MEM-Align* was a quadratic algorithm on the number of extracted MEM. Applying large gl and small sl to the algorithm result in numerous number of MEMs to be extracted (more than the number of bases in the sequences). In such cases, it was more efficient to bypass sequences to Smith-Waterman which is a quadratic algorithm in length of sequence rather than processing them with *MEM-Align* (without Ω optimisation) which was a quadratic algorithm on the number of extracted MEM. Thus applying TM results in *MEM-Align* being faster. However, after applying Ω optimisation, *MEM-Align* becomes less sensitive to the number of extracted MEM and TM optimisation lose its effect.

The reason TM still exists is that we find *MEM-Align* less accurate when aligning repetitive sequences and TM is an effective method to identify such sequences. As as result, an appropriate value of TM could improve accuracy with a slight increase in execution time. For example in DSL dataset,

with $TM = 40$ compare to having no TM ($TM = INF$) execution time is increased 1.37 second (9%) while the number of suboptimal alignment is reduced by 12 (22%).

Row 9, 10 and 11 represent execution time, number of suboptimal alignments and average alignment score difference in suboptimal alignments respectively when TD varies. Since the optimal value of TD is affected by the value of gl, we also consider changing gl in these figures (expecting larger gap means expecting larger distance). Note that $TD = INF$ indicate the case where TD optimisation is disabled. except for $TD \leq 10$, while having no effect on number of suboptimal alignments, TD can reduce execution time slightly. This effect was expected as TD is responsible for avoiding those alignment extensions which are not likely to contribute to the alignment. Note that small value of TD avoids removed short MEMs in the alignment of being recovered using string compare operation. $TD = 0$ is the case that only overlapped or adjacent MEMs are extended.

Row 12, 13 and 14 represent execution time, number of suboptimal alignments and average alignment score difference in suboptimal alignments respectively when TD varies. Since TS is designed to compensate for the adverse effect of banded alignment optimisation, we also consider changing the value of gl in these figures. In these figures the TD is set to 25 and TM is set as in Table 4 of original paper. As shown in these figures TM dramatically reduce the number of suboptimal alignment with a dramatic increase in execution time. The effect of TS is better shown in Row 35 and 36 which represent execution time and the number of suboptimal alignments with and without applying TS. Row 37 is scaled version of Row 36 for better visualisation. As shown in these figures, in addition to a general decrease in the number of suboptimal alignments when applying TS, the number of suboptimal alignments is less sensitive to the value of gl meaning that with banded alignment we could reach almost the same accuracy as normal alignment.

Row 15 represents the average number of extracted MEMs (Θ) when gl and sl vary which is in direct relation to the execution time. Θ is a helpful guideline for identifying the optimal value of TM. Although the exact function has not been identified yet, it appears that $4\Theta < TM < 5\Theta$ is a suitable estimation.

Row 16 represents the average number of extracted MEMs when TM and gl vary. The average is over those sequence pairs processed by *MEM-Align* (are not bypassed to SSW) which is in direct relation with execution time of the alignment phase.

Row 17 illustrates the percentage of alignment extension operations which are avoided because of the set Ω. Row 18 shows that (after applying Ω how much of the alignment extensions are optimised using TD. Row 19 shows the percentage of string compare operations which are optimised.

Row 20 shows the number of sequence pairs bypassed to SSW because of TM. Combined with Row 16 these figures represent how TM is expected to affect the proportion of execution time that *MEM-Align* spent on alignment phase. Row 21 shows the total number of sequence pairs bypassed to SSW and Row 22 shows how much of those are bypassed to SSW by TS (the rest are bypassed to SSW by TM). Row 23 shows the proportion of sequence pairs bypassed to SSW by TM and TS and Row 24 is scaled version of Row 23.

Row 25, 27, 29, 31 and 33 report the effect of gl, sl, TM, TD and TS on execution time of differing phases of *MEM-Align* respectively. Data in these figures is obtained by counting the number of cycles that CPU spent on each algorithmic step of the program. Row 26, 28, 30, 32 and 34 are as same as Row 25, 27, 29, 31 and 33 respectively where all bars are normalised to a 100% bar to better visualise the proportion of execution time spent on each algorithmic phase.

gl seems to affects all phases equally. sl effect the alignment phase dramatically. Decreasing TM increases the time spent on SSW and at the same time, it reduces the time spent on alignment phase. This is because TM bypass sequences before they get processed by *MEM-Align* alignment phase. As expected, TD only effect the alignment phase. TS has a similar effect as TM in reverse direction except that TS does not affect alignment phase since sequences are bypassed after *MEM-Align* finish processing them.

Table 1: Figures related to results for each dataset

Row	DSL	DSH	DLL	DLH	DRQ
1	Figure 21	Figure 58	Figure 95	Figure 132	Figure 169
2	Figure 22	Figure 59	Figure 96	Figure 133	Figure 170
3	Figure 23	Figure 60	Figure 97	Figure 134	Figure 171
4	Figure 24	Figure 61	Figure 98	Figure 135	Figure 172
5	Figure 25	Figure 62	Figure 99	Figure 136	Figure 173
6	Figure 26	Figure 63	Figure 100	Figure 137	Figure 174
7	Figure 27	Figure 64	Figure 101	Figure 138	Figure 175
8	Figure 28	Figure 65	Figure 102	Figure 139	Figure 176
9	Figure 29	Figure 66	Figure 103	Figure 140	Figure 177
10	Figure 30	Figure 67	Figure 104	Figure 141	Figure 178
11	Figure 31	Figure 68	Figure 105	Figure 142	Figure 179
12	Figure 32	Figure 69	Figure 106	Figure 143	Figure 180
13	Figure 33	Figure 70	Figure 107	Figure 144	Figure 181
14	Figure 34	Figure 71	Figure 108	Figure 145	Figure 182
15	Figure 35	Figure 72	Figure 109	Figure 146	Figure 183
16	Figure 36	Figure 73	Figure 110	Figure 147	Figure 184
17	Figure 37	Figure 74	Figure 111	Figure 148	Figure 185
18	Figure 38	Figure 75	Figure 112	Figure 149	Figure 186
19	Figure 39	Figure 76	Figure 113	Figure 150	Figure 187
20	Figure 40	Figure 77	Figure 114	Figure 151	Figure 188
21	Figure 41	Figure 78	Figure 115	Figure 152	Figure 189
22	Figure 42	Figure 79	Figure 116	Figure 153	Figure 190
23	Figure 43	Figure 80	Figure 117	Figure 154	Figure 191
24	Figure 44	Figure 81	Figure 118	Figure 155	Figure 192
25	Figure 45	Figure 82	Figure 119	Figure 156	Figure 193
26	Figure 46	Figure 83	Figure 120	Figure 157	Figure 194
27	Figure 47	Figure 84	Figure 121	Figure 158	Figure 195
28	Figure 48	Figure 85	Figure 122	Figure 159	Figure 196
29	Figure 49	Figure 86	Figure 123	Figure 160	Figure 197
30	Figure 50	Figure 87	Figure 124	Figure 161	Figure 198
31	Figure 51	Figure 88	Figure 125	Figure 162	Figure 199
32	Figure 52	Figure 89	Figure 126	Figure 163	Figure 200
33	Figure 53	Figure 90	Figure 127	Figure 164	Figure 201
34	Figure 54	Figure 91	Figure 128	Figure 165	Figure 202
35	Figure 55	Figure 92	Figure 129	Figure 166	Figure 203
36	Figure 56	Figure 93	Figure 130	Figure 167	Figure 204
37	Figure 57	Figure 94	Figure 131	Figure 168	Figure 205

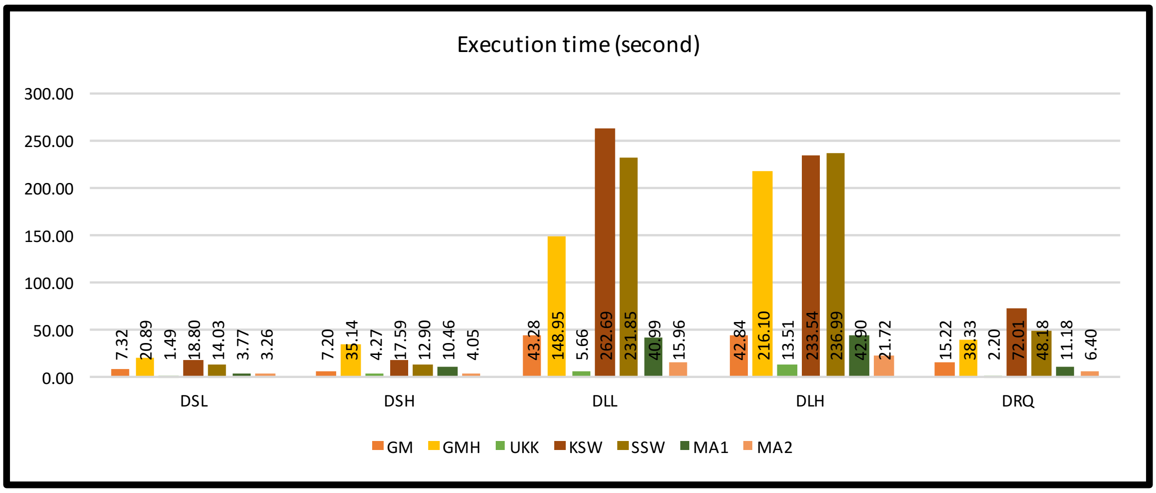

Figure 8: Execution times grouped by dataset.

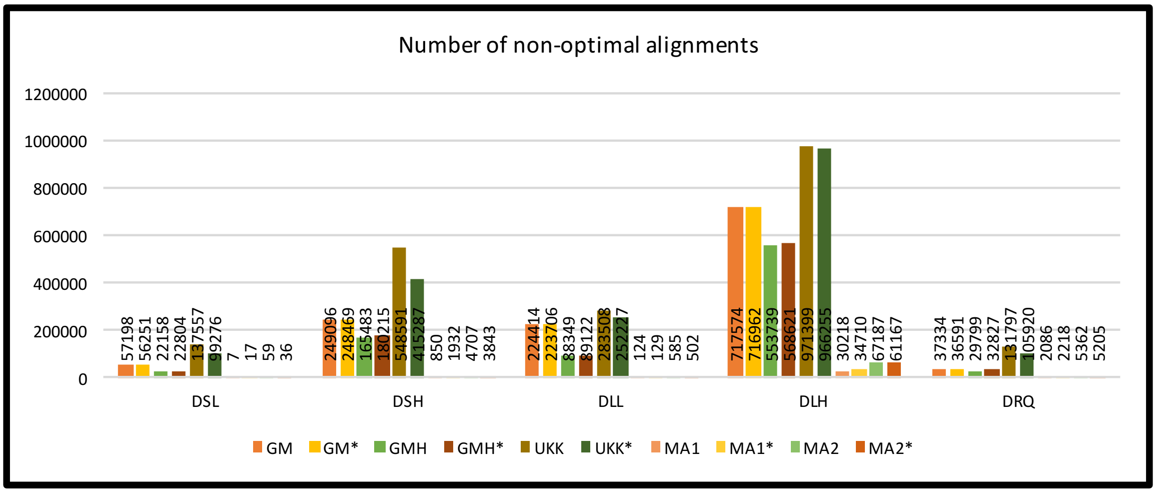

Figure 9: Number of suboptimal alignments grouped by dataset.

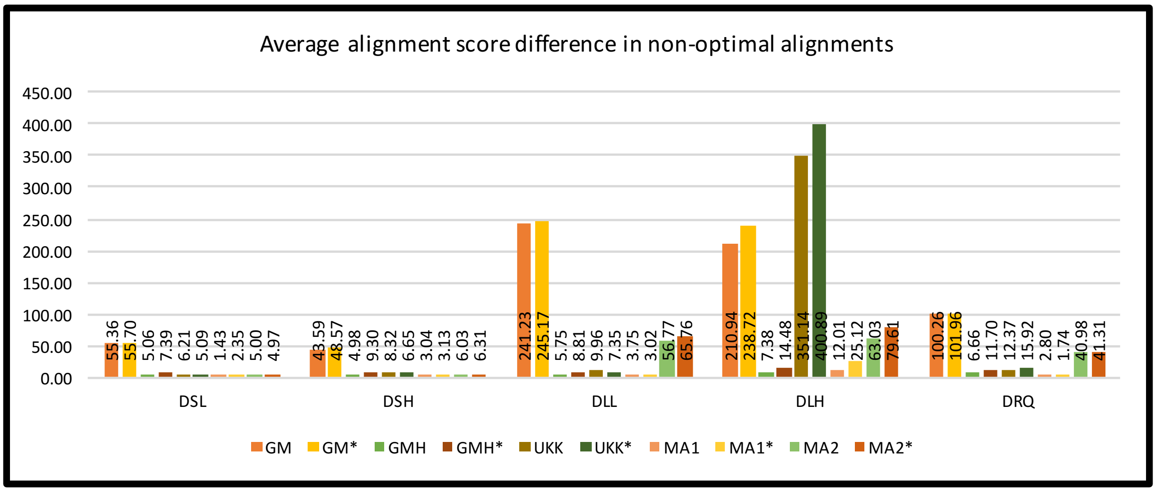

Figure 10: Average alignment score difference in suboptimal alignments grouped by dataset.

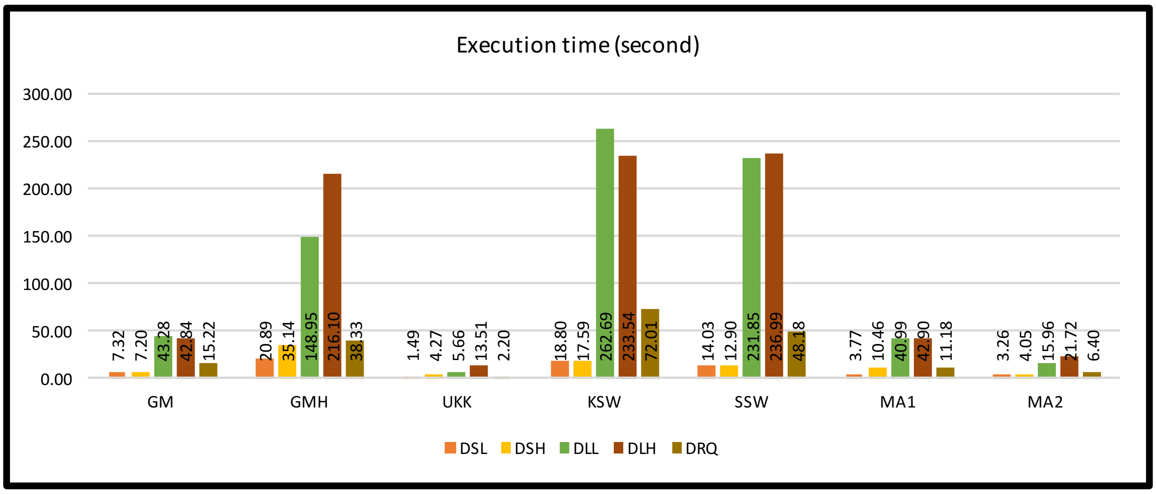

Figure 11: Execution times grouped by algorithm.

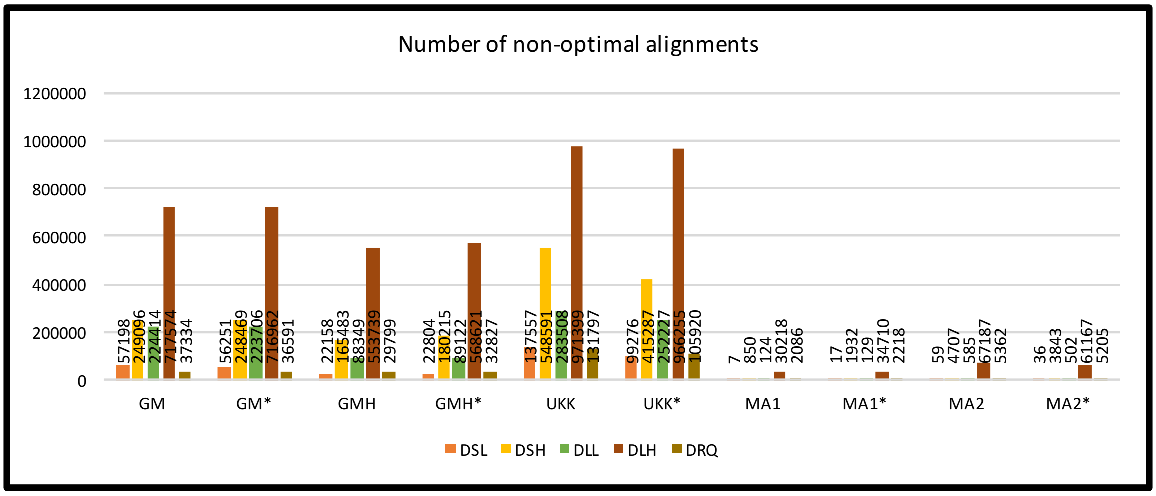

Figure 12: Number of suboptimal alignments grouped by algorithm.

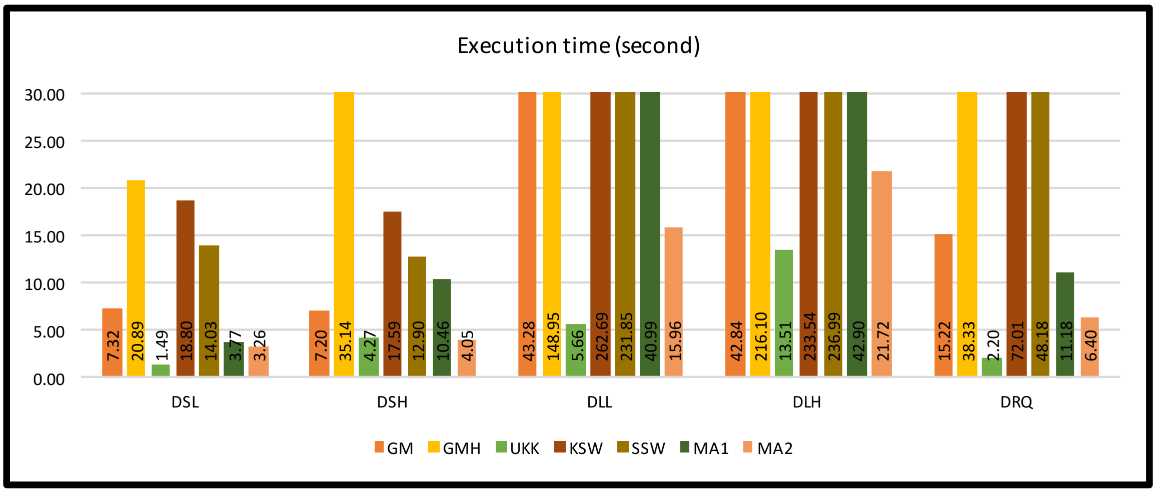

Figure 13: Average alignment score difference in suboptimal alignments grouped by algorithm.

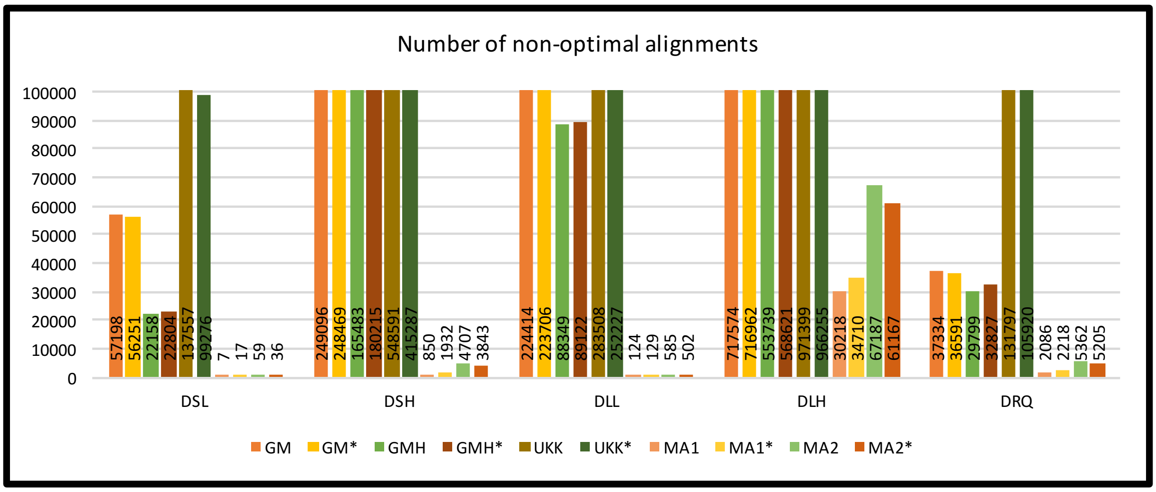

Figure 14: Execution times grouped by dataset.

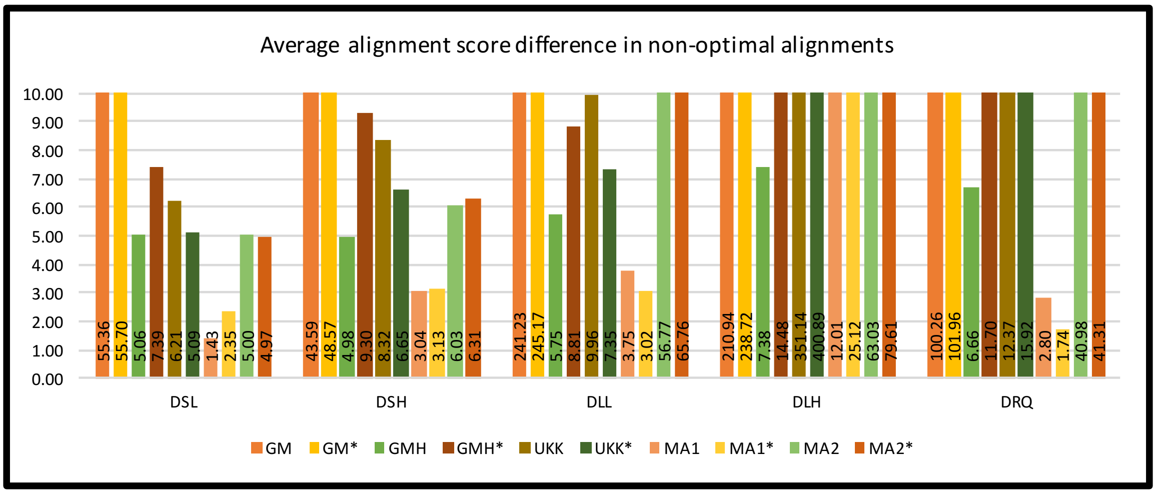

Figure 15: Number of suboptimal alignments grouped by dataset.

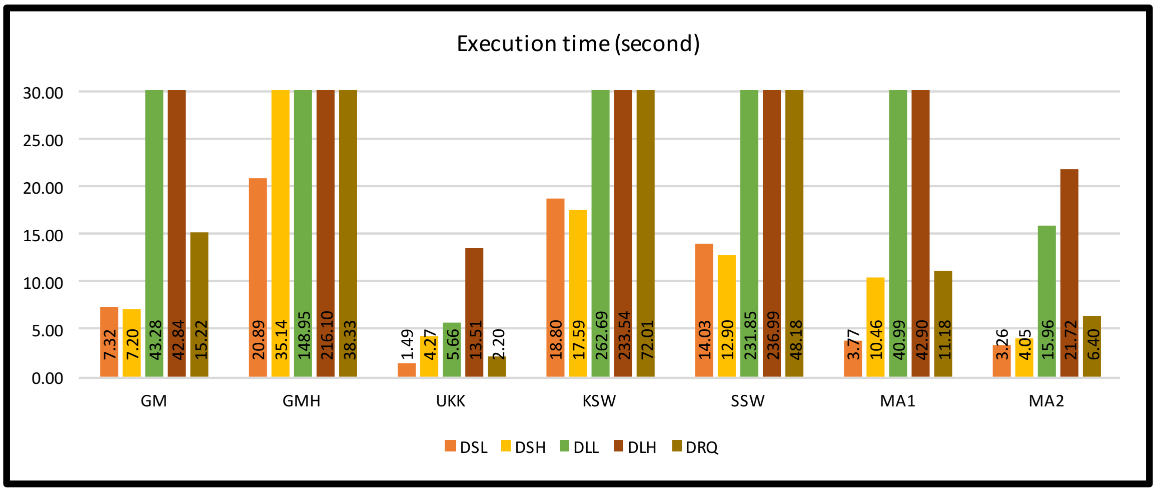

Figure 16: Average alignment score difference in suboptimal alignments grouped by dataset.

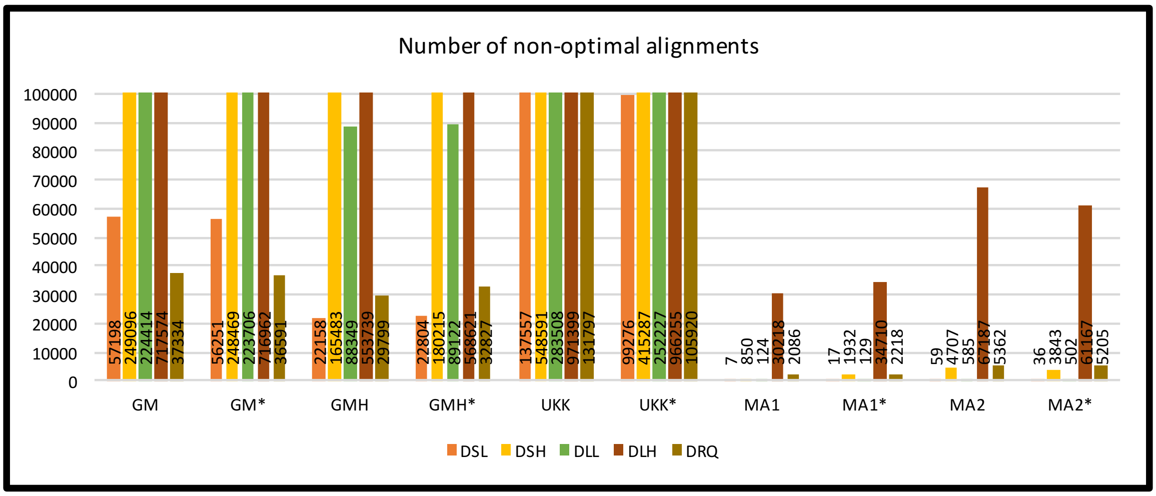

Figure 17: Execution times grouped by algorithm.

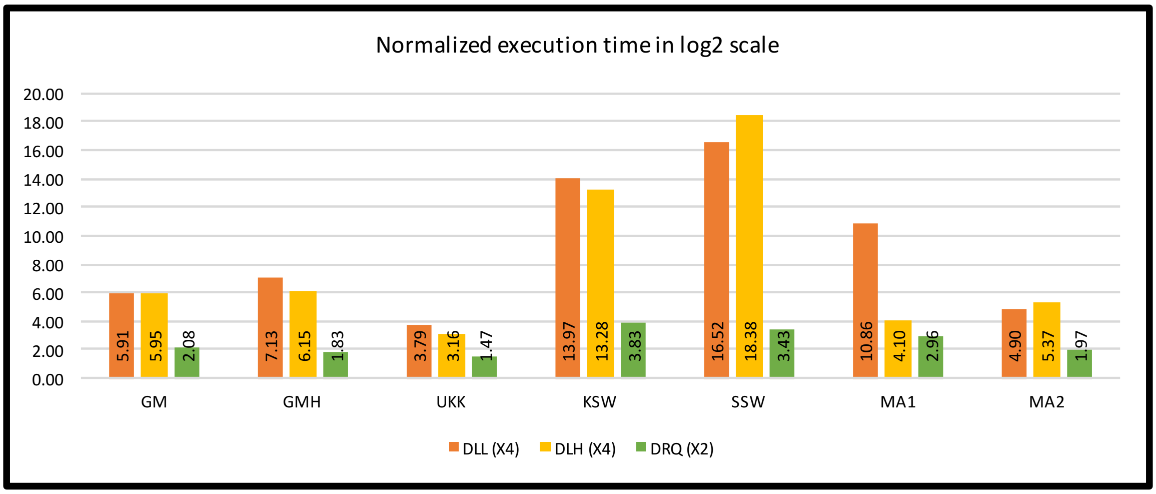

Figure 18: Number of suboptimal alignments grouped by algorithm.

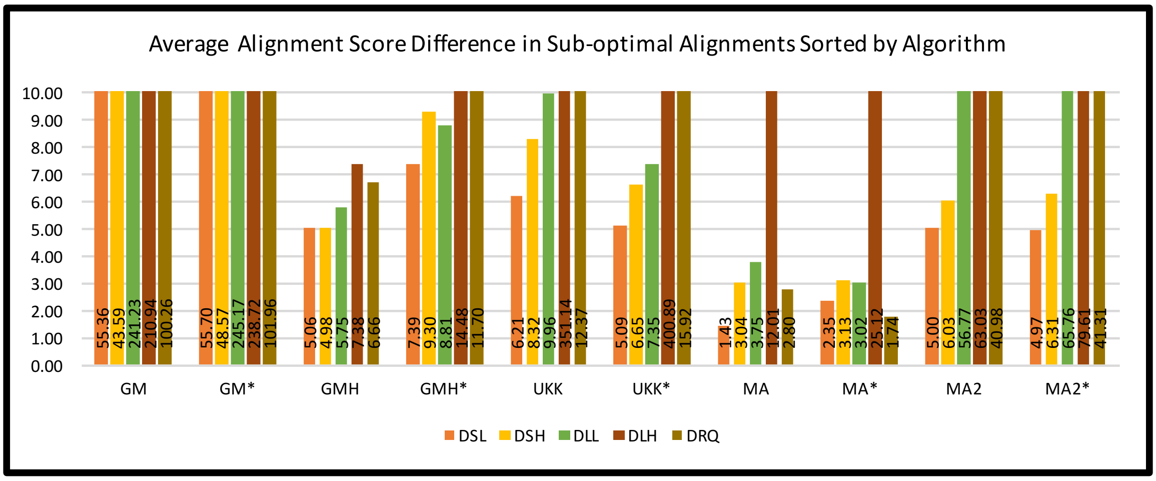

Figure 19: Average alignment score difference in suboptimal alignments grouped by algorithm.

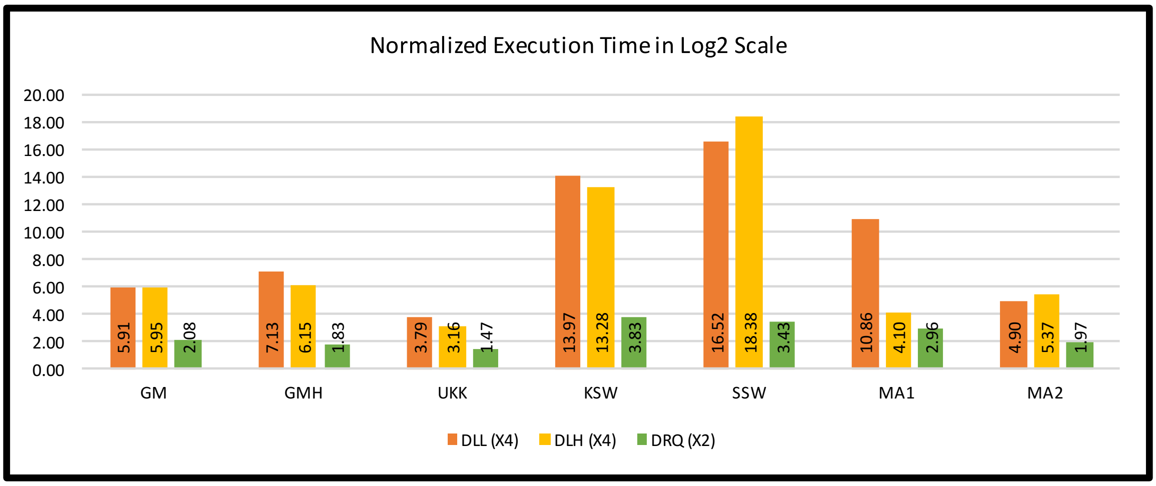

Figure 20: normalised execution time.

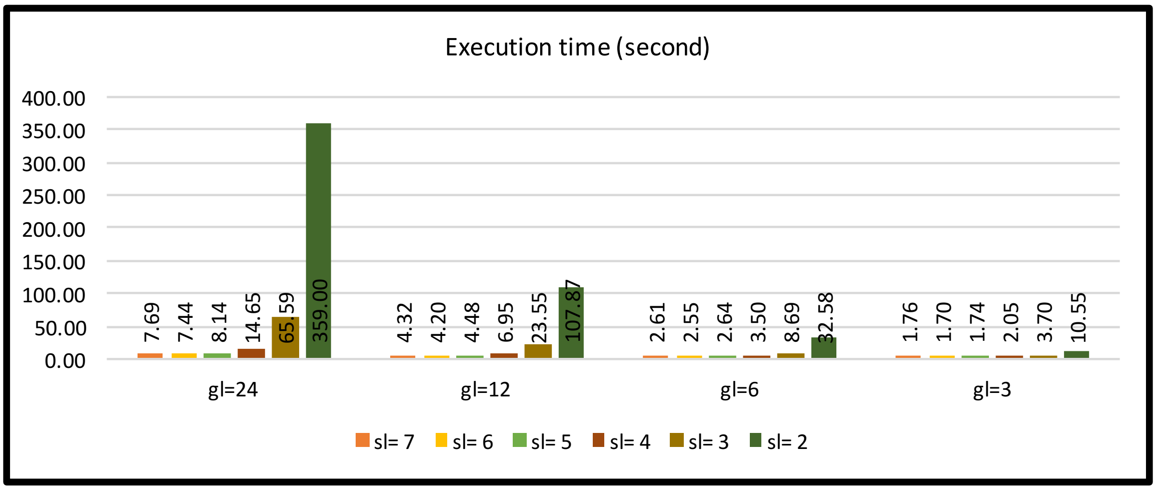

Figure 21: Execution times for DSL dataset when gl and sl varies.

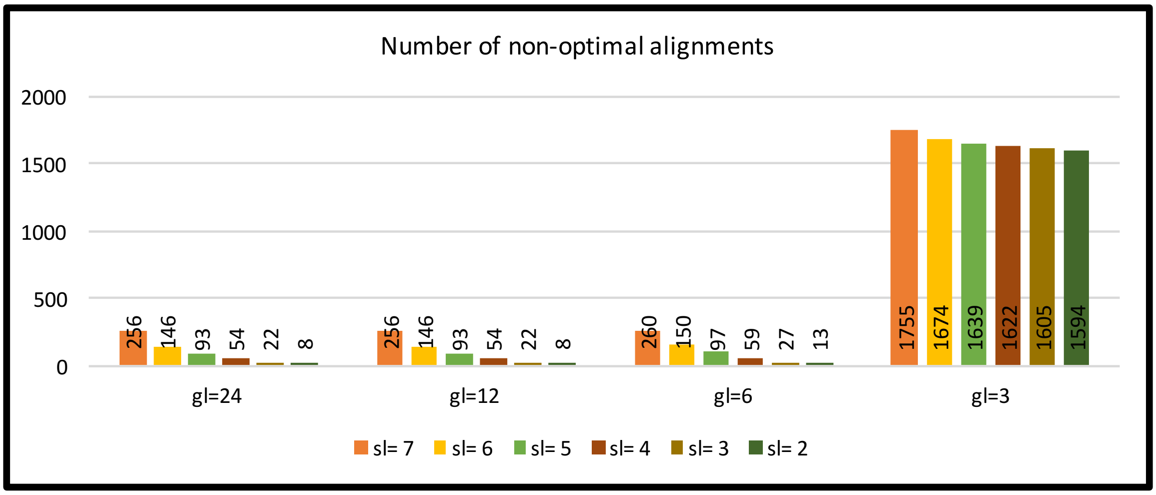

Figure 22: Number of suboptimal alignments for DSL dataset when gl and sl varies.

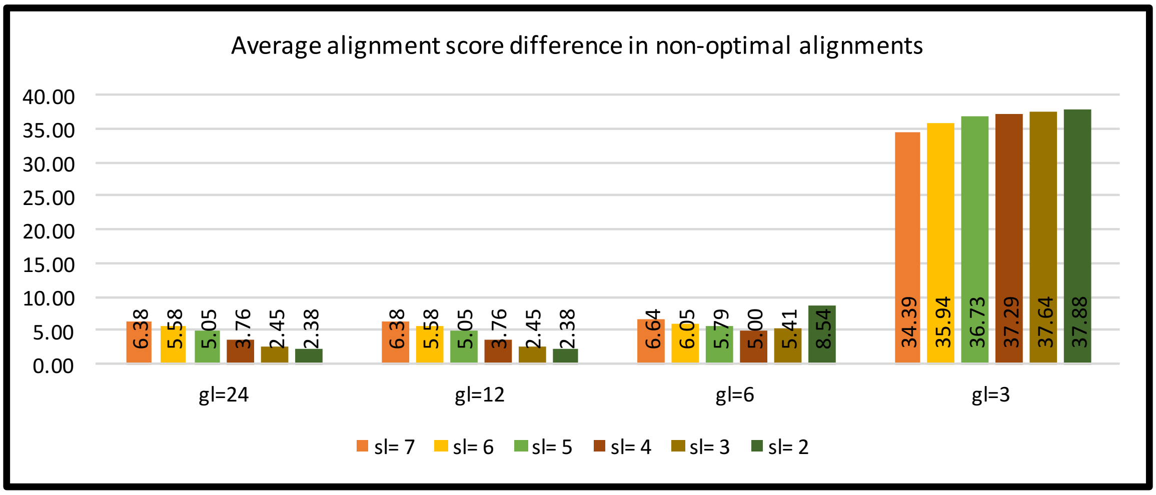

Figure 23: Average alignment score difference in suboptimal alignments for DSL dataset when gl and sl varies.

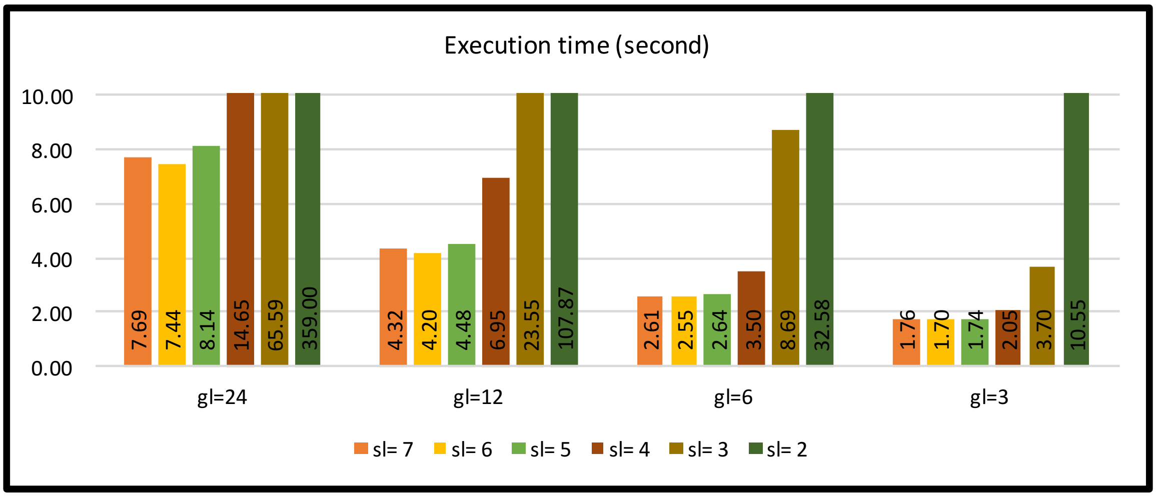

Figure 24: Execution times for DSL dataset when *gl* and *sl* varies (scaled).

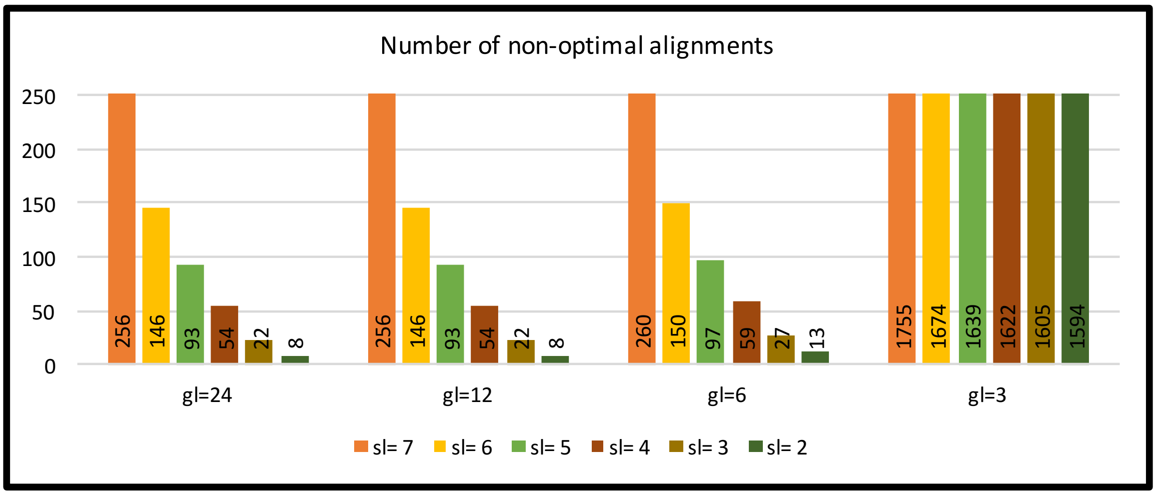

Figure 25: Number of suboptimal alignments for DSL dataset when *gl* and *sl* varies (scaled).

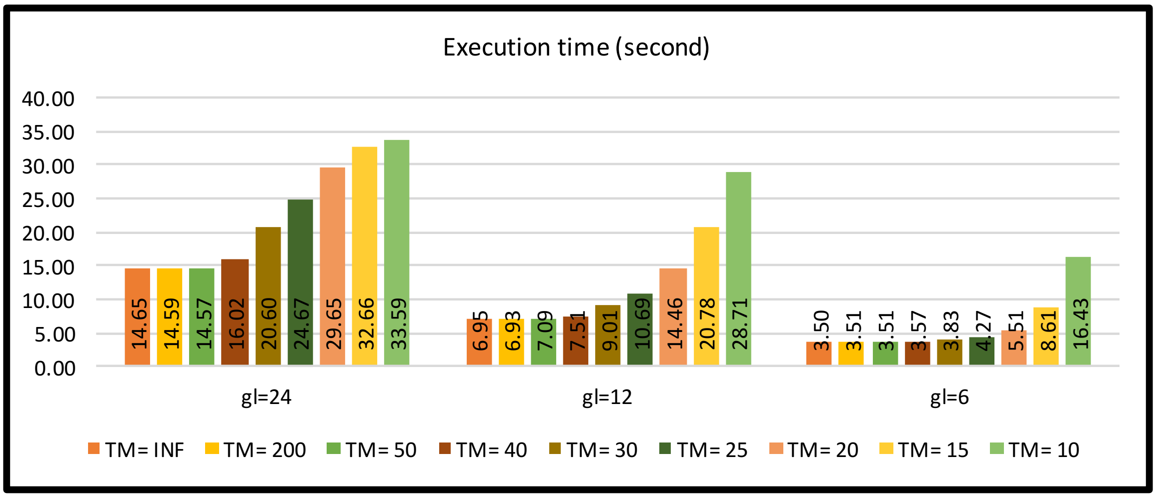

Figure 26: Execution times for DSL dataset when TM varies.

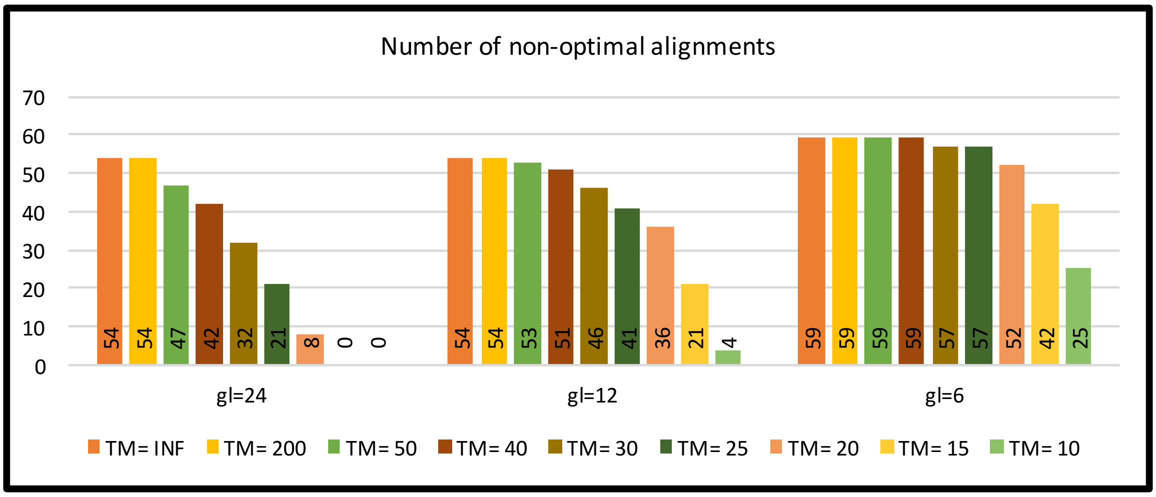

Figure 27: Number of suboptimal alignments for DSL dataset when TM varies.

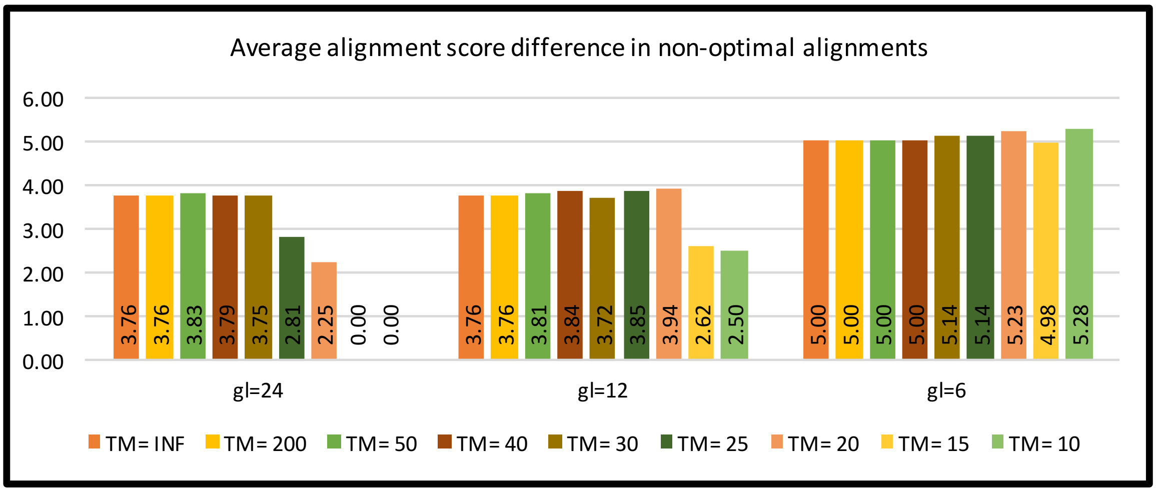

Figure 28: Average alignment score difference in suboptimal alignments for DSL dataset when TM varies.

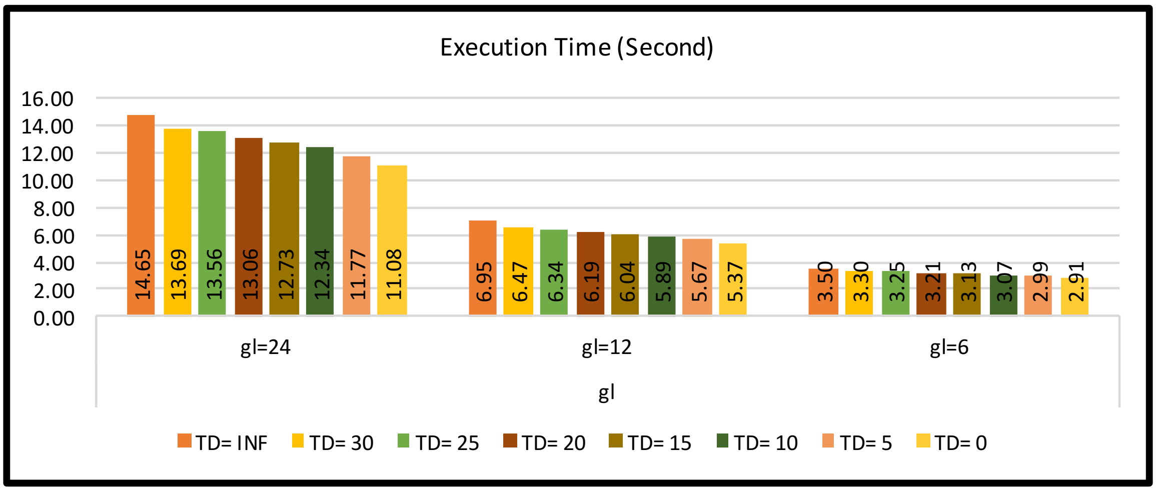

Figure 29: Execution times for DSL dataset when TD varies.

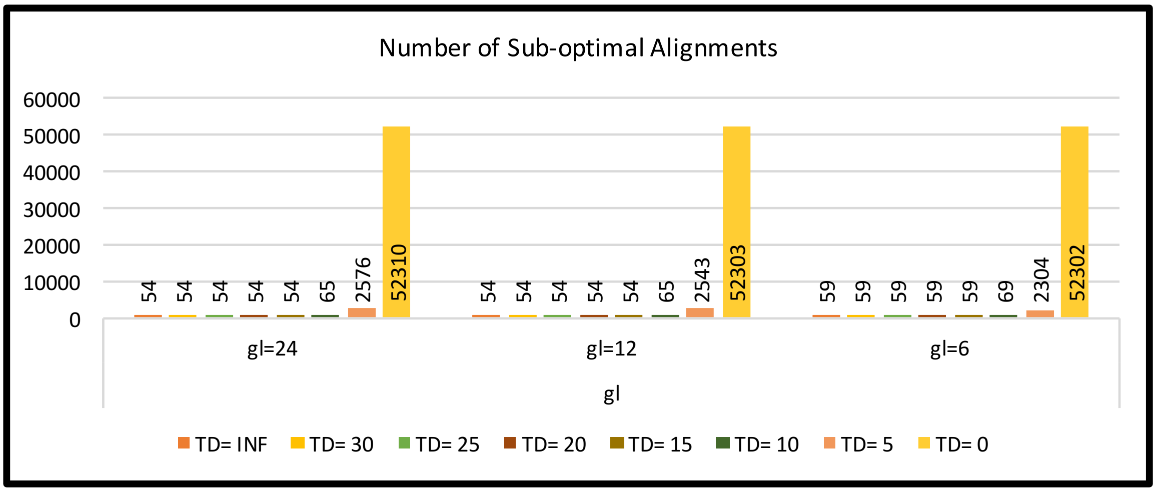

Figure 30: Number of suboptimal alignments for DSL dataset when TD varies.

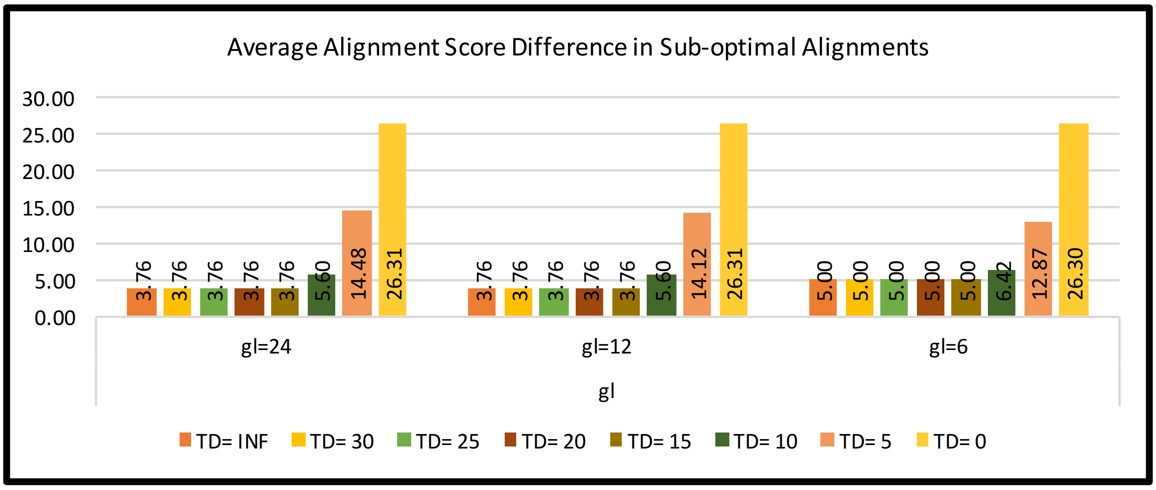

Figure 31: Average alignment score difference in suboptimal alignments for DSL dataset when TM varies.

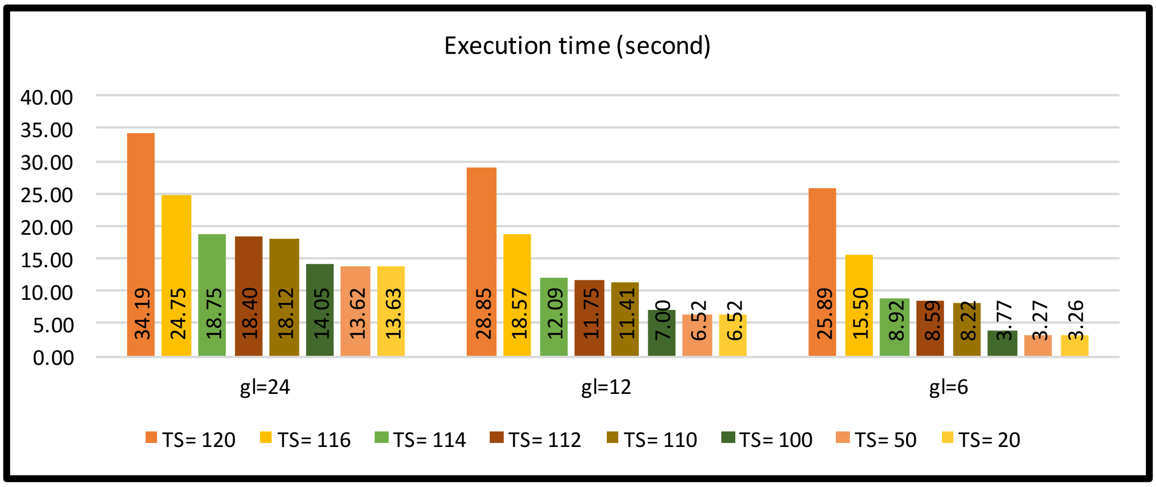

Figure 32: Execution times for DSL dataset when TS varies.

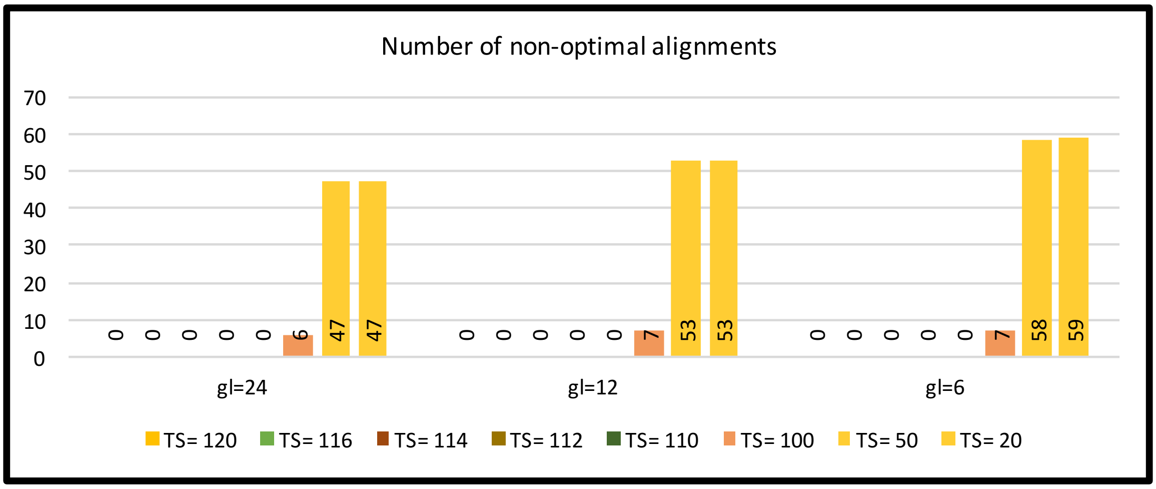

Figure 33: Number of suboptimal alignments for DSL dataset when TS varies.

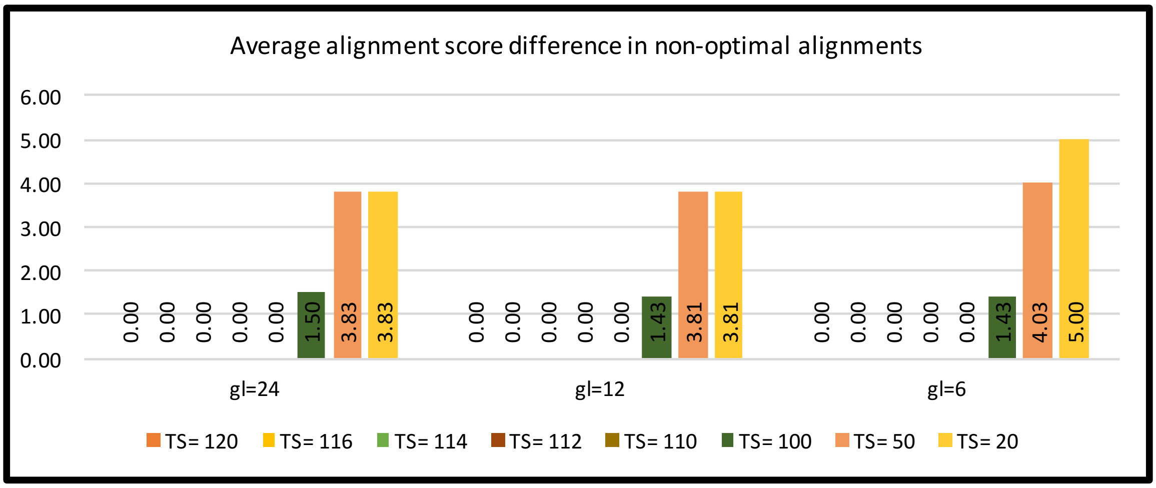

Figure 34: Average alignment score difference in suboptimal alignments for DSL dataset when TS varies.

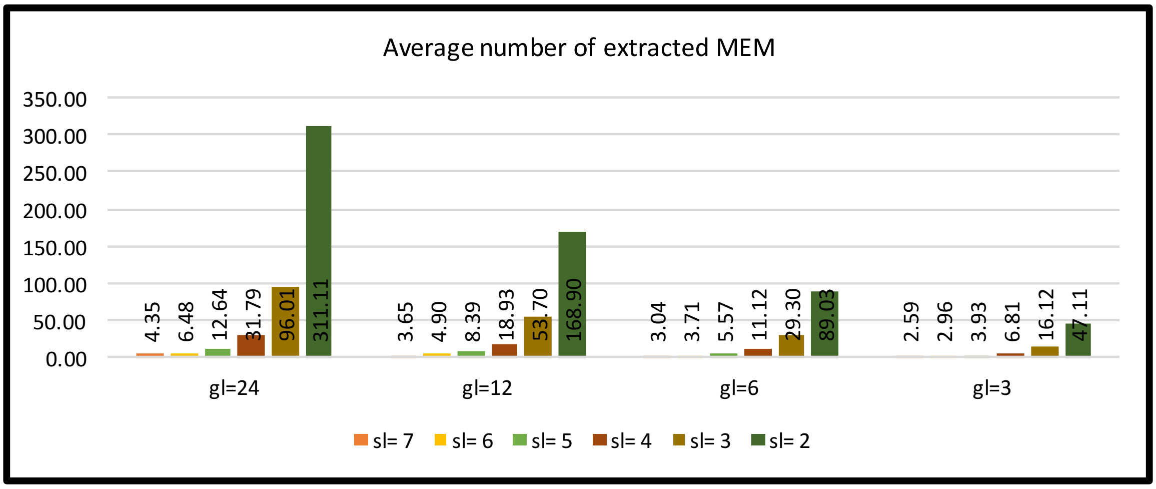

Figure 35: Average number of extracted MEM for DSL dataset when gl and sl varies.

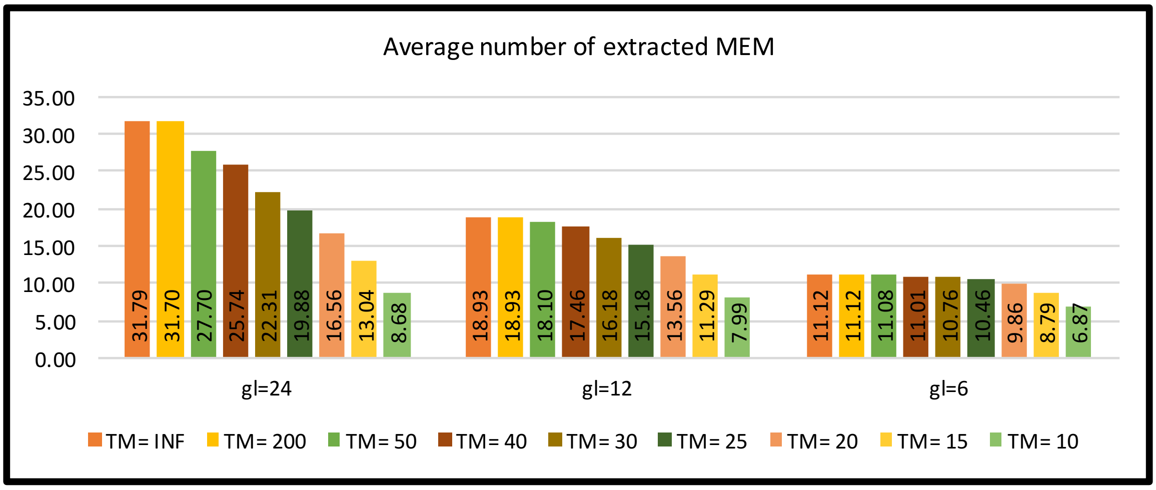

Figure 36: Average number of extracted MEM for DSL dataset when TM varies.

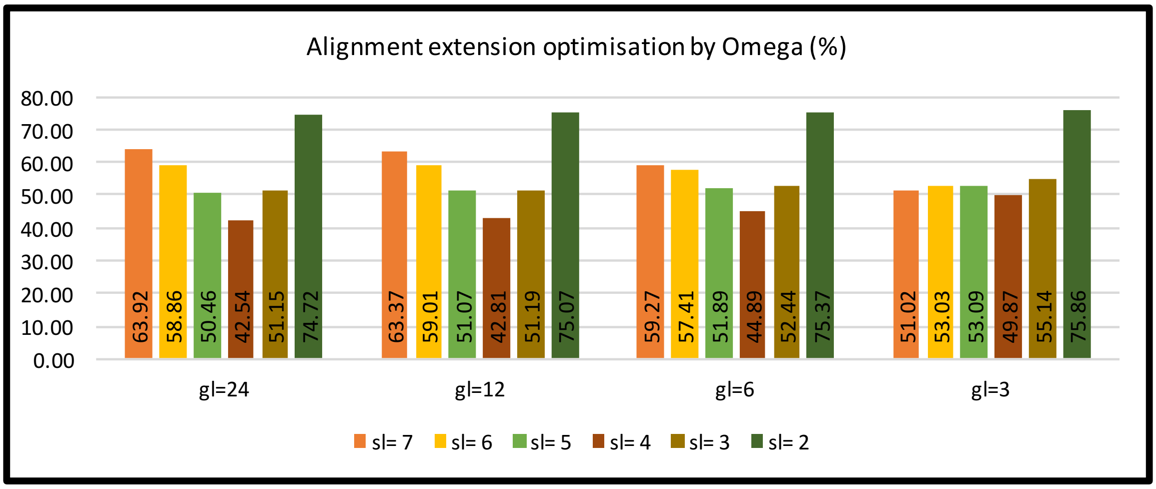

Figure 37: Proportion of alignment extension which are optimised (avoided) by the set Ω when processing DSL dataset.

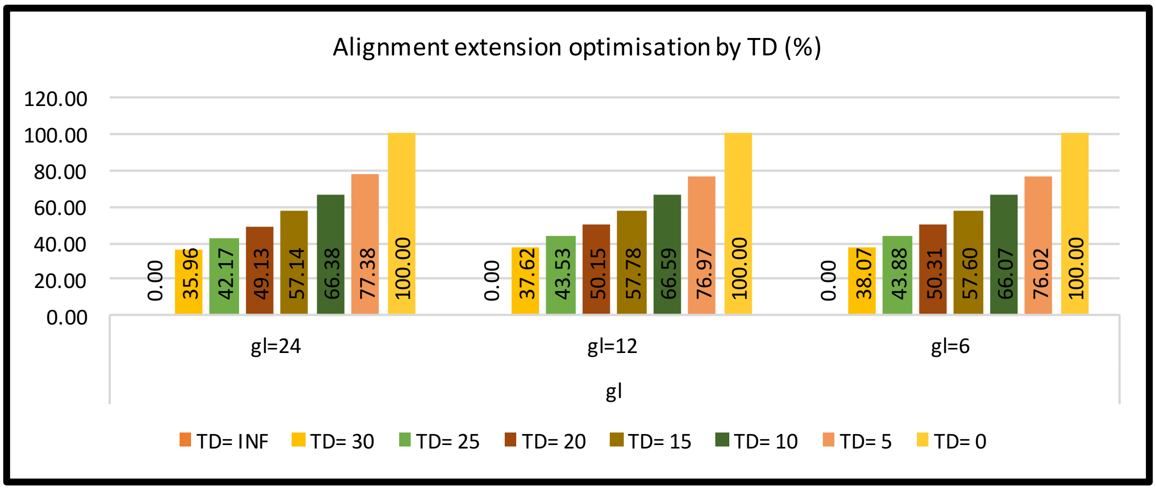

Figure 38: Proportion of alignment extension which are optimised (avoided) by TD after applying the set Ω when processing DSL dataset.

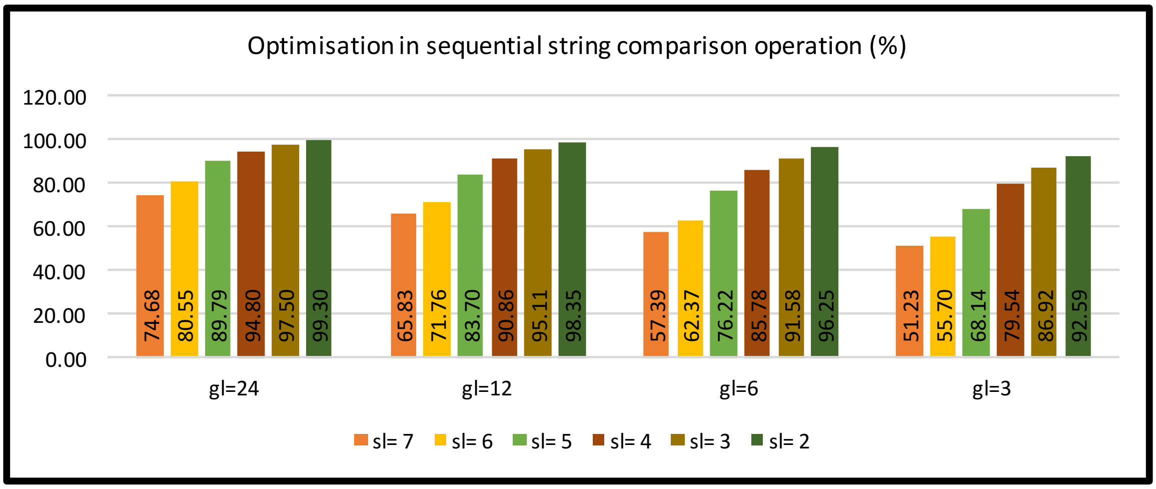

Figure 39: Proportion of sequential string compare operation which are optimised (avoided) when processing DSL dataset.

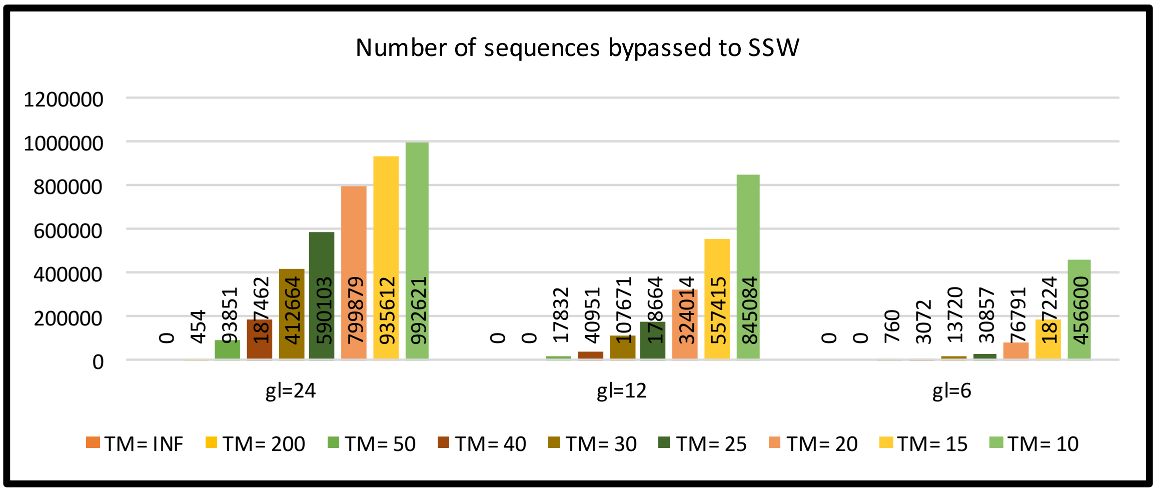

Figure 40: Number of sequences bypassed to SSW by TM (TS has not been applied) when processing DSL dataset.

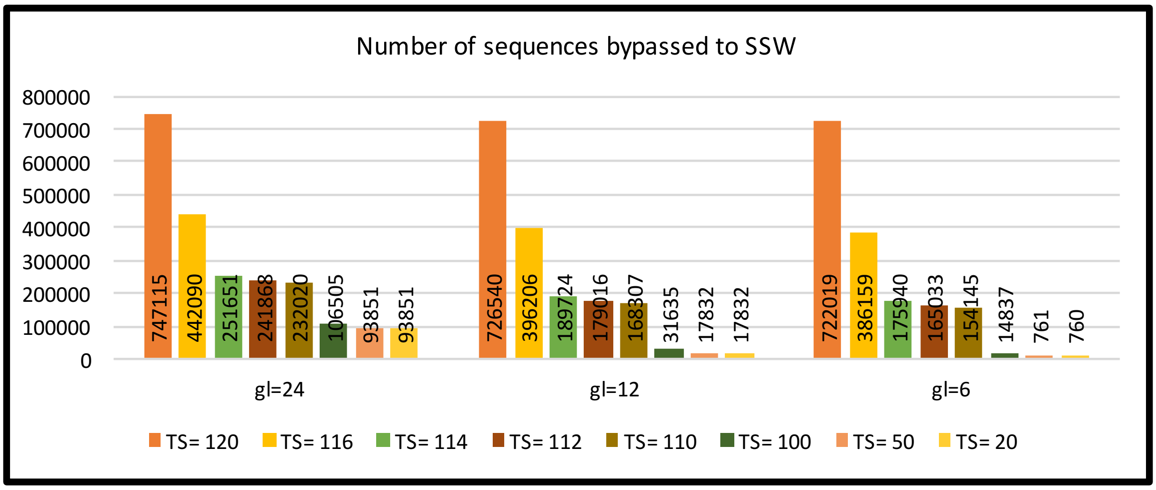

Figure 41: Total number of sequences bypassed to SSW by TM and TS when processing DSL dataset.

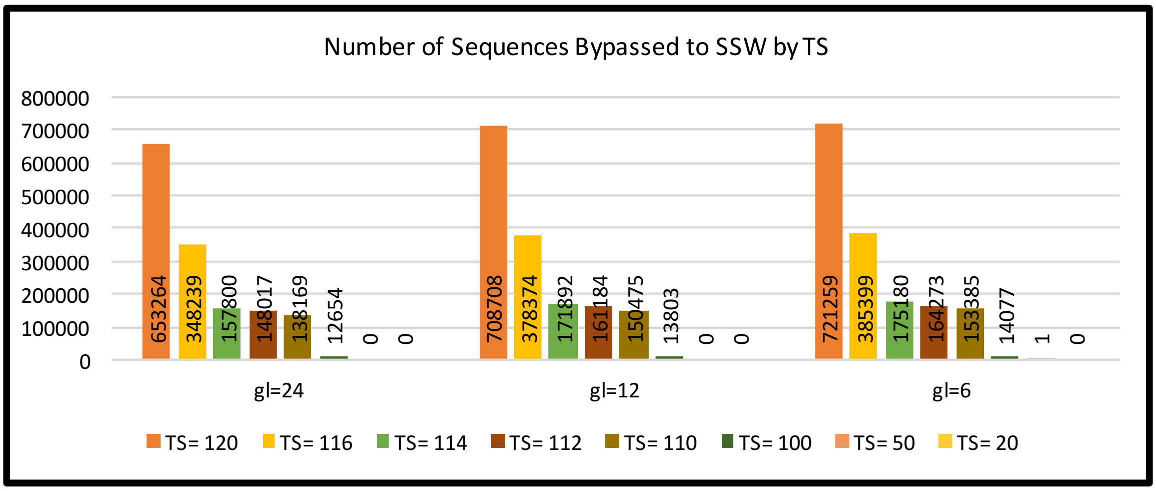

Figure 42: Total number of sequences bypassed to SSW by TS when processing DSL dataset.

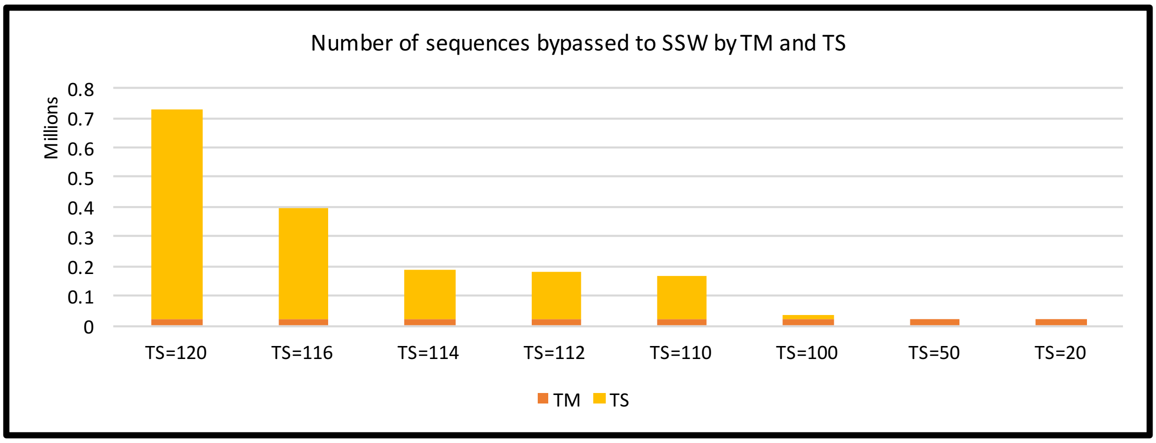

Figure 43: Proportion of input sequence pairs bypassed to SSW by TM and TS when processing DSL dataset.

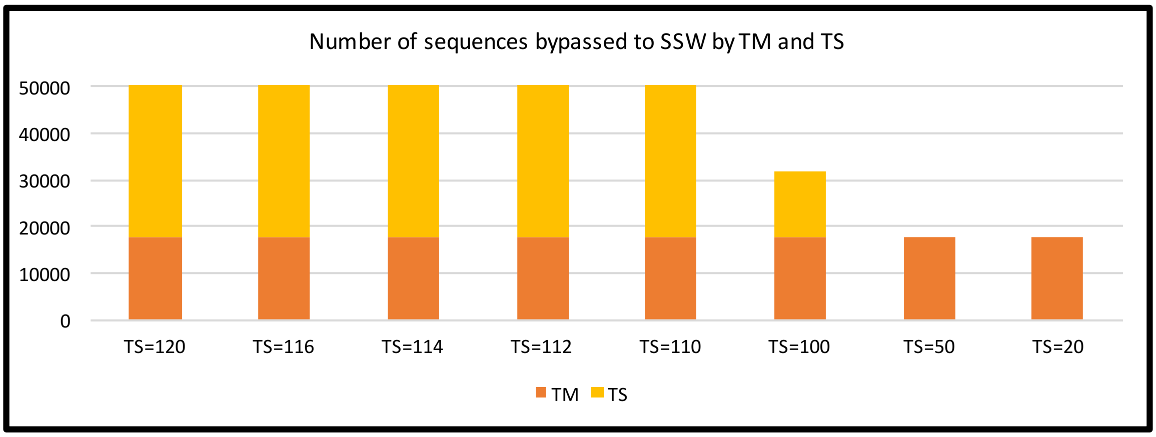

Figure 44: Proportion of input sequence pairs bypassed to SSW by TM and TS (scaled) when processing DSL dataset.

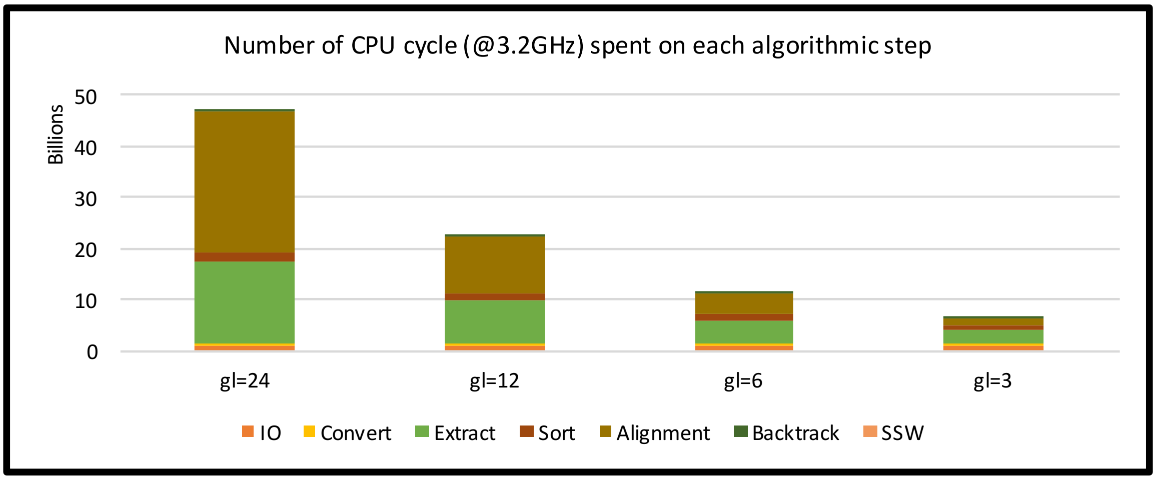

Figure 45: Cycle accurate execution time (DSL dataset) of differing algorithmic steps of *MEM-Align* when *gl* varies (in CPU cycle).

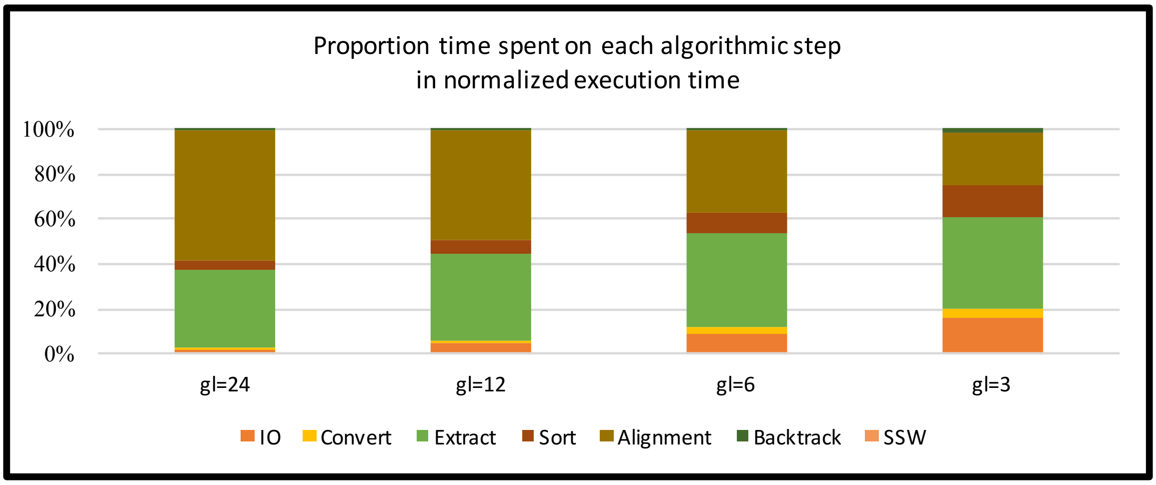

Figure 46: normalised cycle accurate execution time (DSL dataset) of differing algorithmic steps of *MEM-Align* when *gl* varies.

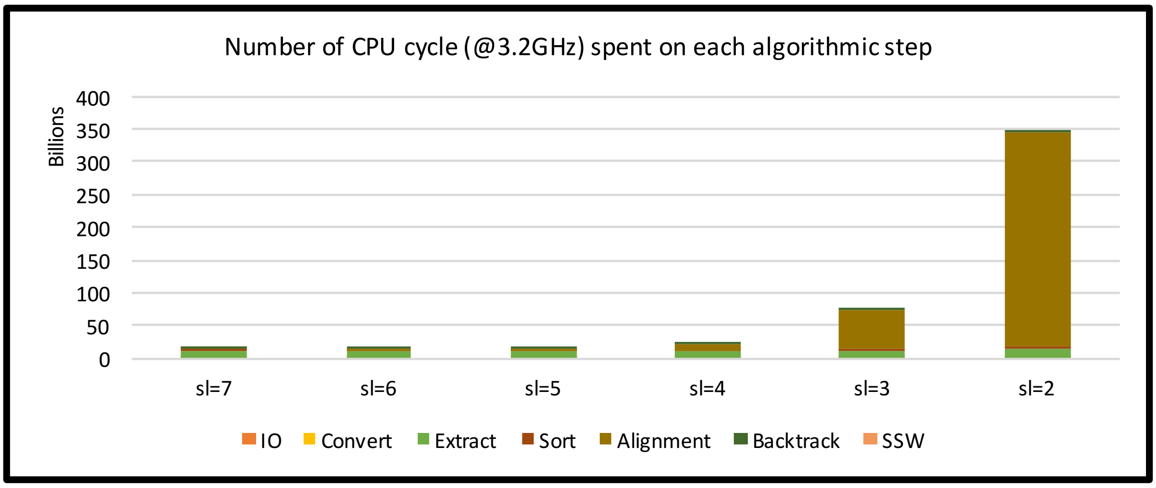

Figure 47: Cycle accurate execution time (DSL dataset) of differing algorithmic steps of *MEM-Align* when *sl* varies (in CPU cycle).

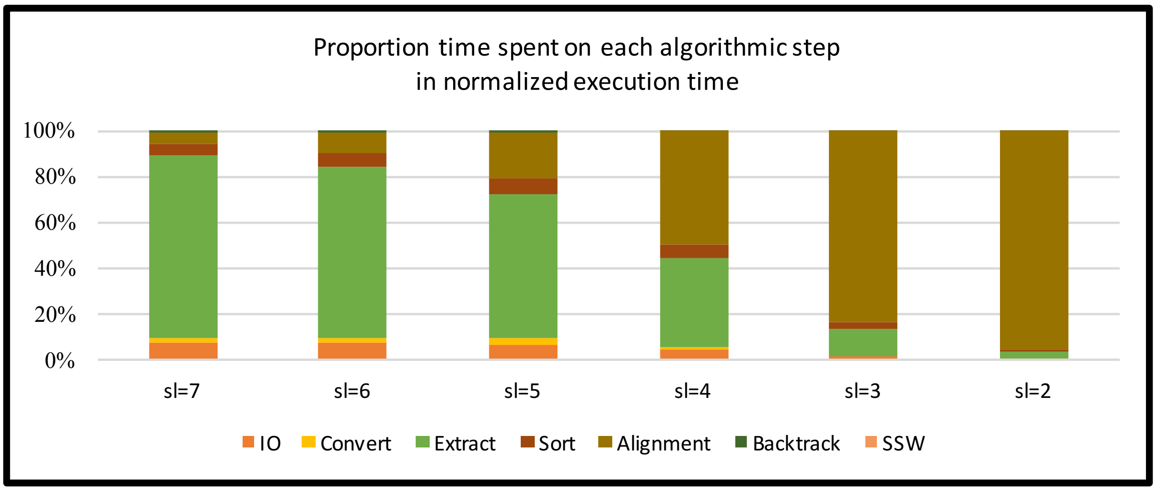

Figure 48: normalised cycle accurate execution time (DSL dataset) of differing algorithmic steps of *MEM-Align* when *sl* varies.

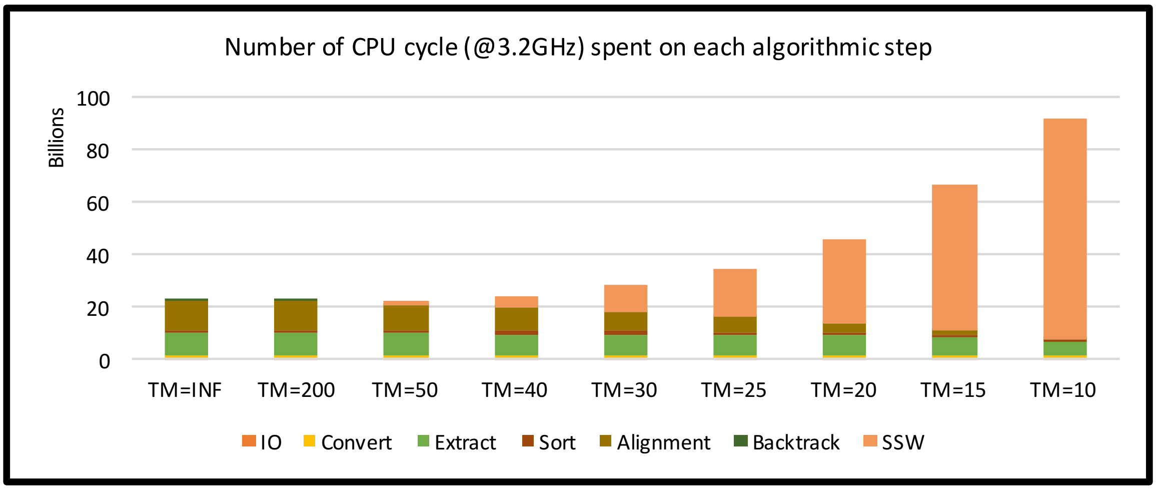

Figure 49: Cycle accurate execution time (DSL dataset) of differing algorithmic steps of *MEM-Align* when TM varies (in CPU cycle).

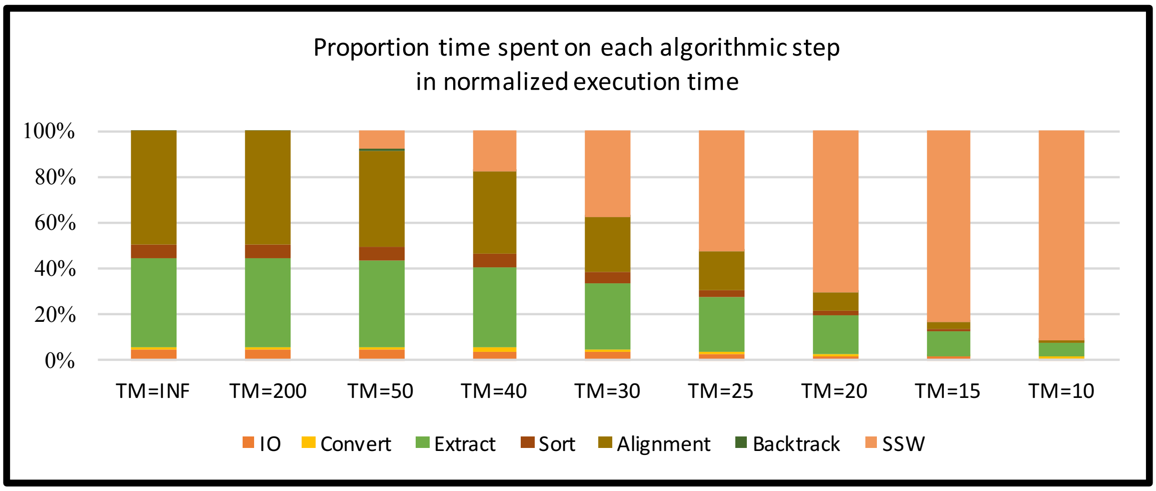

Figure 50: normalised cycle accurate execution time (DSL dataset) of differing algorithmic steps of *MEM-Align* when TM varies.

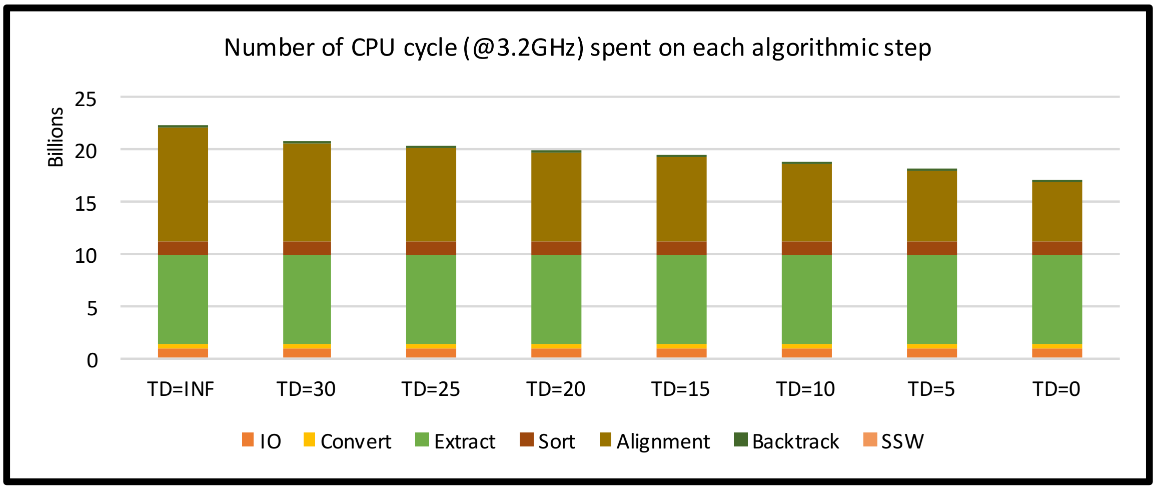

Figure 51: Cycle accurate execution time (DSL dataset) of differing algorithmic steps of *MEM-Align* when TD varies (in CPU cycle).

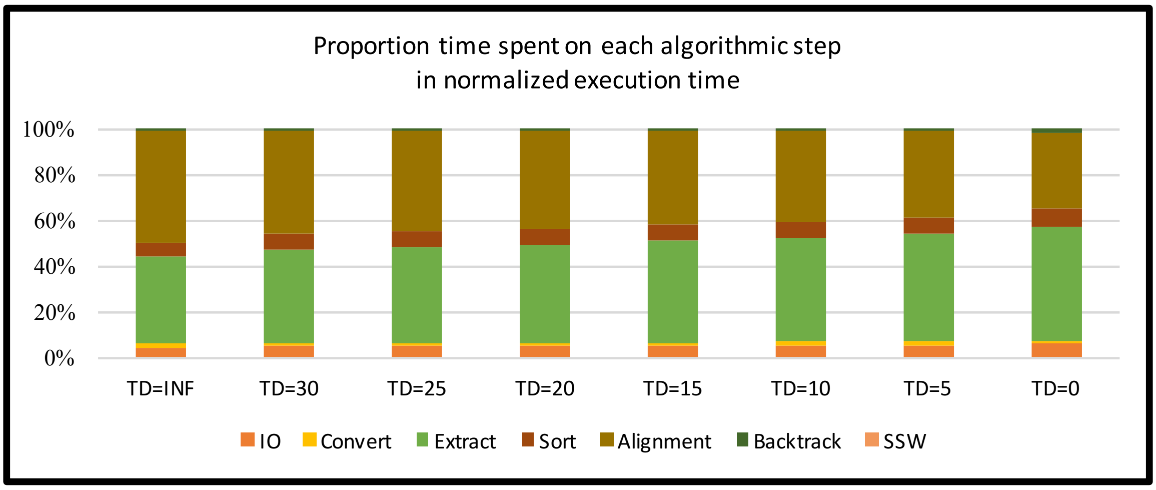

Figure 52: normalised cycle accurate execution time (DSL dataset) of differing algorithmic steps of *MEM-Align* when TD varies.

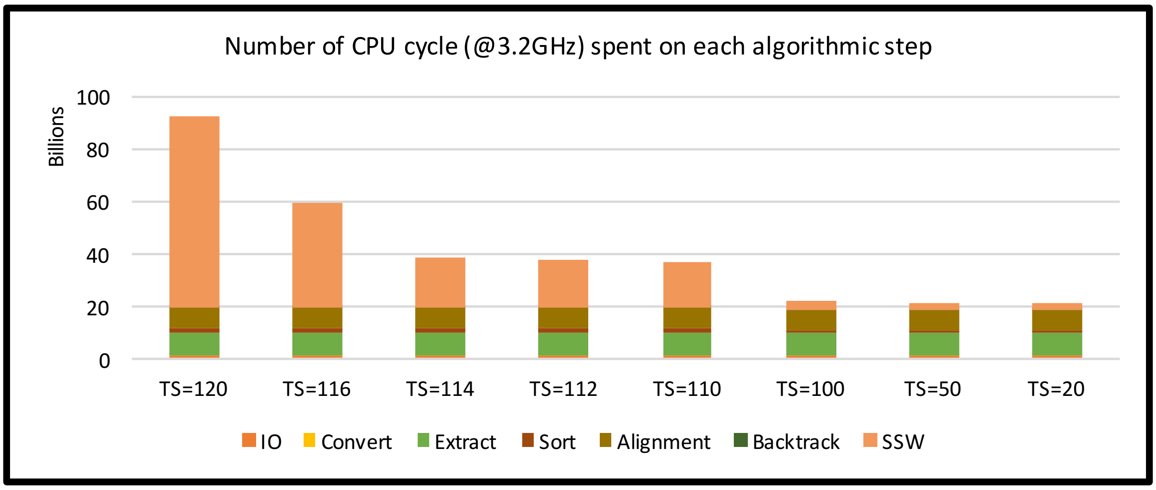

Figure 53: Cycle accurate execution time (DSL dataset) of differing algorithmic steps of *MEM-Align* when TS varies (in CPU cycle).

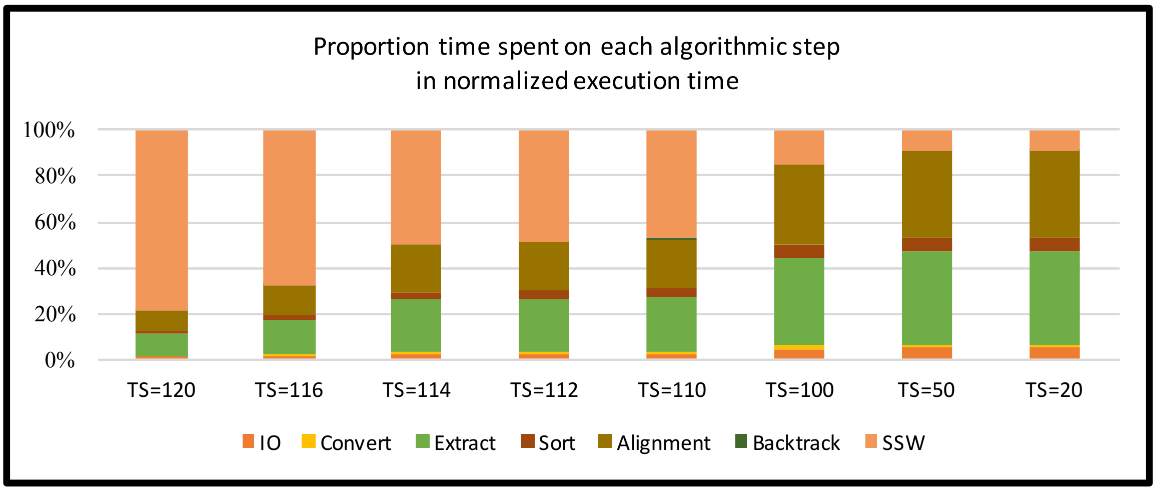

Figure 54: normalised cycle accurate execution time (DSL dataset) of differing algorithmic steps of *MEM-Align* when TS varies.

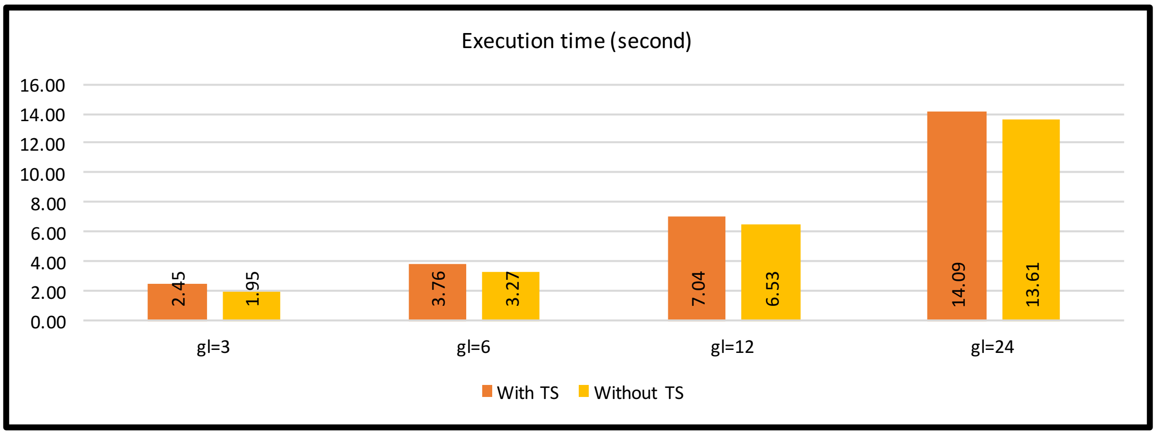

Figure 55: Execution time (DSL dataset) for differing gl with and without TS.

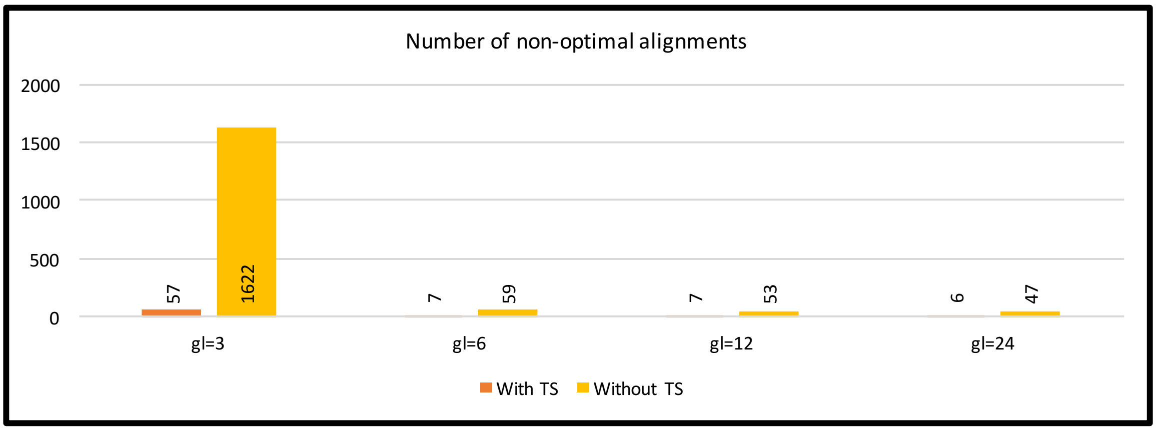

Figure 56: Number of suboptimal alignments (DSL dataset) for differing gl with and without TS.

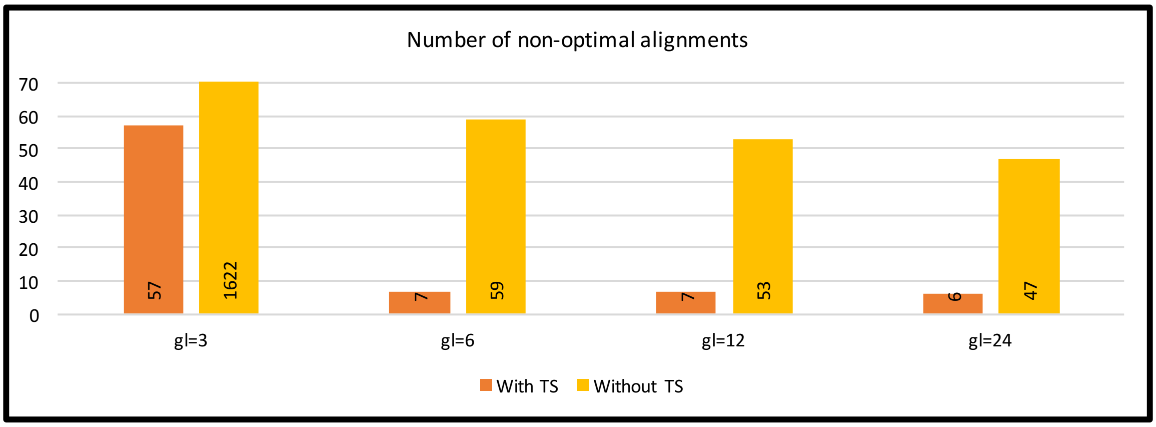

Figure 57: Number of suboptimal alignments (DSL dataset) for differing gl with and without TS (scaled).

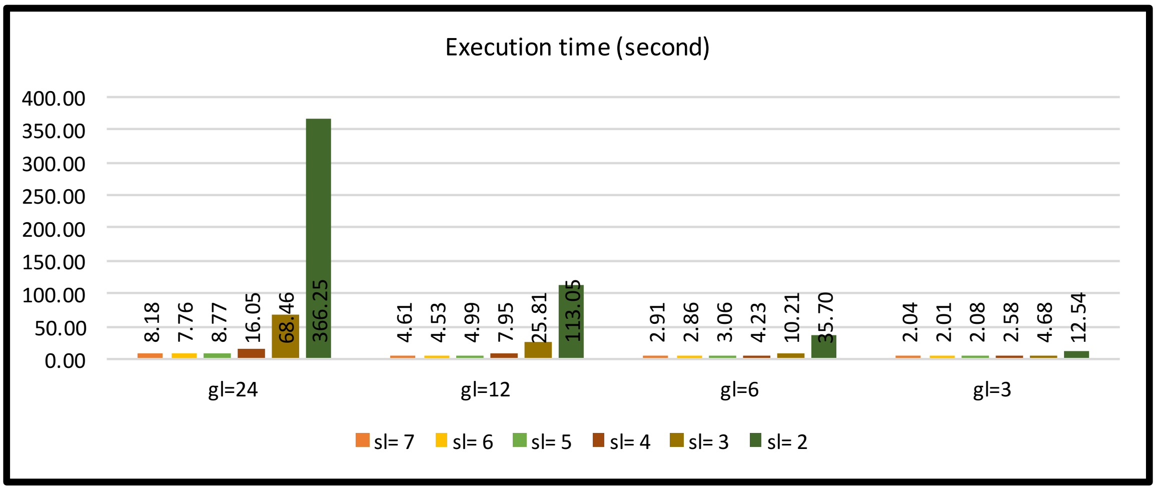

Figure 58: Execution times for DSH dataset when *gl* and *sl* varies.

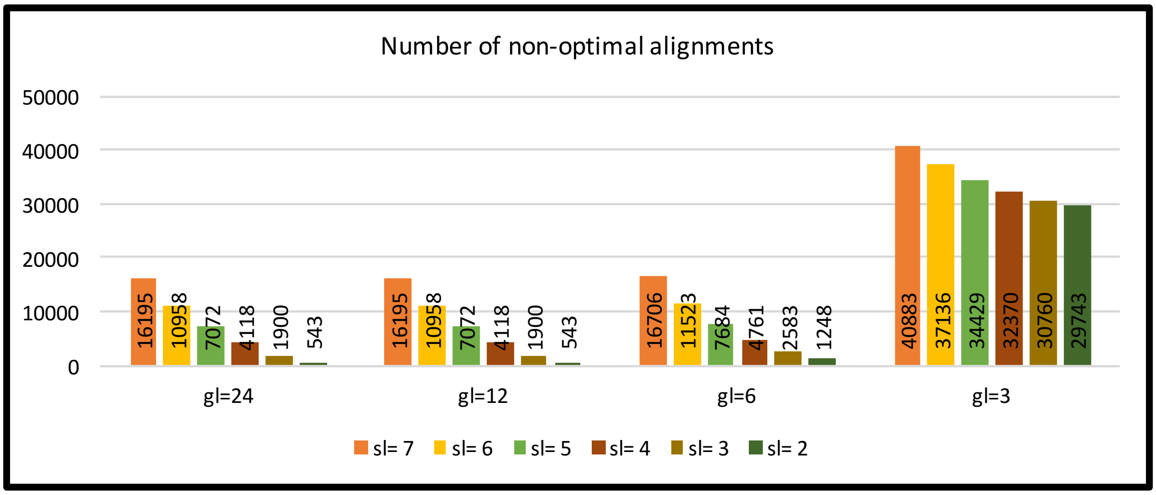

Figure 59: Number of suboptimal alignments for DSH dataset when *gl* and *sl* varies.

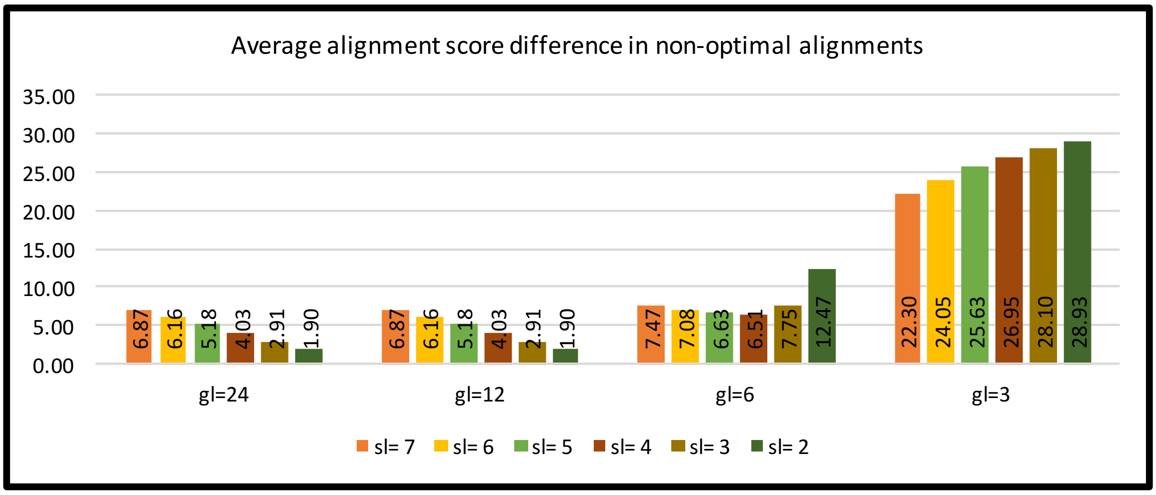

Figure 60: Average alignment score difference in suboptimal alignments for DSH dataset when *gl* and *sl* varies.

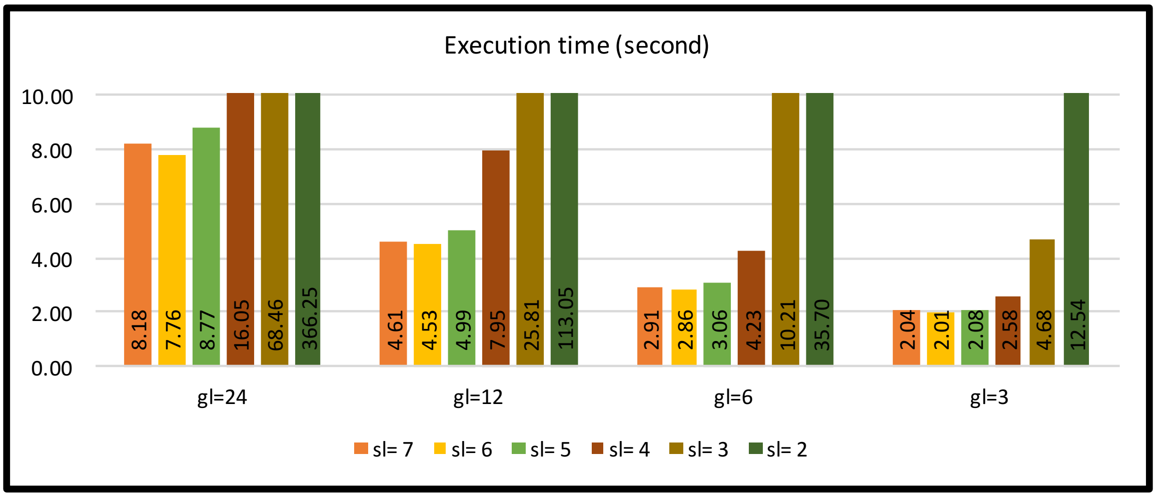

Figure 61: Execution times for DSH dataset when gl and sl varies (scaled).

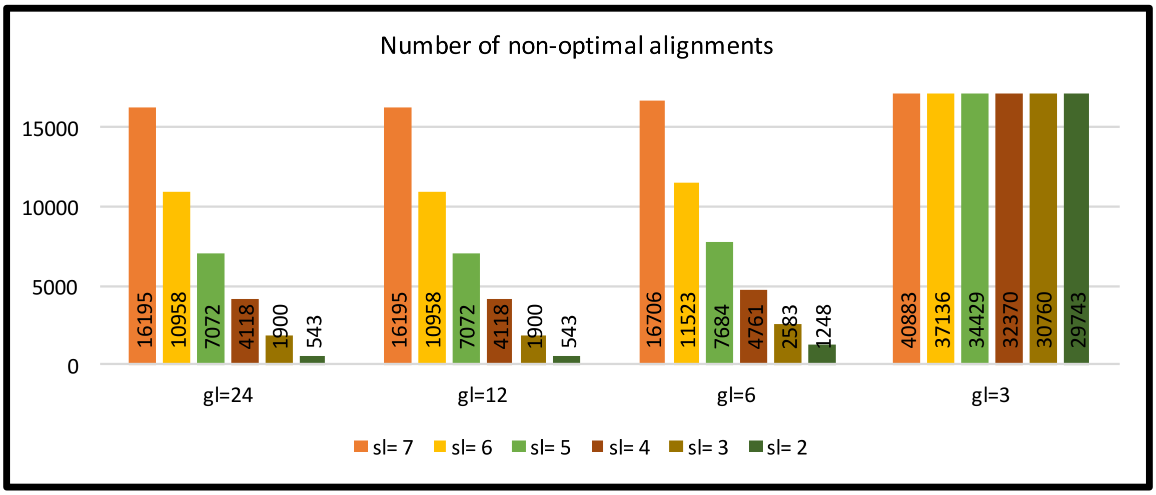

Figure 62: Number of suboptimal alignments for DSH dataset when gl and sl varies (scaled).

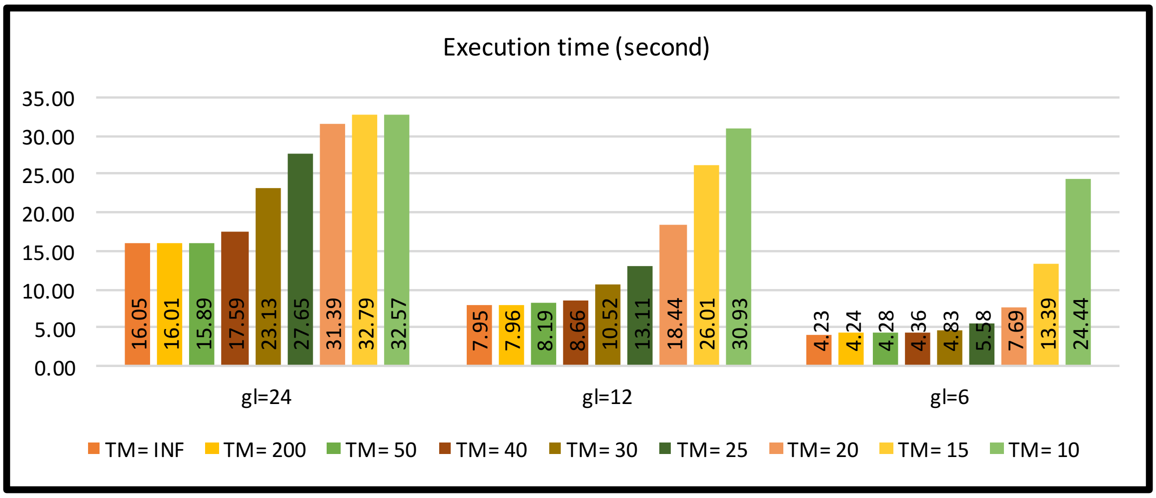

Figure 63: Execution times for DSH dataset when TM varies.

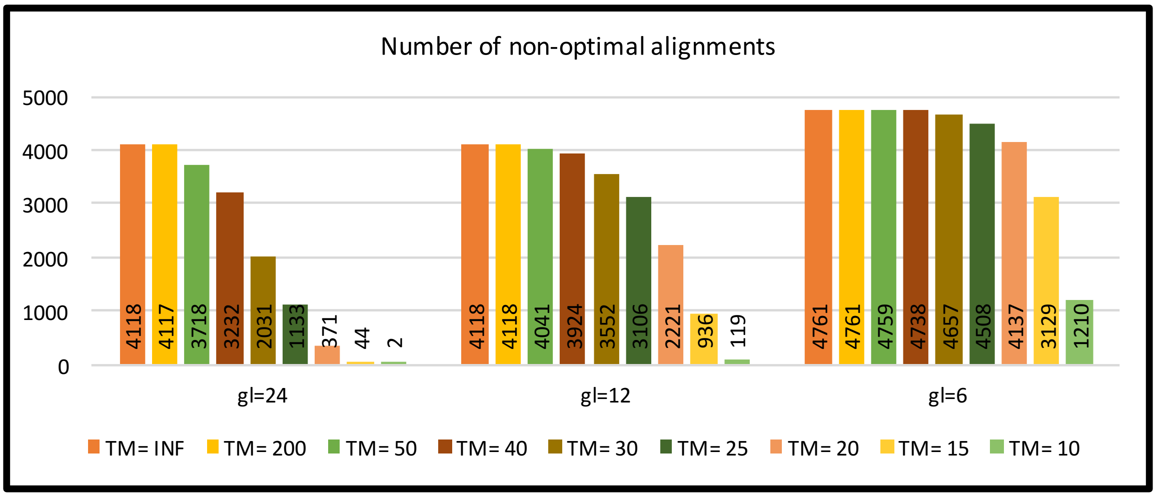

Figure 64: Number of suboptimal alignments for DSH dataset when TM varies.

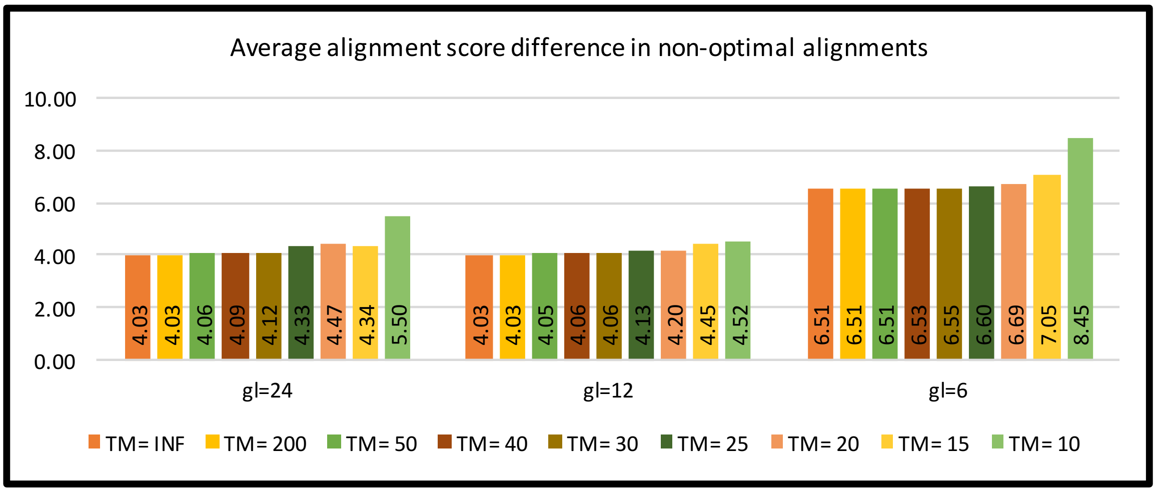

Figure 65: Average alignment score difference in suboptimal alignments for DSH dataset when TM varies.

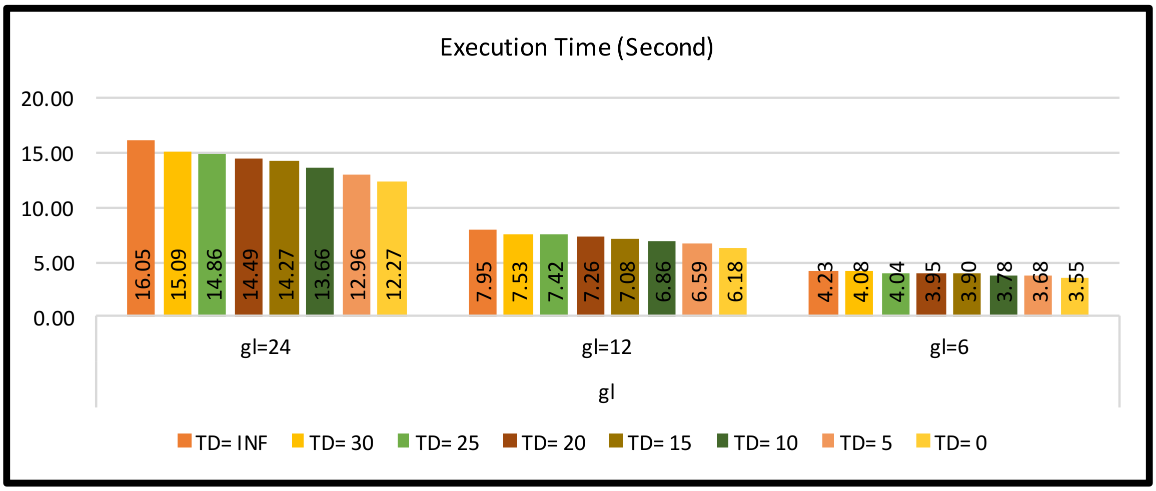

Figure 66: Execution times for DSH dataset when TD varies.

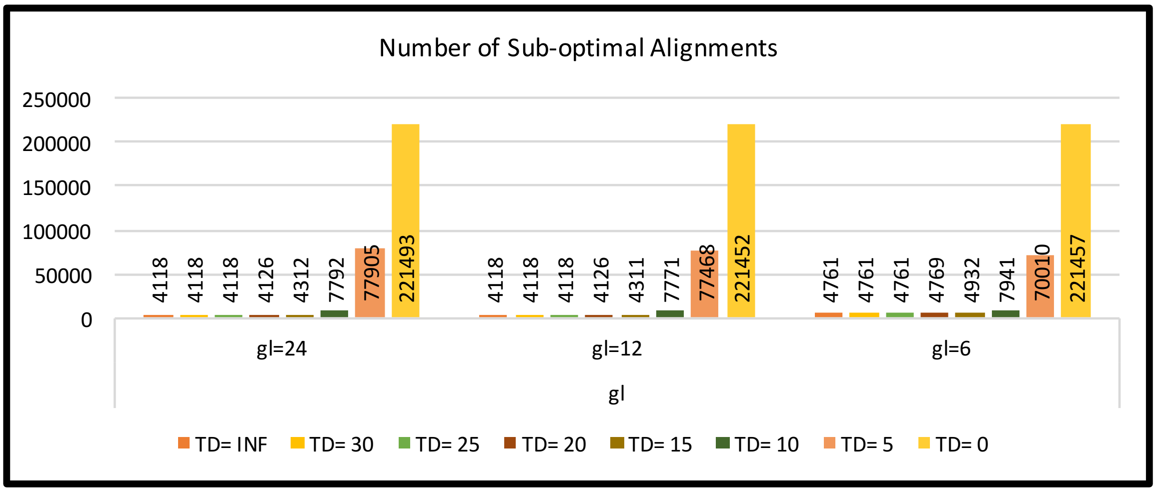

Figure 67: Number of suboptimal alignments for DSH dataset when TD varies.

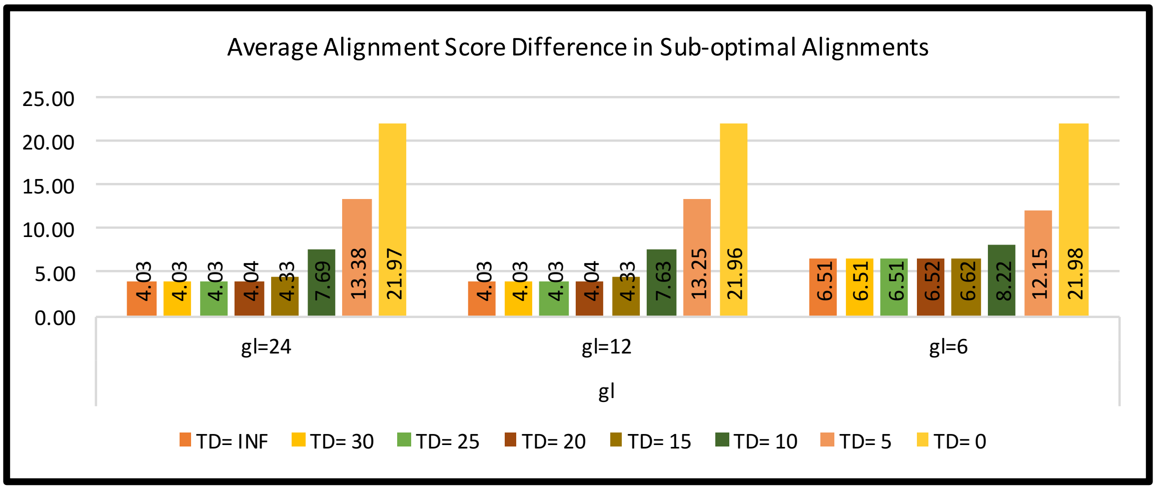

Figure 68: Average alignment score difference in suboptimal alignments for DSH dataset when TM varies.

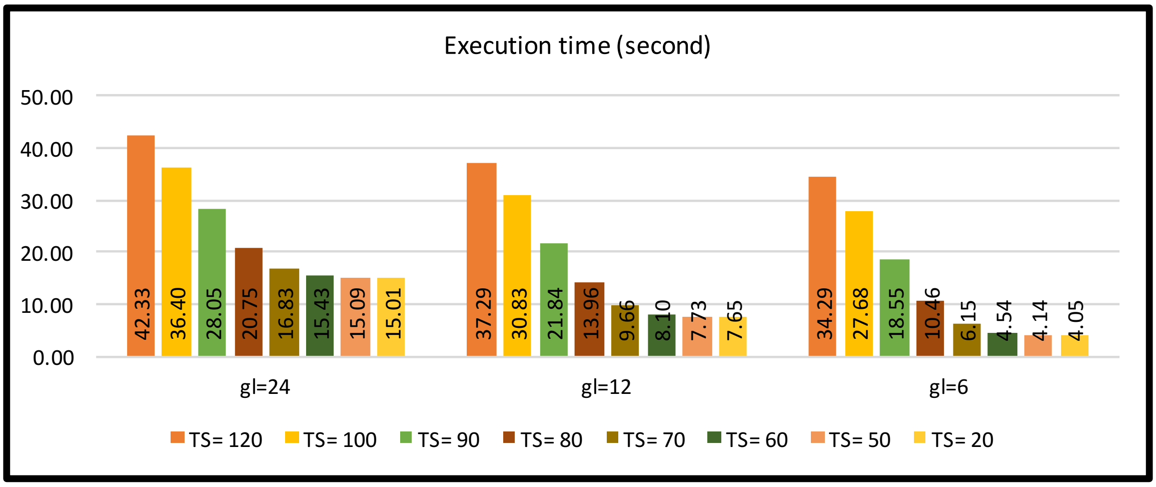

Figure 69: Execution times for DSH dataset when TS varies.

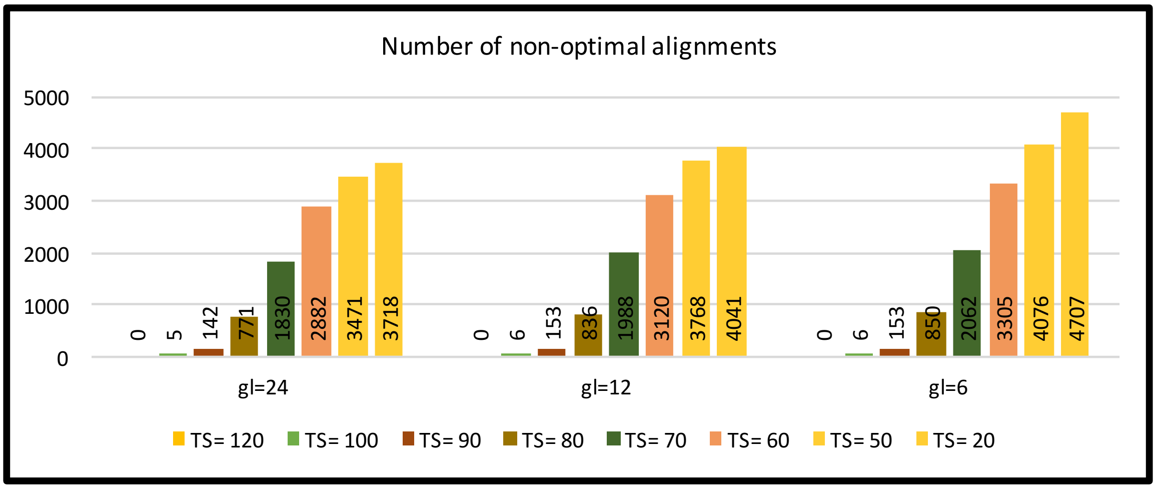

Figure 70: Number of suboptimal alignments for DSH dataset when TS varies.

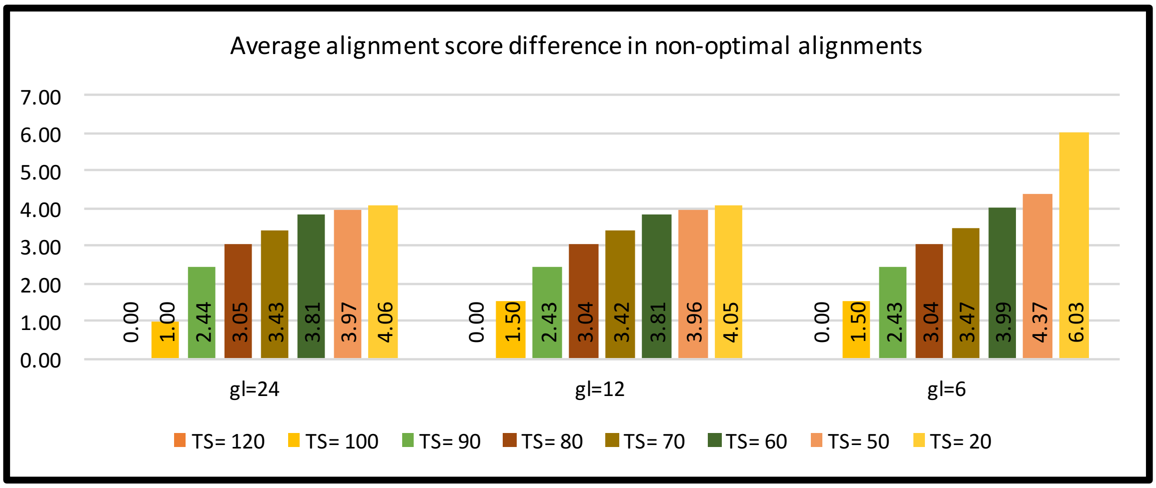

Figure 71: Average alignment score difference in suboptimal alignments for DSH dataset when TS varies.

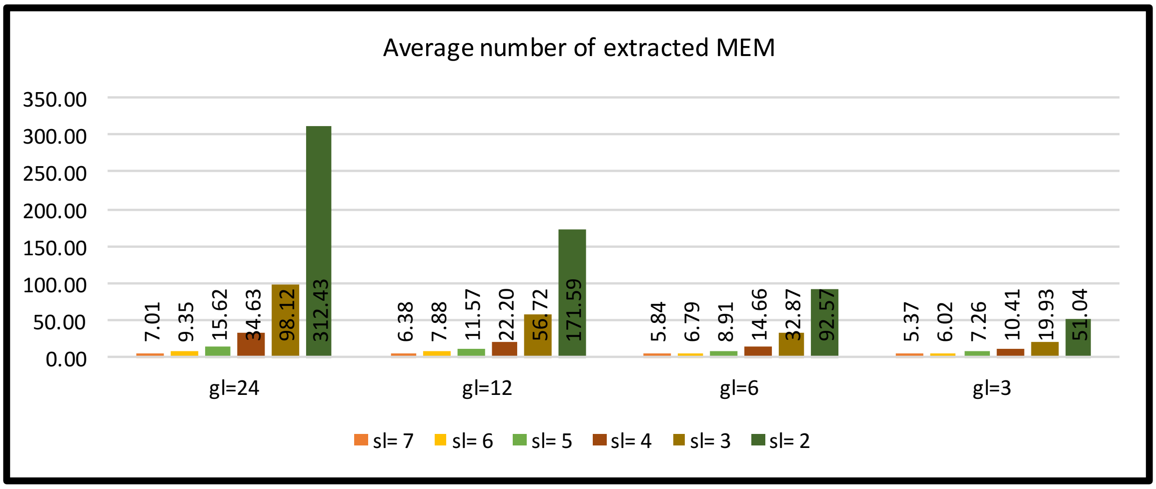

Figure 72: Average number of extracted MEM for DSH dataset when gl and sl varies.

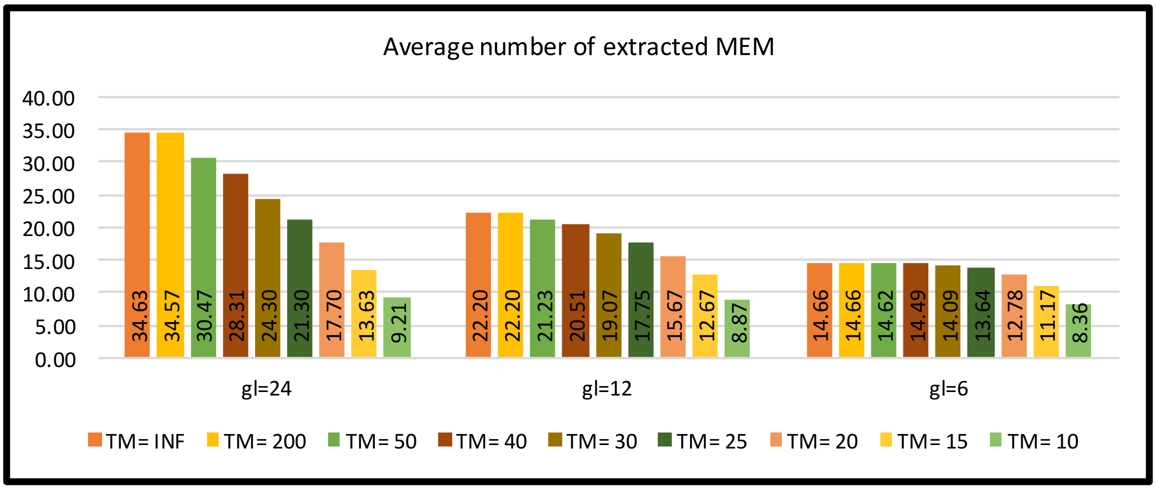

Figure 73: Average number of extracted MEM for DSH dataset when TM varies.

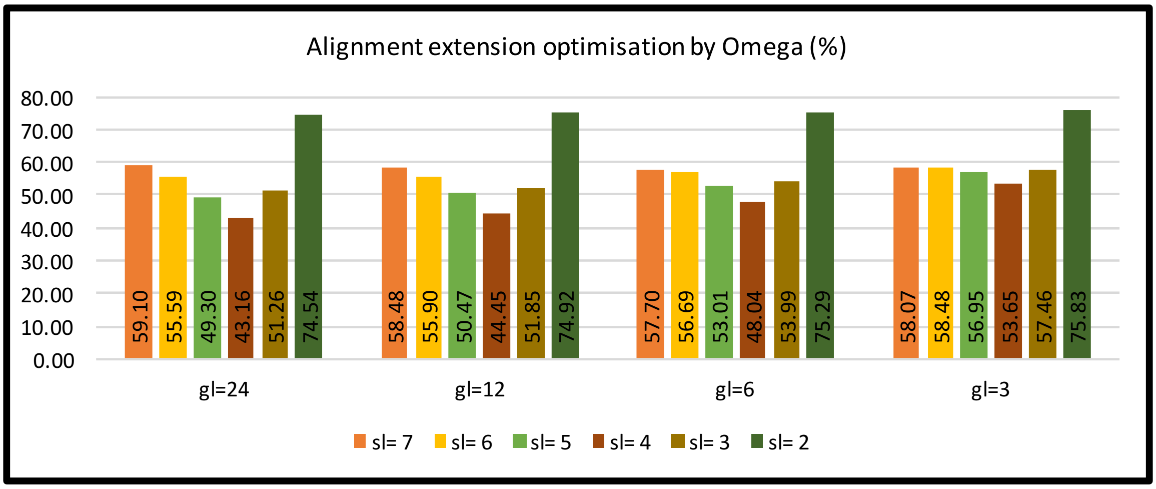

Figure 74: Proportion of alignment extension which are optimised (avoided) by the set Ω when processing DSH dataset.

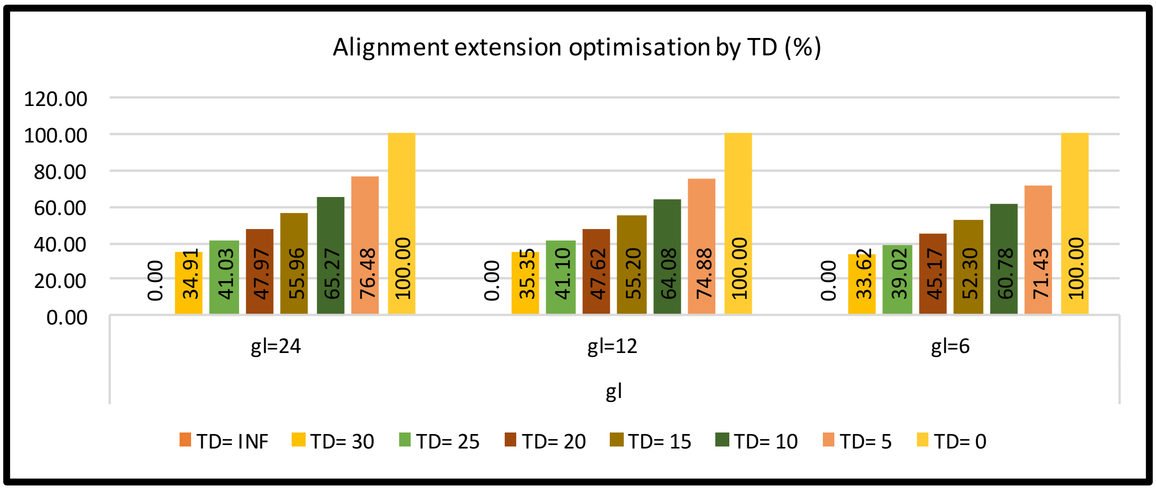

Figure 75: Proportion of alignment extension which are optimised (avoided) by TD after applying the set Ω when processing DSH dataset.

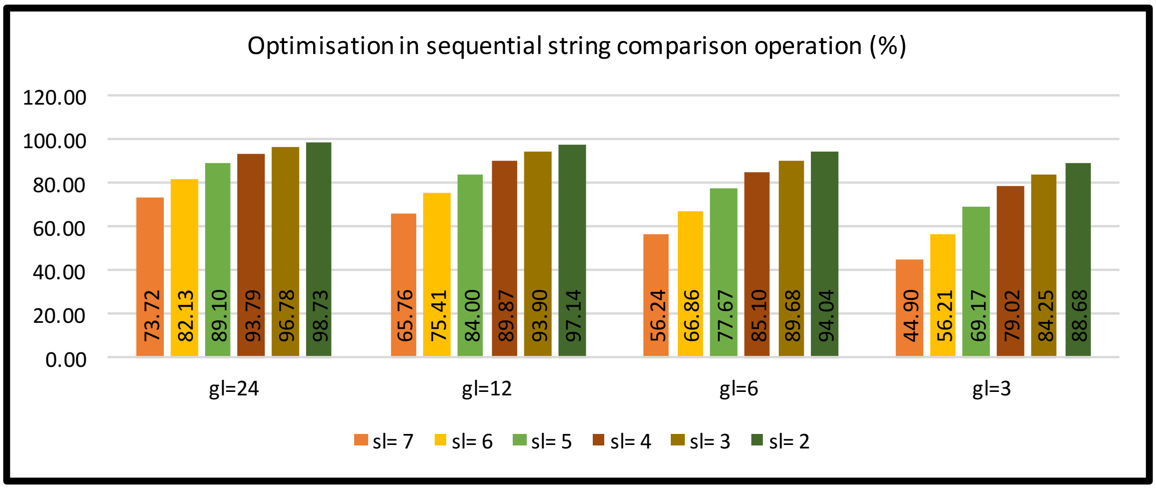

Figure 76: Proportion of sequential string compare operation which are optimised (avoided) when processing DSH dataset.

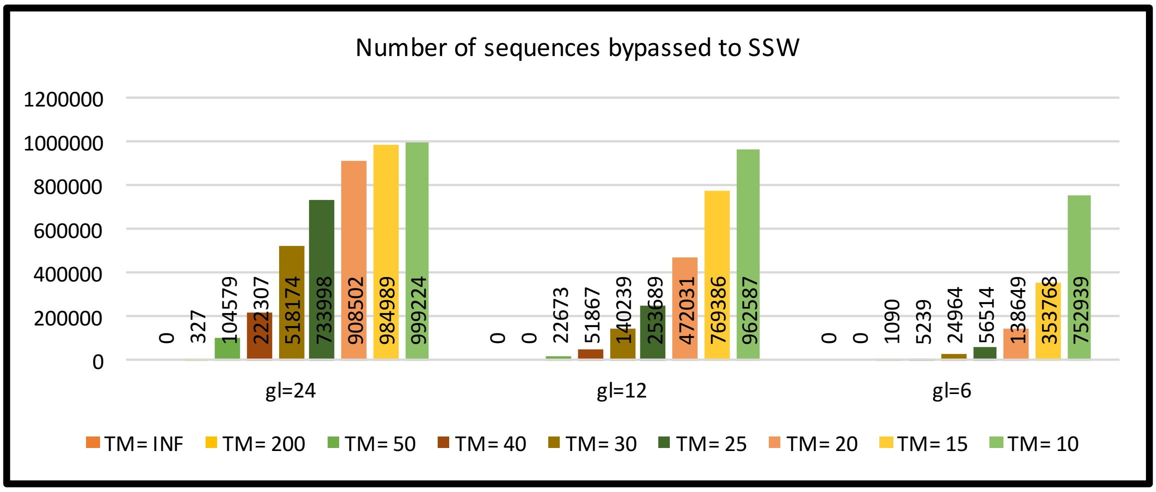

Figure 77: Number of sequences bypassed to SSW by TM (TS has not been applied) when processing DSH dataset.

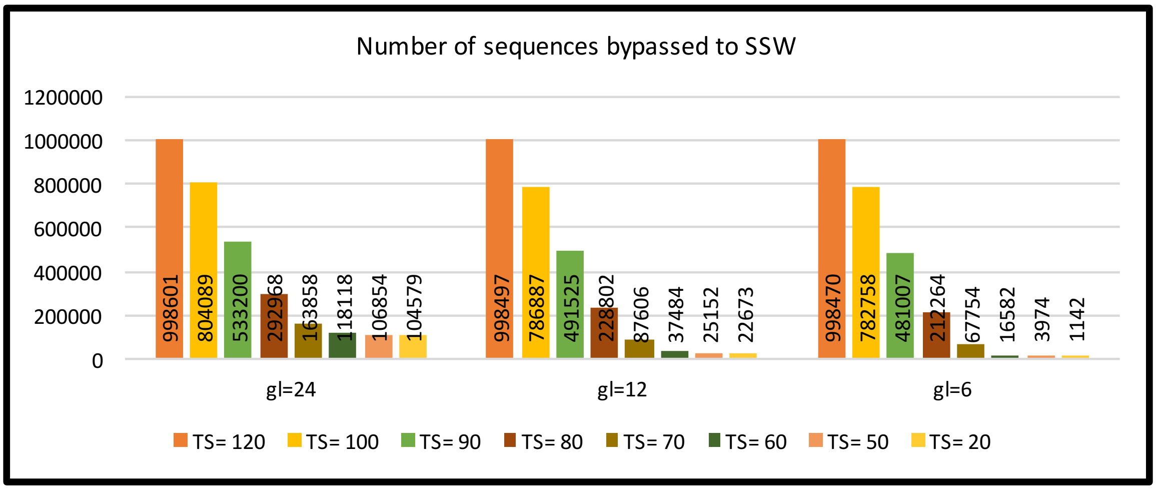

Figure 78: Total number of sequences bypassed to SSW by TM and TS when processing DSH dataset.

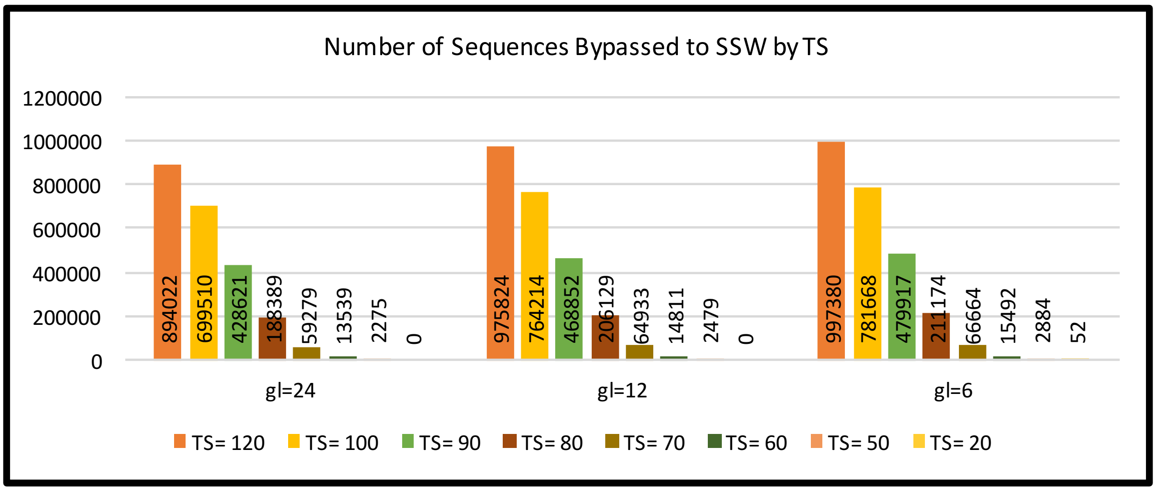

Figure 79: Total number of sequences bypassed to SSW by TS when processing DSH dataset.

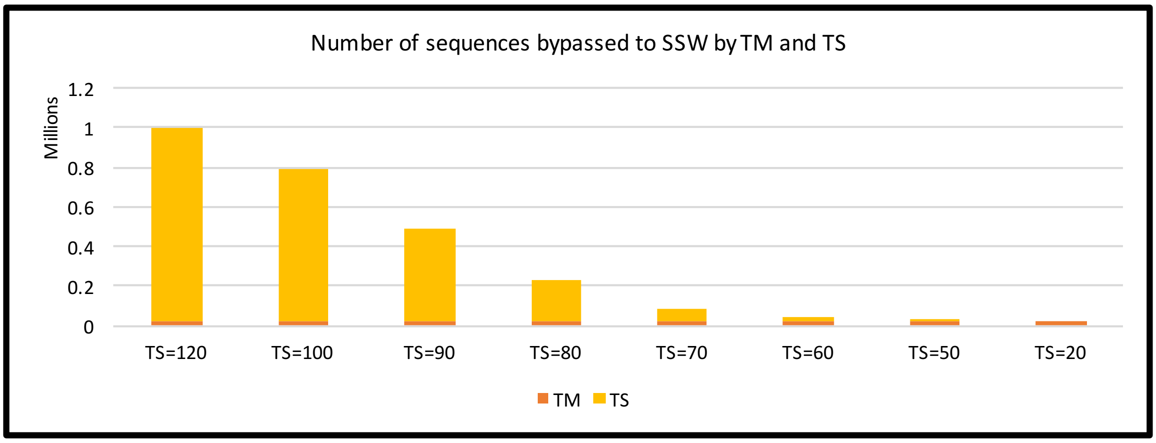

Figure 80: Proportion of input sequence pairs bypassed to SSW by TM and TS when processing DSH dataset.

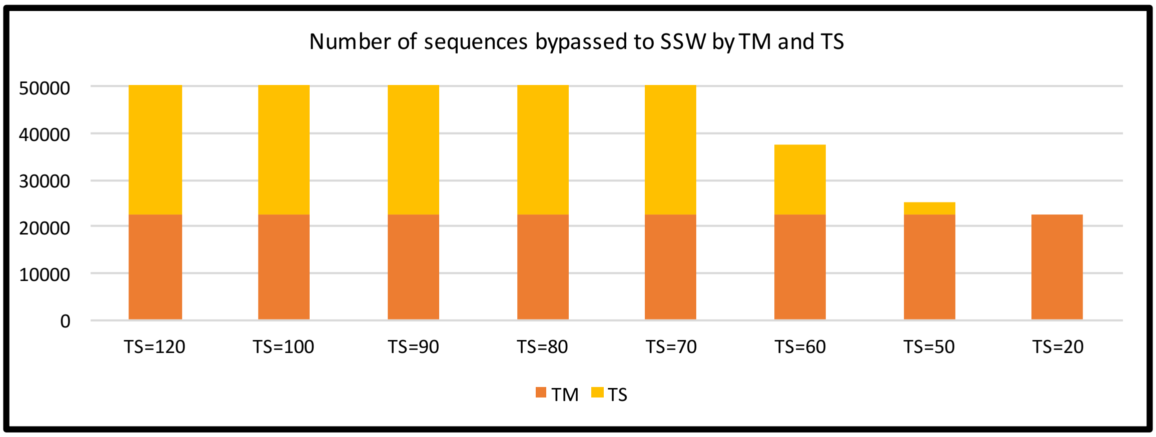

Figure 81: Proportion of input sequence pairs bypassed to SSW by TM and TS (scaled) when processing DSH dataset.

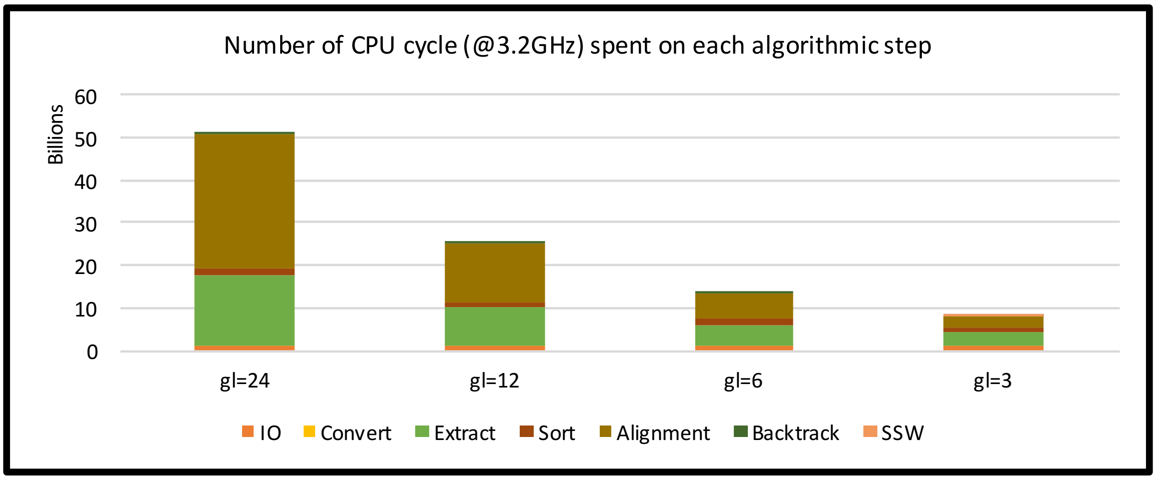

Figure 82: Cycle accurate execution time (DSH dataset) of differing algorithmic steps of *MEM-Align* when *gl* varies (in CPU cycle).

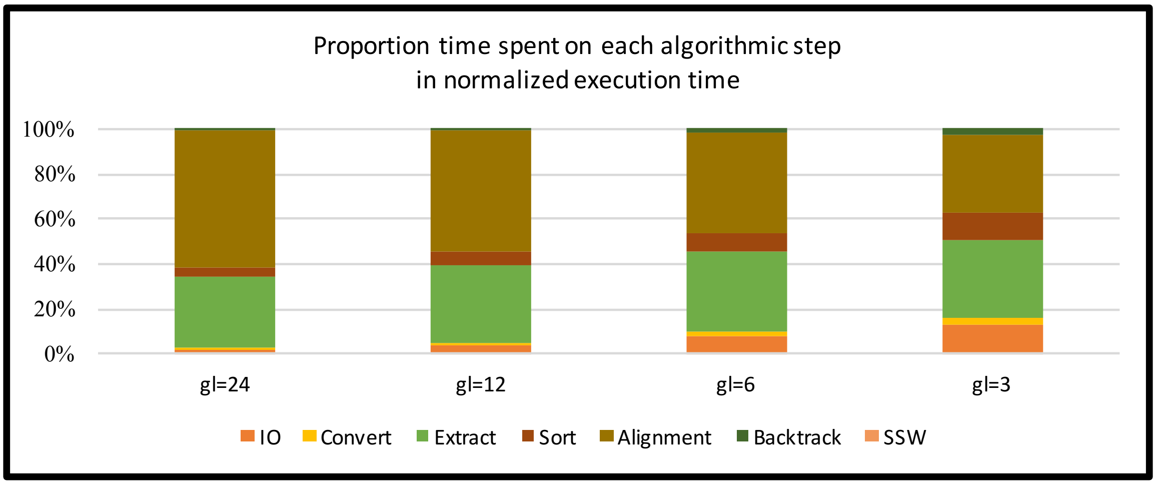

Figure 83: normalised cycle accurate execution time (DSH dataset) of differing algorithmic steps of *MEM-Align* when *gl* varies.

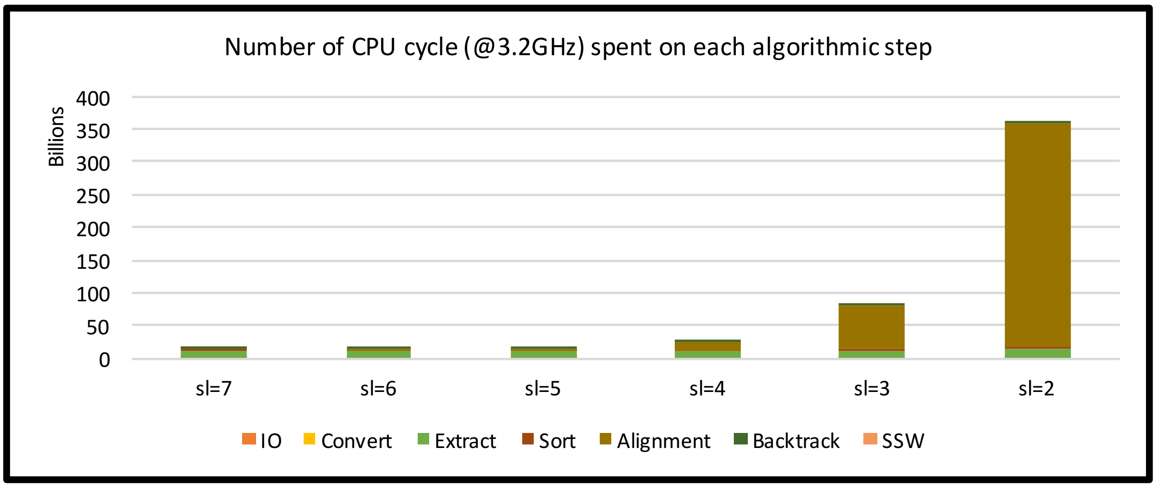

Figure 84: Cycle accurate execution time (DSH dataset) of differing algorithmic steps of *MEM-Align* when *sl* varies (in CPU cycle).

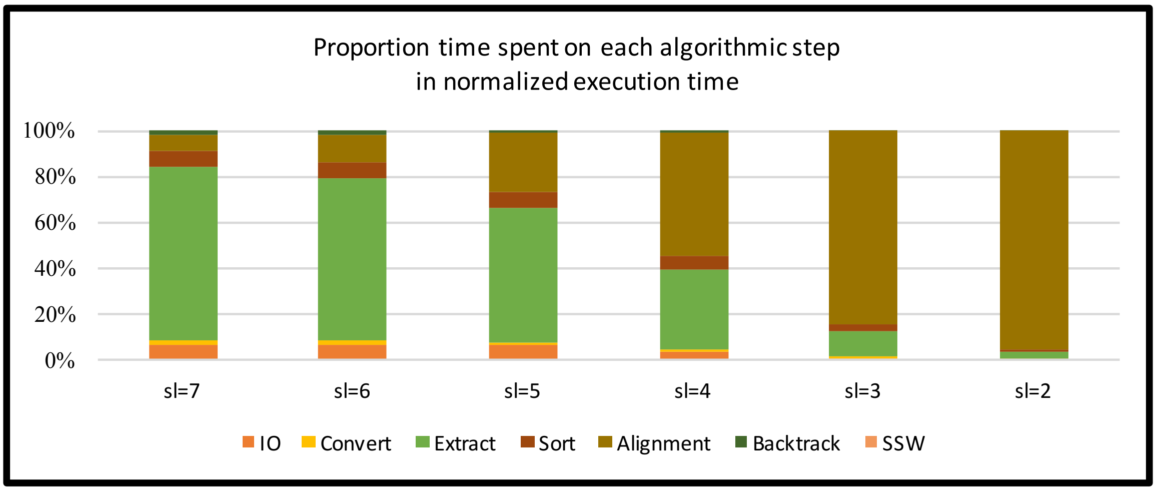

Figure 85: normalised cycle accurate execution time (DSH dataset) of differing algorithmic steps of *MEM-Align* when *sl* varies.

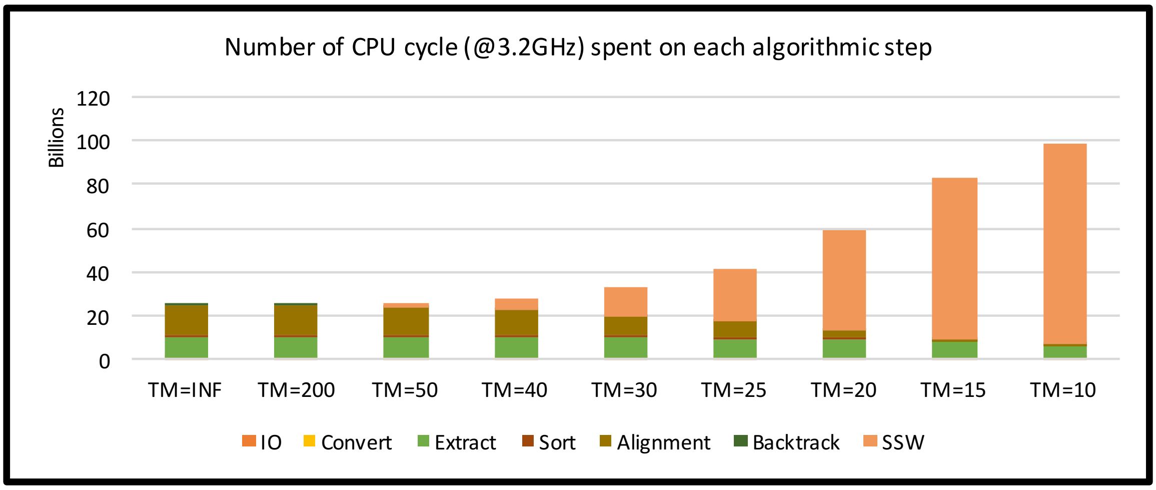

Figure 86: Cycle accurate execution time (DSH dataset) of differing algorithmic steps of *MEM-Align* when TM varies (in CPU cycle).

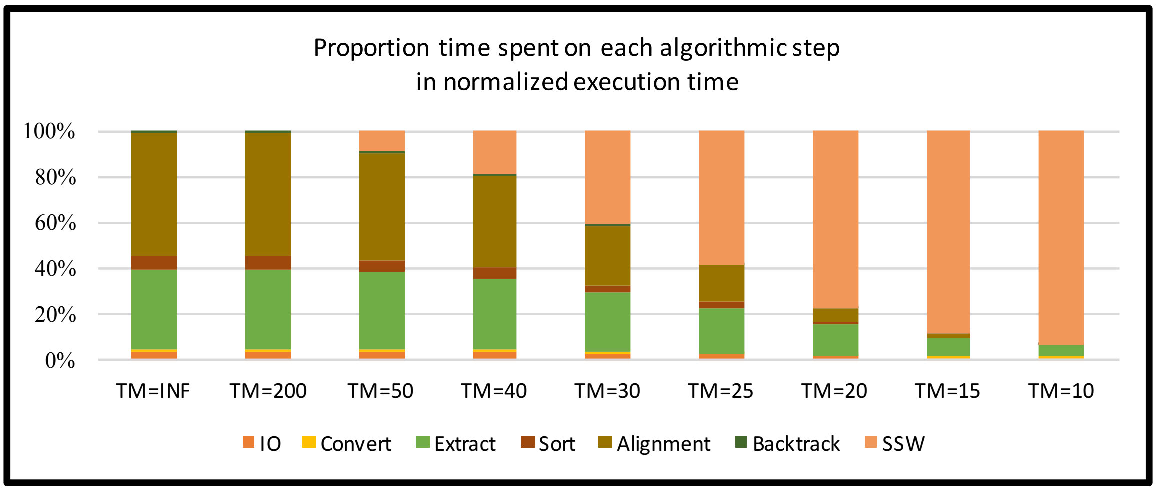

Figure 87: normalised cycle accurate execution time (DSH dataset) of differing algorithmic steps of *MEM-Align* when TM varies.

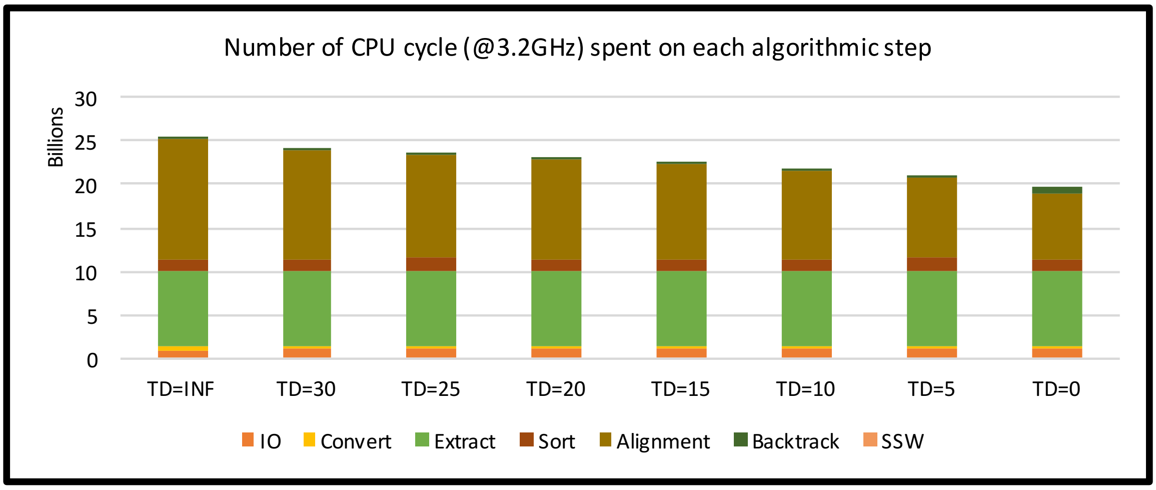

Figure 88: Cycle accurate execution time (DSH dataset) of differing algorithmic steps of *MEM-Align* when TD varies (in CPU cycle).

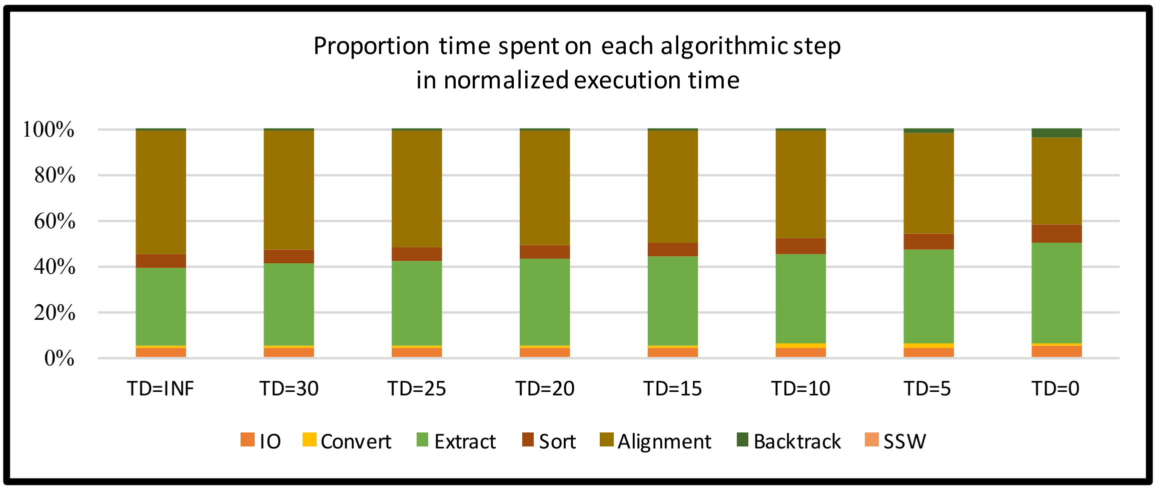

Figure 89: normalised cycle accurate execution time (DSH dataset) of differing algorithmic steps of *MEM-Align* when TD varies.

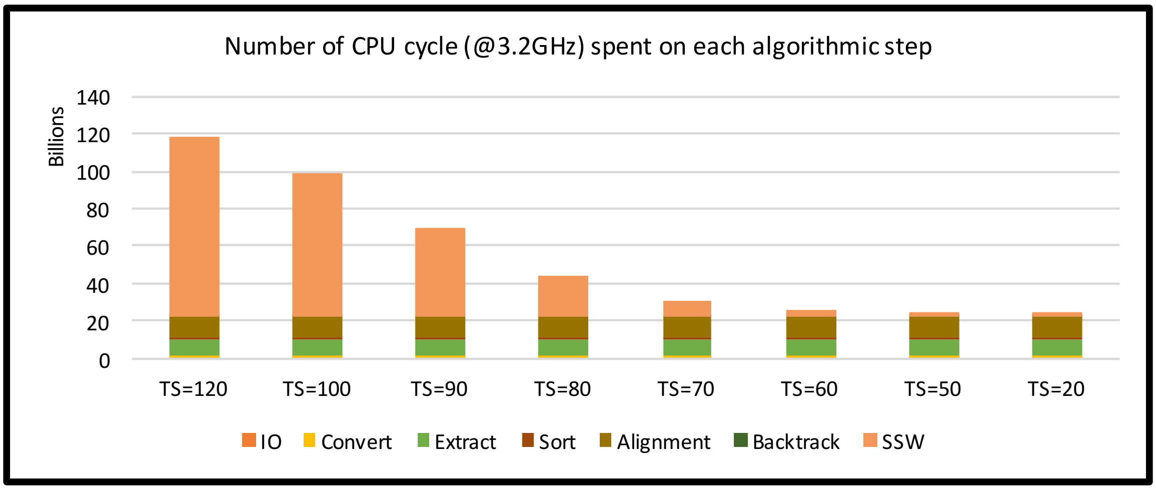

Figure 90: Cycle accurate execution time (DSH dataset) of differing algorithmic steps of *MEM-Align* when TS varies (in CPU cycle).

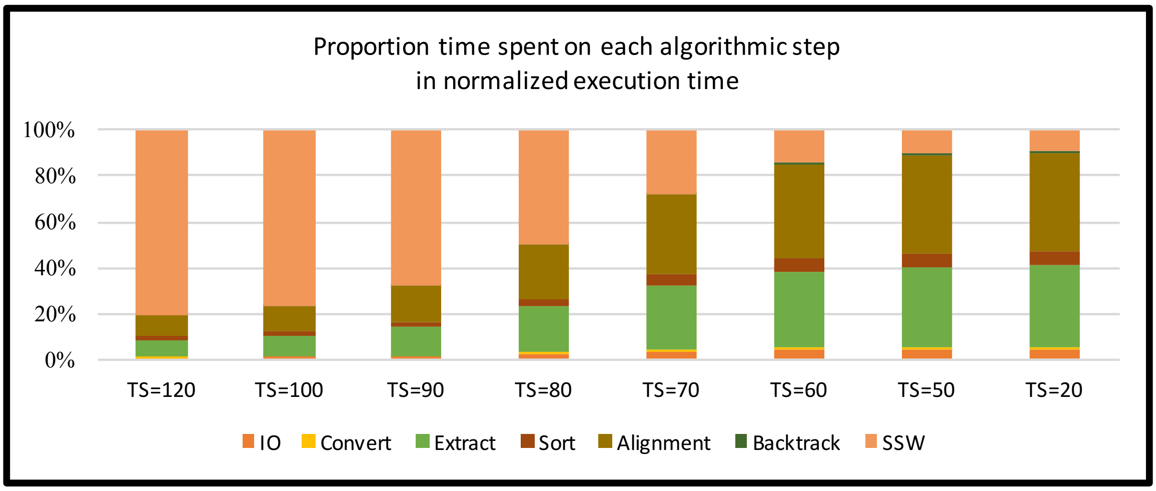

Figure 91: normalised cycle accurate execution time (DSH dataset) of differing algorithmic steps of *MEM-Align* when TS varies.

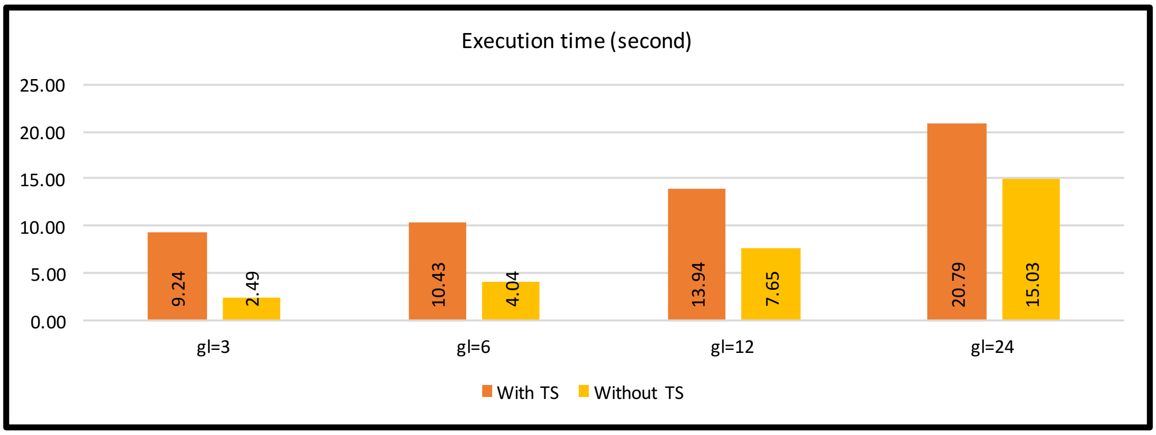

Figure 92: Execution time (DSH dataset) for differing gl with and without TS.

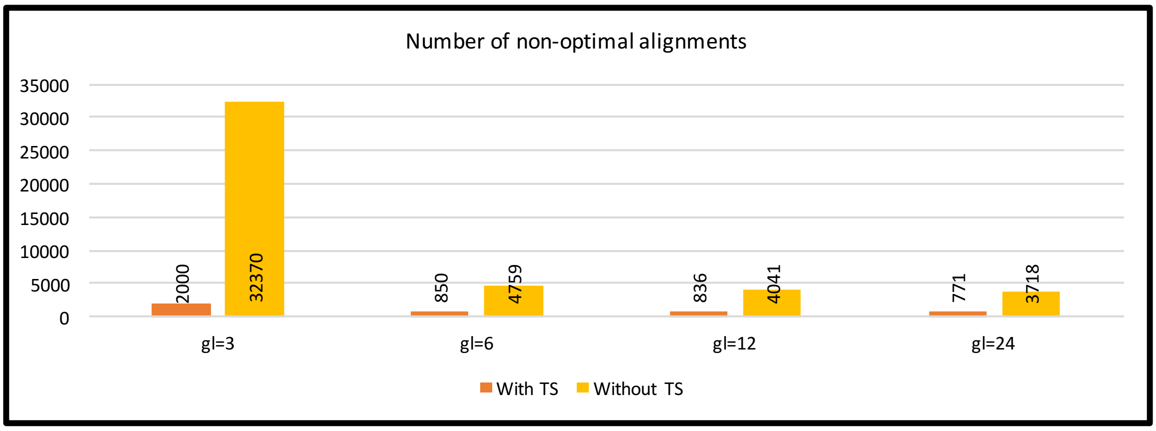

Figure 93: Number of suboptimal alignments (DSH dataset) for differing gl with and without TS.

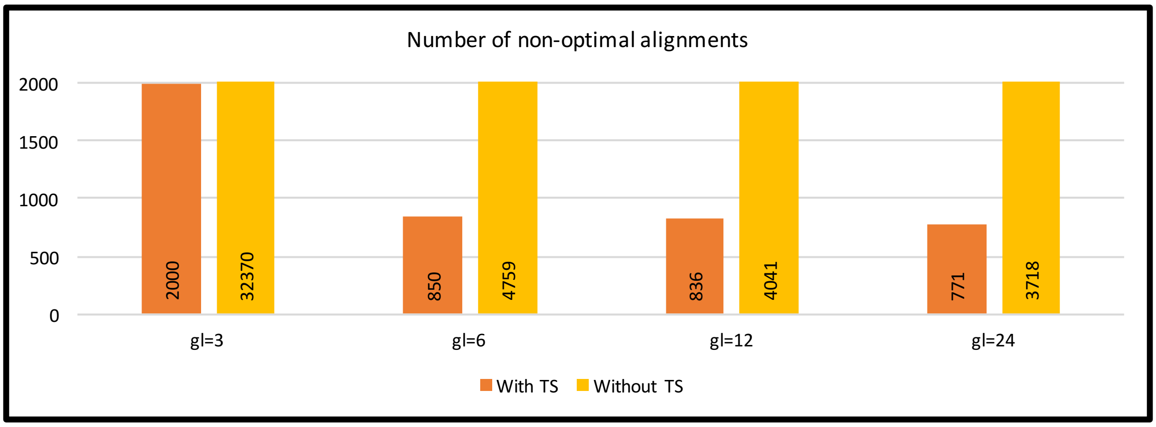

Figure 94: Number of suboptimal alignments (DSH dataset) for differing gl with and without TS (scaled).

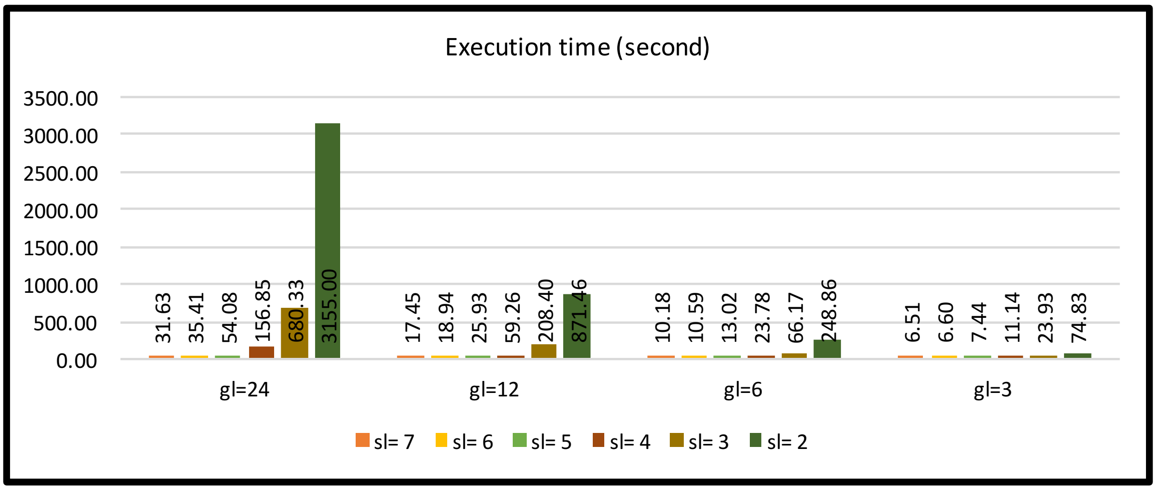

Figure 95: Execution times for DLL dataset when gl and sl varies.

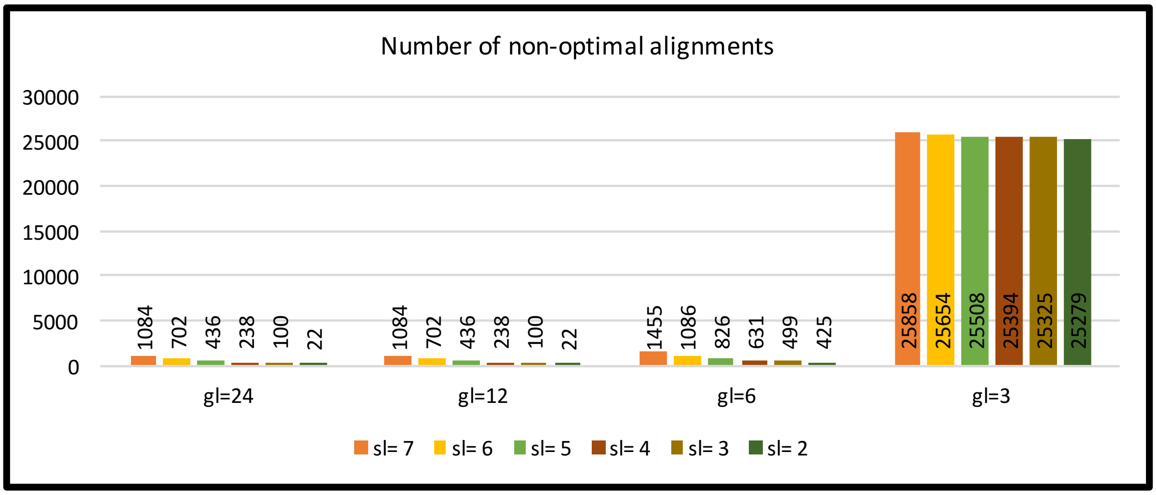

Figure 96: Number of suboptimal alignments for DLL dataset when gl and sl varies.

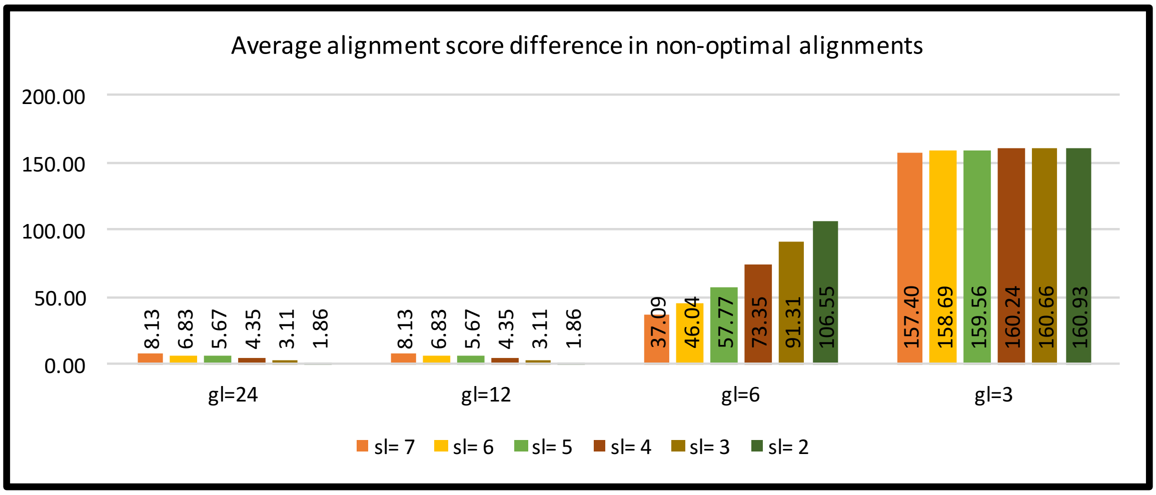

Figure 97: Average alignment score difference in suboptimal alignments for DLL dataset when gl and sl varies.

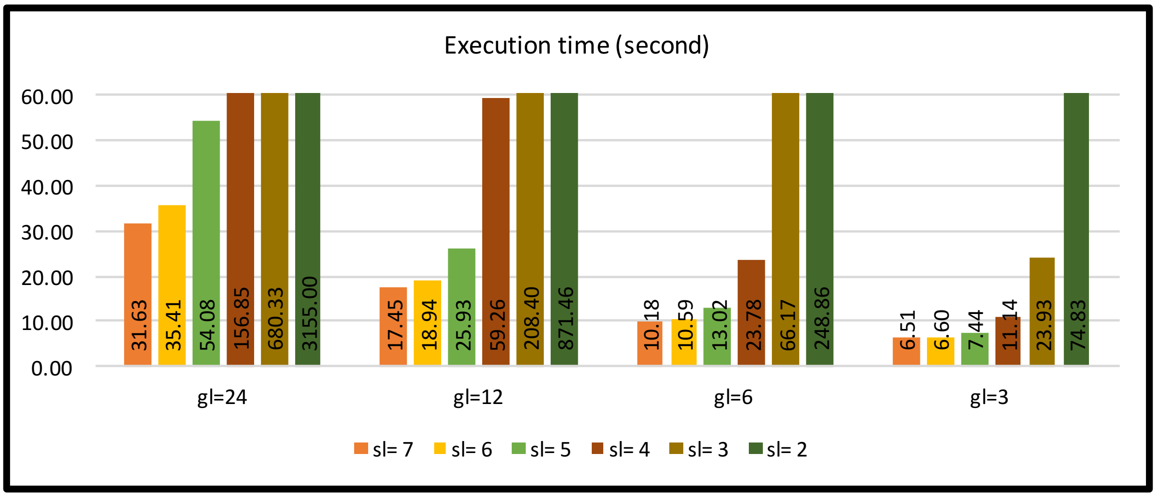

Figure 98: Execution times for DLL dataset when gl and sl varies (scaled).

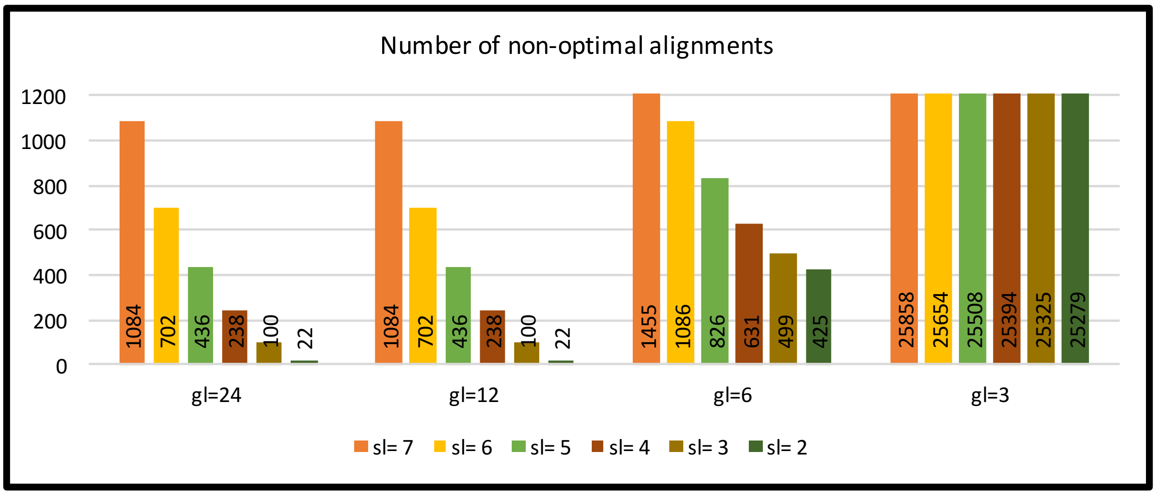

Figure 99: Number of suboptimal alignments for DLL dataset when gl and sl varies (scaled).

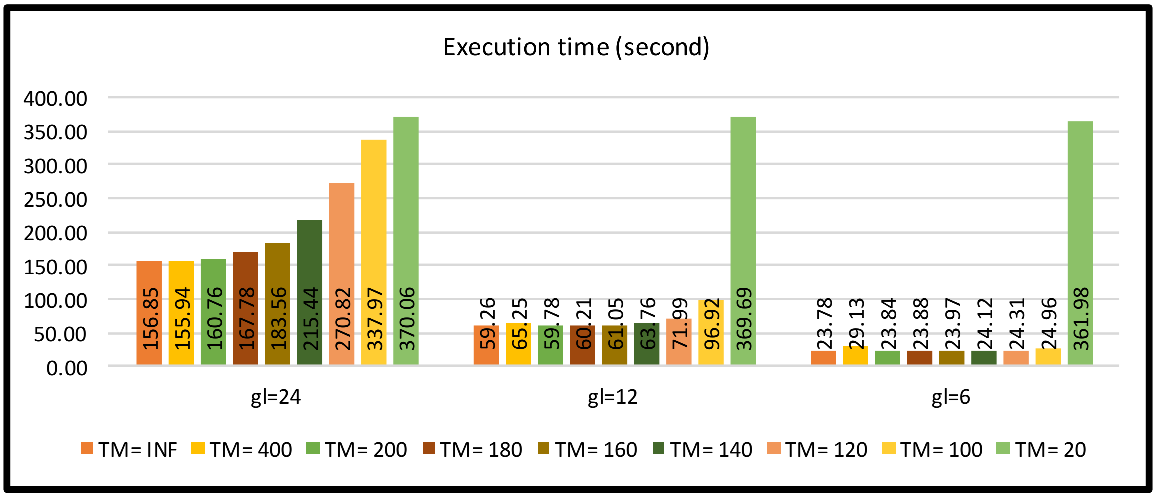

Figure 100: Execution times for DLL dataset when TM varies.

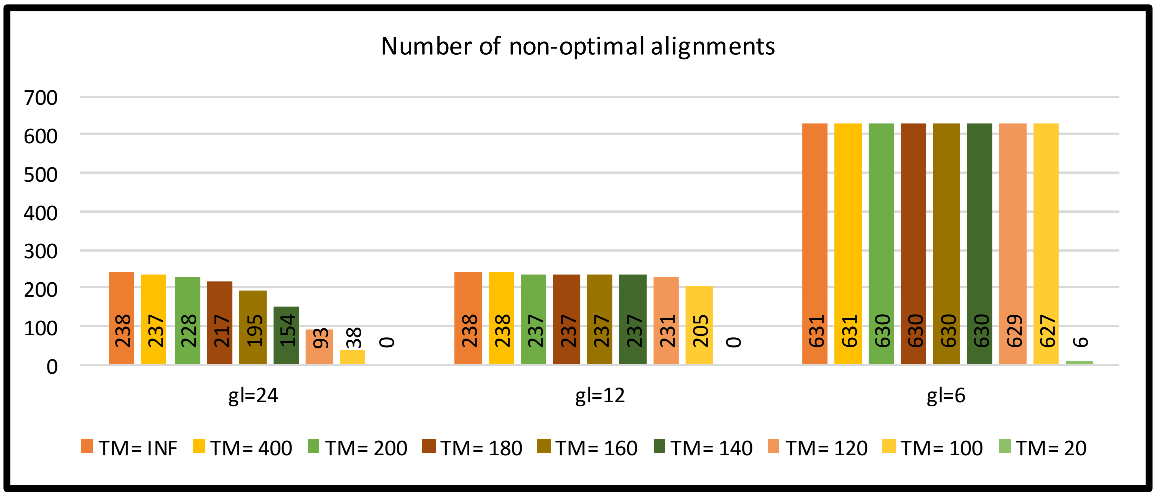

Figure 101: Number of suboptimal alignments for DLL dataset when TM varies.

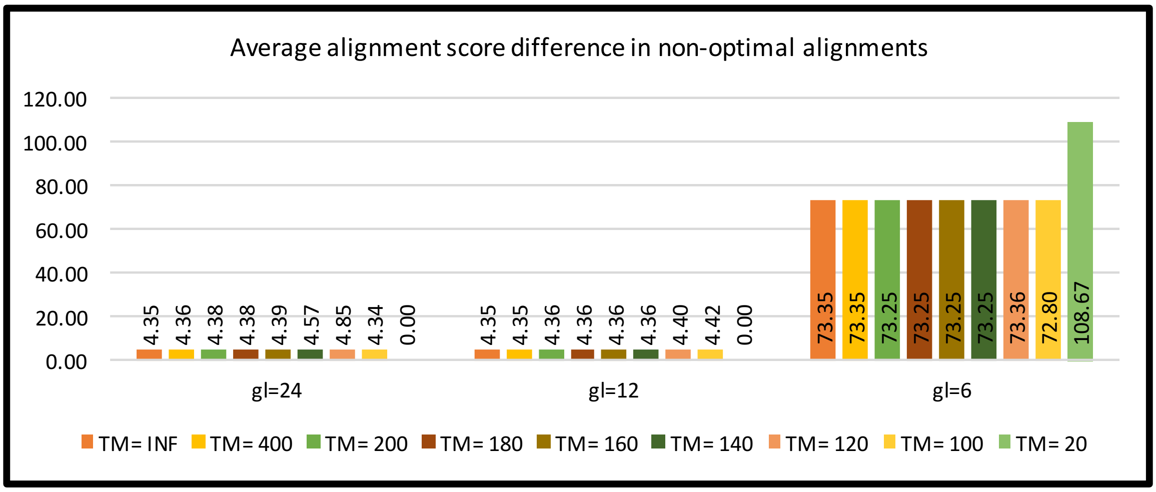

Figure 102: Average alignment score difference in suboptimal alignments for DLL dataset when TM varies.

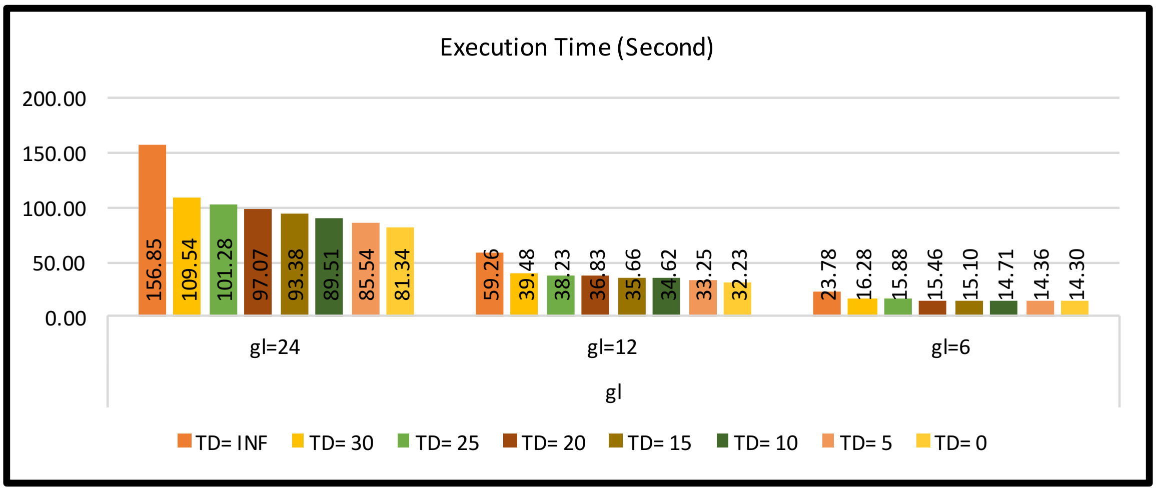

Figure 103: Execution times for DLL dataset when TD varies.

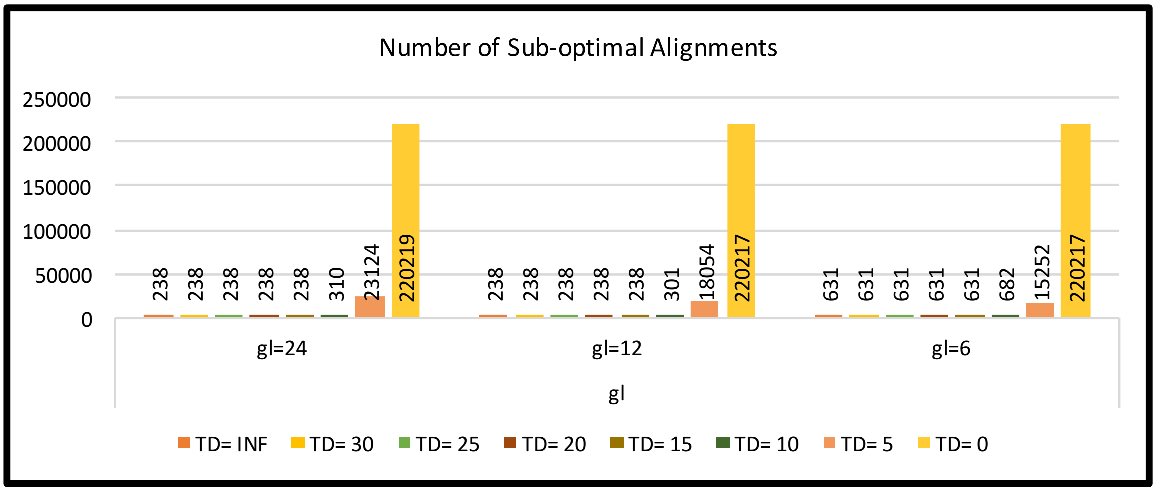

Figure 104: Number of suboptimal alignments for DLL dataset when TD varies.

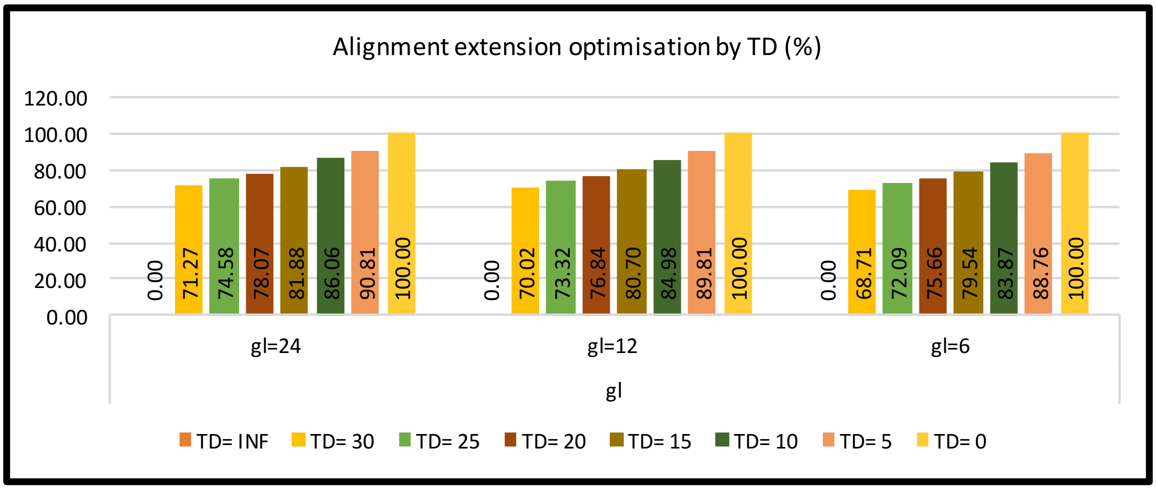

Figure 105: Average alignment score difference in suboptimal alignments for DLL dataset when TM varies.

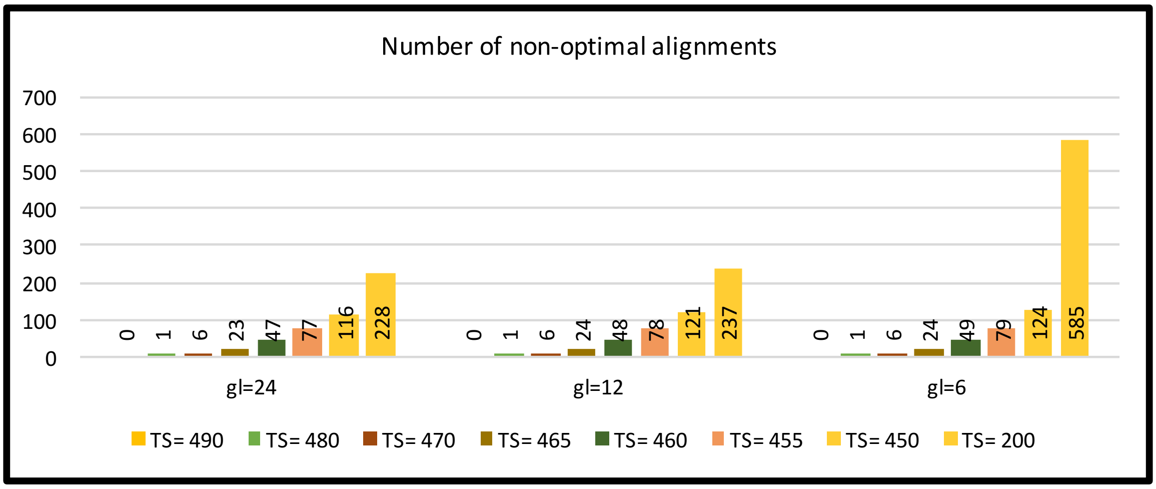

Figure 106: Execution times for DLL dataset when TS varies.

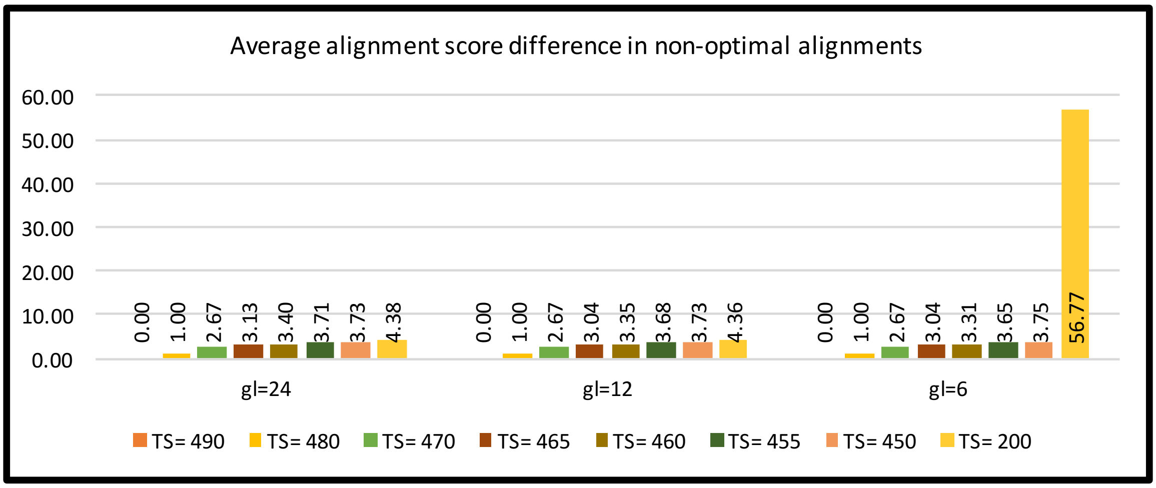

Figure 107: Number of suboptimal alignments for DLL dataset when TS varies.

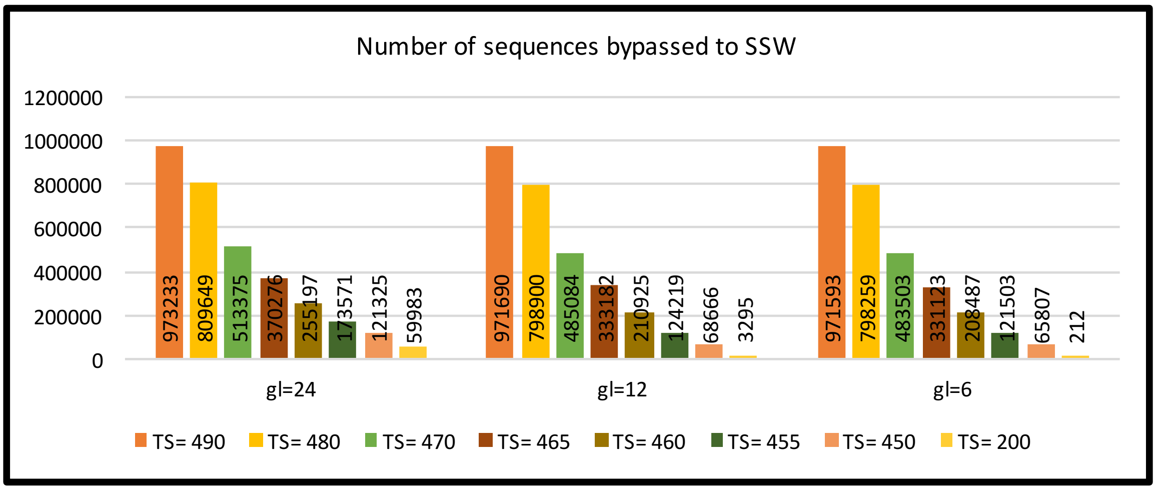

Figure 108: Average alignment score difference in suboptimal alignments for DLL dataset when TS varies.

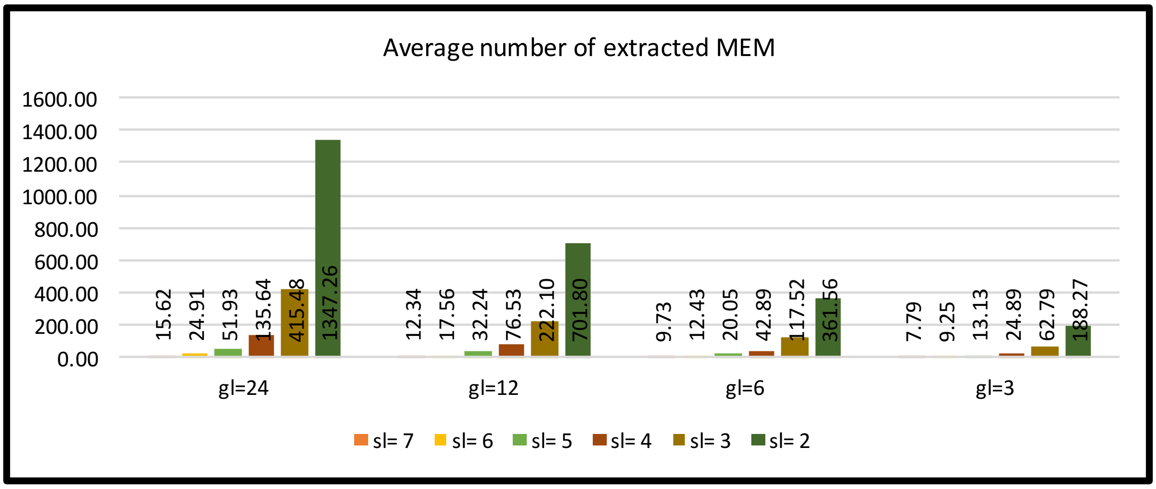

Figure 109: Average number of extracted MEM for DLL dataset when gl and sl varies.

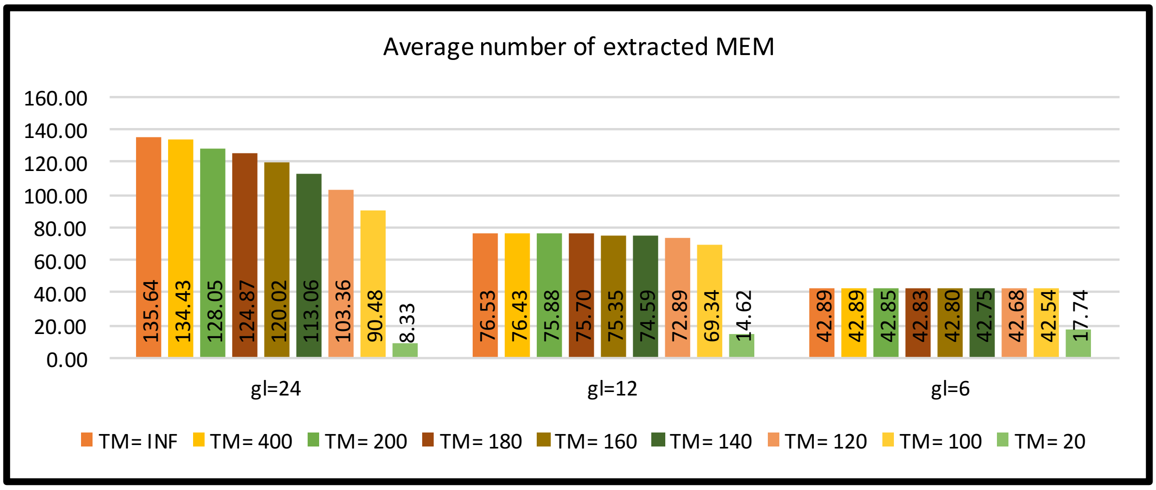

Figure 110: Average number of extracted MEM for DLL dataset when TM varies.

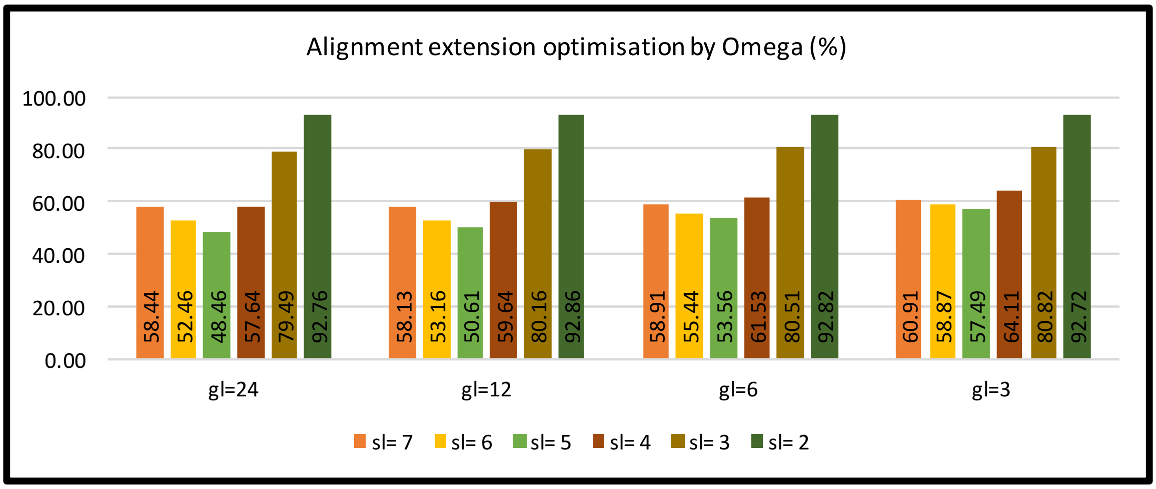

Figure 111: Proportion of alignment extension which are optimised (avoided) by the set Ω when processing DLL dataset.

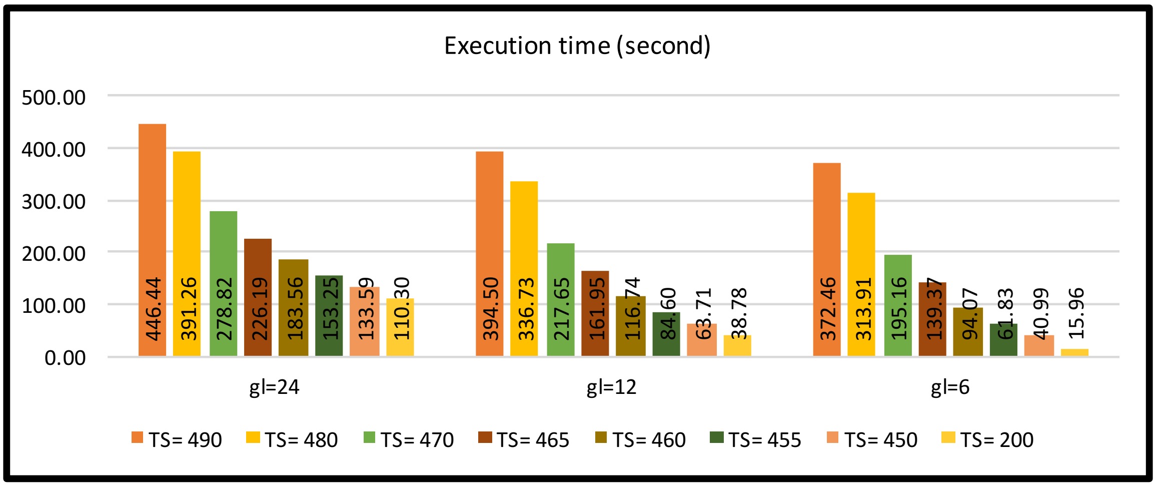

Figure 112: Proportion of alignment extension which are optimised (avoided) by TD after applying the set Ω when processing DLL dataset.

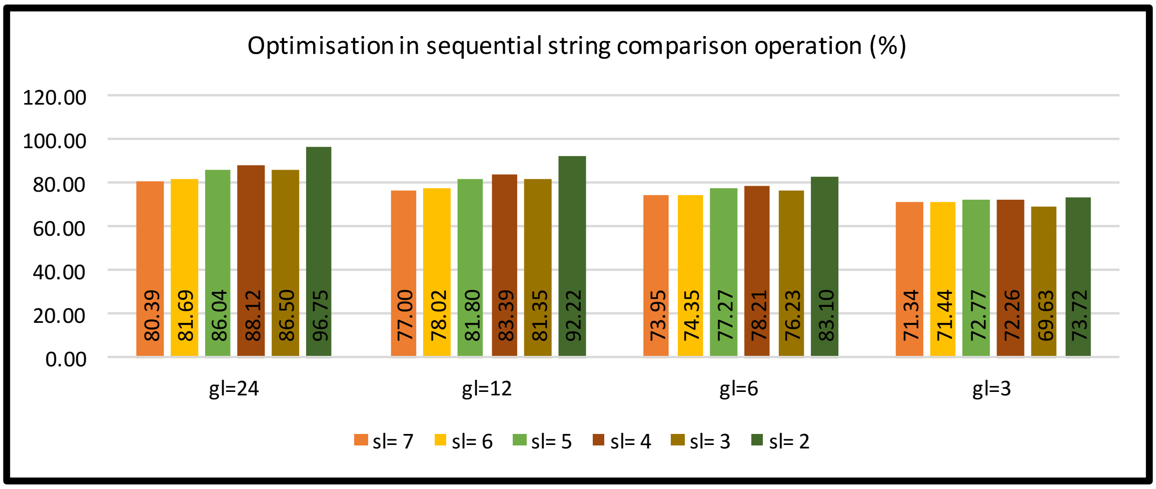

Figure 113: Proportion of sequential string compare operation which are optimised (avoided) when processing DLL dataset.

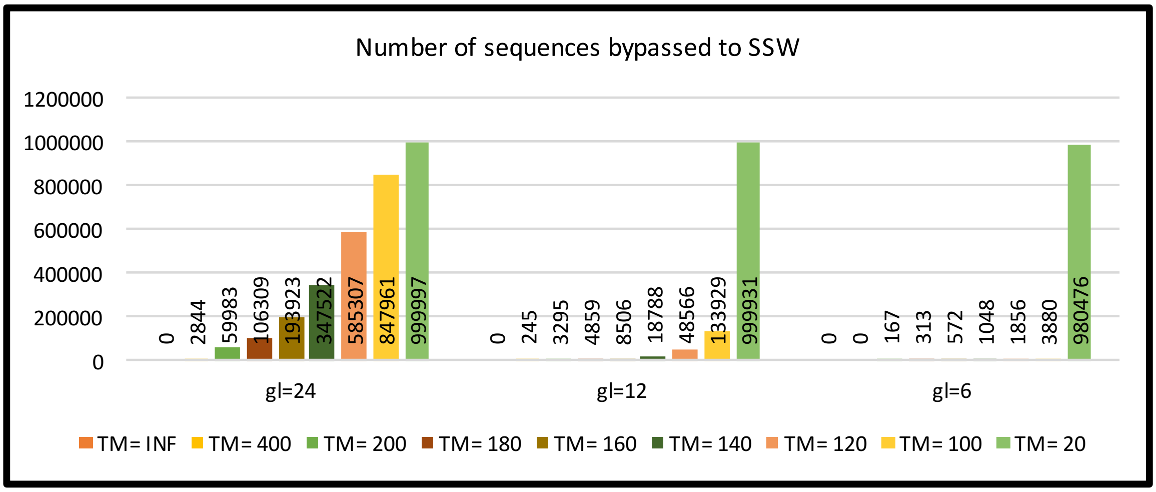

Figure 114: Number of sequences bypassed to SSW by TM (TS has not been applied) when processing DLL dataset.

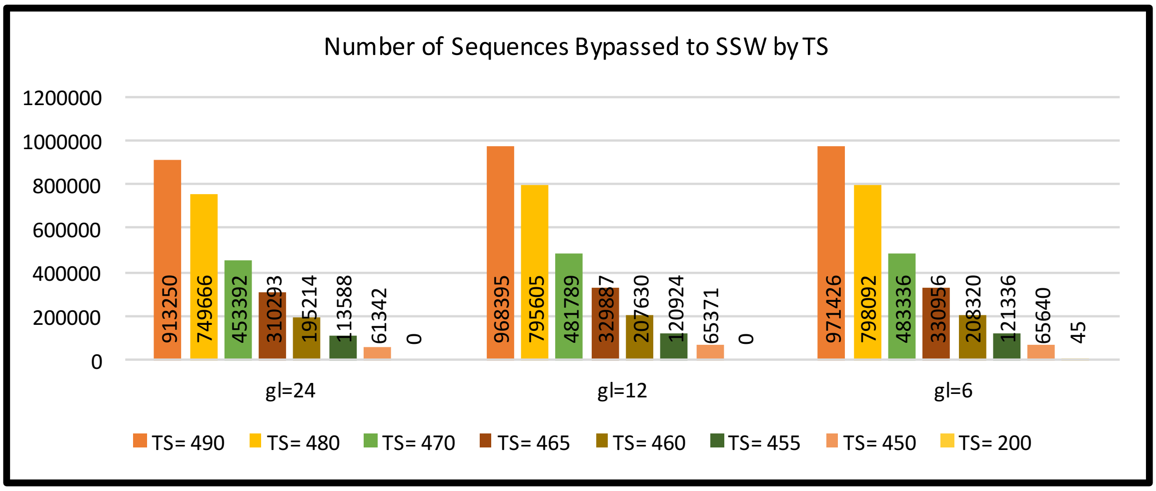

Figure 115: Total number of sequences bypassed to SSW by TM and TS when processing DLL dataset.

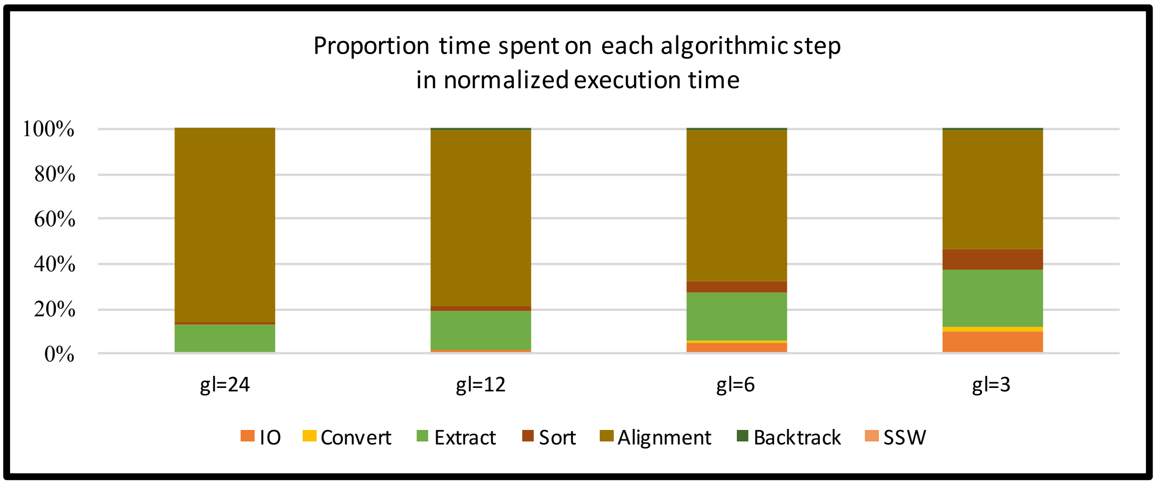

Figure 116: Total number of sequences bypassed to SSW by TS when processing DLL dataset.

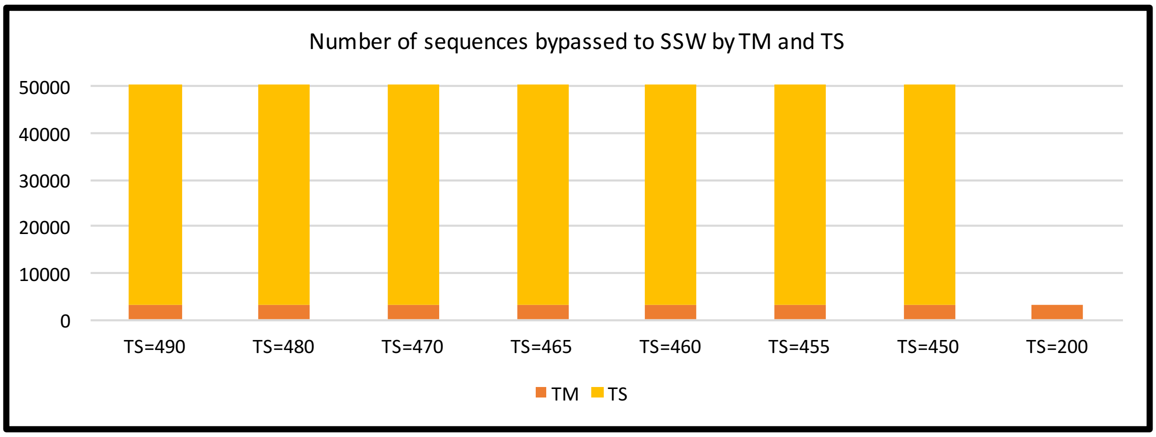

Figure 117: Proportion of input sequence pairs bypassed to SSW by TM and TS when processing DLL dataset.

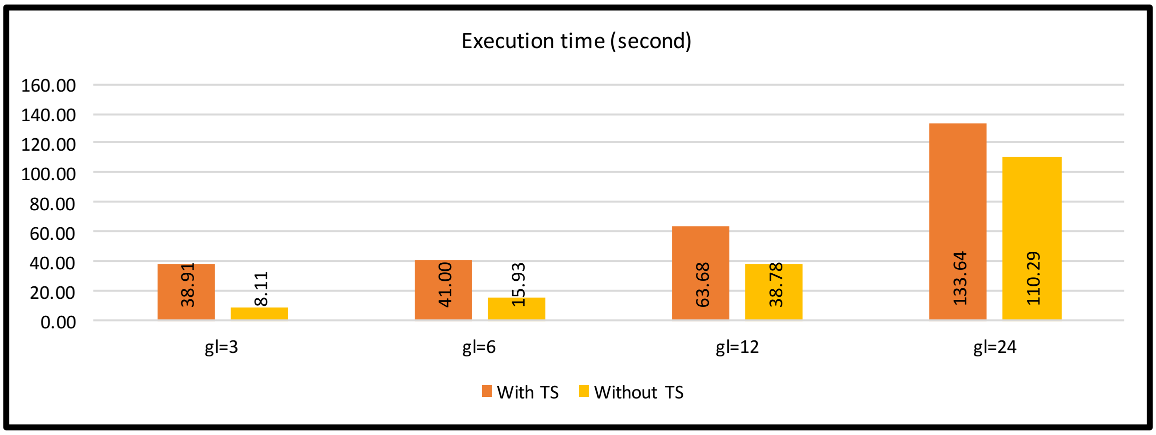

Figure 118: Proportion of input sequence pairs bypassed to SSW by TM and TS (scaled) when processing DLL dataset.

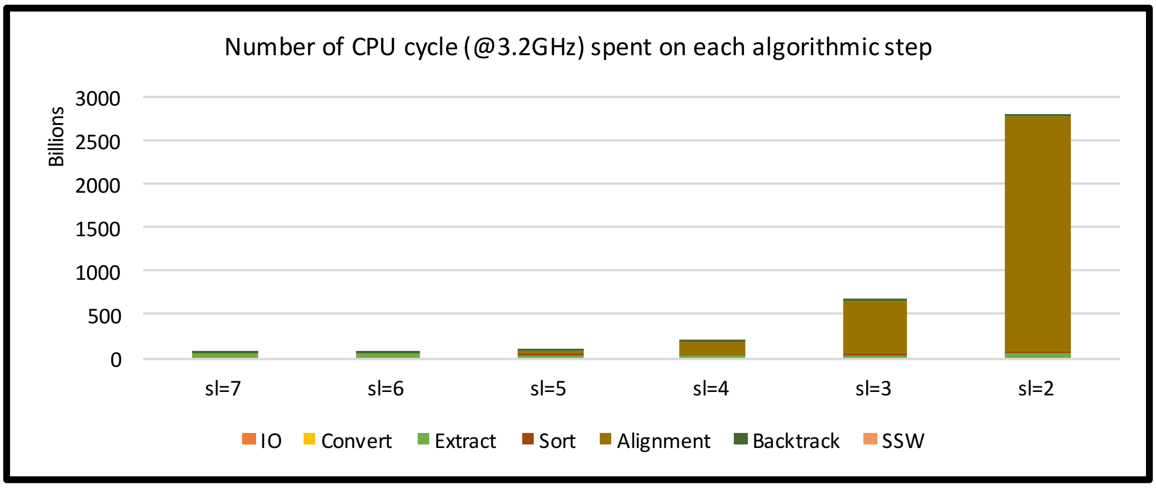

Figure 119: Cycle accurate execution time (DLL dataset) of differing algorithmic steps of *MEM-Align* when *gl* varies (in CPU cycle).

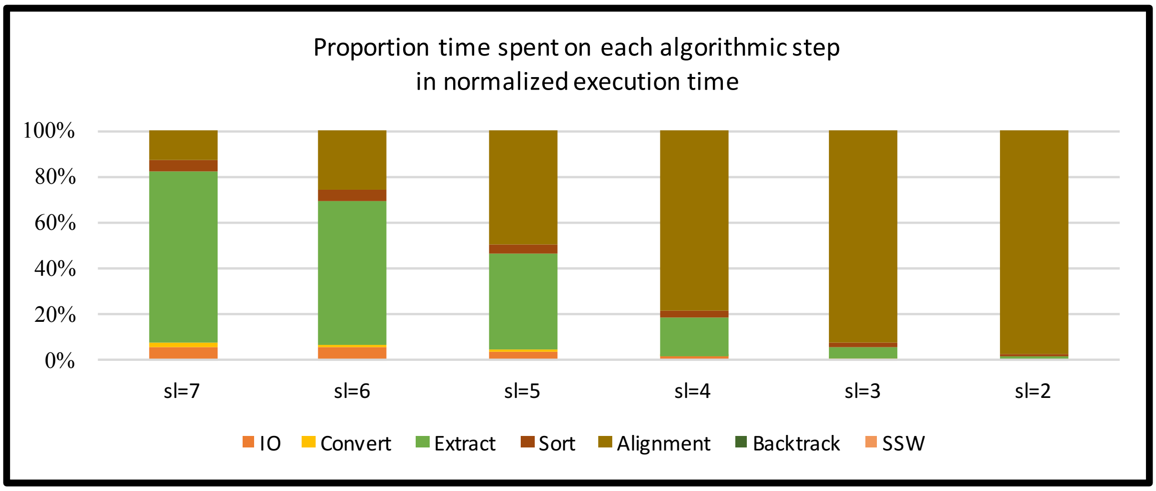

Figure 120: normalised cycle accurate execution time (DLL dataset) of differing algorithmic steps of *MEM-Align* when *gl* varies.

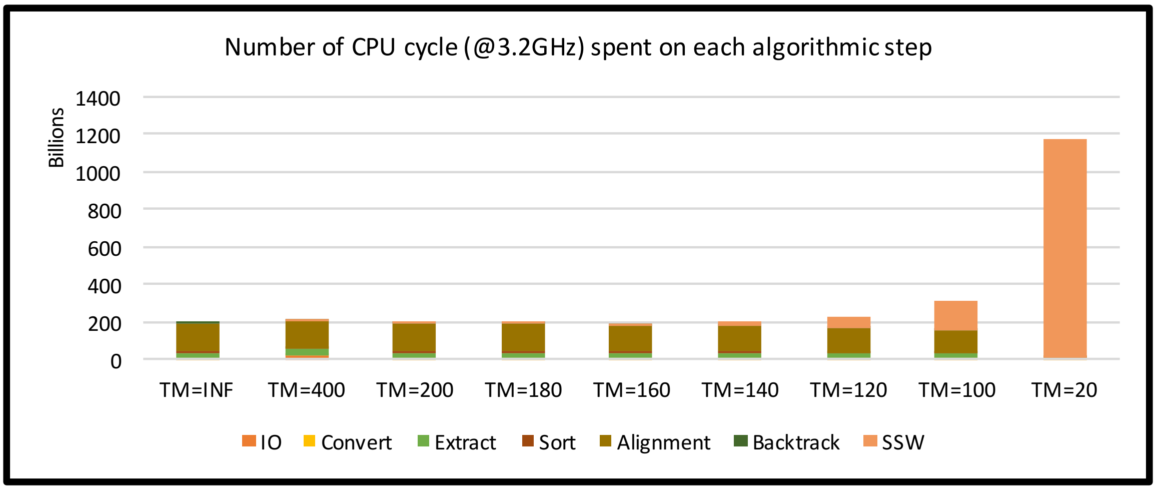

Figure 121: Cycle accurate execution time (DLL dataset) of differing algorithmic steps of *MEM-Align* when *sl* varies (in CPU cycle).

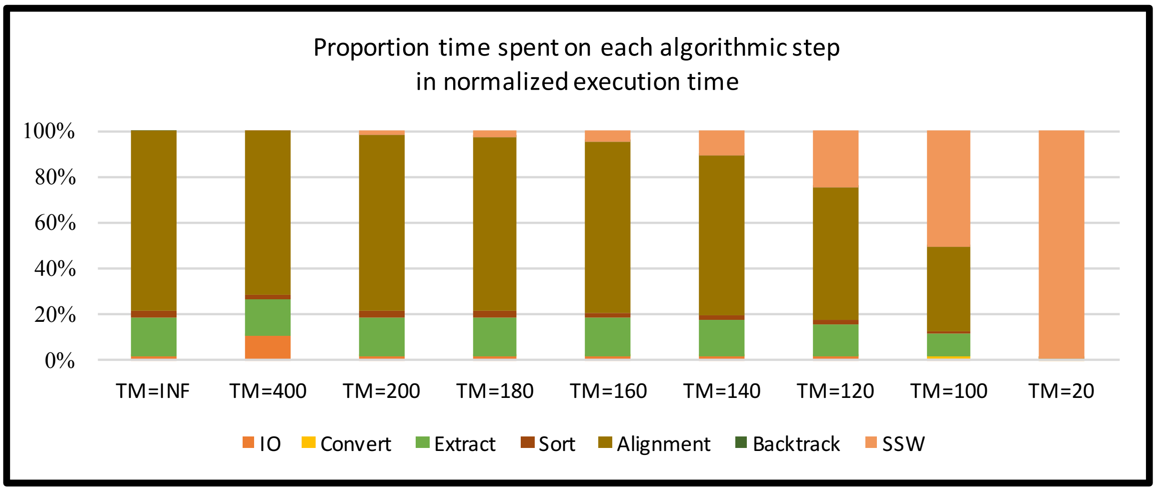

Figure 122: normalised cycle accurate execution time (DLL dataset) of differing algorithmic steps of *MEM-Align* when *sl* varies.

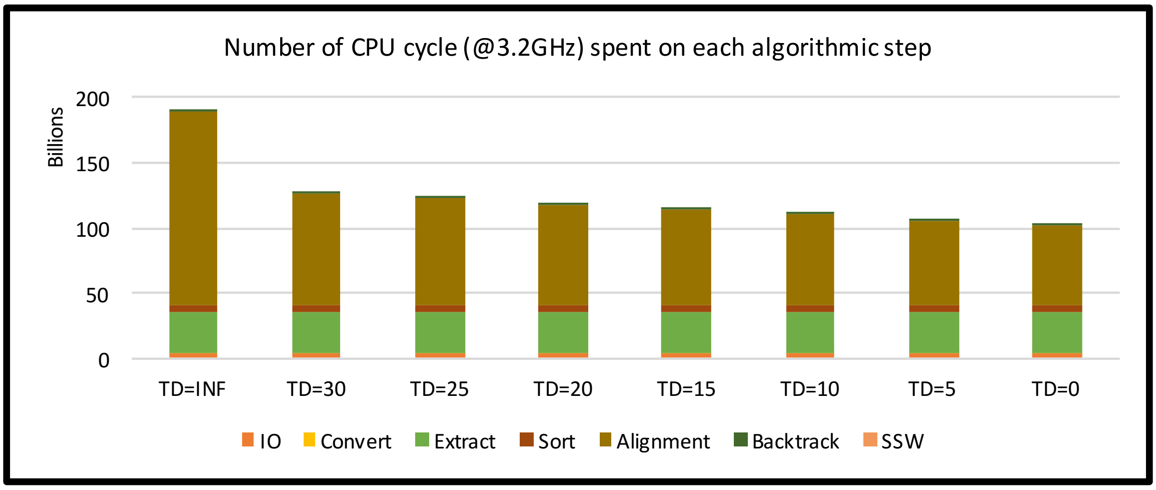

Figure 123: Cycle accurate execution time (DLL dataset) of differing algorithmic steps of *MEM-Align* when TM varies (in CPU cycle).

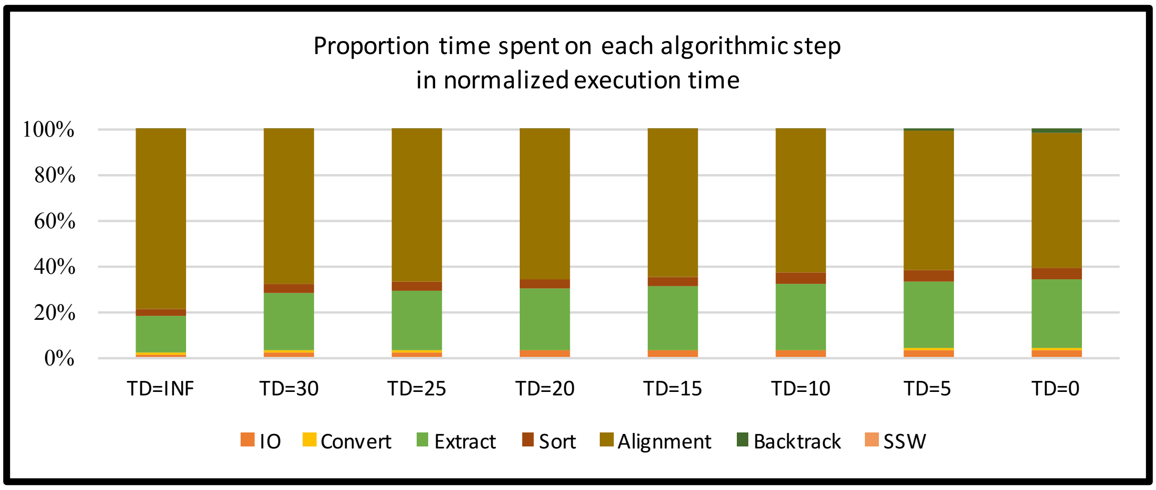

Figure 124: normalised cycle accurate execution time (DLL dataset) of differing algorithmic steps of *MEM-Align* when TM varies.

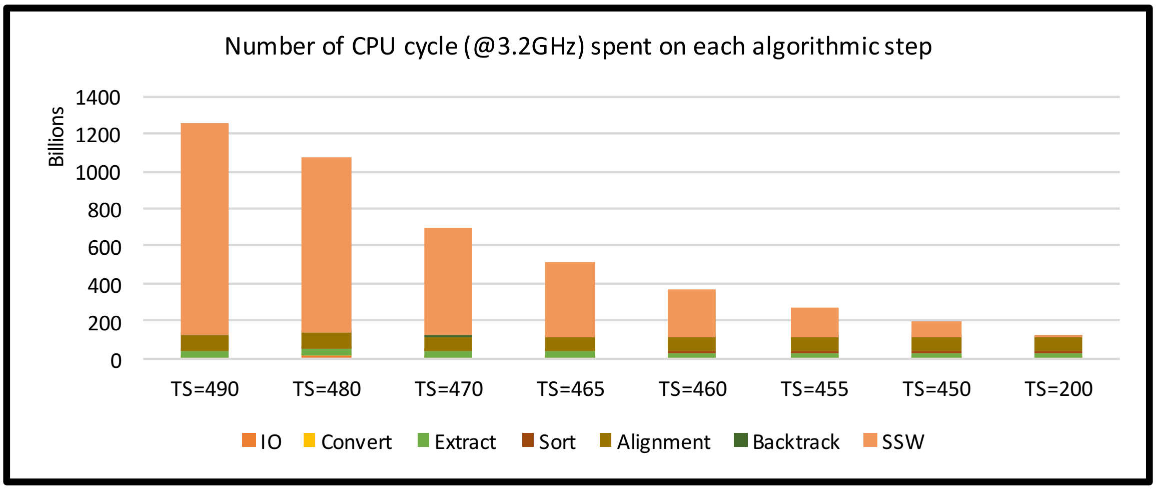

Figure 125: Cycle accurate execution time (DLL dataset) of differing algorithmic steps of *MEM-Align* when TD varies (in CPU cycle).

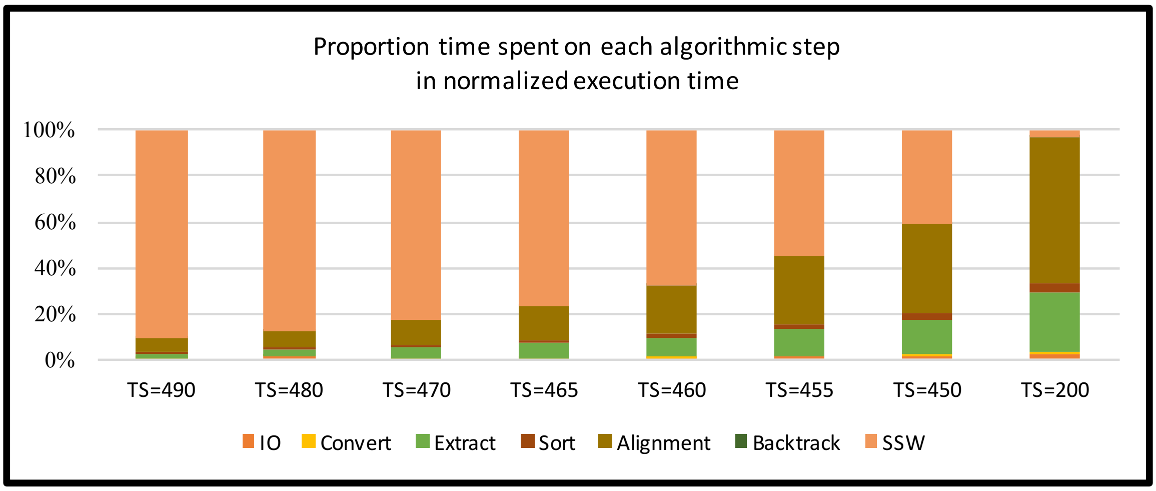

Figure 126: normalised cycle accurate execution time (DLL dataset) of differing algorithmic steps of *MEM-Align* when TD varies.

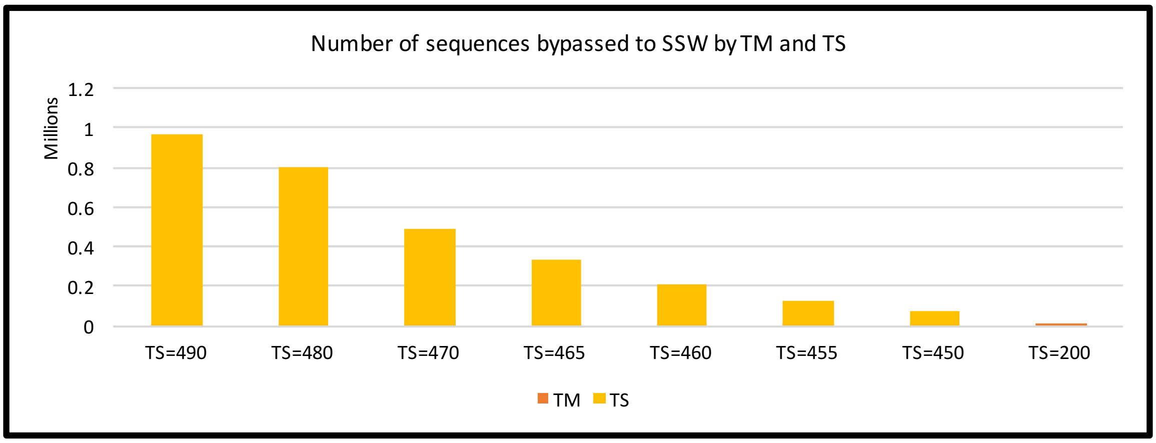

Figure 127: Cycle accurate execution time (DLL dataset) of differing algorithmic steps of *MEM-Align* when TS varies (in CPU cycle).

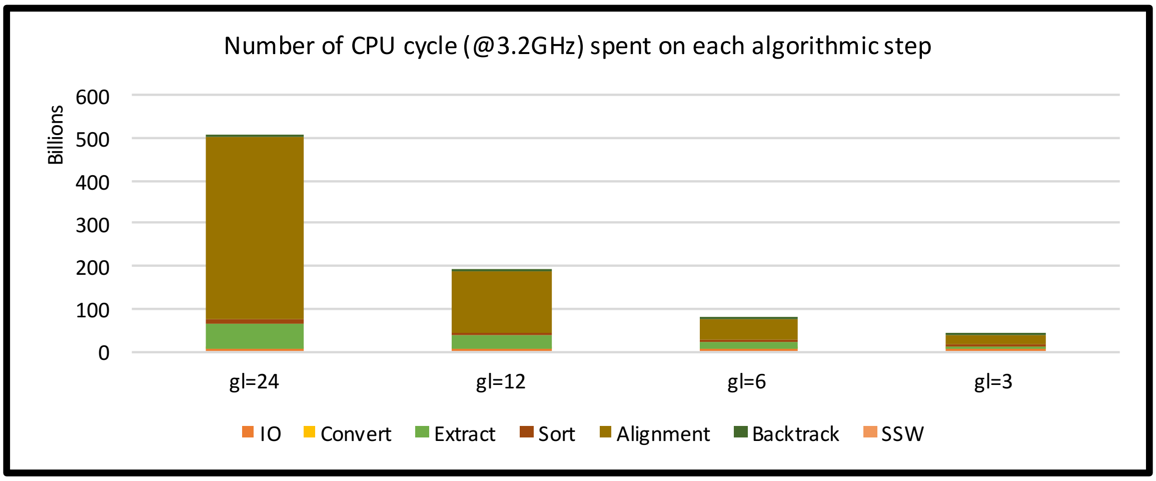

Figure 128: normalised cycle accurate execution time (DLL dataset) of differing algorithmic steps of *MEM-Align* when TS varies.

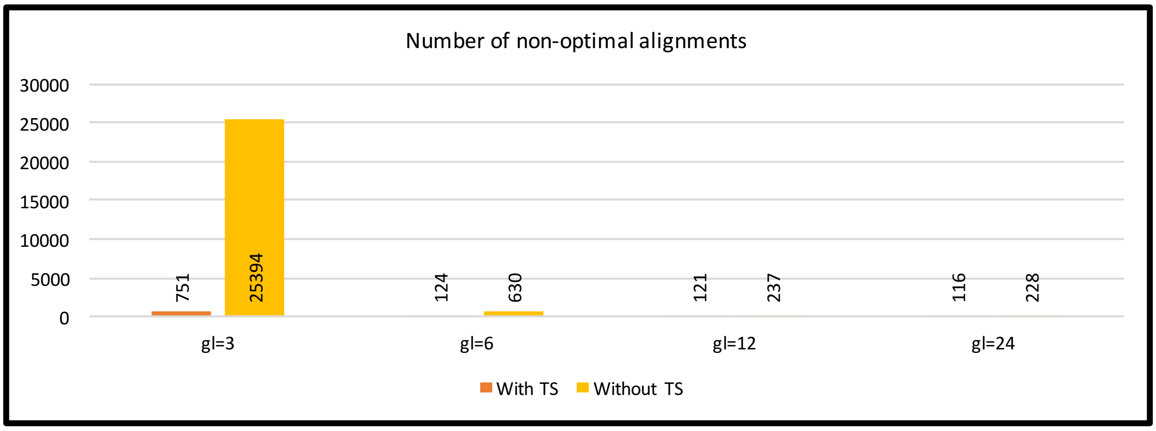

Figure 129: Execution time (DLL dataset) for differing gl with and without TS.

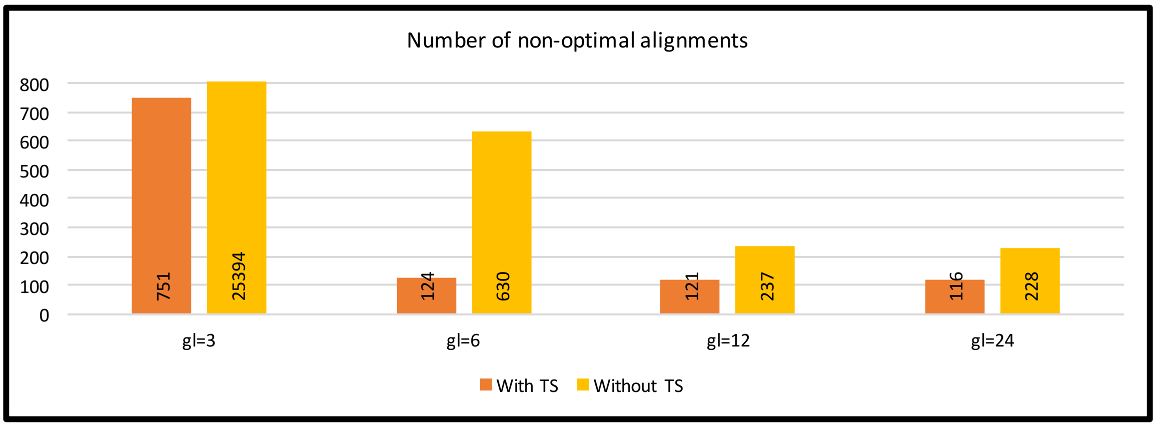

Figure 130: Number of suboptimal alignments (DLL dataset) for differing gl with and without TS.

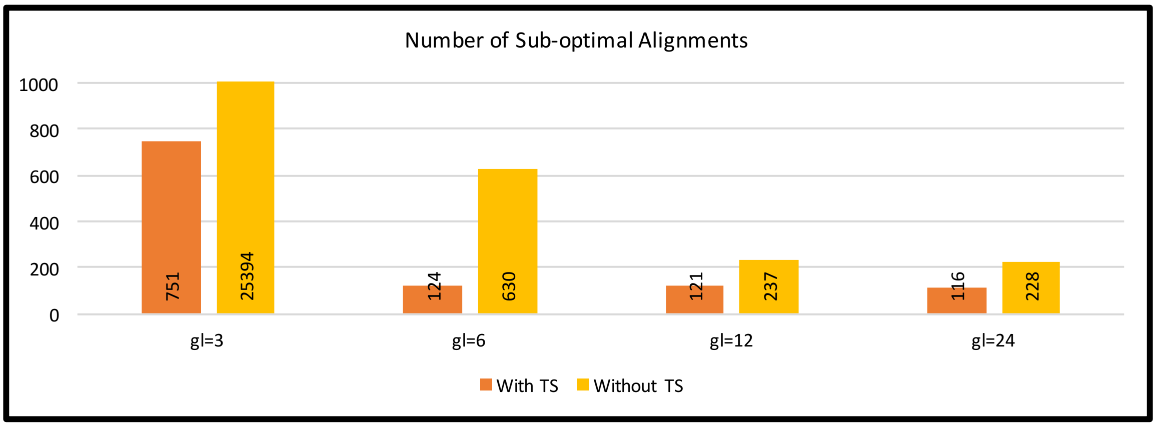

Figure 131: Number of suboptimal alignments (DLL dataset) for differing gl with and without TS (scaled).

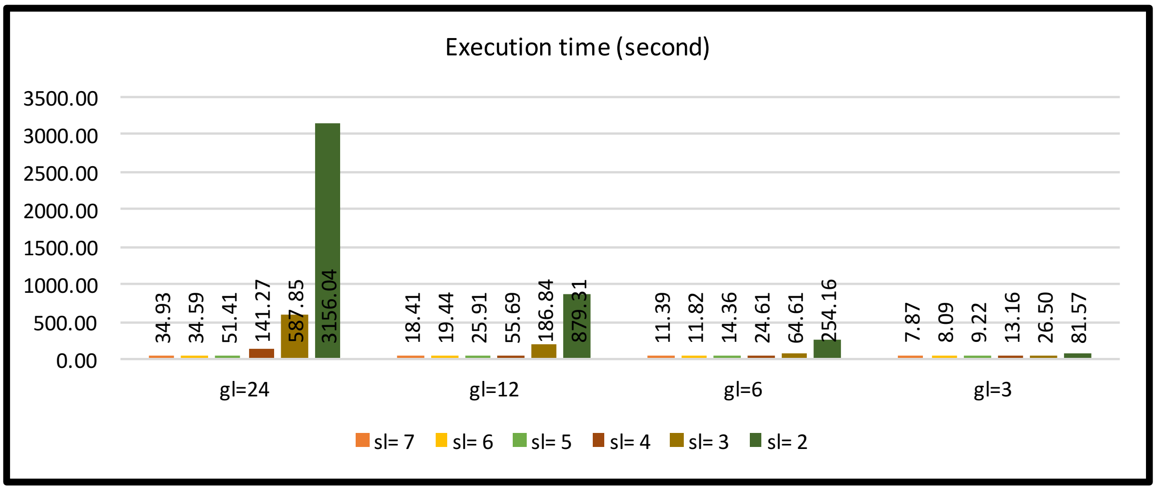

Figure 132: Execution times for DLH dataset when *gl* and *sl* varies.

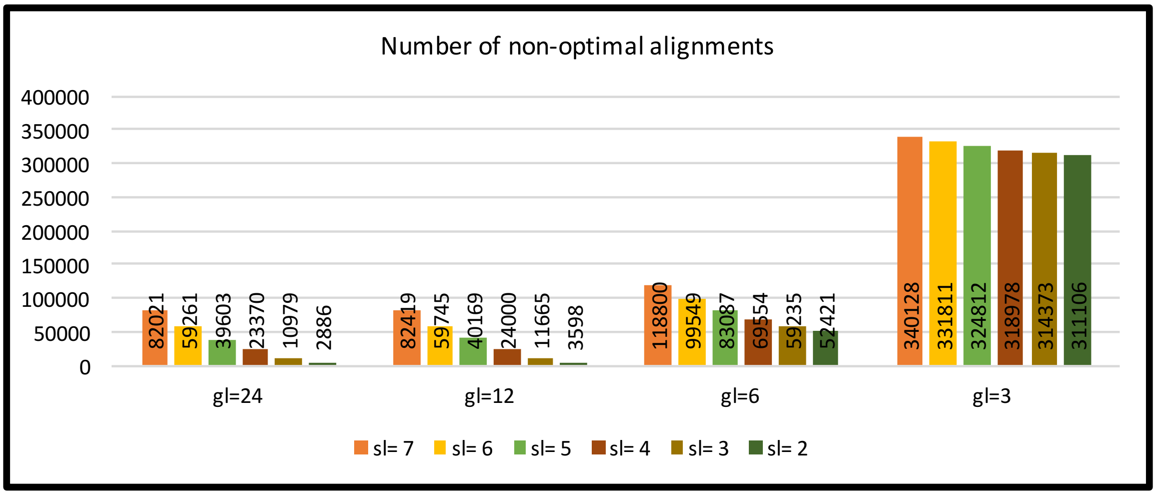

Figure 133: Number of suboptimal alignments for DLH dataset when *gl* and *sl* varies.

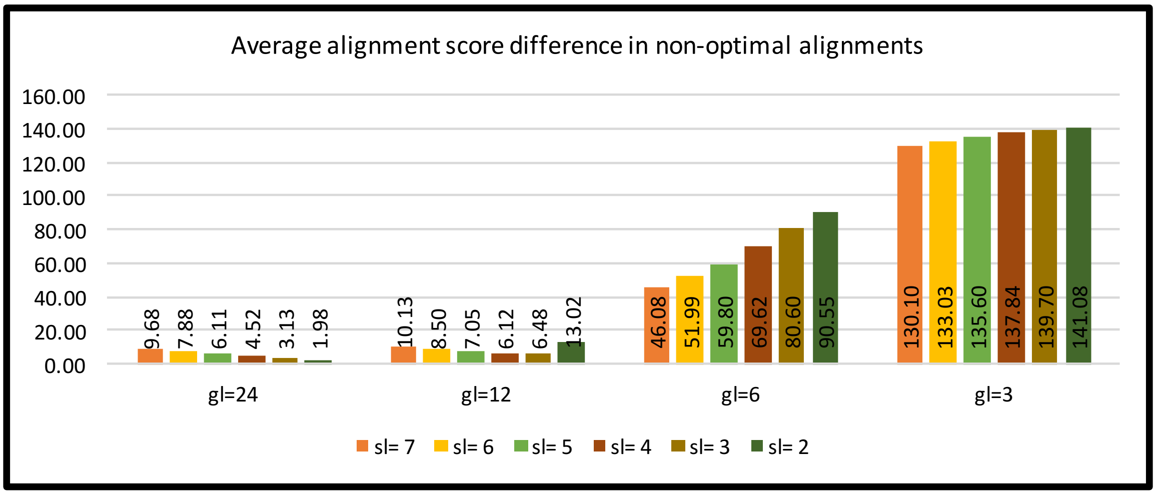

Figure 134: Average alignment score difference in suboptimal alignments for DLH dataset when *gl* and *sl* varies.

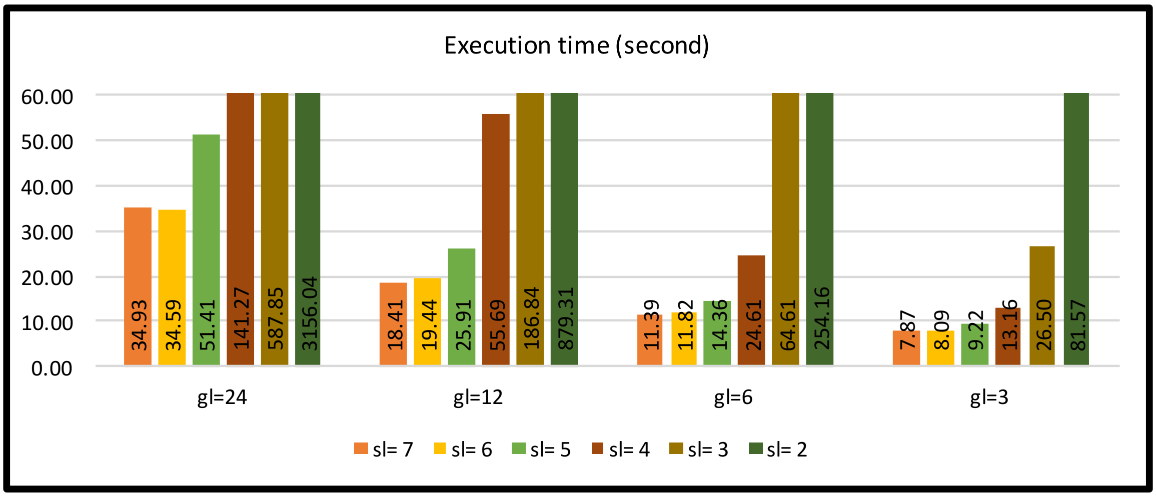

Figure 135: Execution times for DLH dataset when *gl* and *sl* varies (scaled).

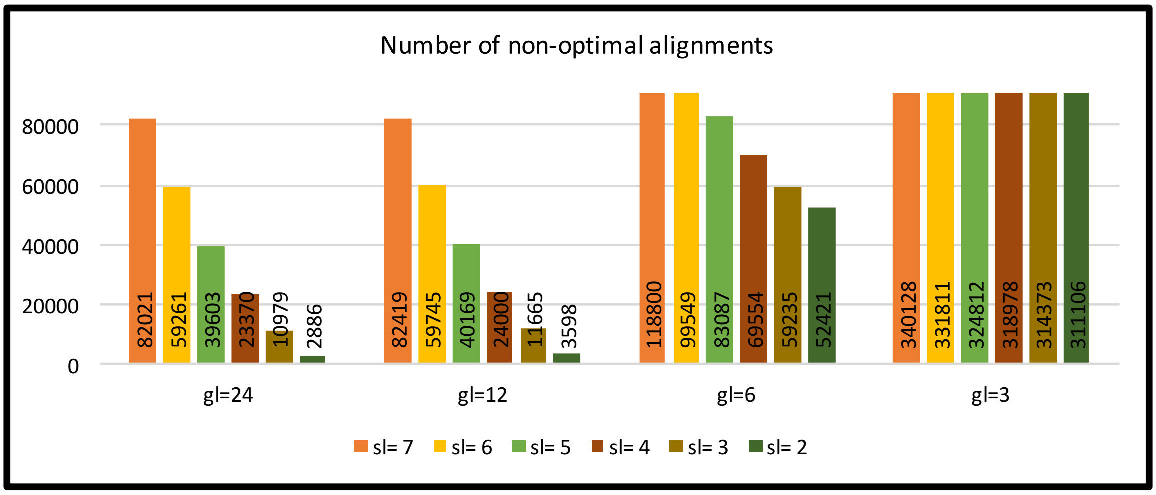

Figure 136: Number of suboptimal alignments for DLH dataset when *gl* and *sl* varies (scaled).

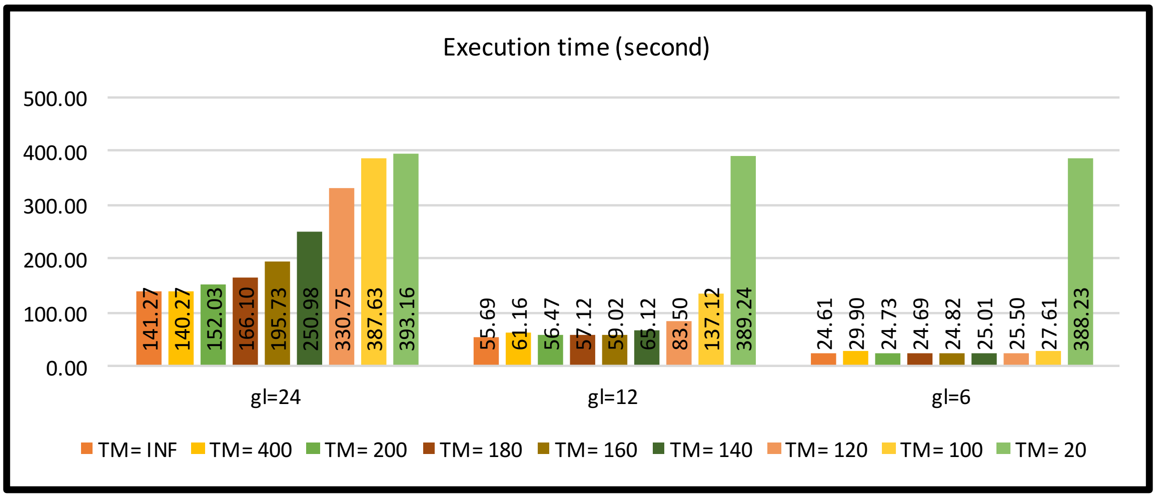

Figure 137: Execution times for DLH dataset when TM varies.

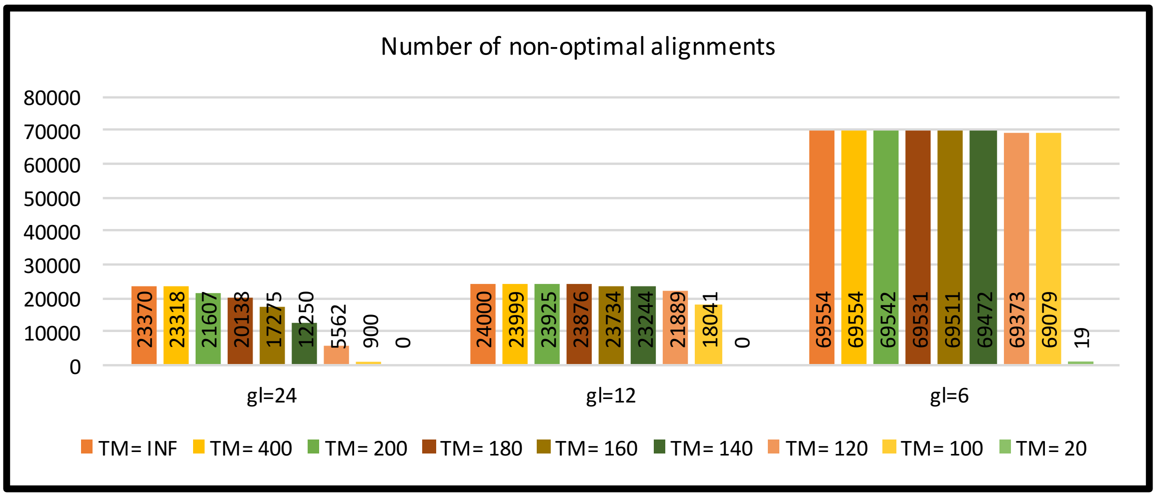

Figure 138: Number of suboptimal alignments for DLH dataset when TM varies.

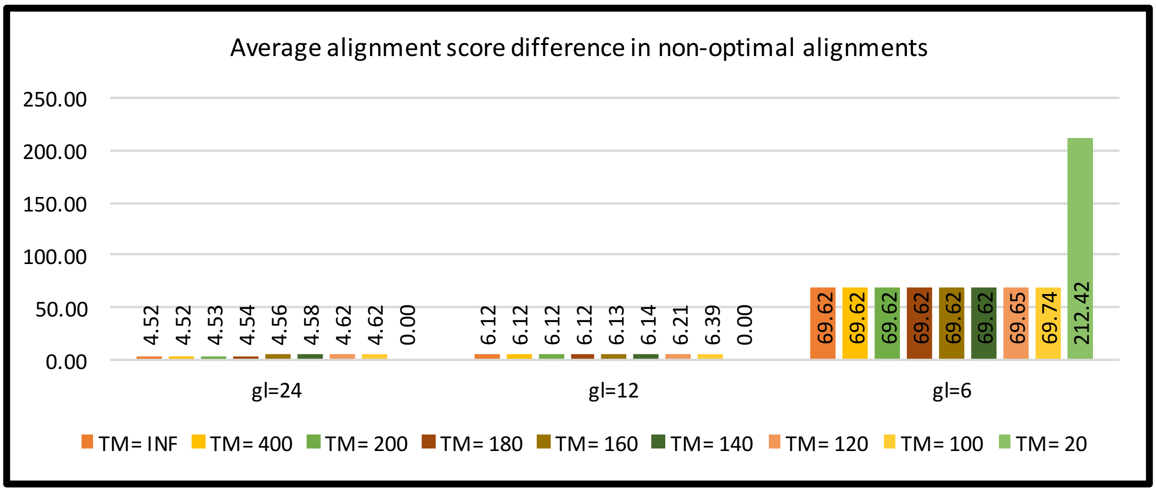

Figure 139: Average alignment score difference in suboptimal alignments for DLH dataset when TM varies.

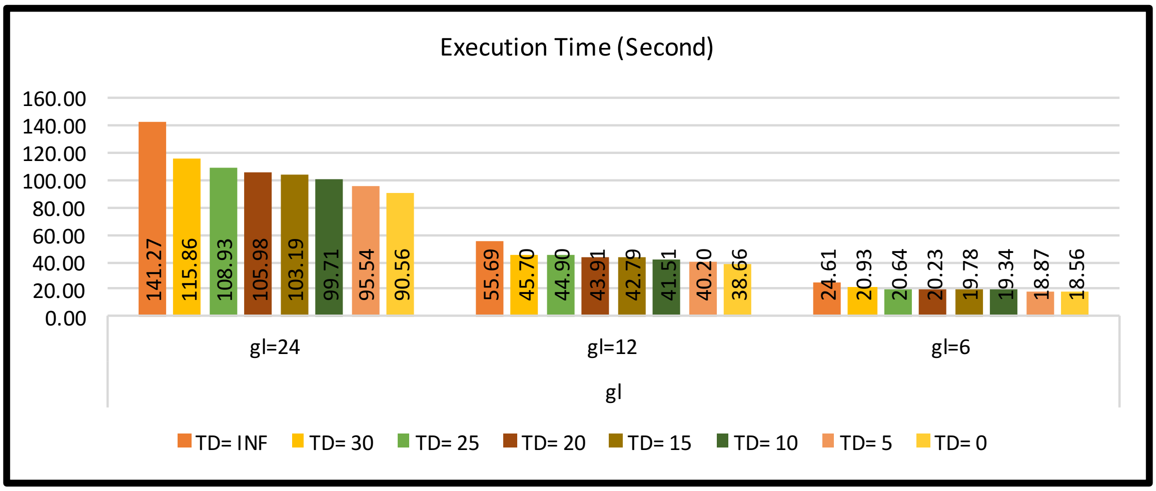

Figure 140: Execution times for DLH dataset when TD varies.

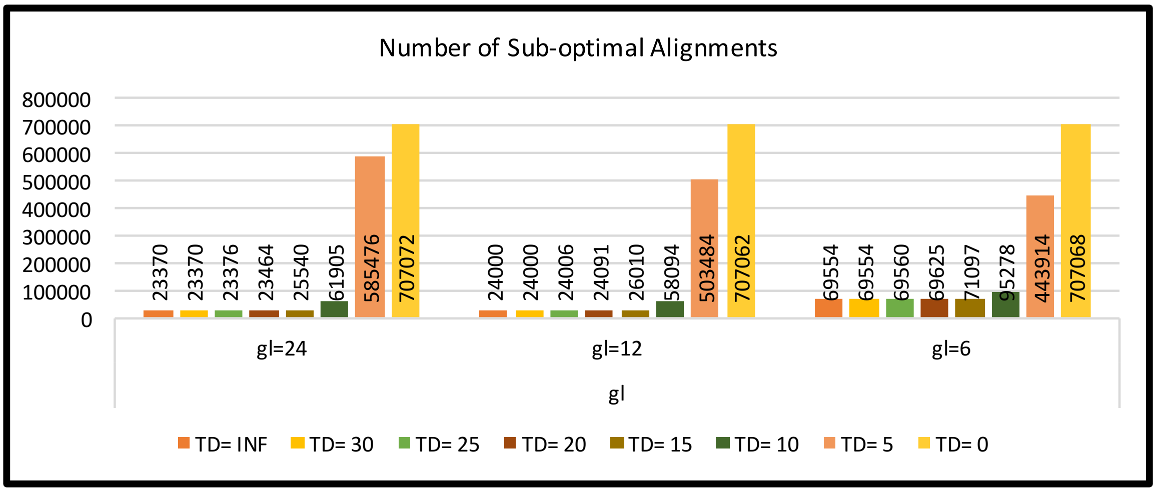

Figure 141: Number of suboptimal alignments for DLH dataset when TD varies.

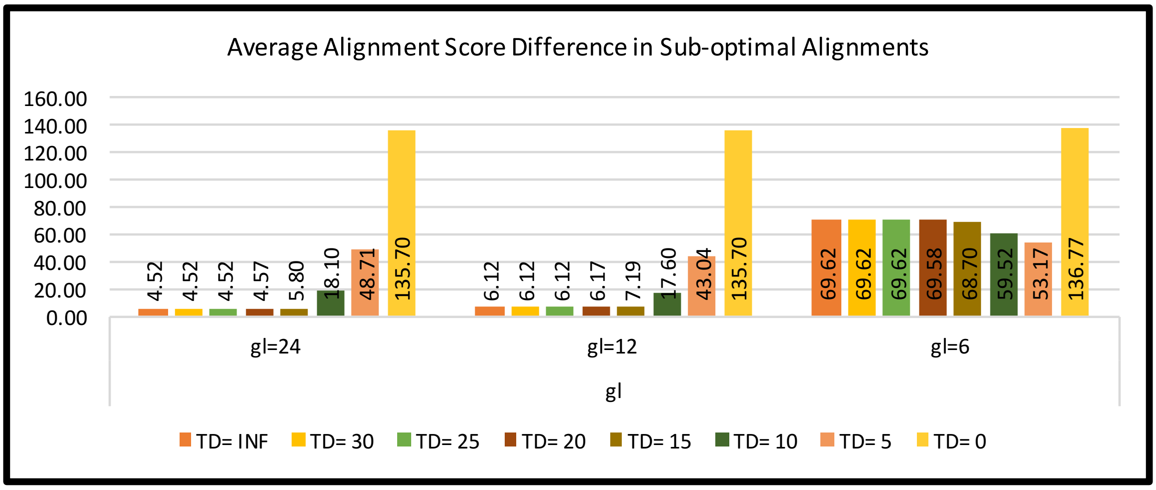

Figure 142: Average alignment score difference in suboptimal alignments for DLH dataset when TM varies.

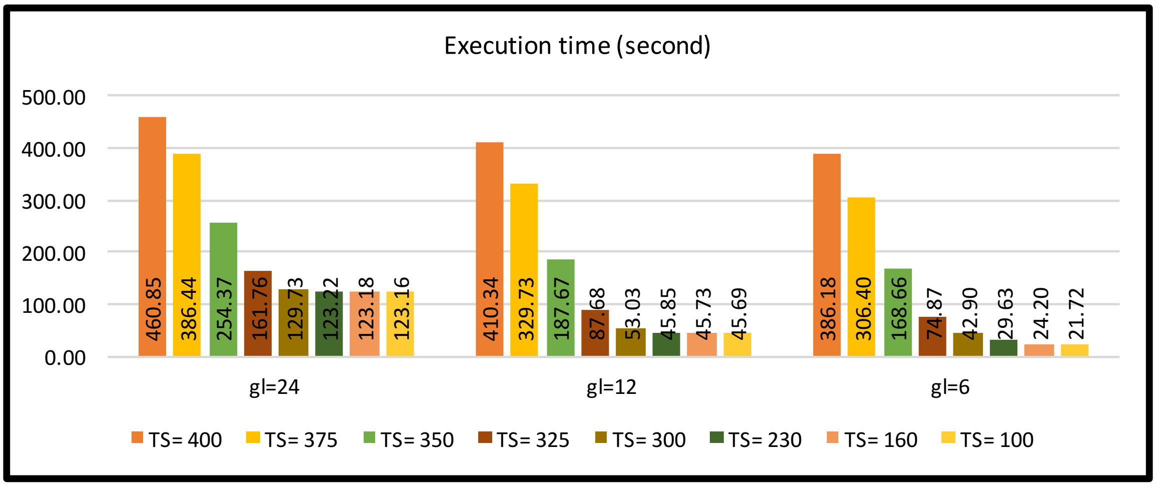

Figure 143: Execution times for DLH dataset when TS varies.

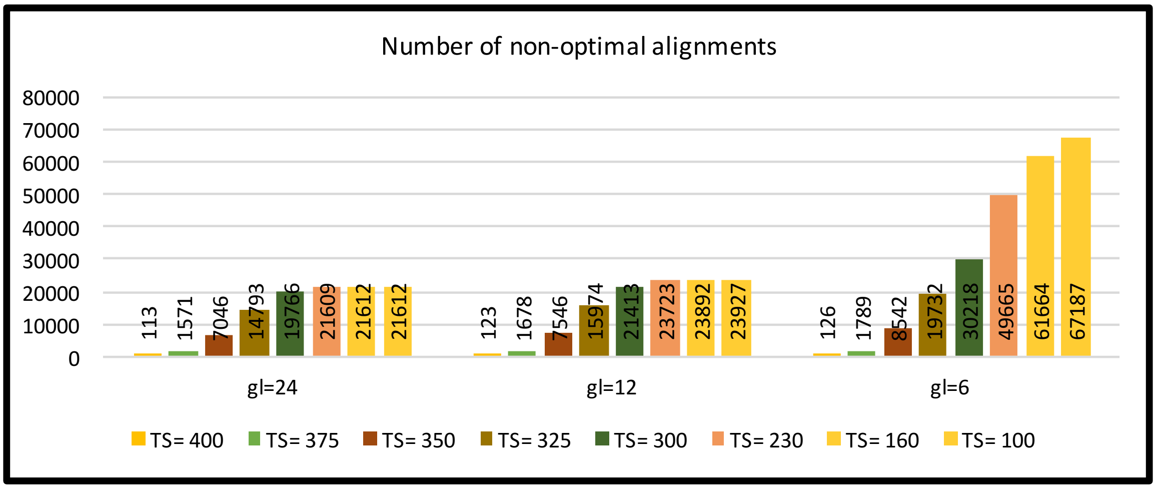

Figure 144: Number of suboptimal alignments for DLH dataset when TS varies.

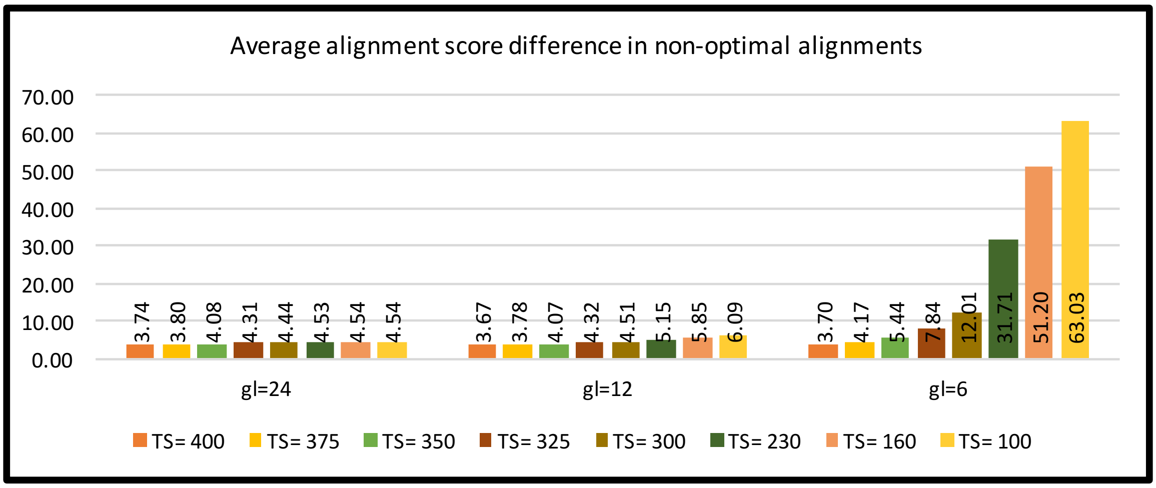

Figure 145: Average alignment score difference in suboptimal alignments for DLH dataset when TS varies.

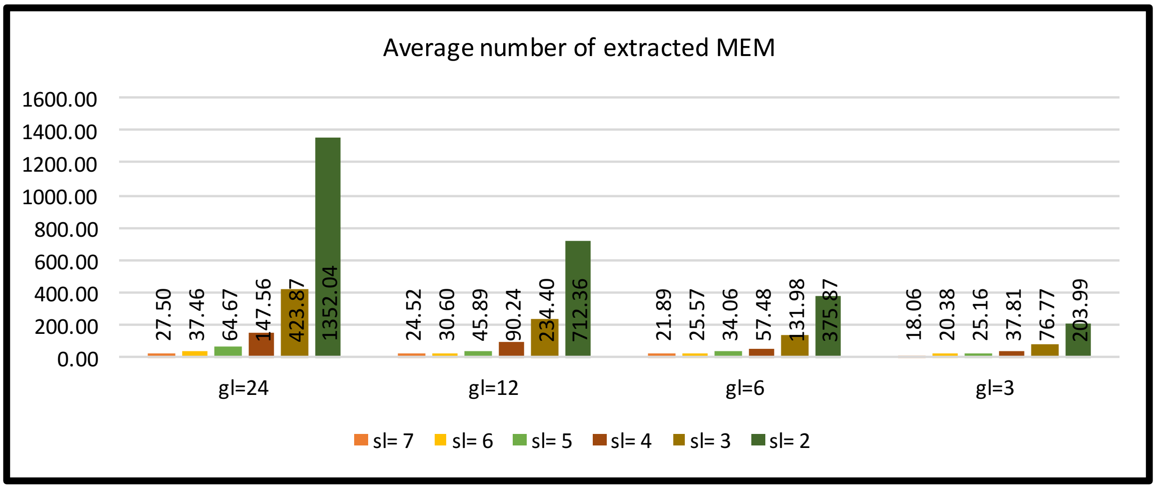

Figure 146: Average number of extracted MEM for DLH dataset when gl and sl varies.

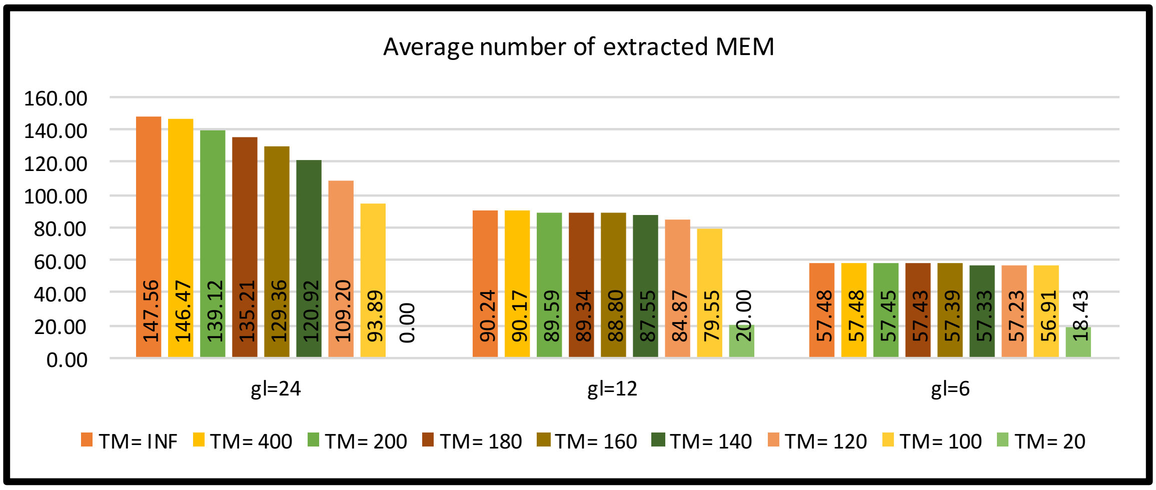

Figure 147: Average number of extracted MEM for DLH dataset when TM varies.

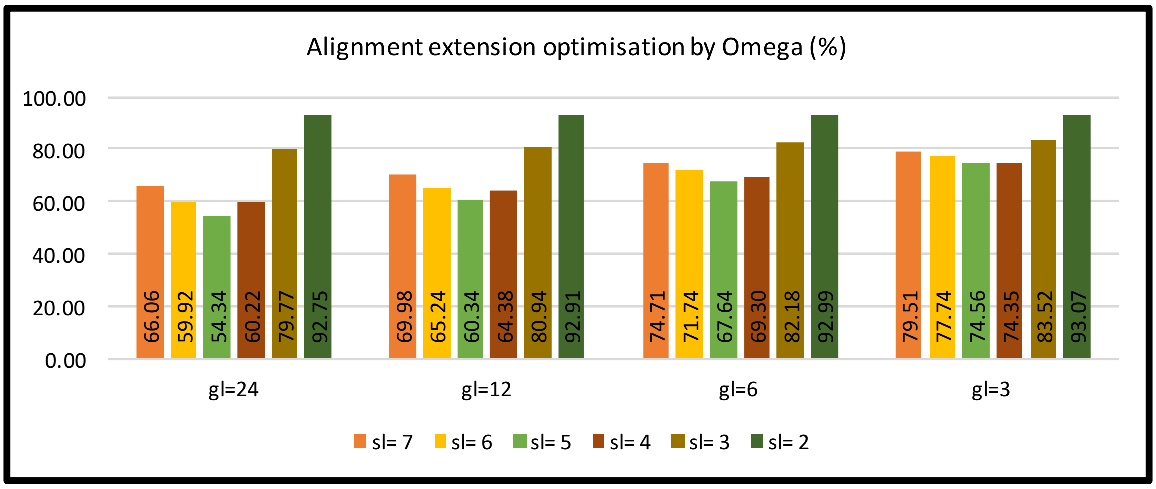

Figure 148: Proportion of alignment extension which are optimised (avoided) by the set Ω when processing DLH dataset.

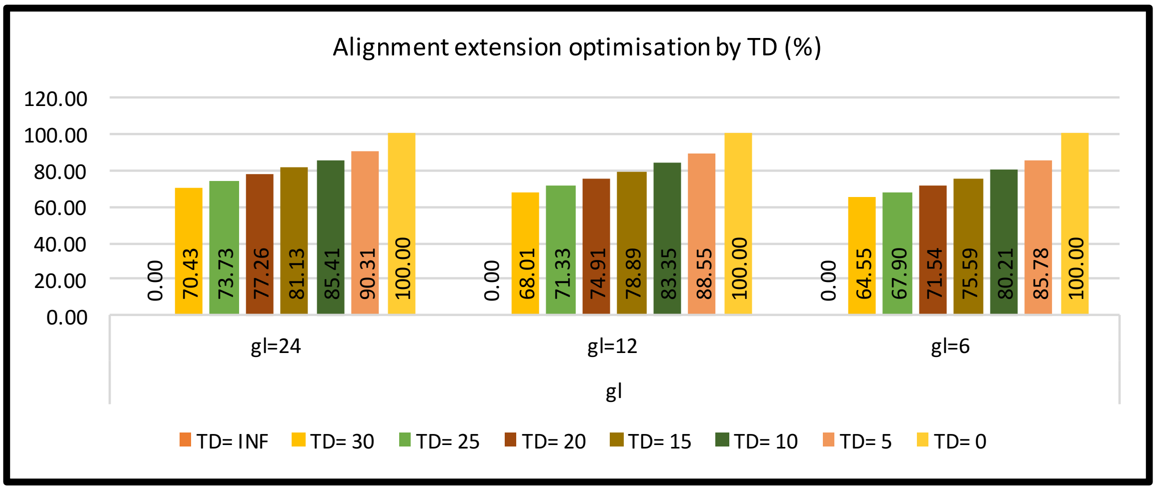

Figure 149: Proportion of alignment extension which are optimised (avoided) by TD after applying the set Ω when processing DLH dataset.

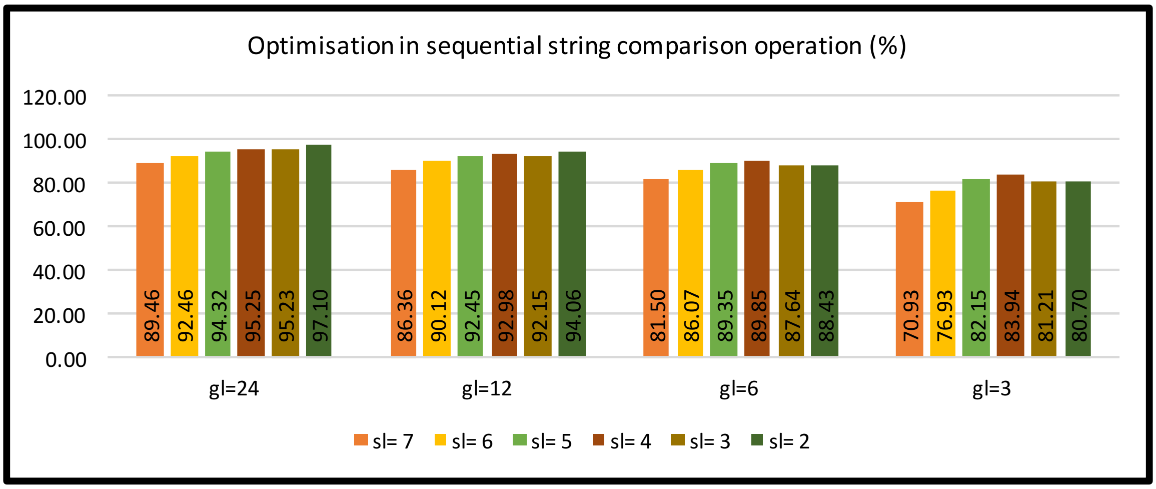

Figure 150: Proportion of sequential string compare operation which are optimised (avoided) when processing DLH dataset.

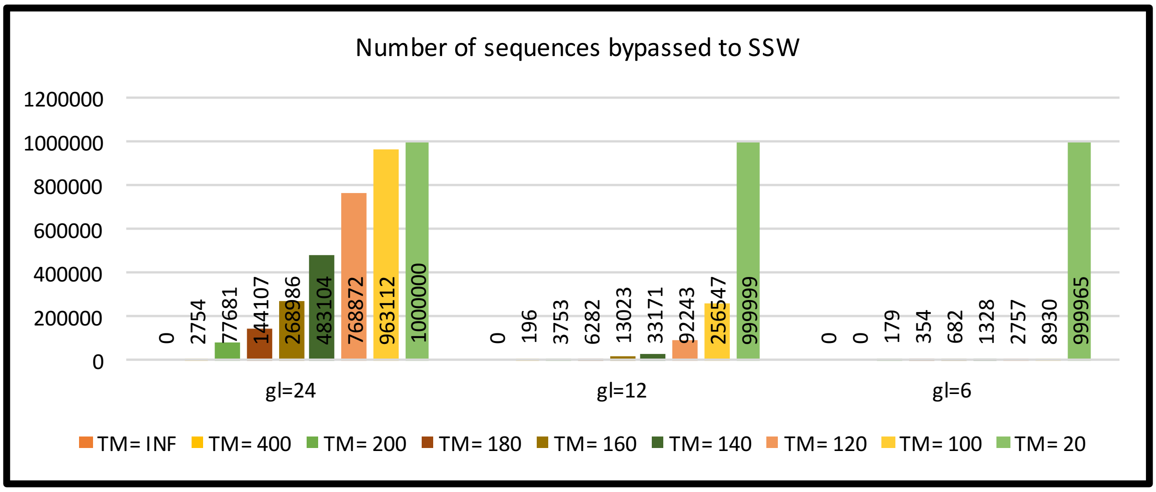

Figure 151: Number of sequences bypassed to SSW by TM (TS has not been applied) when processing DLH dataset.

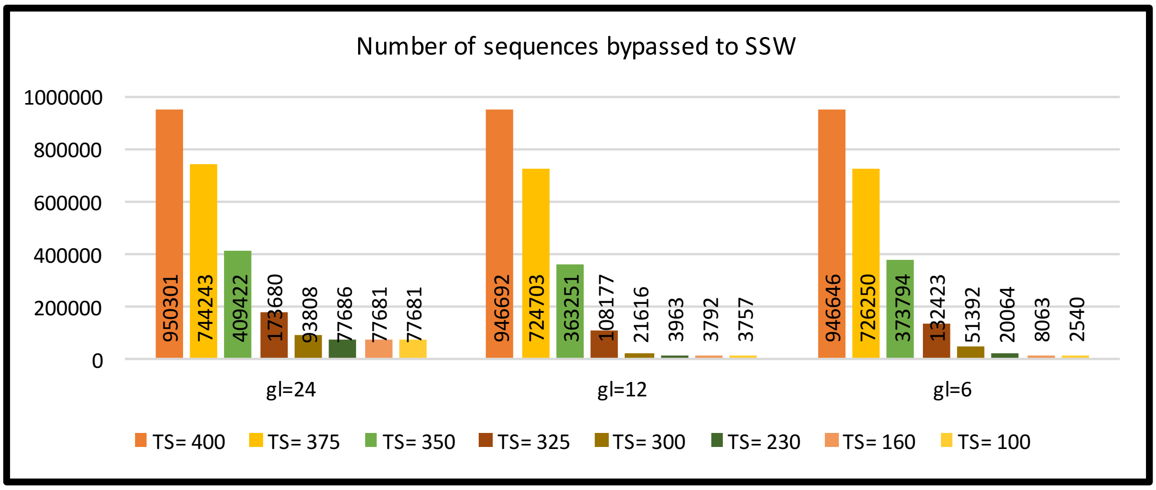

Figure 152: Total number of sequences bypassed to SSW by TM and TS when processing DLH dataset.

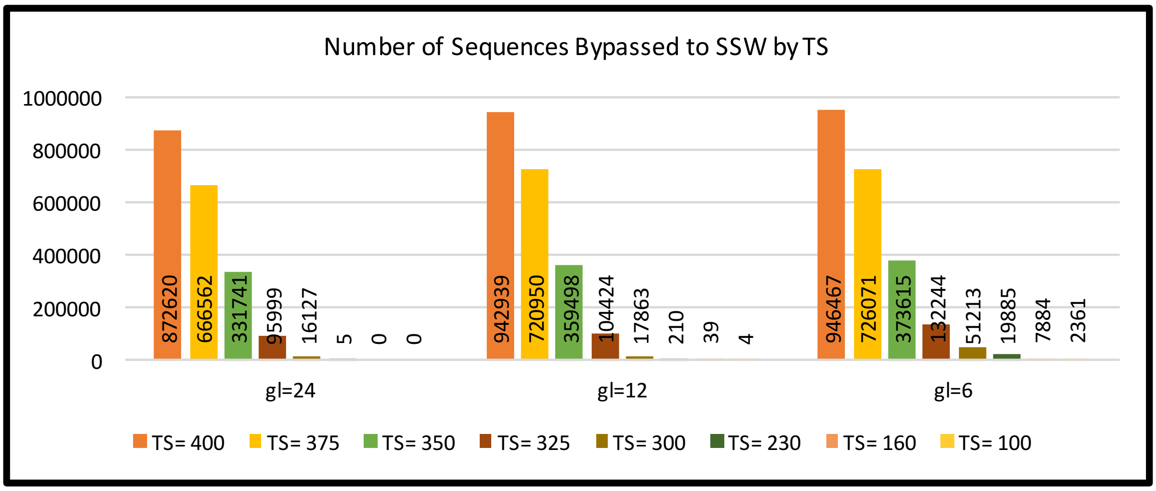

Figure 153: Total number of sequences bypassed to SSW by TS when processing DLH dataset.

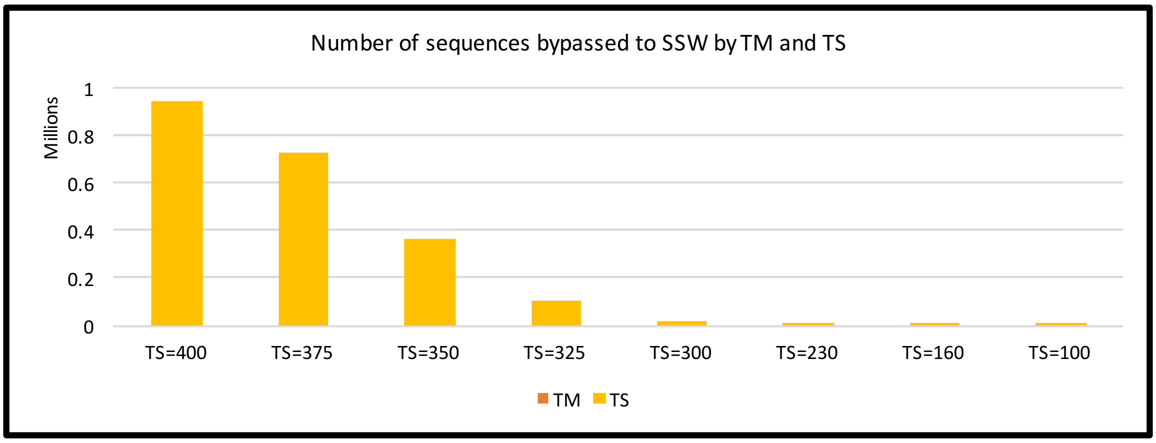

Figure 154: Proportion of input sequence pairs bypassed to SSW by TM and TS when processing DLH dataset.

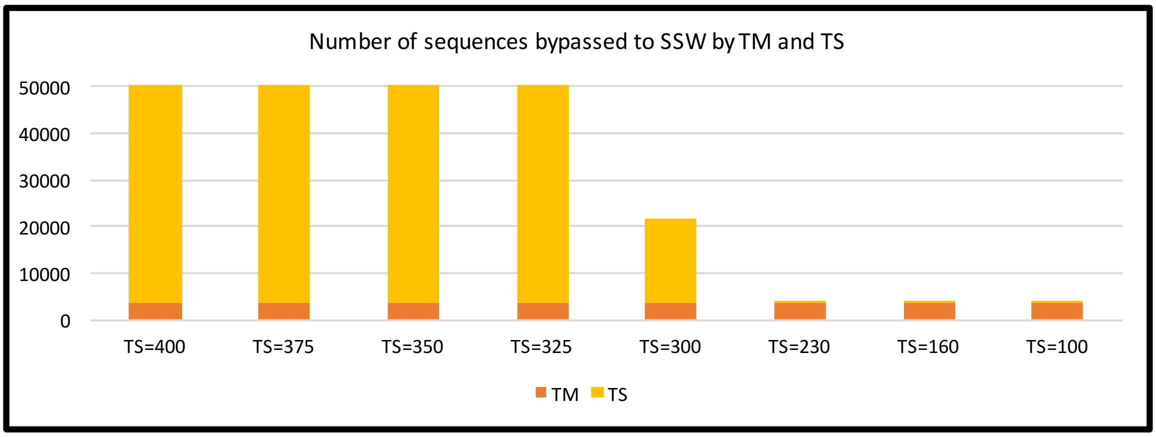

Figure 155: Proportion of input sequence pairs bypassed to SSW by TM and TS (scaled) when processing DLH dataset.

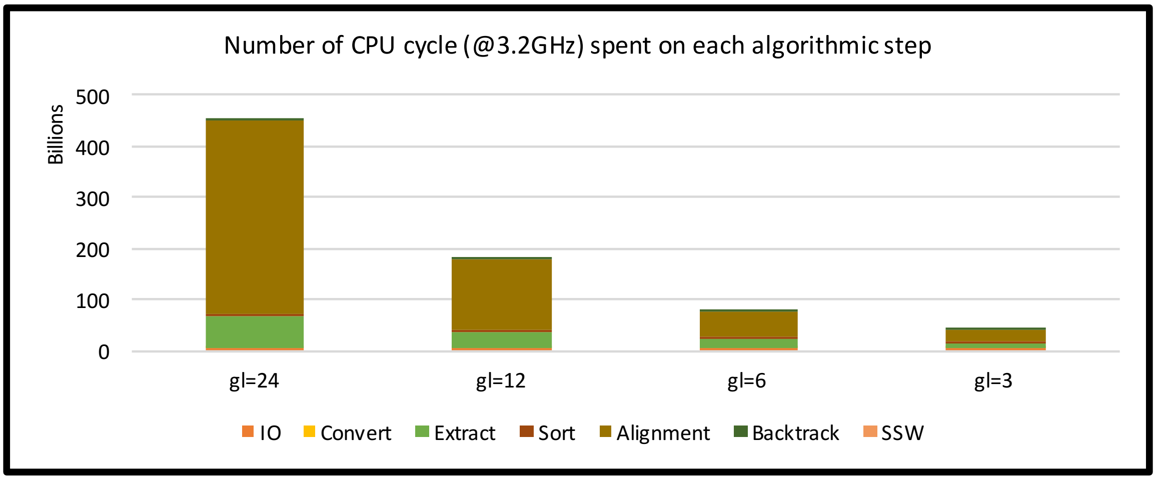

Figure 156: Cycle accurate execution time (DLH dataset) of differing algorithmic steps of *MEM-Align* when *gl* varies (in CPU cycle).

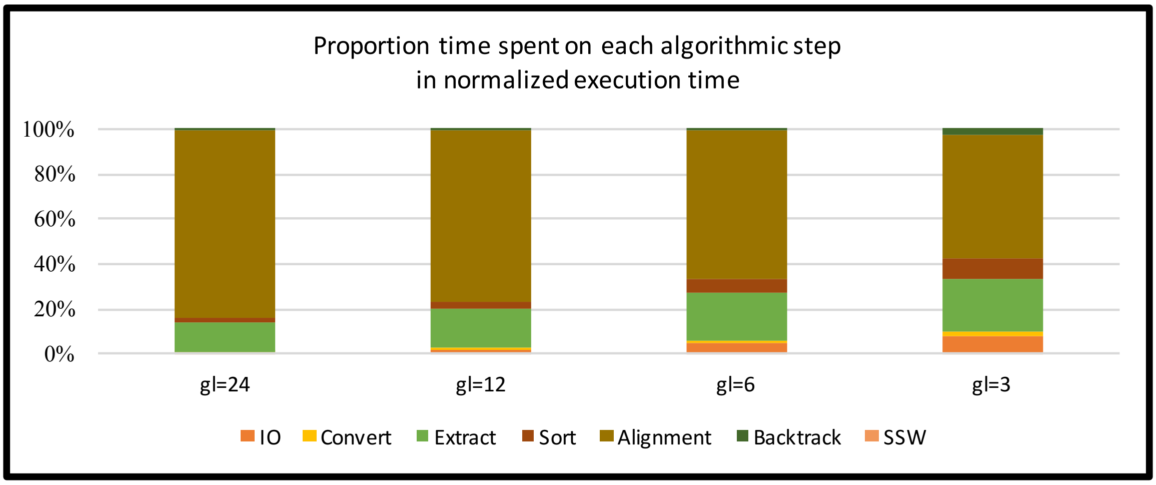

Figure 157: normalised cycle accurate execution time (DLH dataset) of differing algorithmic steps of *MEM-Align* when *gl* varies.

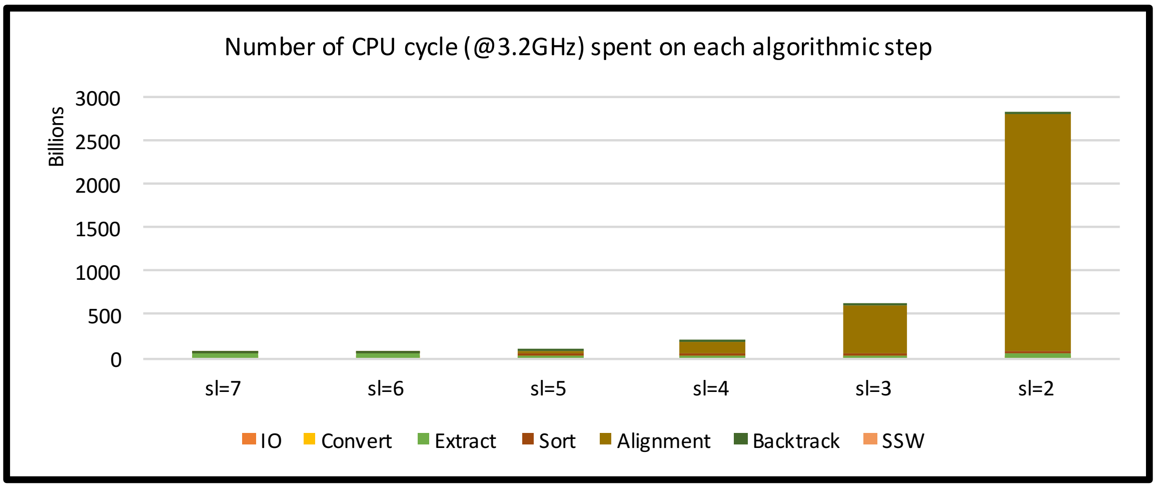

Figure 158: Cycle accurate execution time (DLH dataset) of differing algorithmic steps of *MEM-Align* when *sl* varies (in CPU cycle).

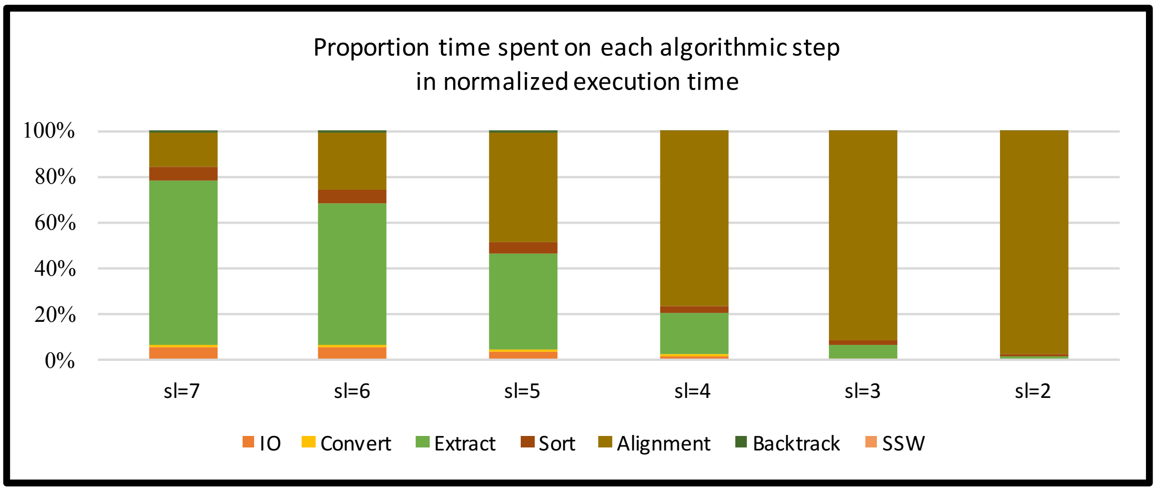

Figure 159: normalised cycle accurate execution time (DLH dataset) of differing algorithmic steps of *MEM-Align* when *sl* varies.

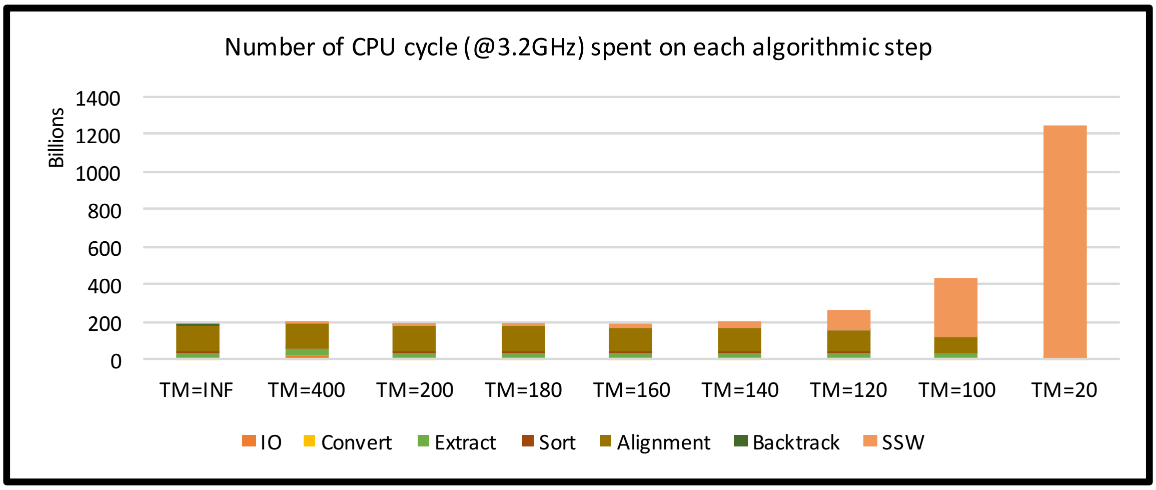

Figure 160: Cycle accurate execution time (DLH dataset) of differing algorithmic steps of *MEM-Align* when TM varies (in CPU cycle).

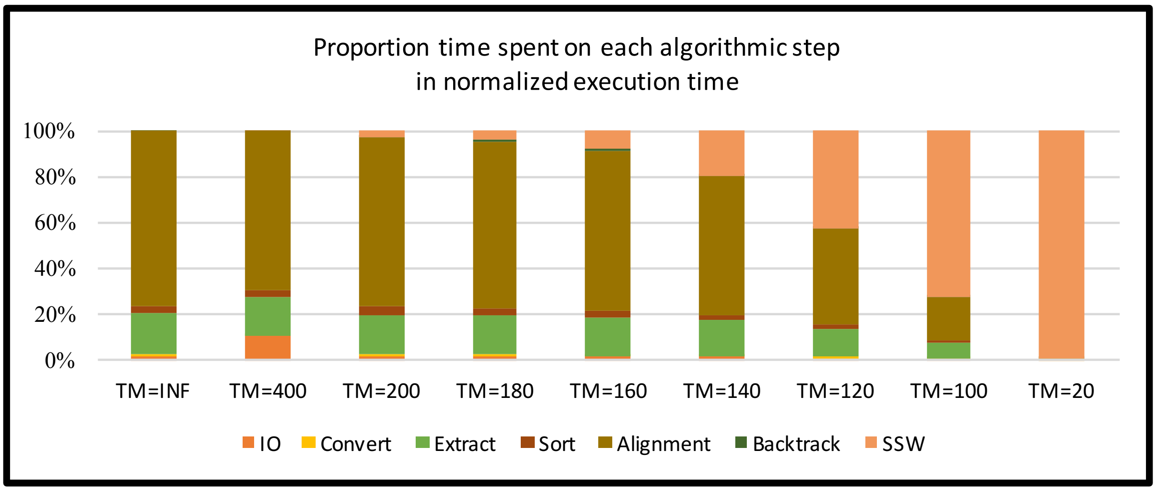

Figure 161: normalised cycle accurate execution time (DLH dataset) of differing algorithmic steps of *MEM-Align* when TM varies.

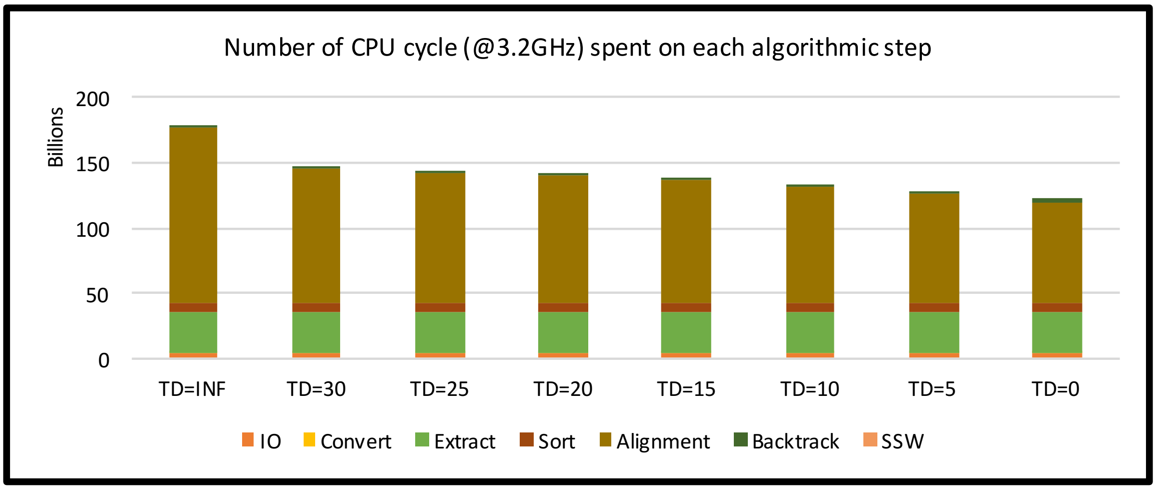

Figure 162: Cycle accurate execution time (DLH dataset) of differing algorithmic steps of *MEM-Align* when TD varies (in CPU cycle).

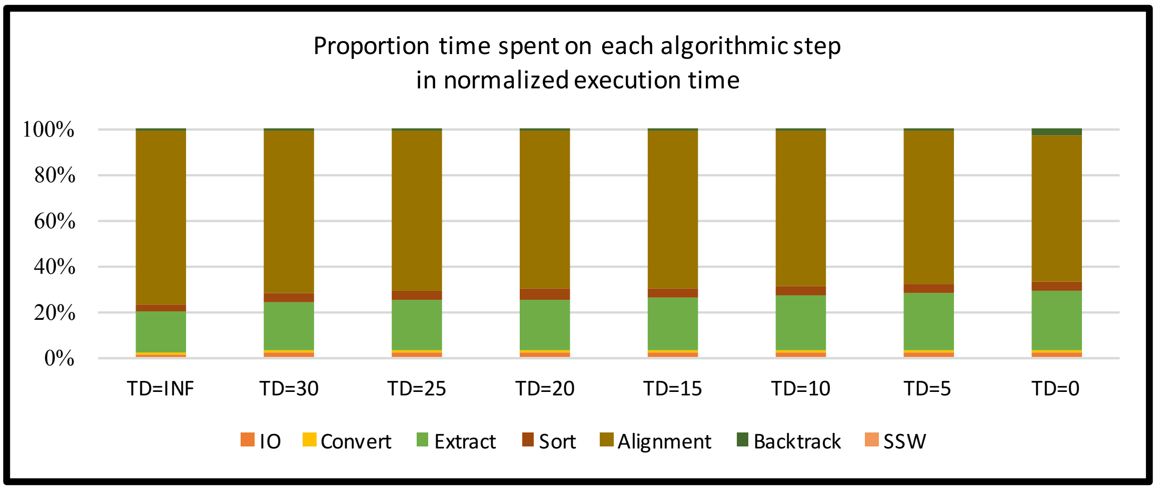

Figure 163: normalised cycle accurate execution time (DLH dataset) of differing algorithmic steps of *MEM-Align* when TD varies.

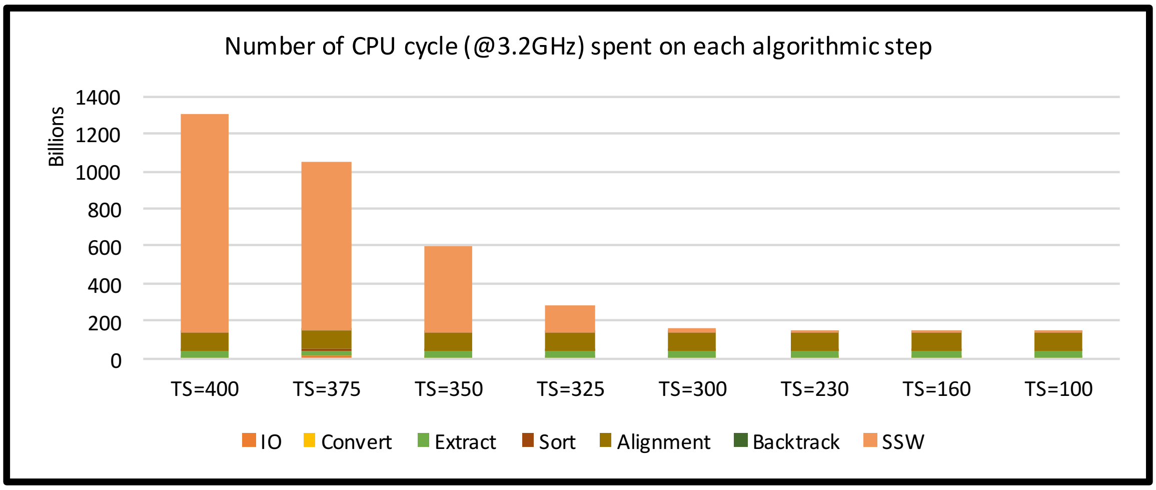

Figure 164: Cycle accurate execution time (DLH dataset) of differing algorithmic steps of *MEM-Align* when TS varies (in CPU cycle).

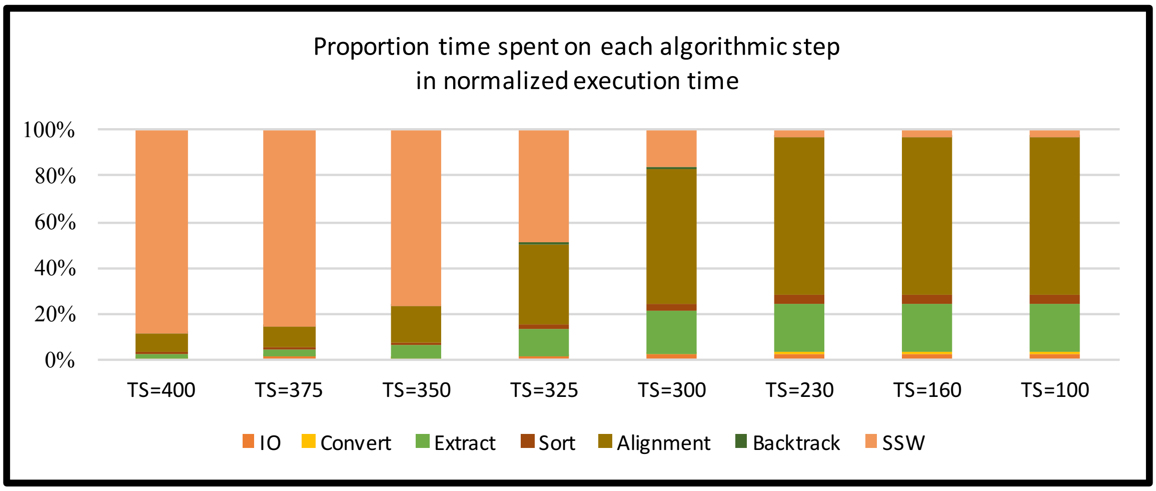

Figure 165: normalised cycle accurate execution time (DLH dataset) of differing algorithmic steps of *MEM-Align* when TS varies.

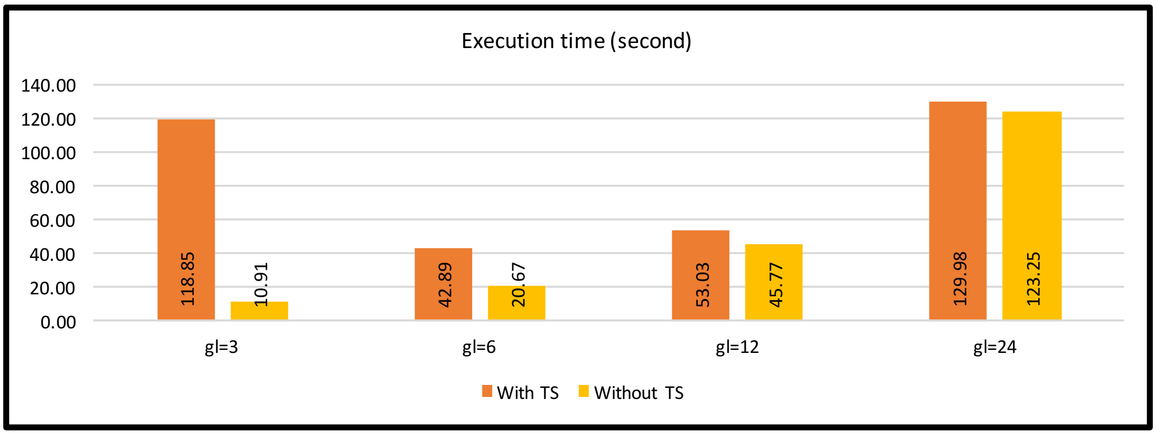

Figure 166: Execution time (DLH dataset) for differing gl with and without TS.

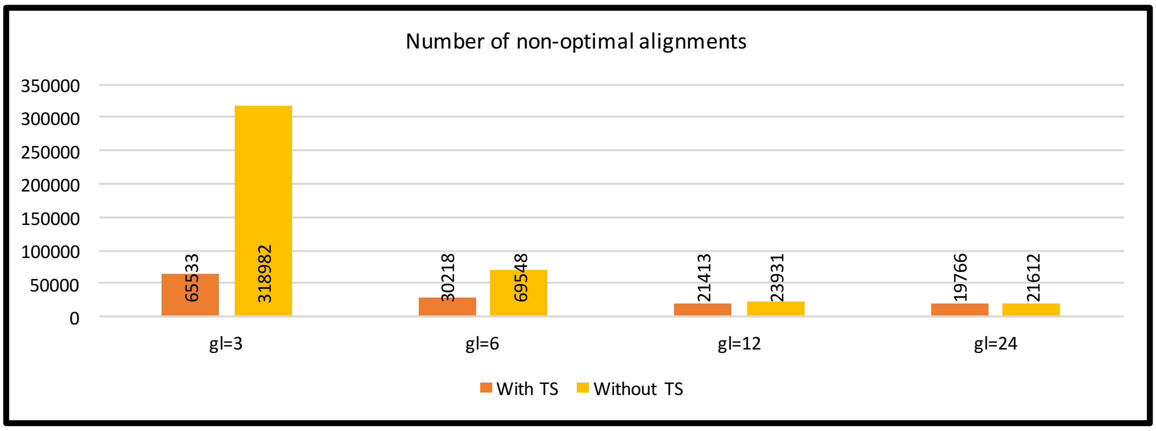

Figure 167: Number of suboptimal alignments (DLH dataset) for differing gl with and without TS.

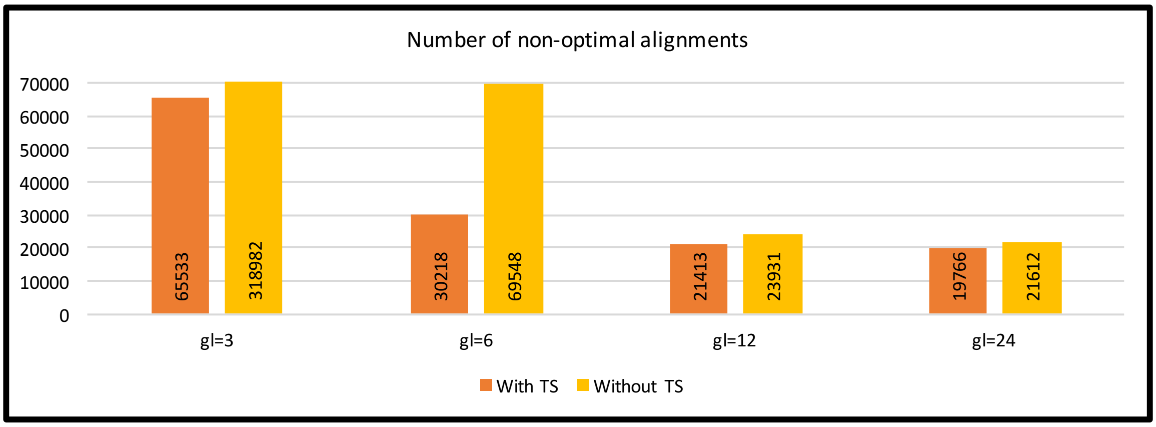

Figure 168: Number of suboptimal alignments (DLH dataset) for differing gl with and without TS (scaled).

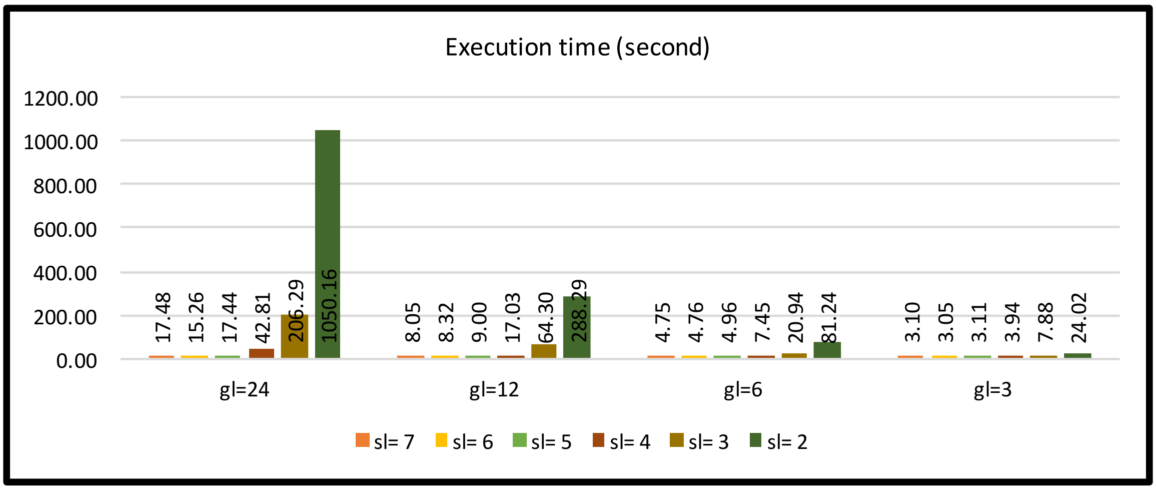

Figure 169: Execution times for DRQ dataset when *gl* and *sl* varies.

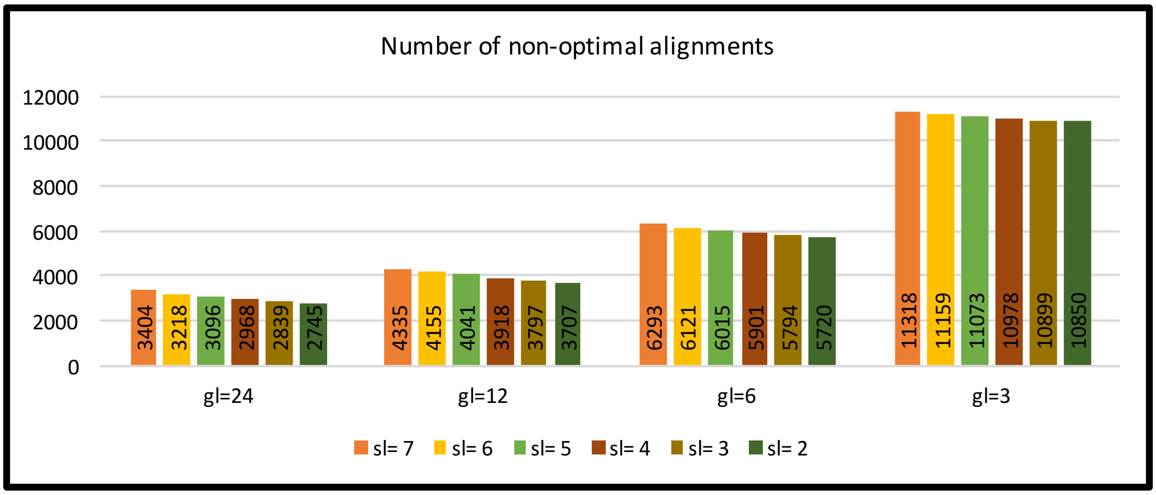

Figure 170: Number of suboptimal alignments for DRQ dataset when *gl* and *sl* varies.

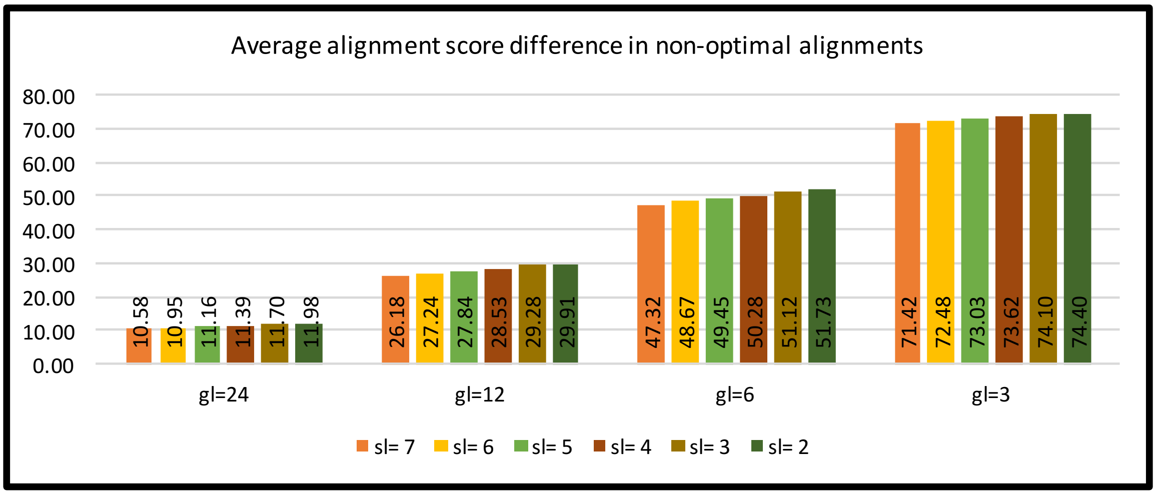

Figure 171: Average alignment score difference in suboptimal alignments for DRQ dataset when *gl* and *sl* varies.

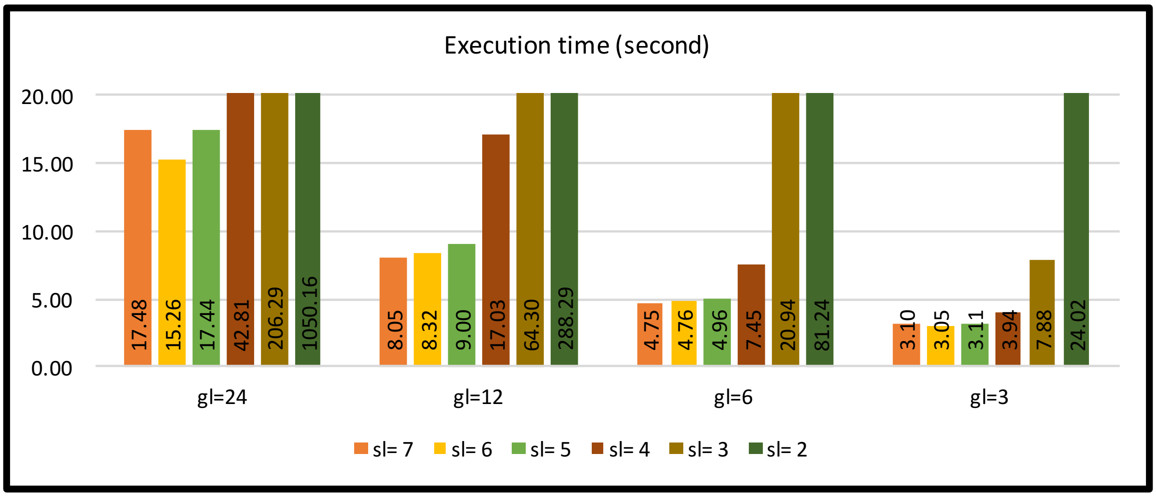

Figure 172: Execution times for DRQ dataset when *gl* and *sl* varies (scaled).

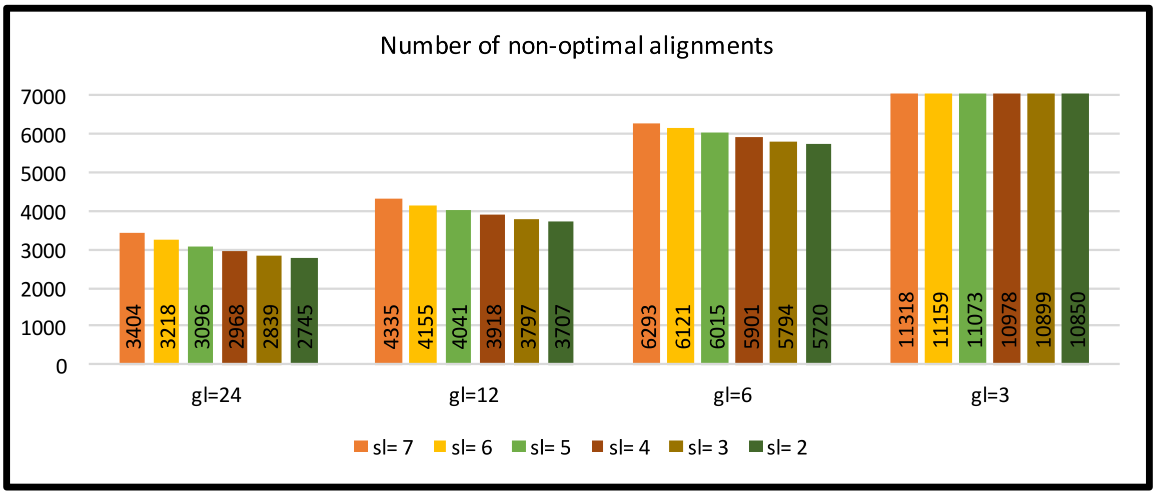

Figure 173: Number of suboptimal alignments for DRQ dataset when *gl* and *sl* varies (scaled).

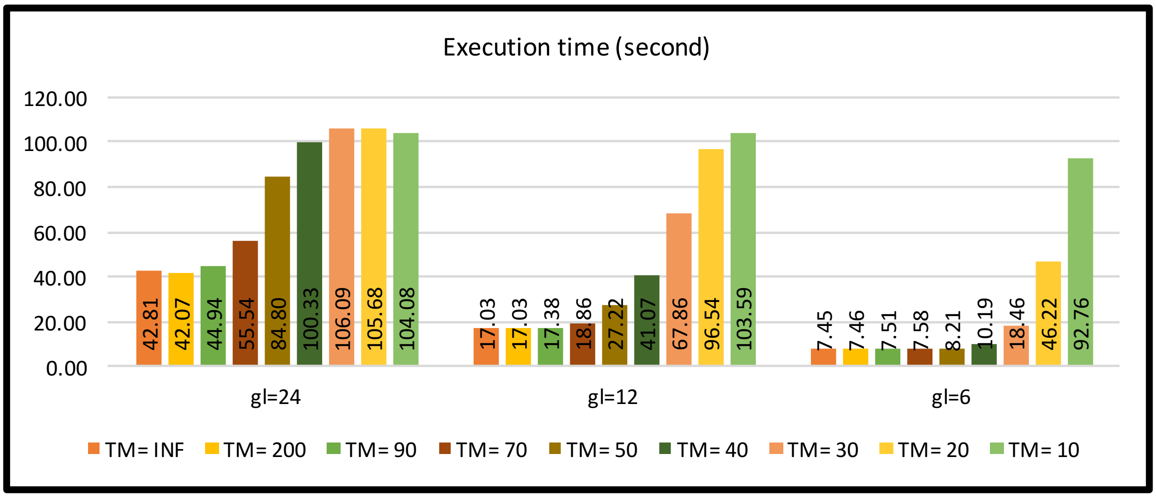

Figure 174: Execution times for DRQ dataset when TM varies.

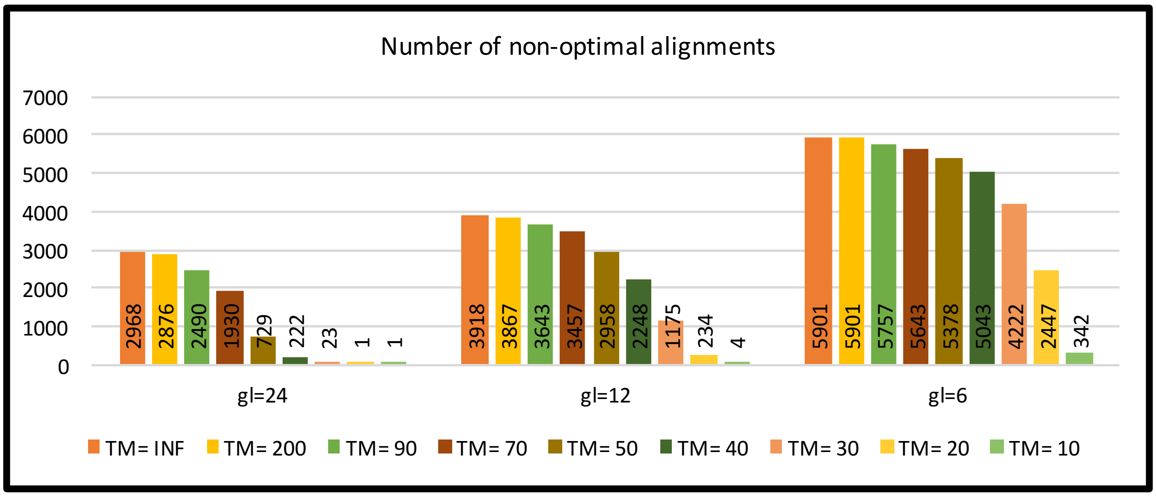

Figure 175: Number of suboptimal alignments for DRQ dataset when TM varies.

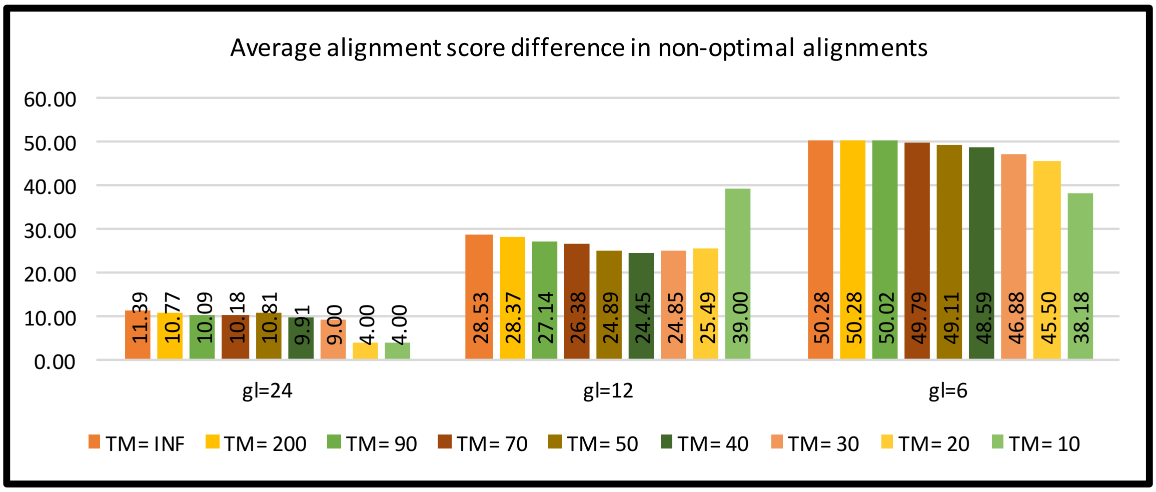

Figure 176: Average alignment score difference in suboptimal alignments for DRQ dataset when TM varies.

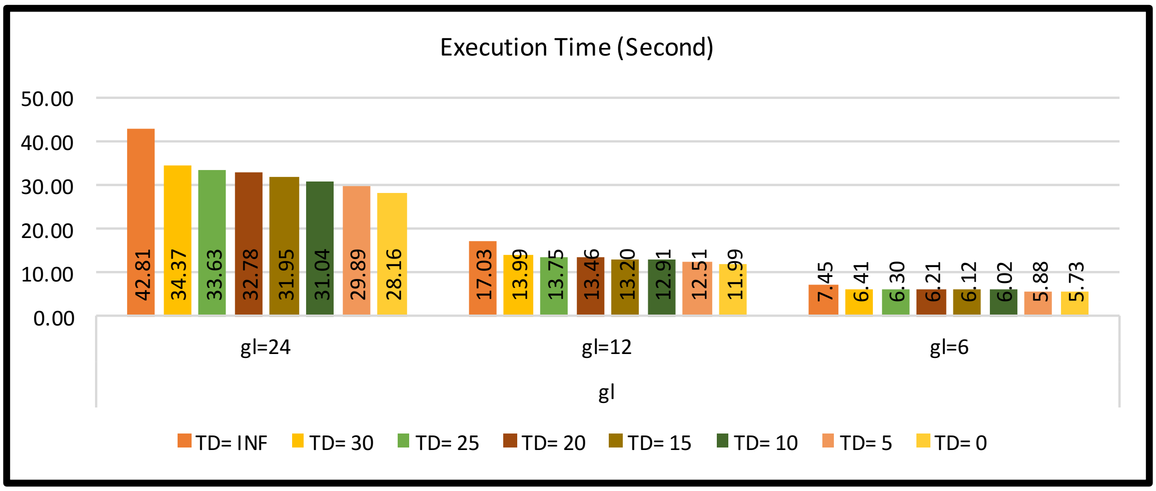

Figure 177: Execution times for DRQ dataset when TD varies.

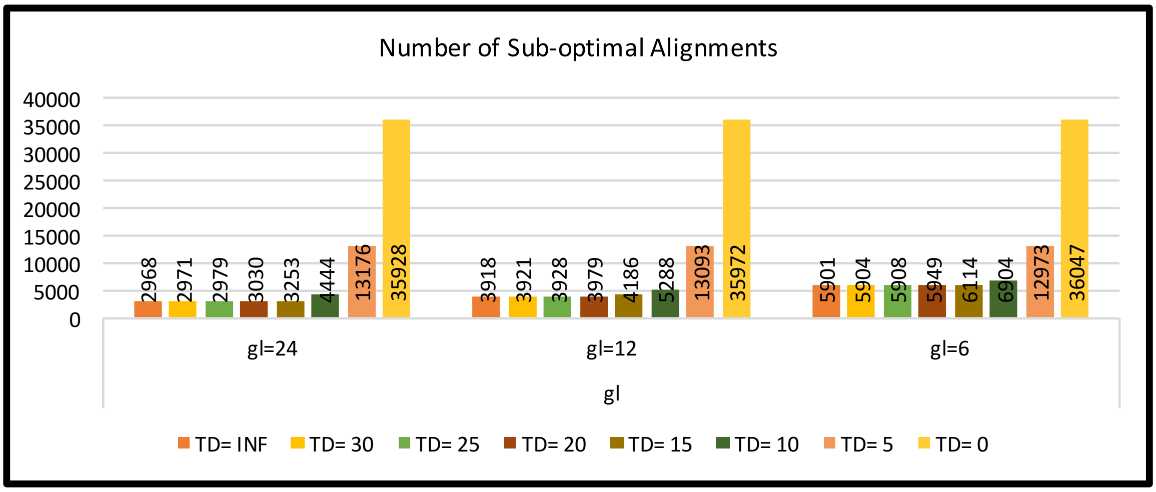

Figure 178: Number of suboptimal alignments for DRQ dataset when TD varies.

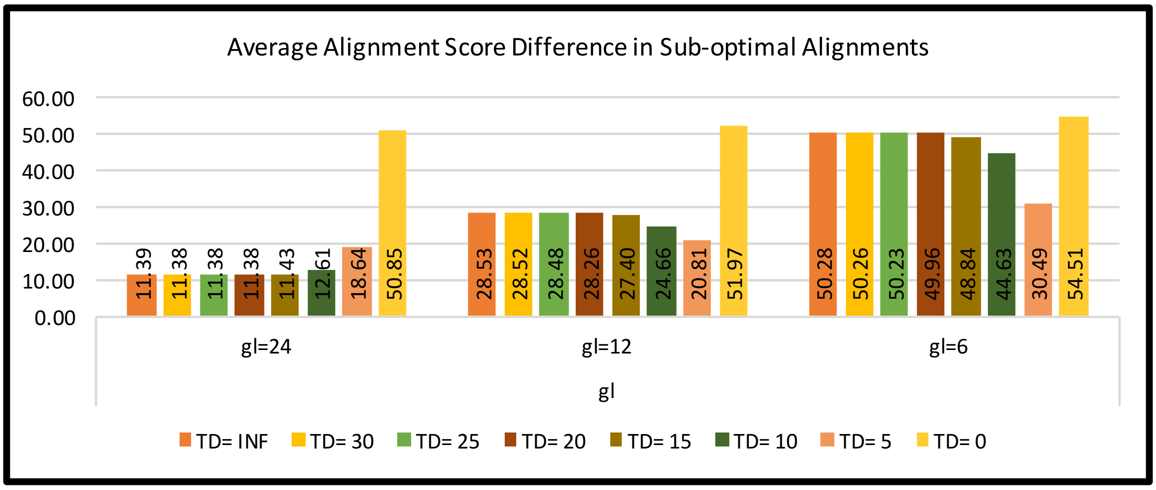

Figure 179: Average alignment score difference in suboptimal alignments for DRQ dataset when TM varies.

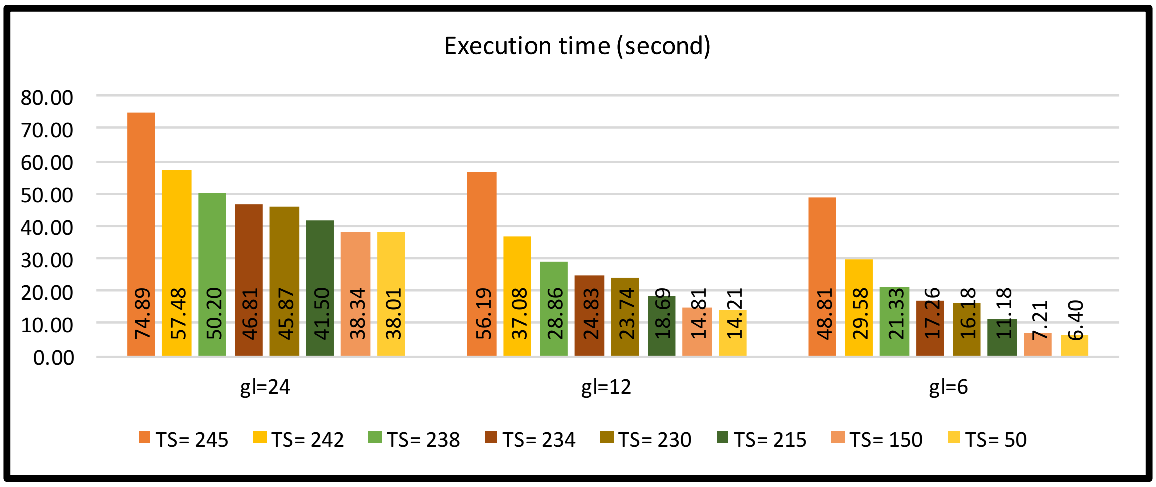

Figure 180: Execution times for DRQ dataset when TS varies.

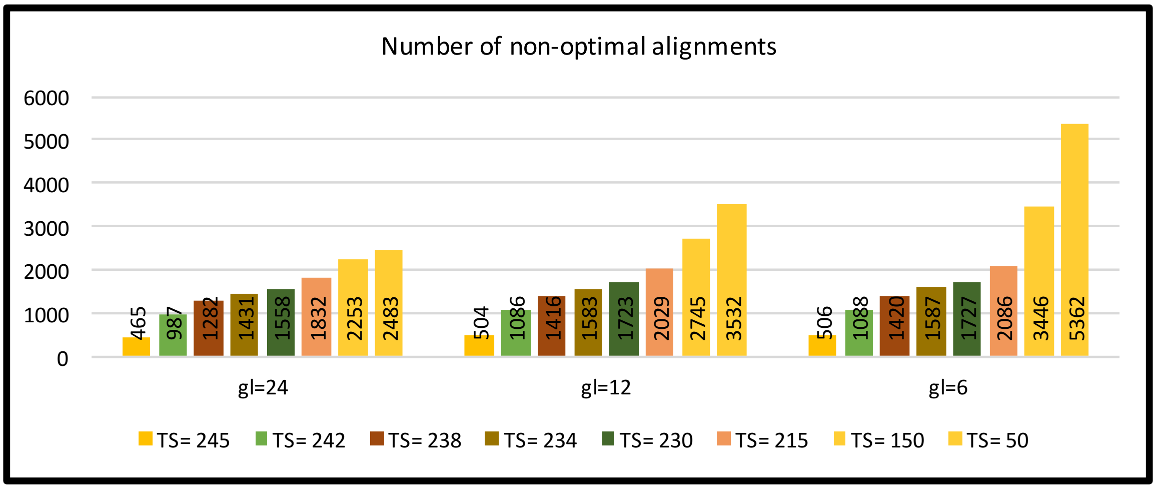

Figure 181: Number of suboptimal alignments for DRQ dataset when TS varies.

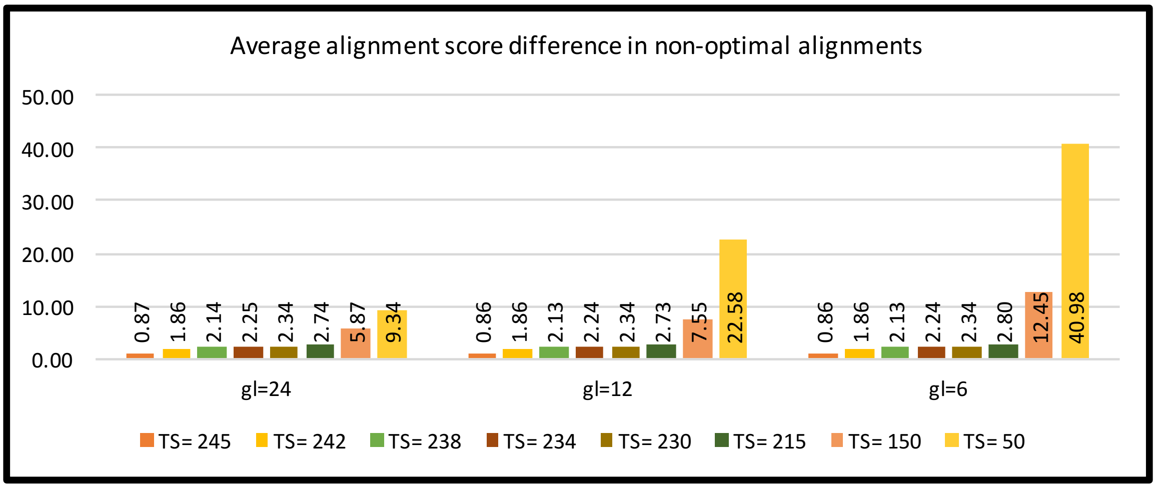

Figure 182: Average alignment score difference in suboptimal alignments for DRQ dataset when TS varies.

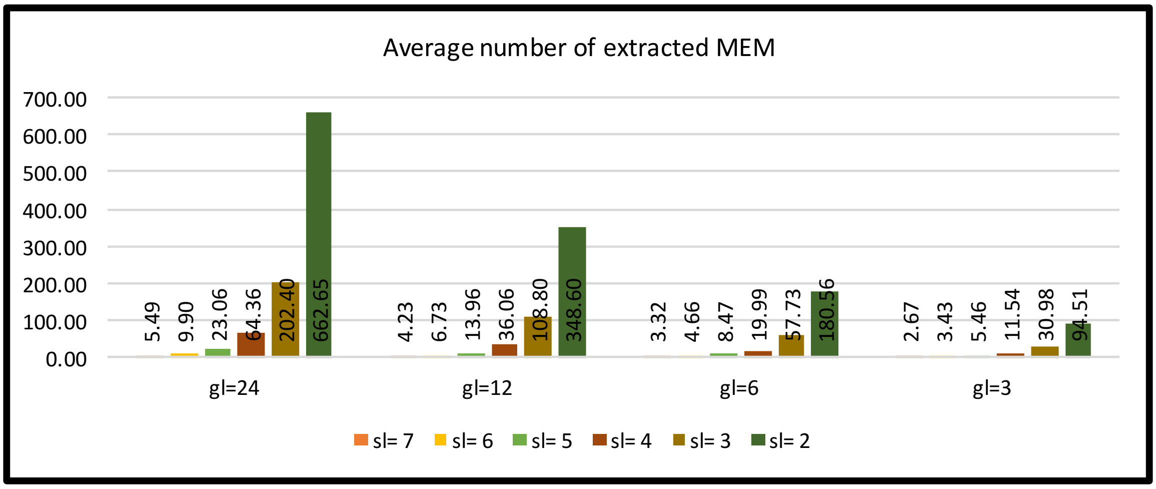

Figure 183: Average number of extracted MEM for DRQ dataset when gl and sl varies.

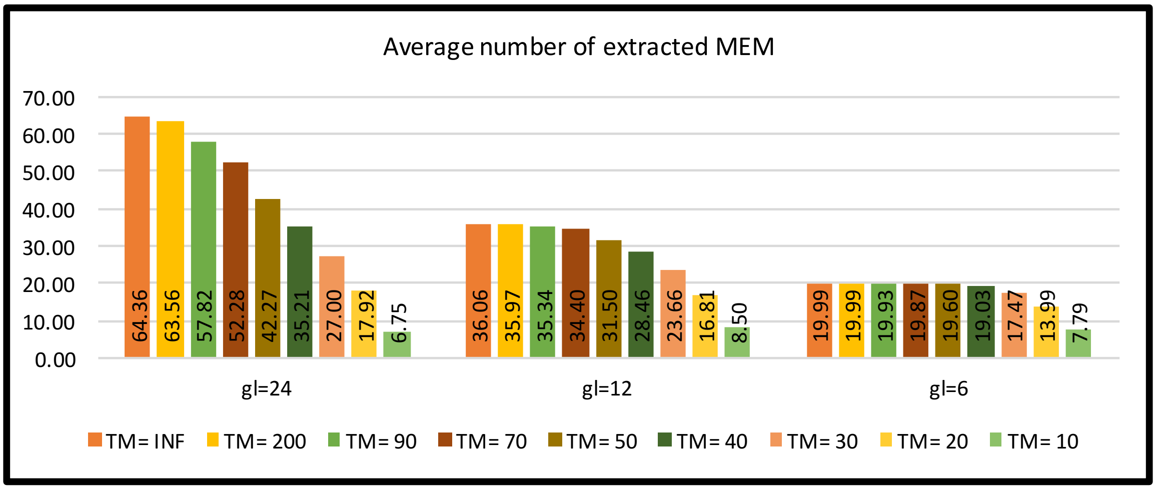

Figure 184: Average number of extracted MEM for DRQ dataset when TM varies.

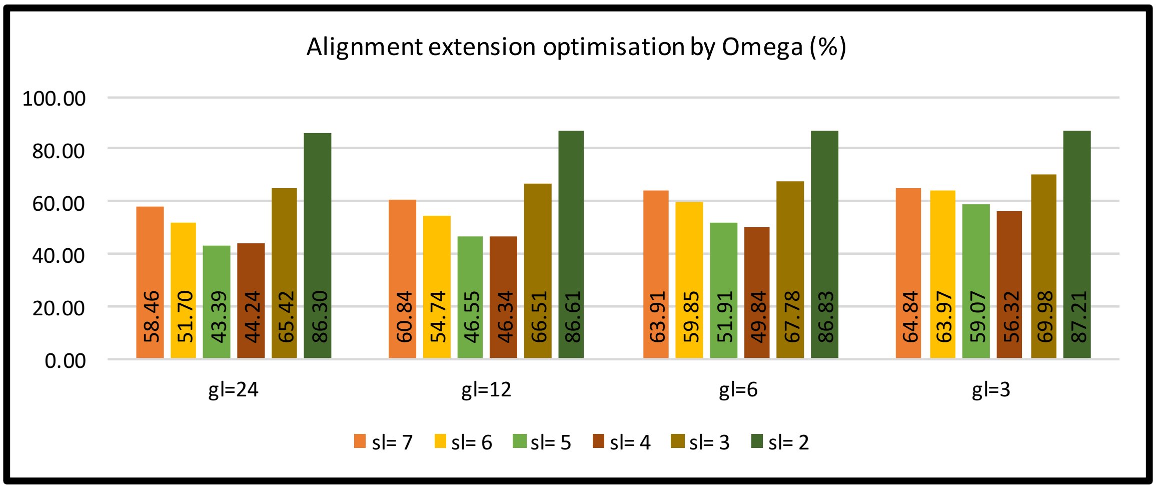

Figure 185: Proportion of alignment extension which are optimised (avoided) by the set Ω when processing DRQ dataset.

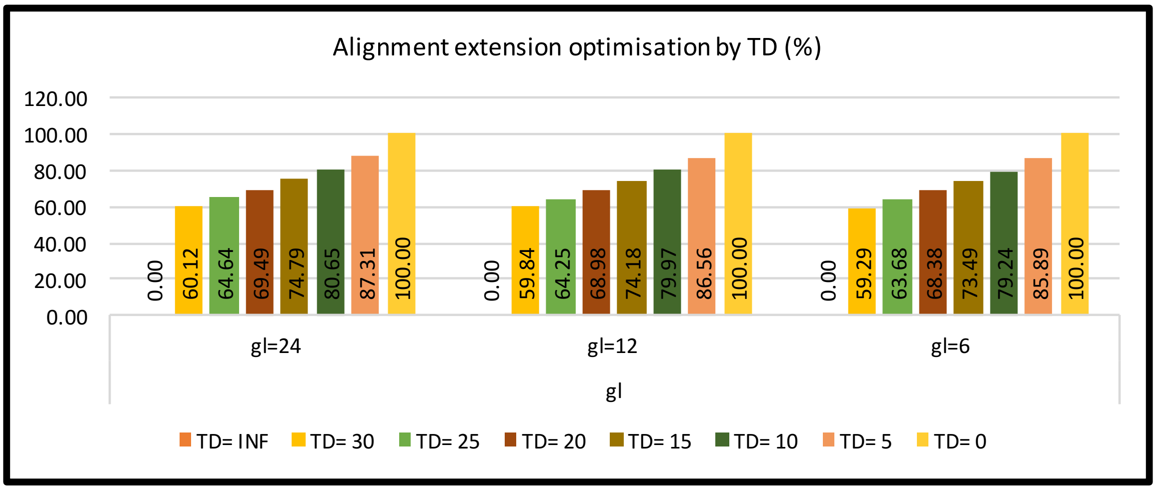

Figure 186: Proportion of alignment extension which are optimised (avoided) by TD after applying the set Ω when processing DRQ dataset.

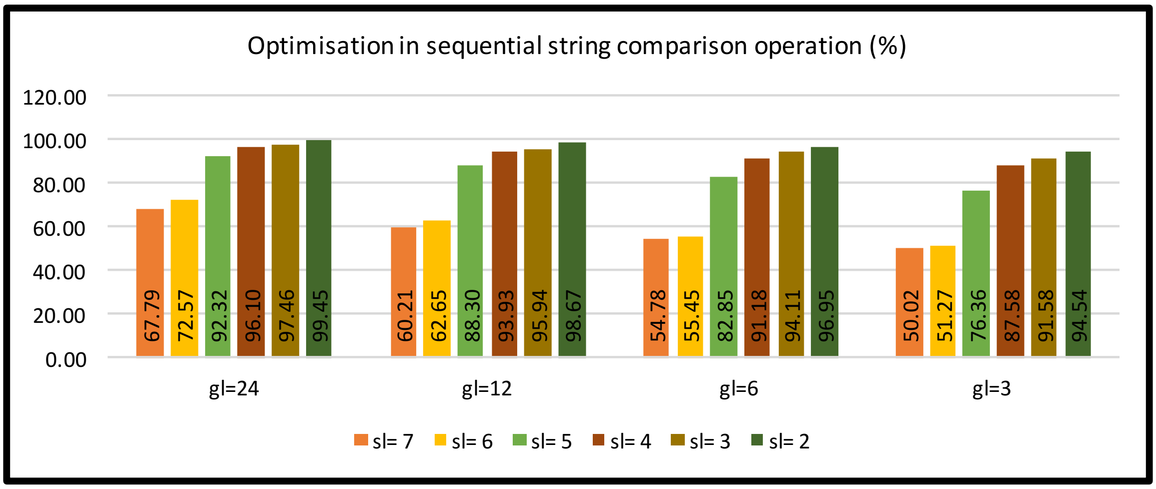

Figure 187: Proportion of sequential string compare operation which are optimised (avoided) when processing DRQ dataset.

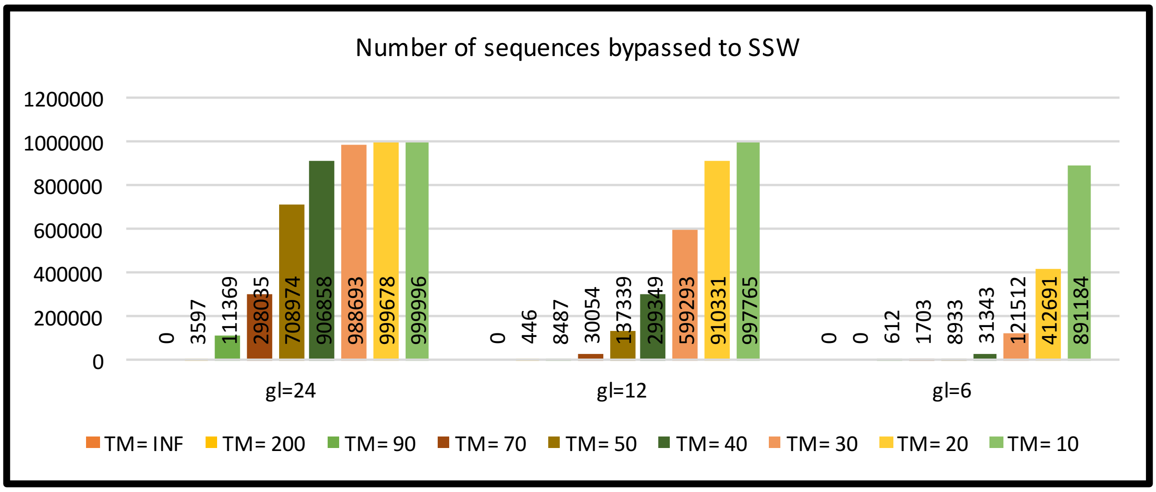

Figure 188: Number of sequences bypassed to SSW by TM (TS has not been applied) when processing DRQ dataset.

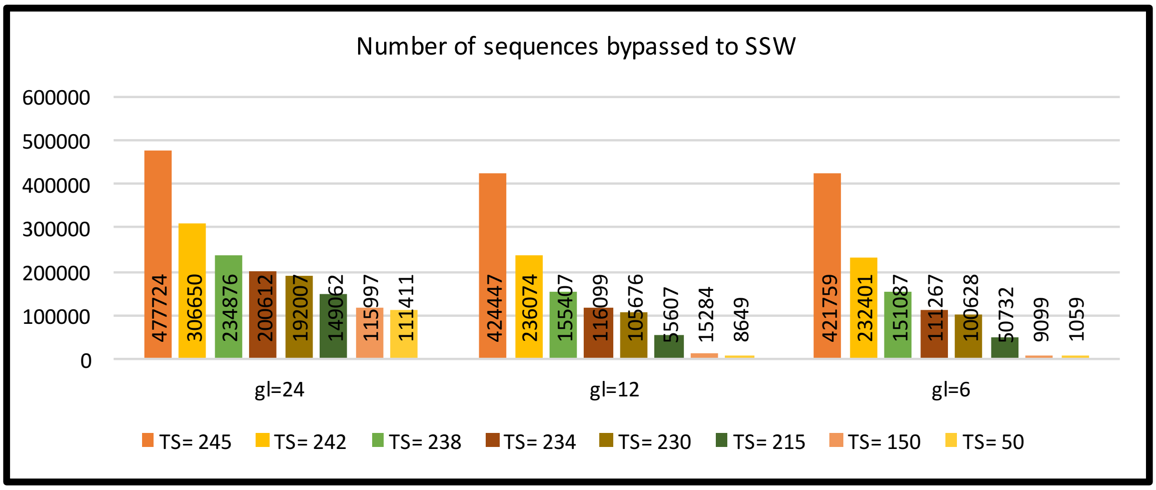

Figure 189: Total number of sequences bypassed to SSW by TM and TS when processing DRQ dataset.

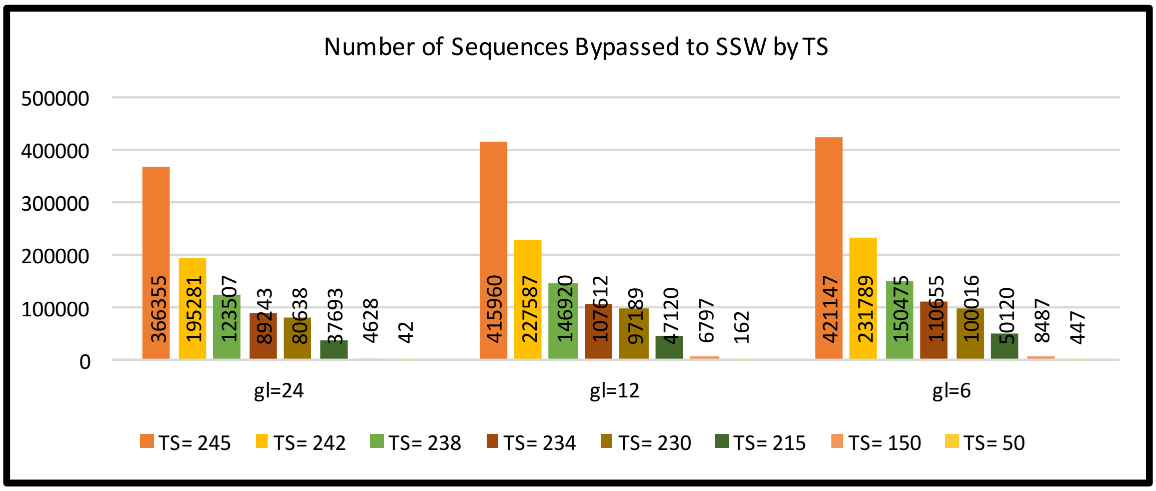

Figure 190: Total number of sequences bypassed to SSW by TS when processing DRQ dataset.

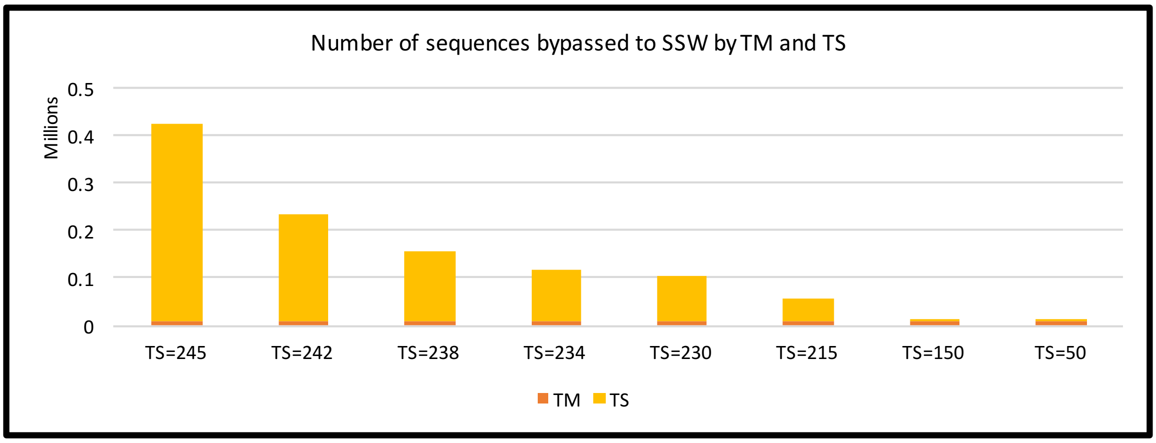

Figure 191: Proportion of input sequence pairs bypassed to SSW by TM and TS when processing DRQ dataset.

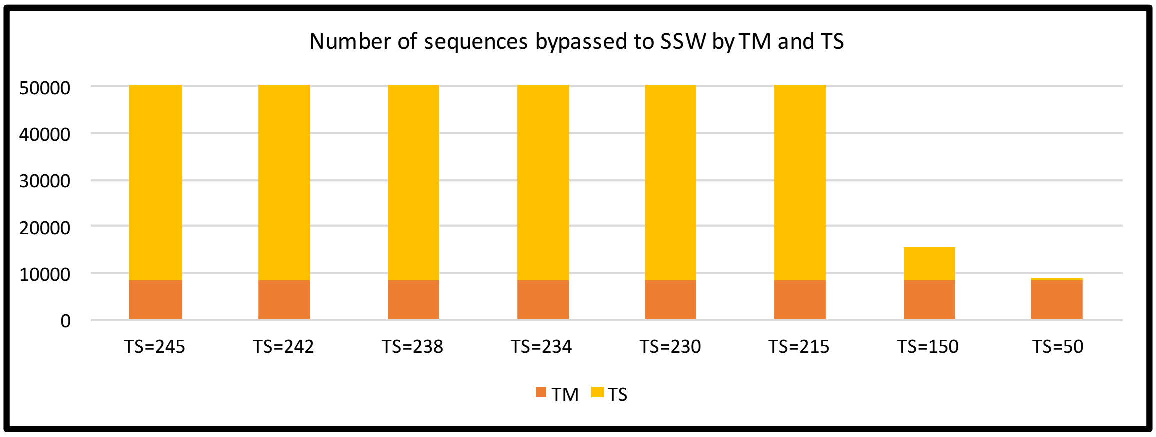

Figure 192: Proportion of input sequence pairs bypassed to SSW by TM and TS (scaled) when processing DRQ dataset.

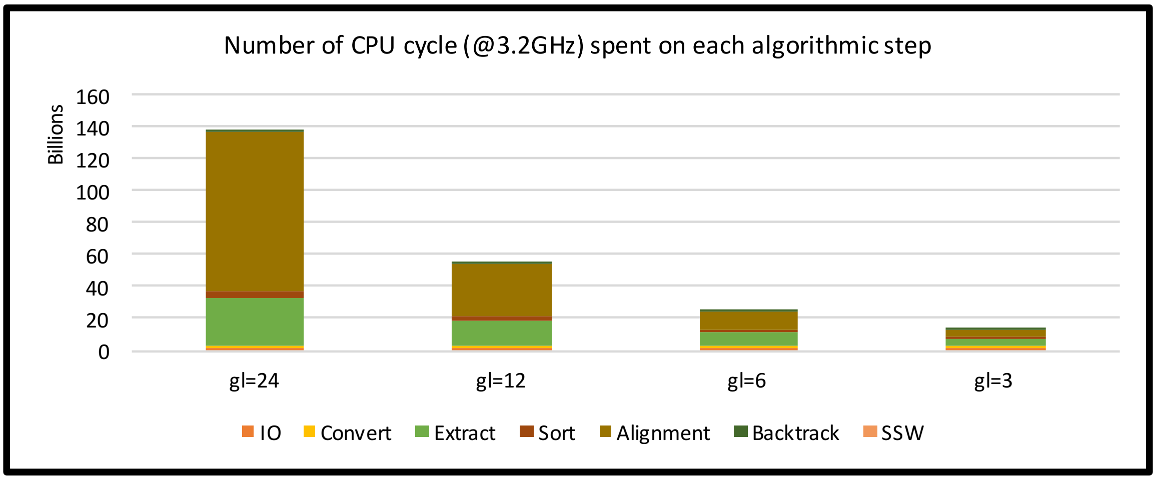

Figure 193: Cycle accurate execution time (DRQ dataset) of differing algorithmic steps of *MEM-Align* when *gl* varies (in CPU cycle).

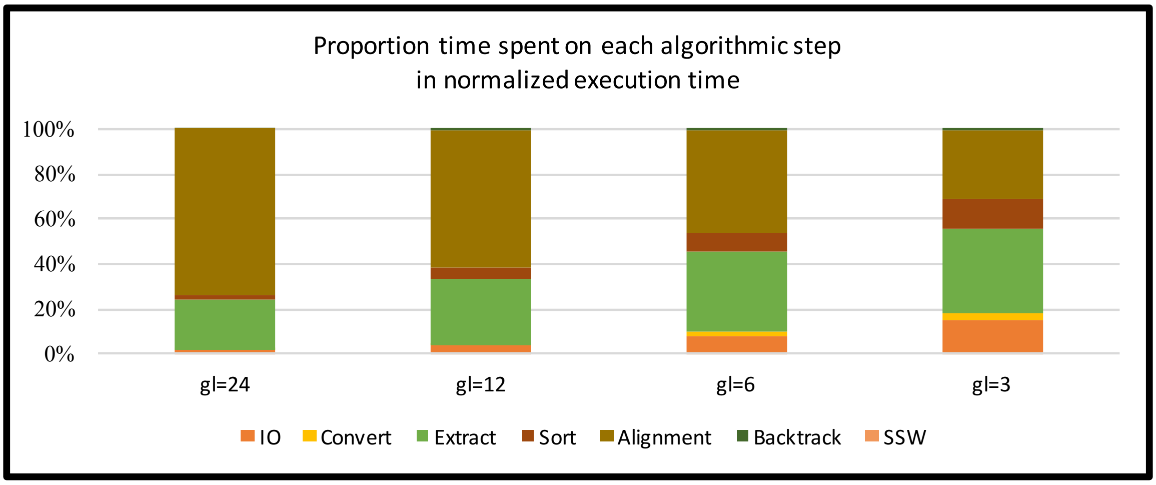

Figure 194: normalised cycle accurate execution time (DRQ dataset) of differing algorithmic steps of *MEM-Align* when *gl* varies.

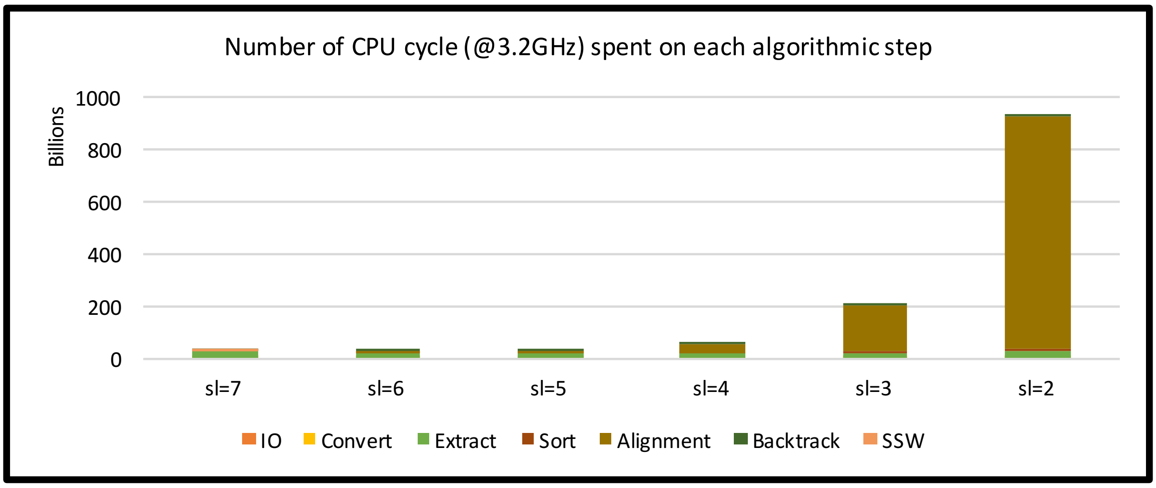

Figure 195: Cycle accurate execution time (DRQ dataset) of differing algorithmic steps of *MEM-Align* when *sl* varies (in CPU cycle).

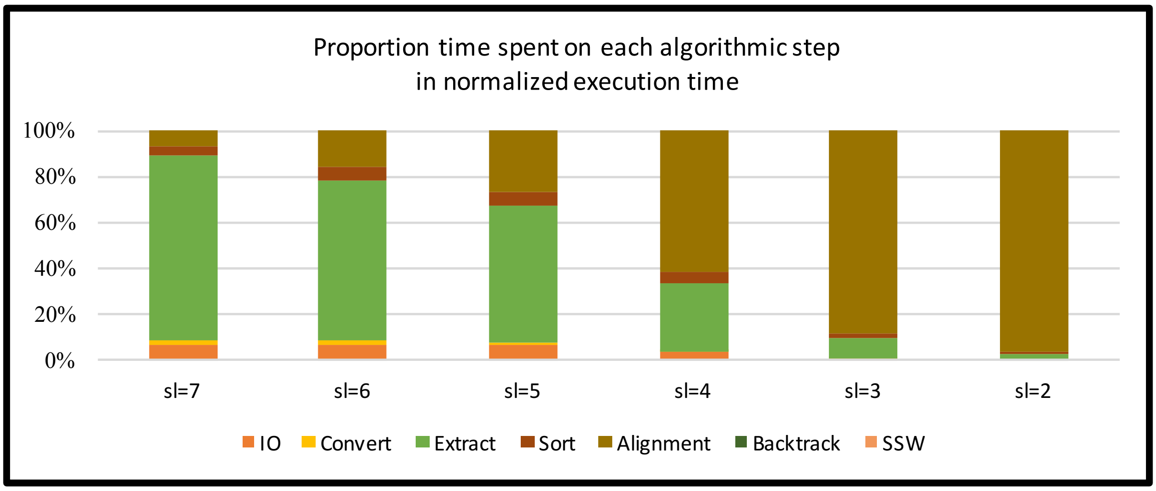

Figure 196: normalised cycle accurate execution time (DRQ dataset) of differing algorithmic steps of *MEM-Align* when *sl* varies.

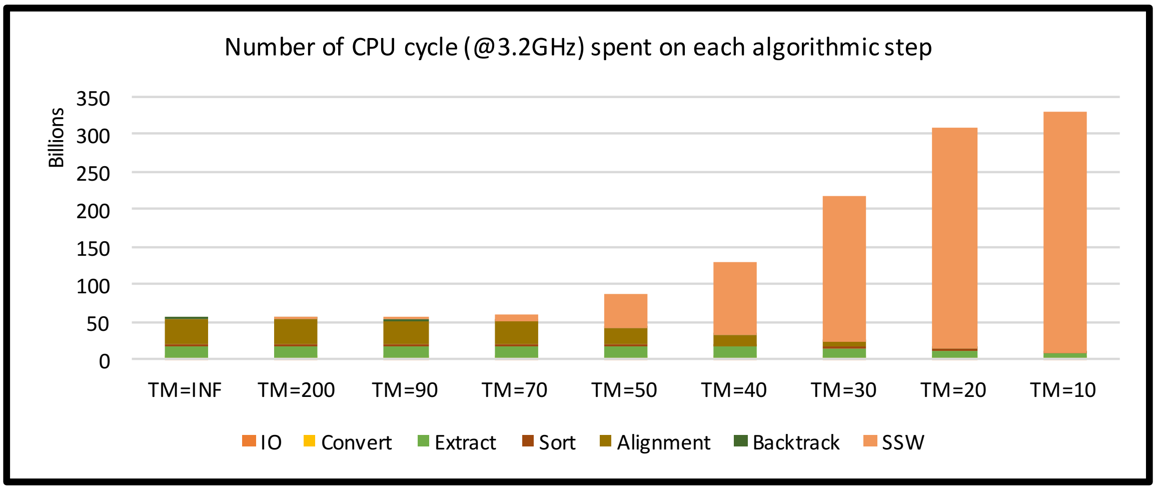

Figure 197: Cycle accurate execution time (DRQ dataset) of differing algorithmic steps of *MEM-Align* when TM varies (in CPU cycle).

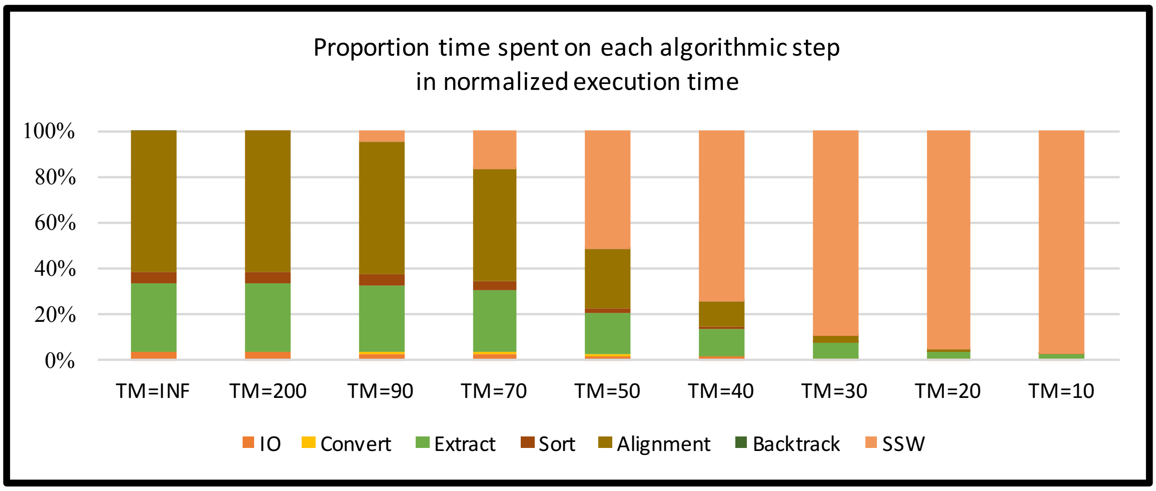

Figure 198: normalised cycle accurate execution time (DRQ dataset) of differing algorithmic steps of *MEM-Align* when TM varies.

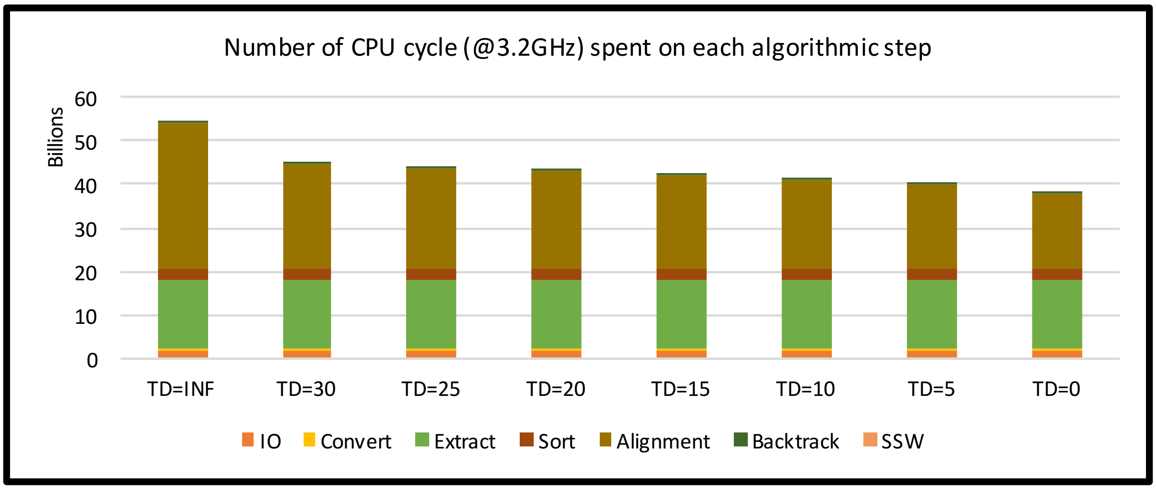

Figure 199: Cycle accurate execution time (DRQ dataset) of differing algorithmic steps of *MEM-Align* when TD varies (in CPU cycle).

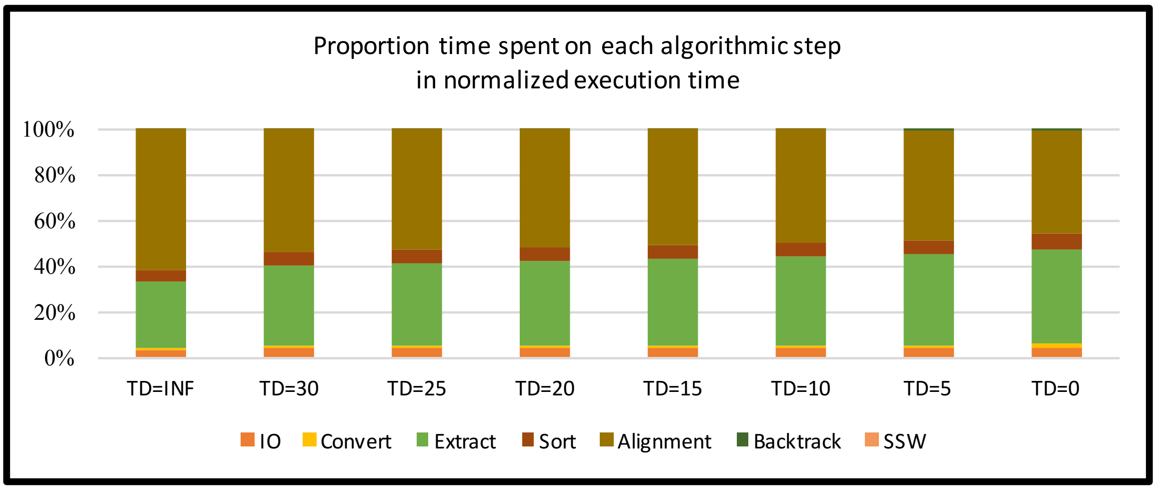

Figure 200: normalised cycle accurate execution time (DRQ dataset) of differing algorithmic steps of *MEM-Align* when TD varies.

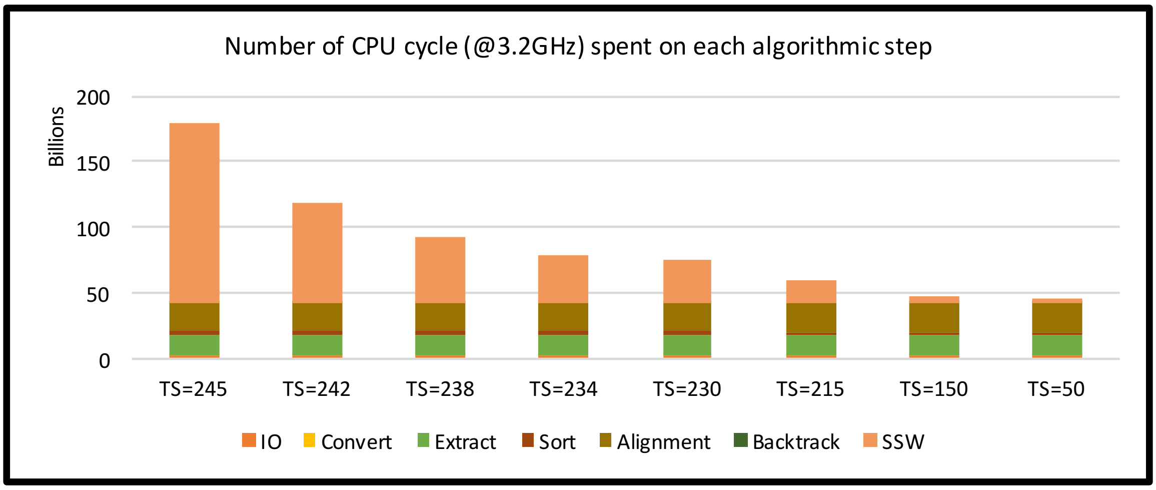

Figure 201: Cycle accurate execution time (DRQ dataset) of differing algorithmic steps of *MEM-Align* when TS varies (in CPU cycle).

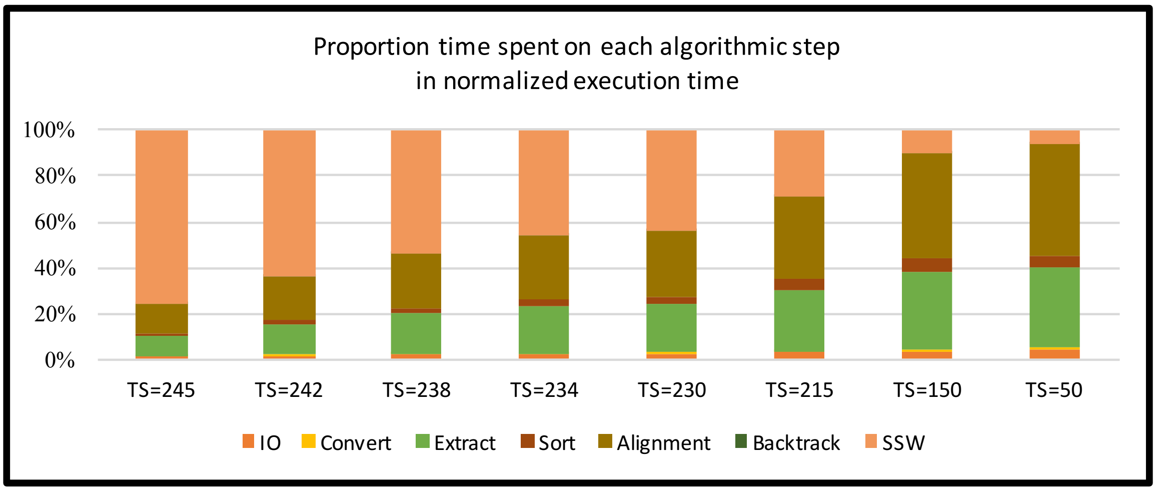

Figure 202: normalised cycle accurate execution time (DRQ dataset) of differing algorithmic steps of *MEM-Align* when TS varies.

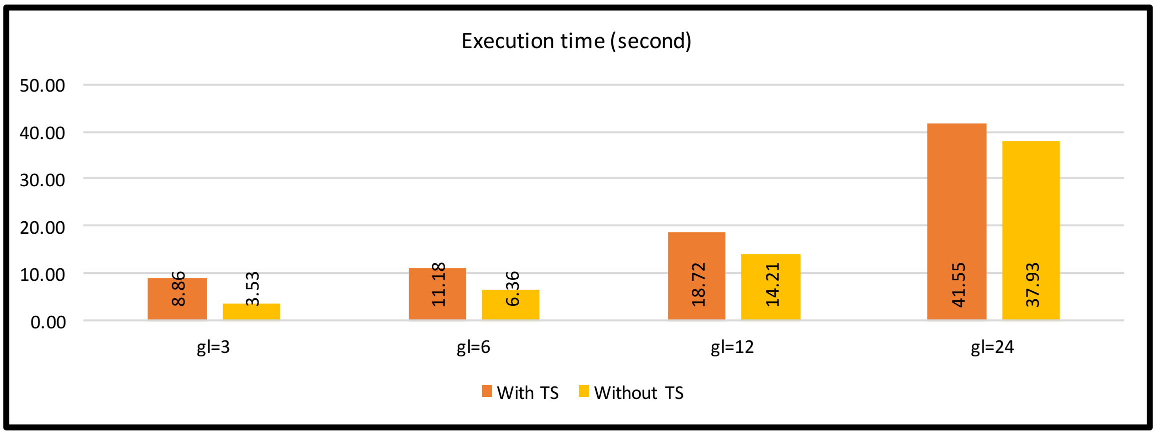

Figure 203: Execution time (DRQ dataset) for differing gl with and without TS.

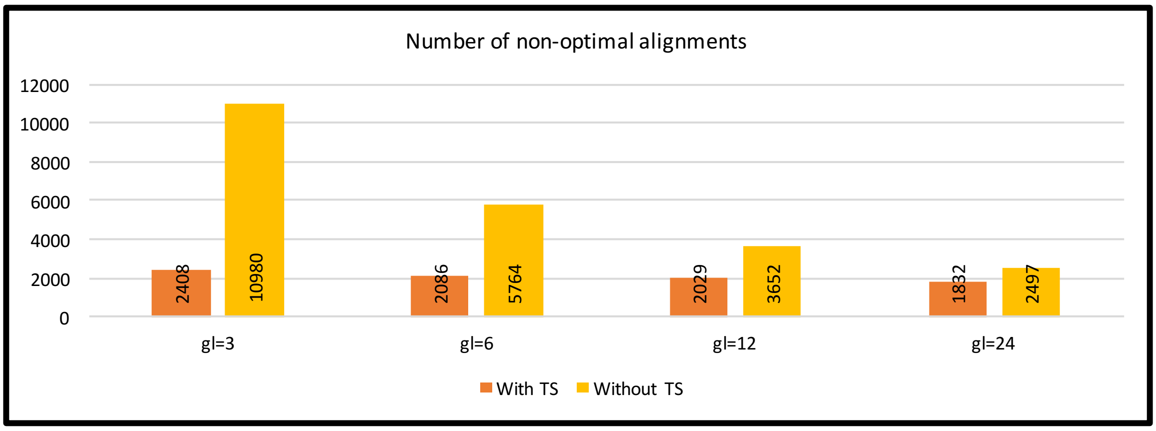

Figure 204: Number of suboptimal alignments (DRQ dataset) for differing gl with and without TS.

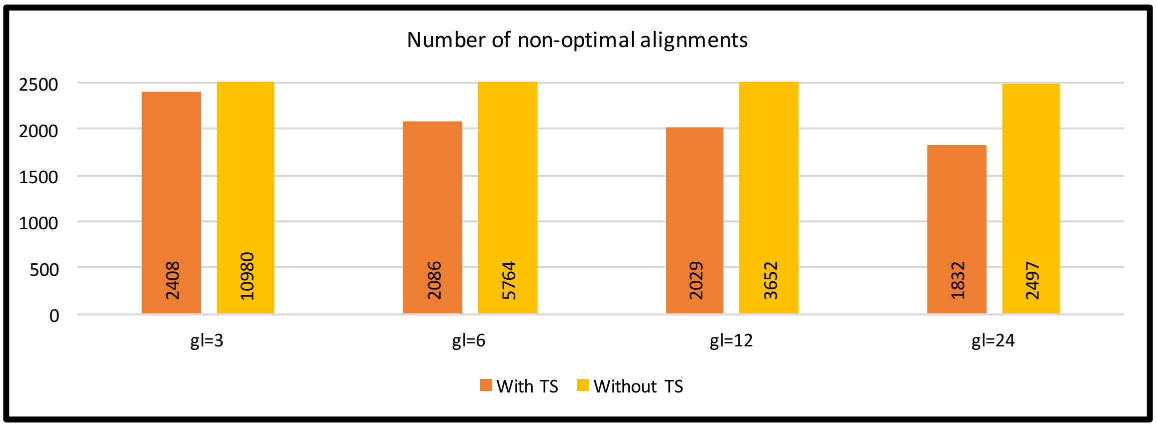

Figure 205: Number of suboptimal alignments (DRQ dataset) for differing gl with and without TS (scaled).

References

[1] Rosenberg, M.S.: Sequence Alignment: Methods, Models, Concepts, and Strategies. Univ of California Press, ??? (2009)

[2] Li, H., Durbin, R.: Fast and accurate short read alignment with Burrows-Wheeler transform. Bioinformatics (Oxford, England) **25**(14), 1754–60 (2009)

[3] Li, H.: Aligning sequence reads, clone sequences and assembly contigs with BWA-MEM, 3 (2013)

[4] Langmead, B., Trapnell, C., Pop, M., Salzberg, S.L.: Ultrafast and memory-efficient alignment of short DNA sequences to the human genome. Genome biology **10**(3), 25 (2009)

[5] Langmead, B., Salzberg, S.L.: Fast gapped-read alignment with Bowtie 2. Nature methods **9**(4), 357–9 (2012)

[6] Altschul, S.F., Gish, W., Miller, W., Myers, E.W., Lipman, D.J.: Basic local alignment search tool. Journal of molecular biology **215**(3), 403–410 (1990)

[7] Kent, W.J.: Blatthe blast-like alignment tool. Genome research **12**(4), 656–664 (2002)

[8] Kurtz, S., Phillippy, A., Delcher, A.L., Smoot, M., Shumway, M., Antonescu, C., Salzberg, S.L.: Versatile and open software for comparing large genomes. Genome biology **5**(2), 12 (2004)

[9] Ma, B., Tromp, J., Li, M.: Patternhunter: faster and more sensitive homology search. Bioinformatics **18**(3), 440–445 (2002)

[10] Li, M., Ma, B., Kisman, D., Tromp, J.: Patternhunter ii: Highly sensitive and fast homology search. Journal of bioinformatics and computational biology **2**(03), 417–439 (2004)

[11] Ferragina, P., Manzini, G.: An experimental study of a compressed index. Information Sciences **135**(1-2), 13–28 (2001)

[12] Zaharia, M., Bolosky, W.J., Curtis, K., Fox, A., Patterson, D., Shenker, S., Stoica, I., Karp, R.M., Sittler, T.: Faster and More Accurate Sequence Alignment with SNAP. arXiv (2011)

[13] Marco-Sola, S., Sammeth, M., Guigó, R., Ribeca, P.: The GEM mapper: fast, accurate and versatile alignment by filtration. Nature methods **9**(12), 1185–8 (2012)

[14] Keich, U., Li, M., Ma, B., Tromp, J.: On spaced seeds for similarity search. Discrete Applied Mathematics **138**(3), 253–263 (2004)

[15] Needleman, S.B., Wunsch, C.D.: A general method applicable to the search for similarities in the amino acid sequence of two proteins. Journal of molecular biology **48**(3), 443–453 (1970)

[16] Smith, T.F., Waterman, M.S.: Identification of common molecular subsequences. Journal of Molecular Biology **147**(1), 195–197 (1981)

[17] Bille, P.: A survey on tree edit distance and related problems. Theoretical computer science **337**(1-3), 217–239 (2005)

[18] Gotoh, O.: An improved algorithm for matching biological sequences. Journal of molecular biology **162**(3), 705–708 (1982)

[19] Liu, Y., Popp, B., Schmidt, B.: CUSHAW3: sensitive and accurate base-space and color-space short-read alignment with hybrid seeding. PloS one **9**(1), 86869 (2014)

[20] Farrar, M.: Striped Smith-Waterman speeds database searches six times over other SIMD implementations. Bioinformatics **23**(2), 156–161 (2007)

[21] Szalkowski, A., Ledergerber, C., Krähenbühl, P., Dessimoz, C.: SWPS3 - fast multi-threaded vectorized Smith-Waterman for IBM Cell/B.E. and x86/SSE2. BMC research notes **1**, 107 (2008)

[22] Zhao, M., Lee, e.a.: SSW Library: An SIMD Smith-Waterman C/C++ Library for Use in Genomic Applications. PLoS ONE **8**(12), 82138 (2013)

[23] Döring, A., Weese, e.a.: SeqAn An efficient, generic C++ library for sequence analysis. BMC Bioinformatics **9**(1), 11 (2008)

[24] Myers, G.: A fast bit-vector algorithm for approximate string matching based on dynamic programming. Journal of the ACM **46**(3), 395–415 (1999)

[25] Ukkonen, E.: Algorithms for approximate string matching. Information and Control **64**(1), 100–118 (1985)

[26] Zhang, Z., Schwartz, S., Wagner, L., Miller, W.: A greedy algorithm for aligning dna sequences. Journal of Computational biology **7**(1-2), 203–214 (2000)

[27] Liu, Y., Wirawan, A., Schmidt, B.: CUDASW++ 3.0: accelerating Smith-Waterman protein database search by coupling CPU and GPU SIMD instructions. BMC Bioinformatics **14**, 117 (2013)

[28] Harris, B., Jacob, A.C., Lancaster, J.M., Buhler, J., Chamberlain, R.D.: A Banded Smith-Waterman FPGA Accelerator for Mercury BLASTP. In: 2007 International Conference on Field Programmable Logic and Applications, pp. 765–769. IEEE, ??? (2007)

[29] Allred, J., Coyne, J., Lynch, W., Natoli, V., Grecco, J., Morrissette, J.: Smith-Waterman implementation on a FSB-FPGA module using the Intel Accelerator Abstraction Layer. In: 2009 IEEE International Symposium on Parallel & Distributed Processing, pp. 1–4. IEEE, ??? (2009)

[30] DePristo, M.A., Banks, e.a.: A framework for variation discovery and genotyping using next-generation DNA sequencing data. Nature genetics **43**(5), 491–8 (2011)

[31] Rimmer, A., Phan, H., Mathieson, I., Lunter, G., McVean, G.: Platypus: A haplotype-based variant caller for next generation sequence data (2013)

[32] Khan, Z., Bloom, J.S., Kruglyak, L., Singh, M.: A practical algorithm for finding maximal exact matches in large sequence datasets using sparse suffix arrays. Bioinformatics **25**(13), 1609–1616 (2009)

[33] Aluru, S.: Handbook of computational molecular biology. Chapman and Hall/CRC (2005)

[34] Chao, K.-M., Pearson, W.R., Miller, W.: Aligning two sequences within a specified diagonal band. Bioinformatics **8**(5), 481–487 (1992)

[35] 1000 Genomes Project Consortium, .: A global reference for human genetic variation. Nature **526**(7571), 68–74 (2015). doi:10.1038/nature15393

[36] Xin, H., Greth, J., Emmons, J., Pekhimenko, G., Kingsford, C., Alkan, C., Mutlu, O.: Shifted Hamming distance: a fast and accurate SIMD-friendly filter to accelerate alignment verification in read mapping. Bioinformatics (Oxford, England) **31**(10), 1553–60 (2015)

[37] Alser, M., Hassan, H., Xin, H., Ergin, O., Mutlu, O., Alkan, C.: GateKeeper : Enabling Fast Pre-Alignment in DNA Short Read Mapping with a New Streaming Accelerator Architecture (2016). 1604.01789

[38] Stephens, Z.D., Lee, S.Y., Faghri, F., Campbell, R.H., Zhai, C., Efron, M.J., Iyer, R., Schatz, M.C., Sinha, S., Robinson, G.E.: Big data: astronomical or genomical? PLoS biology **13**(7), 1002195 (2015)
